# An Electrosynthesis of 1,3,4‐Oxadiazoles from *N*‐Acyl Hydrazones

**DOI:** 10.1002/chem.202403128

**Published:** 2024-10-30

**Authors:** Luke Chen, James D. F. Thompson, Craig Jamieson

**Affiliations:** ^1^ Medicinal Chemistry GSK Gunnels Wood Road Stevenage SG1 2NY United Kingdom; ^2^ Pure & Applied Chemistry University of Strathclyde Glasgow G1 1XL United Kingdom

**Keywords:** 1,3,4-Oxadiazoles, Electrochemistry, Hydrazones, Indirect electrolysis, Oxidation

## Abstract

The 1,3,4‐oxadiazole is a widely encountered motif in the areas of pharmaceuticals, materials, and agrochemicals. This work has established a mediated electrochemical synthesis of 2,5‐disubstituted 1,3,4‐oxadiazoles from *N*‐acyl hydrazones. Using DABCO as the optimal redox mediator has enabled a mild oxidative cyclisation, without recourse to stoichiometric oxidants. In contrast to previous methods, this indirect electrochemical oxidation has enabled a broad range of substrates to be accessed, with yields of up to 83 %, and on gram scale. The simplicity of the method has been further demonstrated by the development of a one‐pot procedure, directly transforming readily available aldehydes and hydrazides into valuable heterocycles.

## Graphical abstract

The indirect electrochemical oxidative cyclisation of N‐acyl hydrazones offers an efficient approach to access 1,3,4‐oxadiazoles, an important motif in medicinal chemistry. The DABCO‐mediated reaction enables the selective synthesis of a broad range of these valuable heterocycles from readily available aldehydes and hydrazides.

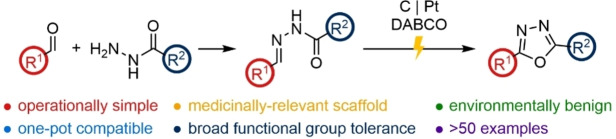

## Introduction

**Scheme 1:**
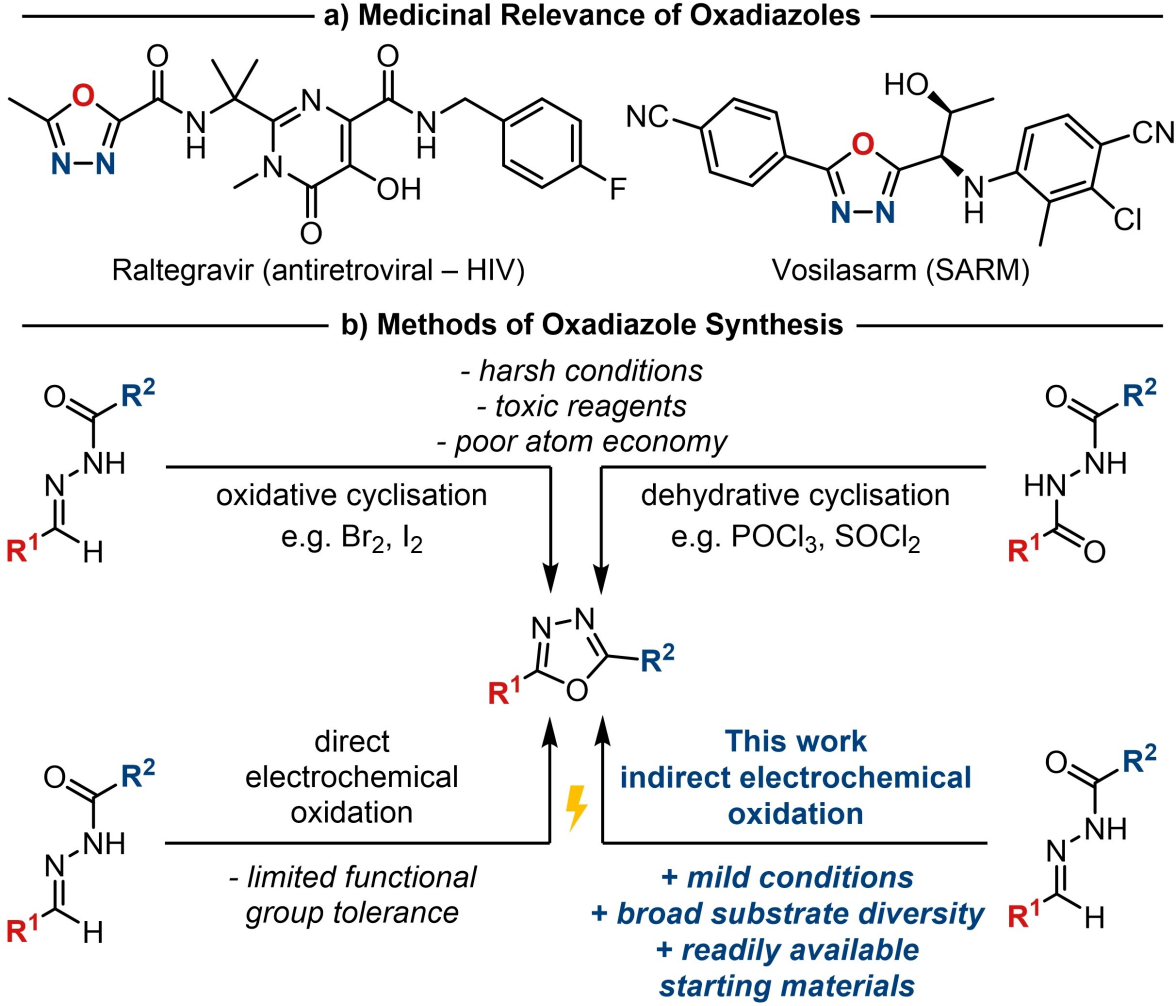
**(**a) Examples of oxadiazole‐containing drugs.[^21^, ^23^] (b) Current approaches to synthesise 2,5‐disubstituted 1,3,4‐oxadiazoles.

The 1,3,4‐oxadiazole is a valuable heterocycle with a range of applications in medicinal chemistry,^[1]^ materials science,[^2^, ^3^, ^4^] and agrochemicals.[^5^, ^6^, ^7^] In the context of drug discovery, the enhanced metabolic stability of this bioisostere of esters and amides can be used to tune pharmacokinetic properties.[^1^, ^8^, ^9^, ^10^] The utility of the oxadiazole is exemplified by its presence in several drug compounds and bioactive molecules,[^11^, ^12^, ^13^, ^14^, ^15^, ^16^, ^17^, ^18^, ^19^, ^20^] including the anti‐HIV drug, raltegravir,[^21^, ^22^] and the selective androgen receptor modulator (SARM), vosilasarm (Scheme 1a).[^23^, ^24^] Given its significance in a broad range of applications, new synthetic methods are needed to address the challenges associated with synthesis. Improved methods to access the oxadiazole motif would, thus, enable more efficient exploration of chemical space in terms of its application, for example, in bioisosterism.

Established synthetic approaches to 1,3,4‐oxadiazoles centre around an oxidative or dehydrative cyclisation of *N*‐acyl hydrazones or diacylhydrazines, respectively (Scheme 1b).[^25^, ^26^, ^27^] Other synthetic methods have also been reported, including photosynthetic approaches,[^28^, ^29^] reactions of hydrazides with nitroalkanes,[^30^, ^31^] the condensation of hydrazides and orthoesters,^[32]^ and the Aza‐Wittig reaction of carboxylic acid analogues with *N*‐isocyaniminotriphenylphosphorane.[^33^, ^34^, ^35^] The majority of these approaches make use of highly reactive and potentially toxic reagents, for example bromine and phosphoryl chloride, which frequently results in limited functional group compatibility. In addition to this, a number of the previously reported approaches lack readily available starting materials or exhibit poor atom economy. Accordingly, there is a need to address these limitations and enable more efficient synthetic access to this privileged scaffold.

Within our laboratories, the reactivity of nitrile imines is a subject of considerable interest, including their application in oxadiazole synthesis.[^28^, ^36^, ^37^, ^38^, ^39^] These reactive intermediates are most commonly generated from tetrazoles or hydrazones and their derivative hydrazonyl halides.[^28^, ^40^] To obviate the use of hazardous reagents and conditions, alternative methods for nitrile imine formation are, therefore, of interest. An attractive approach is an electrochemical oxidation to replace expensive and hazardous oxidants.[^41^, ^42^, ^43^, ^44^] This approach offers mild conditions with improved functional group tolerance. In addition, excess waste can be eliminated to facilitate a safer, more sustainable, and efficient chemical transformation.

The utility of direct electrochemical oxidation methods to cyclise *N*‐acyl hydrazones into 1,3,4‐oxadiazoles has been demonstrated by Chiba and Okimoto,^[45]^ as well as Singh and co‐workers (Scheme 1b).[^46^, ^47^, ^48^, ^49^] However, the success of a direct electrolysis is highly dependent on the inherent redox potential of the starting material.[^50^, ^51^] As such, high potentials may be required for the desired redox event to occur, which significantly restricts the diversity of compatible substrates. Nonetheless, these methodologies provide useful proof of concept to exemplify the application of electrochemistry in the synthesis of oxadiazoles.

The alternative to direct electrolysis is an indirect approach involving the use of a redox mediator.^[52]^ Such mediators typically exhibit lower redox potentials, therefore, the applied potential can be lowered, furnishing greater functional group compatibility. Examples of the use of indirect electrolysis for heterocycle synthesis include Holman's method to synthesise isoxazolines from oximes, and Waldvogel's approach to convert hydrazones into pyrazolines and pyrazoles.^[53]^ These methodologies demonstrate the successful use of halides as mediators to achieve the desired reactivity under mild conditions. Accordingly, in the current study we describe a protocol for a mediated, electrochemical synthesis of the valuable 1,3,4‐oxadiazole cores from *N*‐acyl hydrazones under mild reaction conditions which is tolerant of a raft of functional groups of relevance to medicinal chemistry.

## Results and Discussion

### Reaction Optimisation

**Figure 1:**
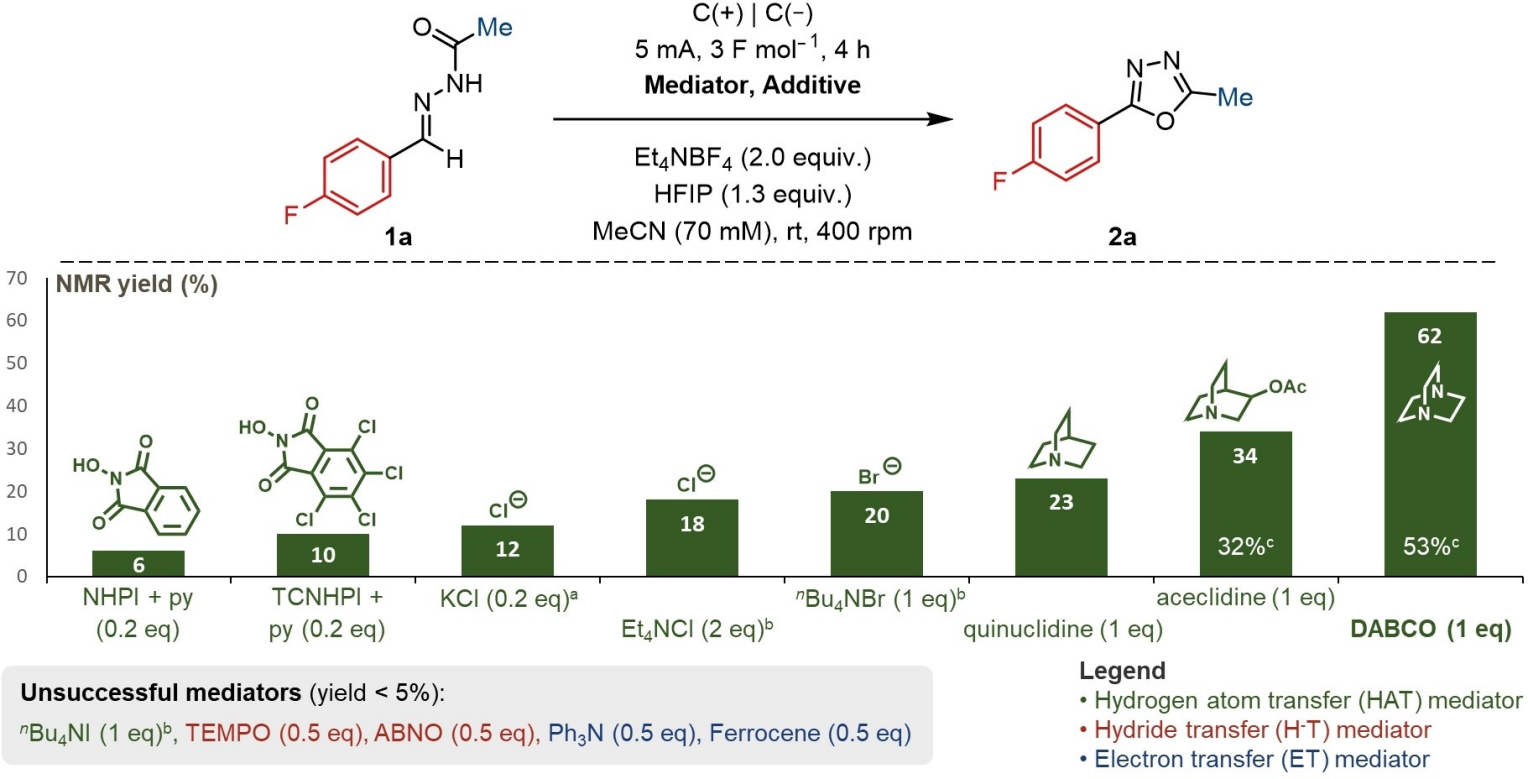
Mediator screen for the optimisation of oxadiazole synthesis. Yields determined by ^19^F NMR spectroscopy. (a) In addition to Et_4_NBF_4_; (b) Instead of Et_4_NBF_4_; (c) isolated yield. C=graphite, NHPI=*N*‐hydroxyphthalimide, py=pyridine,. TCNHPI=*N*‐hydroxytetrachlorophthalimide.

From consideration of work elsewhere in our laboratories on the electrochemical synthesis of isoxazolines from oxime precursors,^[54]^ we initially sought to explore the role of mediators in the process.^[55]^ This made use of graphite electrodes, an ammonium‐based electrolyte, acetonitrile, 1,1,1,3,3,3‐hexafluoroisopropanol (HFIP), and the IKA ElectraSyn system.^[41]^ Using this approach, several common mediators were screened (Figure 1). Hydrogen‐atom transfer (HAT) mediators afforded the greatest success, while hydride and electron transfer mediators were otherwise incompatible. Of these, the readily available and inexpensive 1,4‐diazabicyclo[2.2.2]octane (DABCO) furnished the best conversions as determined by an NMR assay.

Having identified DABCO as the most suitable redox mediator for this reaction, other parameters were subsequently examined (Table 1). When considering the electrode material (Table 1, Entries 1–4), it was found that the pairing of a graphite anode with a platinum cathode gave an improvement in the yield (Table 1, Entry 1). This is likely related to the lower hydrogen overpotential of platinum compared to carbon which may help to limit unwanted reductive side reactions.^[56]^ Different electrolytes were also tested, and these were generally tolerated and had a minimal effect on the reaction (Table 1, Entries 5–8). Unsurprisingly, an exception was when the electrolyte was poorly soluble, such as in the case of the inorganic lithium perchlorate (Table 1, Entry 8). With solubility being an important parameter, various solvents were also investigated. The majority of these were not tolerated, including dimethyl sulfoxide (DMSO), *N*,*N*‐dimethylformamide (DMF), dichloromethane (DCM), and ethyl acetate (SI, Table S2). Environmentally benign solvents, such as methanol and acetone, were otherwise comparable to acetonitrile and could be used as alternatives (Table 1, Entries 9 and 10).

**Table 1:**
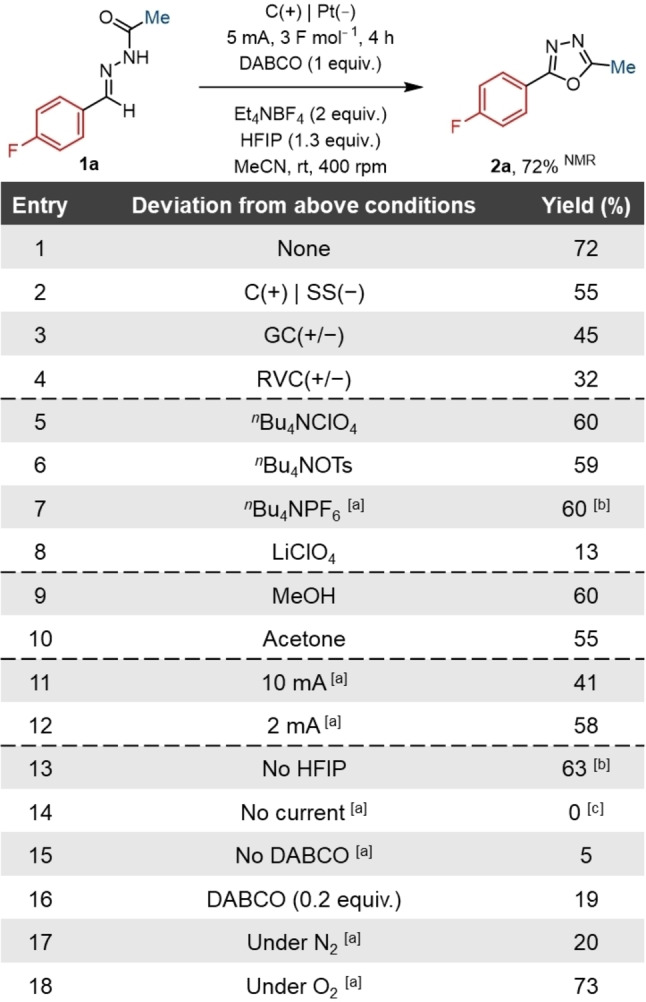
Optimisation of reaction parameters. Yields determined by ^19^F NMR spectroscopy.

[a] No HFIP, electrolyte (1 equiv.). [b] isolated yield. [c] determined by LCMS. SS=stainless steel, GC=glassy carbon, RVC=reticulated vitreous carbon.

The initial screening conditions employed HFIP as an additive which may play multiple roles in the reaction. It has been reported that HFIP can help promote reactions by stabilising ionic and radical intermediates, and by enhancing solubility and conductivity.[^54^, ^57^, ^58^] Furthermore, the oxidative reaction requires a reductive process to close the redox cycle, and this is often the reduction of protons to hydrogen gas.^[41]^ The relatively acidic alcohol can, therefore, serve as a proton source to facilitate this reduction. Other additives that can act as an electron sink were evaluated, including acetic acid, silver nitrate, and dichloromethane, however, no benefit was observed (SI, Table S2).[^59^, ^60^] To gain a better understanding of whether DABCO could also be behaving as a base in the reaction, sodium acetate, triethylamine, and pyridine were also investigated as basic additives (SI, Table S2). Although none of these additives furnished improved yields, it was determined that HFIP was not required, and could be removed without detriment to the reaction (Table 1, Entry 13).

Increasing the current applied to the reaction was associated with greater side‐product formation (Table 1, Entry 11). Conversely, milder conditions could be achieved by decreasing the current, however, a lower current extends the reaction time, and this had no beneficial effects on the yield (Table 1, Entry 12). Although use of an oxygen atmosphere gave a slightly enhanced yield (Table 1, Entry 18), the benefit was marginal, and in the interests of operational simplicity, this was not employed subsequently. Accordingly, the optimum electrochemical conditions utilised a constant current of 5 mA with a total charge of 3 F mol^−1^, which was sufficient for the reaction to proceed to completion.

### Substrate Scope

**Scheme 2:**
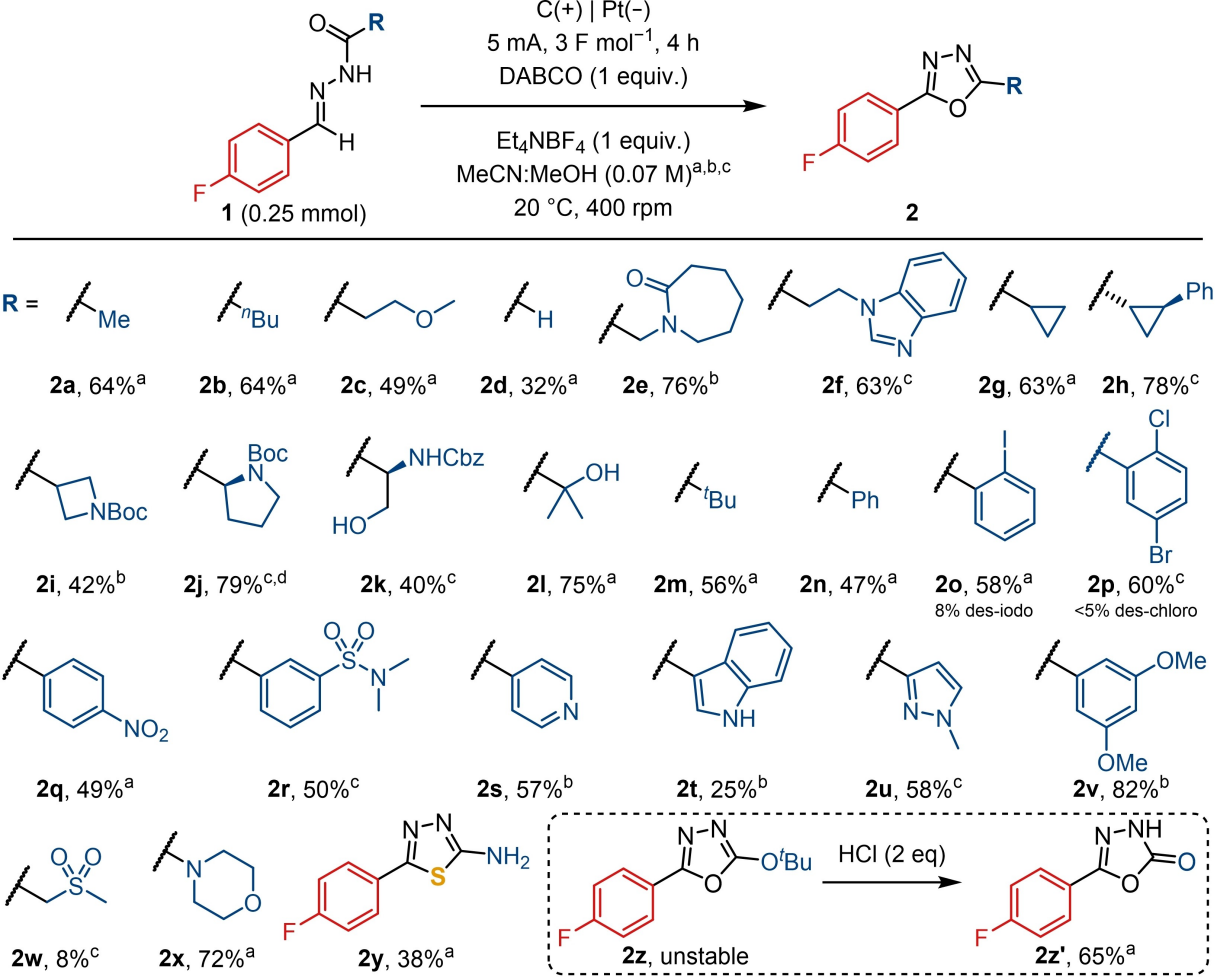
Hydrazide scope. Yields refer to isolated yields. (a) MeCN. (b) MeCN : MeOH 2 : 1. (c) MeCN : MeOH 1 : 2. (d) 5 F mol^−1^.

**Scheme 3:**
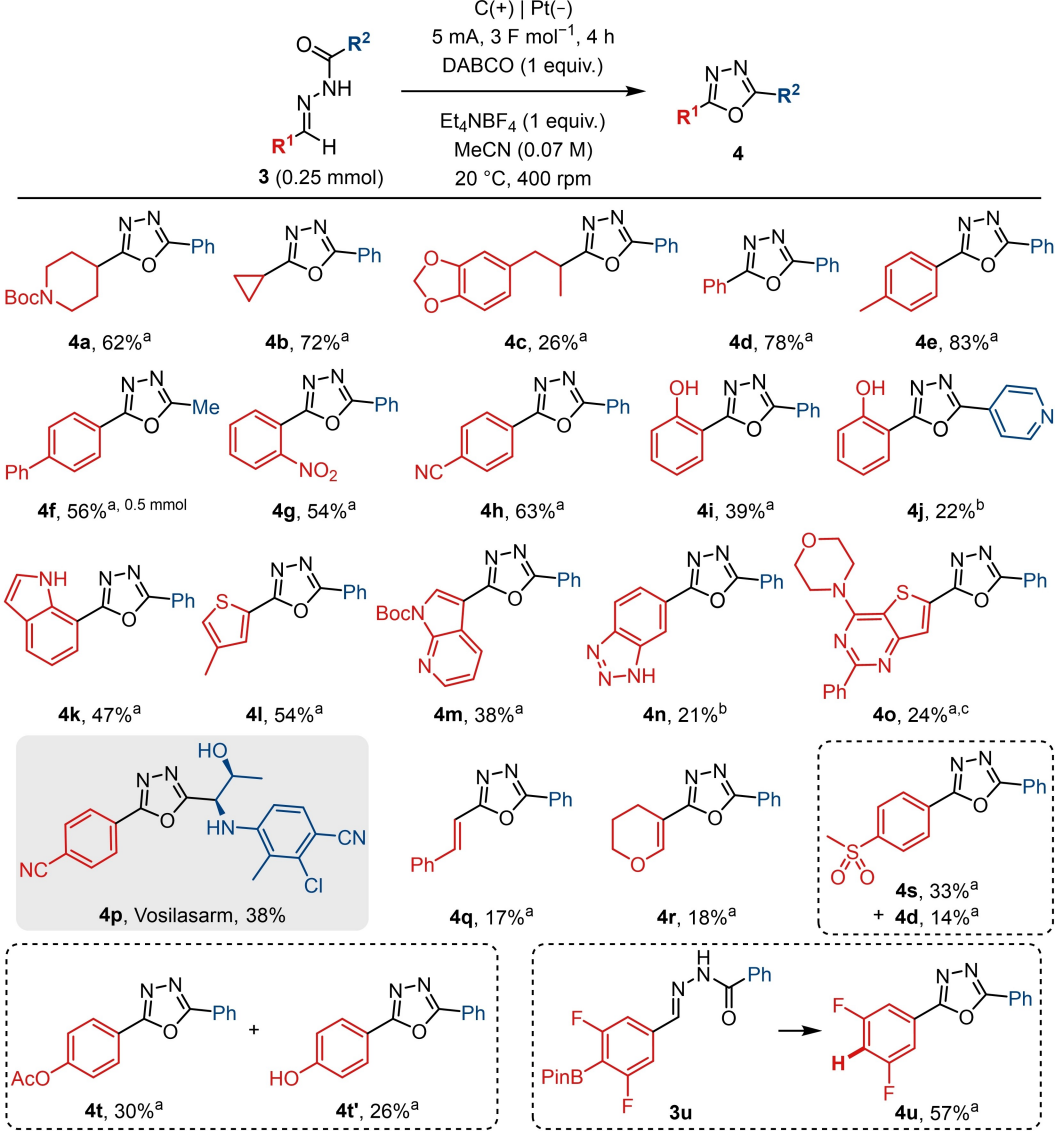
Aldehyde scope. Yields refer to isolated yields. (a) MeCN; (b) MeCN : MeOH 36 : 1; (c) reaction incomplete after 7 F mol^−1^.

**Scheme 4:**
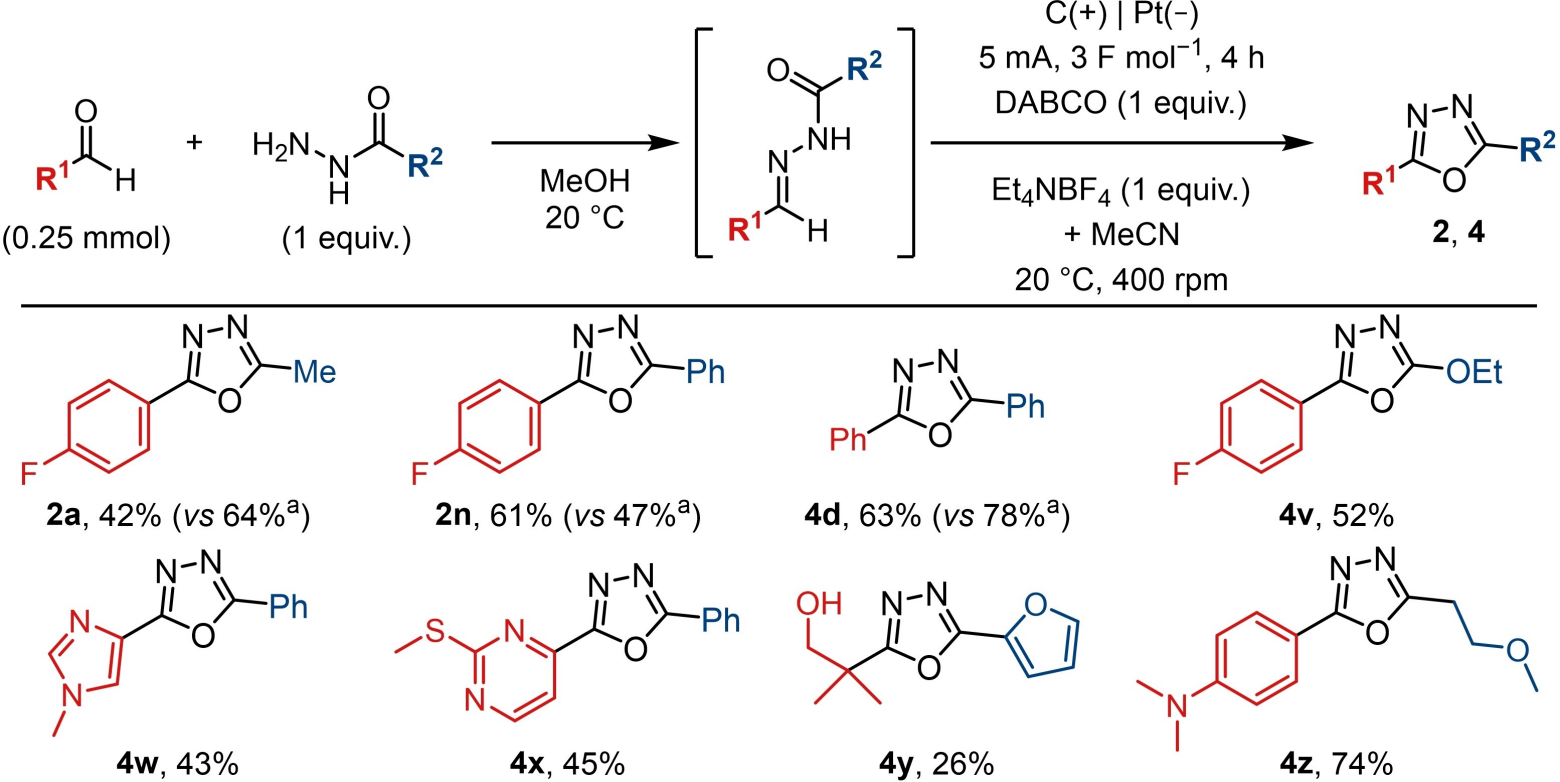
One‐pot reaction scope. Yields refer to the isolated yield over two steps, without isolation of the hydrazone. Exact conditions for the hydrazone formation.n step may vary and are substrate dependent. (a) Yields when the reaction was performed using the isolated hydrazone, for comparison.

Having established the reaction conditions for the electrooxidation, the scope of the reaction was examined (Schemes 2–4). Although the solvent was most typically acetonitrile, in some cases a mixture of acetonitrile and methanol was used to aid the solubility of certain hydrazone substrates. Standard, laboratory grade solvents were employed, and the exclusion of air was not required. In considering changes to the hydrazide, a diverse range of functional groups was shown to be compatible, with moderate to good yields obtained in most cases (Scheme 2). This included unprotected alcohols (**2 k**, **2 l**) which would otherwise not be tolerated under the oxidative or dehydrative conditions traditionally employed in oxadiazole synthesis. Similarly, oxadiazoles bearing heterocycles, such as benzimidazole, pyridine, and pyrazole (**2 f**, **2 s**, **2 u**), could be successfully synthesised. Phenols, indoles, and other electron rich aromatics are frequently challenging substrates for electrochemical reactions as they are susceptible to oxidation.[^61^, ^62^] It was, therefore, pleasing to note that electron‐rich substrates were tolerated (**2 v**, **2 t**). Similarly, products **2 q** and **2 r** demonstrate the compatibility of the reaction with electron deficient substituents, including the nitro group which can be unstable during electrolysis.^[53]^

Hydrazones bearing aryl halides (**2 o**, **2 p**) could also undergo effective cyclisation, with only trace amounts of the dehalogenated compounds detected. In the case where a thioacyl hydrazone was used as the starting material, the equivalent thiadiazole (**2 y**) could be prepared. Similarly, the urea and carbonate analogues were also compatible for the synthesis of *N*‐ and *O*‐linked oxadiazoles (**2 x**, **2 z**). Unfortunately, compound **2 z** was unstable and was found to decompose during purification. The removal of the *tert*‐butoxy group was, therefore, carried out after the electrolysis had taken place to furnish the oxadiazolone **2 z’**, which may be a useful strategy to access this carboxylic acid bioisostere.[^63^, ^64^] Similarly, substrates bearing an acidic proton adjacent to the oxadiazole were also unstable and led to no product or low yields, as in the case of **2 w**. The instability of these acidic α‐H‐containing oxadiazoles has previously been observed by Barker and co‐workers.^[65]^

Subsequently, the scope of the aldehyde component was examined with similar results being observed for a variety of alkyl and aryl substituents (Scheme 3). Phenols (**4 i**, **4 j**) were again tolerated, together with other electron rich heterocycles, including the indole (**4 k**), thiophene (**4 l**), as well as other more complex and heteroatom‐rich aromatics (**4 m**, **4 n**, **4 o**), which are of pharmaceutical relevance.

Less successful substrates included hydrazones bearing alkenyl groups (**4 q**, **4 r**). The current study is likely to involve a nitrile imine intermediate, therefore, it is possible that the lower yields observed for the conjugated vinyl‐containing substrates are due to the alkene moiety reacting with the incipient 1,3‐dipole. An example of this type of intramolecular 1,3‐dipolar cycloaddition reaction was reported by Waldwogel and co‐workers in their electrochemical pyrazoline synthesis.^[53]^ In the case of the acetyl‐protected phenol **4 t**, due to the instability of the ester group, deacetylation occurred and the unprotected phenol product **4 t’** was isolated separately.

Where the substrate contains an aryl sulfone, the desired product (**4 s**) was isolated in addition to the desulfonylated product (**4 d**). This observation was unsurprising given that aryl sulfones have been known to undergo reductive cleavage under electrochemical conditions.[^66^, ^67^, ^68^, ^69^] Similarly, the fully protodeboronated oxadiazole **4 u** was the sole product obtained when the reaction was performed on a hydrazone bearing a boronate ester (**3 u**). This may be due to the instability of the starting material which underwent both hydrolysis and protodeboronation during LCMS analysis. Additionally, it is possible for oxidative cleavage of this redox active boronate group to occur, a process which has been reported under photochemical conditions.[^70^, ^71^]

Furthermore, the methodology was successfully applied to the synthesis of vosilasarm (**4 p**, RAD‐140), a selective androgen receptor modulator.^[23]^ As the reaction is significantly milder compared to traditional approaches, protection and deprotection steps could be avoided which were otherwise present in the established synthesis of the drug. This example showcases the utility of this electrochemical reaction in medicinal chemistry and the potential usefulness for forming the oxadiazole in the final step for exploring changes to the cyanophenyl ring.

Employing a one‐pot, two‐step process would further enhance the efficiency and simplicity of the reaction, and it was established that hydrazone condensation can be performed in methanol or acetonitrile, which are compatible with the electrochemical step.[^53^, ^72^] Accordingly, for a given substrate which is compatible with the electrooxidation, the intermediate hydrazone could be taken directly into the electrolysis without isolation (Scheme 4). Comparable yields to the two‐step procedure were observed for **2 a**, **2 n**, and **4 d**. An additional selection of substrates showed that the functional group tolerance was equally broad. This included various heterocycles (**4 w**, **4 x**, **4 y**), a thioether (**4 x**), and an aniline (**4 z**). Having established a one‐pot procedure, it is therefore straightforward to convert commercially available aldehydes and hydrazides into a wide range of oxadiazoles.

Our emerging approach was then compared with the methodology of Singh and co‐workers who reported a yield of 76 % for their synthesis of oxadiazole **4 e**.^[46]^ Their reaction conditions were replicated on the ElectraSyn using the same electrode material, electrolyte, solvent, and constant potential, however, the scale had to be reduced from 10 mmol–0.42 mmol. This reaction resulted in a significantly lower yield of 14 %, compared to the reported 76 % as well as 83 % from the DABCO‐mediated reaction. These differences in yields highlight the mildness and greater selectivity of the indirect electrolysis, as well as the advantage of using a standardised electrochemical reactor for enhanced reproducibility.

### Control Experiments and Mechanistic Analysis

Control experiments were conducted to establish a greater understanding of the reaction. It was possible to successfully scale‐up the batch synthesis of **2 a** to a 1 mmol scale (Table 2, Entry 1). Upon scaling the reaction to 1 g, a significantly higher concentration was necessary due to the volume constraints of the ElectraSyn reaction vessel and unfortunately, this led to a reduction in yield (Table 2, Entry 2). Furthermore, maintaining the same current density with electrodes of the same size subsequently results in a significantly longer reaction time. Nonetheless, the reaction can be performed on a synthetically useful scale. Further scale‐up may benefit from other, larger electrochemical reactors or by making use of flow electrochemistry.[^53^, ^73^]

**Table 2:**
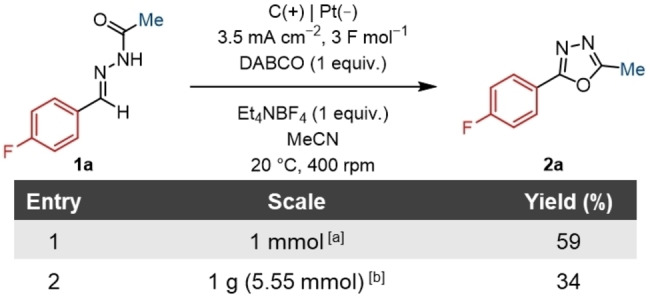
Scale‐up of the synthesis of **1 a** from 0.25 mmol to gram scale.

[a] Volume=14.4 mL, 70 mM, electrode surface area=2 cm^2^, 7 mA, total charge=3 F mol^−1^, time=11.5 h. [b] volume=16 mL, 347 mM, electrode surface area=3.04 cm^2^, 10.6 mA, total charge=4.5 F mol^−1^, time=83.5 h.

**Figure 2:**
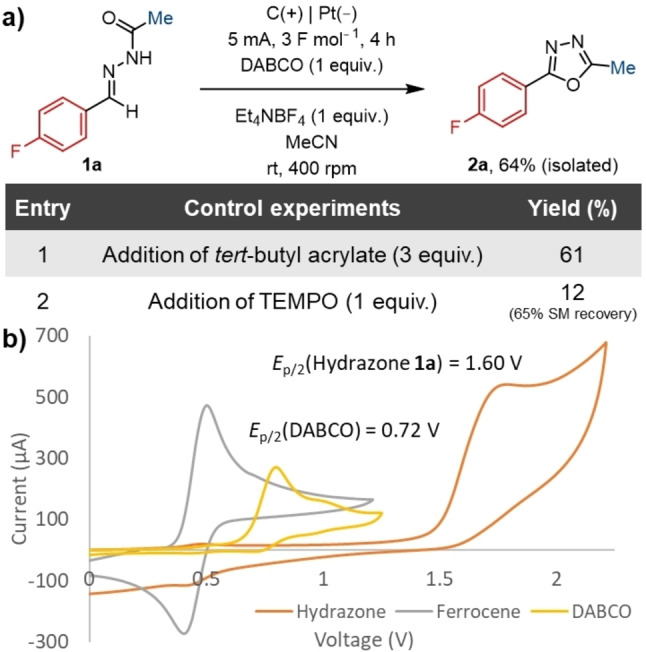
**(**a) Control experiments. Isolated yields. Yields determined by ^19^F NMR spectroscopy. SM=starting material. (b) Cyclic voltammograms of select mediators and hydrazone **1 a**.

In the absence of the DABCO mediator, product formation was significantly reduced (Table 1, Entry 15). Comparing the oxidation potential of DABCO and the model hydrazone **1 a** shows a substantial difference in the voltage required for oxidation (Figure 2b). This underpins the advantage of mediated electrolysis to enable milder conditions. The trace amount of product is likely the result of direct electrolysis, however, a range of unidentifiable impurities were also observed due to the poor selectivity of the direct oxidation. Similarly, a lower yield is observed when the stoichiometry of the mediator is changed from 1–0.2 equivalents (Table 1, Entry 16). This implies that DABCO is not catalytic under these reaction conditions.

Additionally, the exclusion of air by performing the reaction inside a glovebox under nitrogen led to a reduction in yield (Table 1, Entry 17). Conversely, the use of an O_2_ balloon saw an increase in product formation (Table 1, Entry 18), and this dependency on the oxygen content would suggest that oxygen plays an important role in the reaction. When no current is passed through the reaction mixture, no change to the starting material was observed by LCMS analysis (Table 1, Entry 14). Electricity and the mediator are, therefore, still crucial as simply stirring the mixture under air does not result in product formation. Although the enrichment of oxygen was shown to be beneficial, this process was excluded from the standard protocol when investigating the substrate scope to ensure that the approach was operationally simple and accessible. For more challenging substrates, however, it may be worthwhile to make use of an oxygen atmosphere.

Radical trapping experiments were performed using *tert*‐butyl acrylate and TEMPO. In the case where the acrylate was added, the yield was not affected by the addition of this radical trap (Figure 2a, Entry 1). This would imply that no radical species that could be trapped by the acrylate are formed. When TEMPO was added to the reaction, the yield was significantly reduced (Figure 2a, Entry 2). A possible explanation is that the *N*‐oxyl radical can be oxidised and then behave as a mediator. Consequently, TEMPO may be outcompeting DABCO for the anodic oxidation, given that it has a lower oxidation potential.[^39^, ^45^] This in turn inhibits productive reaction, and would account for the limited starting material consumption. Substrates bearing cyclopropyl groups (**2 g**, **2 h**, **4 b**) could be successfully synthesised, without the observation of ring opened products, and therefore, this suggests that any radical formed is short‐lived, and formation of the key nitrile imine intermediate occurs rapidly.

**Scheme 5:**
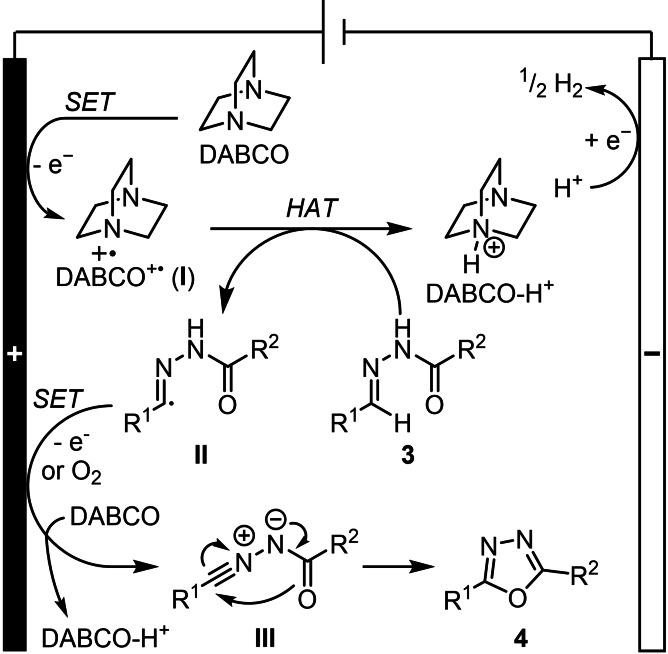
Proposed reaction mechanism.

Based on all the above, the following mechanism has been proposed (Scheme 5). The reaction begins with the initial single electron oxidation of DABCO at the anode. This generates a radical cation (**I**) which can abstract a hydrogen atom from the hydrazone **3** to form a radical species (**II**). Subsequent oxidation of the radical intermediate **II** can take place at the anode or *via* oxygen, given its putative role in the reaction. This is coupled by a deprotonation to form nitrile imine **III** which can then undergo a 1,5‐electrocyclisation to afford the product oxadiazole **4**.

## Conclusions

In conclusion, a facile synthesis of 2,5‐disubstituted 1,3,4‐oxadiazoles from *N*‐acyl hydrazones using an indirect, electrochemical oxidation has been developed. The reaction is amenable to a one‐pot process, significantly enhancing the accessibility of oxadiazoles directly from commercially available aldehyde and hydrazide precursors and on preparatively useful scales. This approach has enabled the development of mild conditions and is tolerant of a diverse range of medicinally relevant functional groups. The application of the methodology for the synthesis of drug compounds, such as vosilasarm (**4 p**), was demonstrated without the need to use protecting group strategies. Additionally, the reaction is operationally straightforward using a commercially available and standardised electrochemical system without requiring moisture exclusion or an inert atmosphere. Work is ongoing to establish the reactivity of hydrazones under electrochemical conditions to access other motifs of importance in drug discovery.

## Experimental

General procedures for 1,3,4‐oxadiazole synthesis are given below. For full details and characterisation data, see Supporting Information.

### General Procedure for Oxadiazole Synthesis from Isolated Hydrazones

To a 5 mL ElectraSyn 2.0 reaction vessel containing a stirrer bar, tetraethylammonium tetrafluoroborate (54.4 mg, 0.25 mmol, 1 equiv.), DABCO (28.1 mg, 0.25 mmol, 1 equiv.), and hydrazone (0.25 mmol., 1 equiv.) were added, followed by acetonitrile (3.6 mL). The reaction mixture was electrolysed under a constant current of 5 mA at room temperature with a graphite anode and a platinum foil cathode, stirring at 400 rpm until a total charge of 3 F mol^−1^ had been passed. The electrodes were rinsed with MeOH into the reaction mixture which was concentrated *in vacuo* and purified by column chromatography.

### General Procedure for One‐Pot Oxadiazole Synthesis

To a 5 mL ElectraSyn 2.0 reaction vessel containing a stirrer bar, aldehyde (0.25 mmol, 1 equiv.), hydrazide (0.25 mmol, 1 equiv.), and MeOH (1.2 mL) were added. The reaction mixture was stirred until hydrazone formation was complete as monitored by LCMS. Tetraethylammonium tetrafluoroborate (54.4 mg, 0.25 mmol, 1 equiv.), DABCO (28.1 mg, 0.25 mmol, 1 equiv.), and acetonitrile (2.4 mL) were added. The reaction mixture was electrolysed under a constant current of 5 mA at room temperature (20–21 °C) with a graphite anode and a platinum foil cathode, stirring at 400 rpm until a total charge of 3 F mol^−1^ had been passed. The electrodes were rinsed with MeOH (~2 mL) into the reaction mixture and the reaction mixture was concentrated *in vacuo* and purified by column chromatography.

## Conflict of Interests

The authors declare no conflict of interest.

1

## Supporting information

As a service to our authors and readers, this journal provides supporting information supplied by the authors. Such materials are peer reviewed and may be re‐organized for online delivery, but are not copy‐edited or typeset. Technical support issues arising from supporting information (other than missing files) should be addressed to the authors.

Supporting Information

## Data Availability

The data that support the findings of this study are available in the supplementary material of this article.

## References

[1] J. Boström , A. Hogner , A. Llinàs , E. Wellner , A. T. Plowright , J. Med. Chem. 2012, 55, 1817–1830. 22185670 10.1021/jm2013248

[2] S. Gong , Y. Chen , X. Zhang , P. Cai , C. Zhong , D. Ma , J. Qin , C. Yang , J. Mater. Chem. 2011, 21, 11197.

[3] S. Chidirala , H. Ulla , A. Valaboju , M. R. Kiran , M. E. Mohanty , M. N. Satyanarayan , G. Umesh , K. Bhanuprakash , V. J. Rao , J. Org. Chem. 2016, 81, 603–614. 26651353 10.1021/acs.joc.5b02423

[4] A. Paun , N. D. Hadade , C. C. Paraschivescu , M. Matache , J. Mater. Chem. C 2016, 4, 8596–8610.

[5] L. Zhong , C. Wu , M. Li , J. Wu , Y. Chen , Z. Ju , C. Tan , Org. Biomol. Chem. 2023, 21, 7511–7524. 37671568 10.1039/d3ob00934c

[6] D. Zeng , S.-S. Liu , W.-B. Shao , T.-H. Zhang , P.-Y. Qi , H.-W. Liu , X. Zhou , L.-W. Liu , H. Zhang , S. Yang , J. Agric. Food Chem. 2023, 71, 2804–2816. 36744848 10.1021/acs.jafc.2c07486

[7] J. Ji , W.-B. Shao , P.-L. Chu , H.-M. Xiang , P.-Y. Qi , X. Zhou , P.-Y. Wang , S. Yang , J. Agric. Food Chem. 2022, 70, 7929–7940. 35731909 10.1021/acs.jafc.2c01988

[8] J. R. Frost , C. C. G. Scully , A. K. Yudin , Nat. Chem. 2016, 8, 1105–1111. 27874866 10.1038/nchem.2636

[9] D. Ramsbeck , M. Buchholz , B. Koch , L. Böhme , T. Hoffmann , H. U. Demuth , U. Heiser , J. Med. Chem. 2013, 56, 6613–6625. 23886302 10.1021/jm4001709

[10] K. Goldberg , S. Groombridge , J. Hudson , A. G. Leach , P. A. MacFaul , A. Pickup , R. Poultney , J. S. Scott , P. H. Svensson , J. Sweeney , MedChemComm 2012, 3, 600–604.

[11] D. Tiwari , R. Narang , K. Sudhakar , V. Singh , S. Lal , M. Devgun , Chem. Biol. Drug Des. 2022, 100, 1086–1121. 35676800 10.1111/cbdd.14100

[12] C. Steeneck , C. Gege , O. Kinzel , M. Albers , G. Kleymann , T. Schlüter , A. Schulz , X. Xue , M. D. Cummings , A. M. Fourie , K. A. Leonard , B. Scott , J. P. Edwards , T. Hoffmann , S. D. Goldberg , Bioorg. Med. Chem. Lett. 2020, 30, 127174. 32334912 10.1016/j.bmcl.2020.127174

[13] A. A. Othman , M. Kihel , S. Amara , Arab. J. Chem. 2019, 12, 1660–1675.

[14] R. Knegtel , J.-D. Charrier , S. Durrant , C. Davis , M. O'Donnell , P. Storck , S. MacCormick , D. Kay , J. Pinder , A. Virani , H. Twin , M. Griffiths , P. Reaper , P. Littlewood , S. Young , J. Golec , J. Pollard , J. Med. Chem. 2019, 62, 5547–5561. 31074988 10.1021/acs.jmedchem.9b00426

[15] P. Pitasse-Santos , V. Sueth-Santiago , M. Lima , J. Braz. Chem. Soc. 2018, 29, 435–456.

[16] K. Nakajima , R. Chatelain , K. B. Clairmont , R. Commerford , G. M. Coppola , T. Daniels , C. J. Forster , T. A. Gilmore , Y. Gong , M. Jain , A. Kanter , Y. Kwak , J. Li , C. D. Meyers , A. D. Neubert , P. Szklennik , V. Tedesco , J. Thompson , D. Truong , Q. Yang , B. K. Hubbard , M. H. Serrano-Wu , J. Med. Chem. 2017, 60, 4657–4664. 28498655 10.1021/acs.jmedchem.7b00173

[17] A. Zarghi , Z. Hajimahdi , Expert Opin. Ther. Pat. 2013, 23, 1209–1232. 23663160 10.1517/13543776.2013.797409

[18] S. Bajaj , V. Asati , J. Singh , P. P. Roy , Eur. J. Med. Chem. 2015, 97, 124–141. 25965776 10.1016/j.ejmech.2015.04.051

[19] S. Vardan , H. Smulyan , S. Mookherjee , R. Eich , Clin. Pharmacol. Ther. 1983, 34, 290–296. 6883905 10.1038/clpt.1983.170

[20] S. Naseem , A. Temirak , A. Imran , S. Jalil , S. Fatima , P. Taslimi , J. Iqbal , M. Tasleem , M. N. Tahir , Z. Shafiq , RSC Adv. 2023, 13, 17526–17535. 37304812 10.1039/d3ra01953e PMC10253498

[21] V. Summa , A. Petrocchi , F. Bonelli , B. Crescenzi , M. Donghi , M. Ferrara , F. Fiore , C. Gardelli , O. Gonzalez Paz , D. J. Hazuda , P. Jones , O. Kinzel , R. Laufer , E. Monteagudo , E. Muraglia , E. Nizi , F. Orvieto , P. Pace , G. Pescatore , R. Scarpelli , K. Stillmock , M. V. Witmer , M. Rowley , J. Med. Chem. 2008, 51, 5843–5855. 18763751 10.1021/jm800245z

[22] Z. Wang , M. Wang , X. Yao , Y. Li , W. Qiao , Y. Geng , Y. Liu , Q. Wang , Eur. J. Med. Chem. 2012, 50, 361–369. 22369862 10.1016/j.ejmech.2012.02.015

[23] C. P. Miller , M. Shomali , C. R. Lyttle , L. S. L. O'Dea , H. Herendeen , K. Gallacher , D. Paquin , D. R. Compton , B. Sahoo , S. A. Kerrigan , M. S. Burge , M. Nickels , J. L. Green , J. A. Katzenellenbogen , A. Tchesnokov , G. Hattersley , ACS Med. Chem. Lett. 2011, 2, 124–129. 24900290 10.1021/ml1002508 PMC4018048

[24] A. H. Tien , M. D. Sadar , Int. J. Mol. Sci. 2024, 25, 1817.38339092

[25] R. M. El-Masry , H. H. Kadry , A. T. Taher , S. M. Abou-Seri , Molecules 2022, 27, 2709. 35566059 10.3390/molecules27092709 PMC9102899

[26] K. D. Patel , S. M. Prajapati , S. N. Panchal , H. D. Patel , Synth. Commun. 2014, 44, 1859–1875.

[27] C. S. de Oliveira , B. F. Lira , J. M. Barbosa-Filho , J. G. F. Lorenzo , P. F. de Athayde-Filho , Molecules 2012, 17, 10192–10231. 22926303 10.3390/molecules170910192 PMC6268307

[28] L. Green , K. Livingstone , S. Bertrand , S. Peace , C. Jamieson , Chem. Eur. J. 2020, 26, 14866–14870. 32786060 10.1002/chem.202002896 PMC7756889

[29] J.-L. Li , H.-Y. Li , S.-S. Zhang , S. Shen , X.-L. Yang , X. Niu , J. Org. Chem. 2023, 88, 14874–14886. 37862710 10.1021/acs.joc.3c01078

[30] K. Tokumaru , J. N. Johnston , Chem. Sci. 2017, 8, 3187–3191. 28507694 10.1039/c7sc00195a PMC5414388

[31] A. V. Aksenov , V. Khamraev , N. A. Aksenov , N. K. Kirilov , D. A. Domenyuk , V. A. Zelensky , M. Rubin , RSC Adv. 2019, 9, 6636–6642. 35518500 10.1039/c9ra00976k PMC9060929

[32] K. K. Gnanasekaran , B. Nammalwar , M. Murie , R. A. Bunce , Tetrahedron Lett. 2014, 55, 6776–6778.

[33] D. Matheau-Raven , D. J. Dixon , J. Org. Chem. 2022, 87, 12498–12505. 36054913 10.1021/acs.joc.2c01669 PMC9486941

[34] A. Ramazani , A. Rezaei , Org. Lett. 2010, 12, 2852–2855. 20481612 10.1021/ol100931q

[35] D. Matheau-Raven , D. J. Dixon , Angew. Chem. Int. Ed. 2021, 60, 19725–19729. 10.1002/anie.202107536 PMC8457168 34191400

[36] S. Stewart , R. Harris , C. Jamieson , Synlett 2014, 25, 2480–2484.

[37] M. Boyle , K. Livingstone , M. C. Henry , J. M. L. Elwood , J. D. Lopez-Fernandez , C. Jamieson , Org. Lett. 2022, 24, 334–338. 34964648 10.1021/acs.orglett.1c03993 PMC8762704

[38] J. M. L. Elwood , M. C. Henry , J. D. Lopez-Fernandez , J. M. Mowat , M. Boyle , B. Buist , K. Livingstone , C. Jamieson , Org. Lett. 2022, 24, 9491–9496. 36524745 10.1021/acs.orglett.2c03971 PMC9806851

[39] M. C. Henry , L. Minty , A. C. W. Kwok , J. M. L. Elwood , A. J. Foulis , J. Pettinger , C. Jamieson , J. Org. Chem. 2024, 89, 7913–7926. 38778786 10.1021/acs.joc.4c00575 PMC11165588

[40] R. Yang , L. Dai , J. Org. Chem. 1993, 58, 3381–3383.

[41] C. Kingston , M. D. Palkowitz , Y. Takahira , J. C. Vantourout , B. K. Peters , Y. Kawamata , P. S. Baran , Acc. Chem. Res. 2020, 53, 72–83. 31823612 10.1021/acs.accounts.9b00539 PMC6996934

[42] G. Hilt , ChemElectroChem 2020, 7, 395–405.

[43] C. Zhu , N. W. J. Ang , T. H. Meyer , Y. Qiu , L. Ackermann , ACS Cent. Sci. 2021, 7, 415–431. 33791425 10.1021/acscentsci.0c01532 PMC8006177

[44] C. Schotten , T. P. Nicholls , R. A. Bourne , N. Kapur , B. N. Nguyen , C. E. Willans , Green Chem. 2020, 22, 3358–3375.

[45] T. Chiba , M. Okimoto , J. Org. Chem. 1992, 57, 1375–1379.

[46] S. Singh , L. K. Sharma , A. Saraswat , I. R. Siddiqui , H. K. Kehri , R. K. P. Singh , RSC Adv. 2013, 3, 4237–4245.

[47] L. K. Sharma , S. Kumar , S. Singh , R. K. P. Singh , Russ. J. Electrochem. 2010, 46, 34–40.

[48] L. K. Sharma , A. Saraswat , S. Singh , M. K. Srivastav , R. K. P. Singh , Proc. Natl. Acad. Sci. India Sect. A 2015, 85, 29–34.

[49] S. Singh , L. K. Sharma , A. Saraswat , I. R. Siddiqui , R. K. P. Singh , Russ. J. Electrochem. 2014, 50, 831–837.

[50] Y. Kawamata , M. Yan , Z. Liu , D.-H. Bao , J. Chen , J. T. Starr , P. S. Baran , J. Am. Chem. Soc. 2017, 139, 7448–7451. 28510449 10.1021/jacs.7b03539 PMC5465511

[51] A. J. J. Lennox , J. E. Nutting , S. S. Stahl , Chem. Sci. 2018, 9, 356–361. 29732109 10.1039/c7sc04032f PMC5909123

[52] F. Wang , S. S. Stahl , Acc. Chem. Res. 2020, 53, 561–574. 32049487 10.1021/acs.accounts.9b00544 PMC7295176

[53] M. Linden , S. Hofmann , A. Herman , N. Ehler , R. M. Bär , S. R. Waldvogel , Angew. Chem. Int. Ed. 2023, 62, e202214820. 10.1002/anie.202214820 36478106

[54] S. D. L. Holman , A. G. Wills , N. J. Fazakerley , D. L. Poole , D. M. Coe , L. A. Berlouis , M. Reid , Chem. Eur. J. 2022, 28, e202103728. 35076117 10.1002/chem.202103728 PMC9303936

[55] R. Francke , R. D. Little , Chem. Soc. Rev. 2014, 43, 2492. 24500279 10.1039/c3cs60464k

[56] D. M. Heard , A. J. J. Lennox , Angew. Chem. 2020, 132, 19026–19044.

[57] J. L. Röckl , M. Dörr , S. R. Waldvogel , ChemElectroChem 2020, 7, 3686–3694.

[58] J. M. Ramos-Villaseñor , E. Rodríguez-Cárdenas , C. E. Barrera Díaz , B. A. Frontana-Uribe , J. Electrochem. Soc. 2020, 167, 155509.

[59] M. D. Palkowitz , G. Laudadio , S. Kolb , J. Choi , M. S. Oderinde , T. E.-H. Ewing , P. N. Bolduc , T. Chen , H. Zhang , P. T. W. Cheng , B. Zhang , M. D. Mandler , V. D. Blasczak , J. M. Richter , M. R. Collins , R. L. Schioldager , M. Bravo , T. G. M. Dhar , B. Vokits , Y. Zhu , P.-G. Echeverria , M. A. Poss , S. A. Shaw , S. Clementson , N. N. Petersen , P. K. Mykhailiuk , P. S. Baran , J. Am. Chem. Soc. 2022, 144, 17709–17720. 36106767 10.1021/jacs.2c08006 PMC9805175

[60] J. Xiang , M. Shang , Y. Kawamata , H. Lundberg , S. H. Reisberg , M. Chen , P. Mykhailiuk , G. Beutner , M. R. Collins , A. Davies , M. Del Bel , G. M. Gallego , J. E. Spangler , J. Starr , S. Yang , D. G. Blackmond , P. S. Baran , Nature 2019, 573, 398–402. 31501569 10.1038/s41586-019-1539-y PMC6996793

[61] F. Medici , S. Resta , A. Puglisi , S. Rossi , L. Raimondi , M. Benaglia , Molecules 2021, 26, 6968. 34834060 10.3390/molecules26226968 PMC8618477

[62] S. R. Waldvogel , S. Lips , M. Selt , B. Riehl , C. J. Kampf , Chem. Rev. 2018, 118, 6706–6765. 29963856 10.1021/acs.chemrev.8b00233

[63] J. C. Ruble , B. D. Wakefield , G. M. Kamilar , K. R. Marotti , E. Melchior , M. T. Sweeney , G. E. Zurenko , D. L. Romero , Bioorg. Med. Chem. Lett. 2007, 17, 4040–4043. 17561394 10.1016/j.bmcl.2007.04.074

[64] A. K. Ecker , D. A. Levorse , D. A. Victor , M. J. Mitcheltree , ACS Med. Chem. Lett. 2022, 13, 964–971. 35707148 10.1021/acsmedchemlett.2c00114 PMC9190035

[65] J. Y. F. Wong , J. M. Tobin , F. Vilela , G. Barker , Chem. Eur. J. 2019, 25, 12439–12445. 31361052 10.1002/chem.201902917

[66] J. Simonet , Phosphorus Sulfur Silicon Relat. Elem. 1993, 74, 93–112.

[67] L. J. Klein , D. G. Peters , O. Fourets , J. Simonet , J. Electroanal. Chem. 2000, 487, 66–71.

[68] J. Delaunay , G. Mabon , M. C. El Badre , A. Orliac , J. Simonet , Tetrahedron Lett. 1992, 33, 2149–2150.

[69] J.-F. Pilard , O. Fourets , J. Simonet , L. J. Klein , D. G. Peters , J. Electrochem. Soc. 2001, 148, E171.

[70] H. Ye , H. Zhao , S. Ren , H. Ye , D. Cheng , X. Li , X. Xu , Tetrahedron Lett. 2019, 60, 1302–1305.

[71] Y. Chen , N. Ni , D. Cheng , X. Xu , Tetrahedron Lett. 2020, 61, 152425.

[72] J. Pisk , I. Đilović , T. Hrenar , D. Cvijanović , G. Pavlović , V. Vrdoljak , RSC Adv. 2020, 10, 38566–38577. 35517547 10.1039/d0ra06845d PMC9057299

[73] D. Lehnherr , L. Chen , Org. Process Res. Dev. 2024, 28, 338–366.

