# Supplementary material for: An Electrosynthesis of 1,3,4‐Oxadiazoles from N‐Acyl Hydrazones
Source: Chemistry. 2024 Oct 30;30(69):e202403128. doi: 10.1002/chem.202403128 (PMC11632415; doi:10.1002/chem.202403128)
Supplement: Supplementary file 1 — Supporting Information [file CHEM-30-e202403128-s001.pdf]

Chemistry–A European Journal

Supporting Information

An Electrosynthesis of 1,3,4-Oxadiazoles from *N*-Acyl Hydrazones

Luke Chen, James D. F. Thompson,* and Craig Jamieson*

Supporting Information

An Electrosynthesis of 1,3,4-Oxadiazoles from *N*-Acyl Hydrazones

Luke Chen,[a,b] James D. F. Thompson,*[a] and Craig Jamieson*[b]

[a] Luke Chen, James D. F. Thompson, Medicinal Chemistry, GSK, Gunnels Wood Road, Stevenage, SG1 2NY, United Kingdom. E-mail: james.d.thompson@gsk.com

[b] Luke Chen, Craig Jamieson, Pure & Applied Chemistry, University of Strathclyde, Glasgow, G1 1XL, United Kingdom. E-Mail: craig.jamieson@strath.ac.uk

Table of Contents

1. General information 2

1.1 General analytical procedures 2

2. General Procedures 3

P1: Hydrazone Synthesis 1 3

P2: Hydrazone Synthesis 2 3

P3: General Screening Procedure 3

P4: Oxadiazole Synthesis (from Isolated Hydrazones) 3

P5: One-Pot Oxadiazole Synthesis 3

3. Reaction Optimisation 4

4. Synthesis and Characterisation of Hydrazones 6

5. Synthesis and Characterisation of Oxadiazoles 41

5.1 Unsuccessful substrates 75

6. Mechanistic studies 75

7. Cyclic voltammetry 76

8. NMR Spectra of Oxadiazole Products 76

9. References 121

1. General information

Unless otherwise stated, all chemicals were used as received, all solutions used during work-ups were saturated and aqueous, and all reactions were carried out at ambient temperature. All reactions were followed by liquid chromatography-mass spectrometry (LCMS) or thin layer chromatography (TLC).

1.1 General analytical procedures

TLC was carried out on Merck Kieselgel 60 F254 plates which were visualised using ultraviolet light (254 nm).

Flash column chromatography was conducted with Teledyne ISCO CombiFlash® Rf+ apparatus with RediSep® Rf silica or Biotage KP-C18- HS cartridges, and the solvents are reported as vol:vol mixtures.

Reverse phase prep HPLC was conducted using the ACCQPrep H125 instrument and XSelect CSH C18 Column.

Fourier transform infrared (IR) spectroscopy was performed using a Perkin-Elmer Spectrum-Two spectrometer and the maximum absorbance wavelengths (v_{max}) are reported as reciprocal centimetres (cm^{-1}) and the type of signal (s = strong, vs = very strong, w = weak, m = medium, br = broad).

Melting points (mp) were measured using a BUCHI Melting Point M-565 machine.

Nuclear magnetic resonance (NMR) spectroscopy was carried out on a Bruker-AV400 MHz machine at ambient temperature (unless otherwise stated), using an internal deuterium lock. ^{1}H NMR data are presented as: chemical shift (δ) in ppm relative to the residual solvent peak (δ_H = 7.26 ppm in $CDCl_3$, 2.50 ppm in DMSO-d_6, 2.05 ppm in acetone-d_6), multiplicity (s = singlet, d = doublet, t = triplet, q = quartet, quin = quintet, m = multiplet, br = broad, app = apparent), coupling constants (*J* in Hz), proton integration and assignment to the structure. The following abbreviations (or a combination thereof) were used to account for signal multiplicities. Signals are assigned according to the numbering scheme presented on the structure itself, which is arbitrarily chosen and is not related to the numbering used in the IUPAC title name given to each compound. Assignments have been made using the 1D data presented, using COSY, HMBC and HSQC experiments, or by analogy with interpreted spectral data for similar compounds. ^{13}C NMR spectra are presented as a chemical shift (δ) in ppm relative to the residual solvent peak (δ_C = 77.16 ppm in $CDCl_3$, 39.52 ppm in DMSO-d_6).

LCMS analysis was conducted using an Acquity UPLC BEH or CSH C18 column (2.1 mm × 50 mm i.d. 1.7 µm packing diameter) using two different systems: Formic (System A) – Conducted by eluting with 0.1 % formic acid in H_2O (solvent A), and 0.1 % formic acid in MeCN (solvent B), with the following elution gradient for BEH column: 0.0–1.5 min 1–97 % B, 1.5–1.9 min 97 % B, 1.9–2.0 min 97–1 % B, and for CSH column: 0.0–1.5 min 3–100 % B, 1.5–1.9 min 100 % B, 1.9–2.0 min 100–3 % B, at a flow rate of 1 mL min^{-1} at 40 °C. HpH (System B) – Conducted by eluting with 10 mM ammonium bicarbonate in H_2O adjusted to pH 10 using aqueous ammonia (solvent A), and MeCN (solvent B), with the following elution gradient for BEH column: 0.0–1.5 min 1–97 % B, 1.5–1.9 min 97 % B, 1.9–2.0 min 97–1 % B, and for CSH column: 0.0–1.5 min 3–95 % B, 1.5–1.9 min 95 % B, 1.9–2.0 min 95–3 % B, at a flow rate of 1 mL min^{-1} at 40 °C. For both methods, the UV detection was based on a signal averaged from wavelengths of 210 nm to 350 nm, and mass spectra were recorded on a mass spectrometer with alternate-scan electrospray positive and negative mode ionisation (ES+ve and ES−ve).

2. General Procedures

P1: Hydrazone Synthesis 1

In a 20 mL vial, hydrazide (1.50 mmol, 1 equiv.), 4-fluorobenzaldehyde (162 µL, 187 mg, 1.51 mmol, 1 equiv.), EtOH (5 mL), and acetic acid (20 µL, 21.0 mg, 0.35 mmol, 0.2 equiv.) were added. The reaction mixture was stirred for 16 h at room temperature (20 – 21 °C). The reaction mixture was filtered, washed with solvent, and dried under vacuum to afford the product hydrazone.

P2: Hydrazone Synthesis 2

In a 20 mL vial, aldehyde (1.00 mmol, 1 equiv.), benzohydrazide (136 mg, 1.00 mmol, 1 equiv.), and MeOH (5 mL) were added. The reaction mixture was stirred for 3 h at 50 °C. The product was isolated or purified *via* filtration or column chromatography.

P3: General Screening Procedure

To a 5 mL ElectraSyn 2.0 reaction vessel containing a stirrer bar, (*E*)-*N*'-(4-fluorobenzylidene) acetohydrazide (45.1 mg, 0.25 mmol), electrolyte (1 equiv.), mediator, and additive were added, followed by acetonitrile (3.6 mL). The reaction mixture was electrolysed under a constant current of 5 mA at room temperature (20 – 21 °C) with electrodes, stirring at 400 rpm until a total charge of 3 F mol^{-1} had been passed. 4-Fluoroanisole (85 µL, 0.75 mmol, 3 equiv.) was added to the reaction mixture which was mixed thoroughly. An aliquot (0.2 mL) of the reaction mixture was transferred to an NMR tube, diluted with DMSO-d_6 (0.25 mL) and analysed by ^{19}F NMR spectroscopy (ratio of internal standard to product = 3:1, -125 ppm:-109 ppm).

P4: Oxadiazole Synthesis (from Isolated Hydrazones)

To a 5 mL ElectraSyn 2.0 reaction vessel containing a stirrer bar, tetraethylammonium tetrafluoroborate (54.4 mg, 0.25 mmol, 1 equiv.), DABCO (28.1 mg, 0.25 mmol, 1 equiv.), and hydrazone (0.25 mmol, 1 equiv.) were added, followed by acetonitrile (3.6 mL). The reaction mixture was electrolysed under a constant current of 5 mA at room temperature (20 – 21 °C) with a graphite anode and a platinum foil cathode, stirring at 400 rpm until a total charge of 3 F mol^{-1} had been passed. The electrodes were rinsed with MeOH (~2 mL) into the reaction mixture and the reaction mixture was concentrated *in vacuo* and purified by column chromatography.

P5: One-Pot Oxadiazole Synthesis

To a 5 mL ElectraSyn 2.0 reaction vessel containing a stirrer bar, aldehyde (0.25 mmol, 1 equiv.), hydrazide (0.25 mmol, 1 equiv.), and MeOH (1.2 mL) were added. The reaction mixture was stirred until hydrazone formation was complete as monitored by LCMS. Tetraethylammonium tetrafluoroborate (54.4 mg, 0.25 mmol, 1 equiv.), DABCO (28.1 mg, 0.25 mmol, 1 equiv.), and acetonitrile (2.4 mL) were added. The reaction mixture was electrolysed under a constant current of 5 mA at room temperature (20 – 21 °C) with a graphite anode and a platinum foil cathode, stirring at 400 rpm until a total charge of 3 F mol^{-1} had been passed. The electrodes were rinsed with MeOH (~2 mL) into the reaction mixture and the reaction mixture was concentrated *in vacuo* and purified by column chromatography.

3. Reaction Optimisation

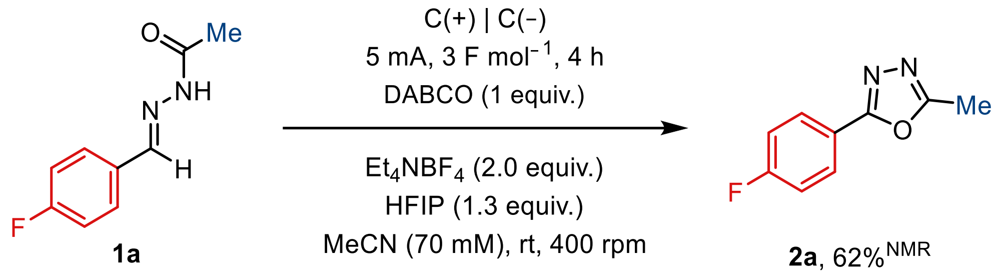

Table S1. Screening of mediators. Yields determined by ^{19}F NMR spectroscopy. [a] Instead of Et_4NBF_4; [b] In addition to Et_4NBF_4.

Entry	Deviation from above conditions	NMR yield (%)
1	aceclidine (1 equiv.)	34
2	quinuclidine (1 equiv.)	23
3	$^n Bu_4NBr$ (1 equiv.)[a]	20
4	Et_4NCl (2 equiv.)[a]	18
5	KCl (0.2 equiv.)[b]	12
6	NHPI (0.2 equiv.) + pyridine (0.2 equiv.)	10
7	TCNHPI (0.2 equiv.) + pyridine (0.2 equiv.)	8
8	$^n Bu_4NI$ (1 equiv.)[a]	13
9	TEMPO (0.5 equiv.)	0
10	ABNO (0.5 equiv.)	0
11	ACT (0.5 equiv.)	1
12	BzO-TEMPO (0.5 equiv.)	8
13	AZADO (0.5 equiv.)	3
14	Ph_3N (0.5 equiv.)	4
15	$(4\text{-}BrPh)_3N$ (0.5 equiv.)	8
16	Ferrocene (0.5 equiv.)	0

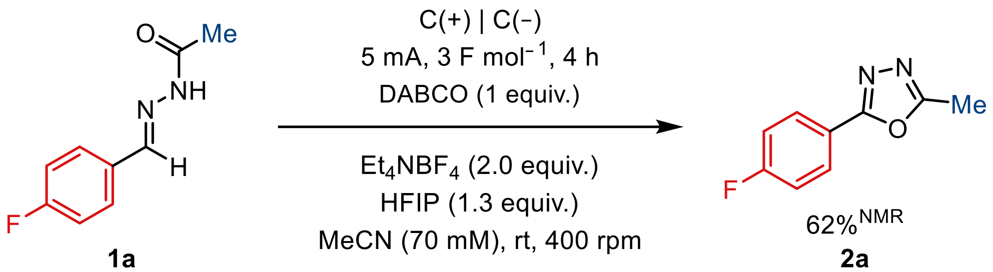

Table S2. Screening of reaction parameters. Yields determined by ^{19}F NMR spectroscopy. [a] C|Pt, no HFIP; [b] isolated yield.

Entry	Deviation from above conditions	Yield (%)	Entry	Deviation from above conditions	Yield (%)
1	C(+)|Pt(−)	72	17	2 mA	58
2	C(+)|SS(−)	55	18	10 mA	41
3	GC(+/−)	45	19	15 mA	20
4	RVC(+/−)	32	20	25 mA	16
5	$^{n}Bu_4NCl_4$	60	21	No HFIP	63[b]
6	$^{n}Bu_4NOTs$	59	22	Pyridine (1 equiv.)	56
7	$^{n}Bu_4NPF_6$[a]	60[b]	23	NaOAc (1 equiv.)	51
8	$LiClO_4$	13	24	NEt_3 (1 equiv.)	8
9	MeOH	60	25	AcOH (1 equiv.)	40
10	Acetone	55	26	$AgNO_3$ (1 equiv.)	4
11	DMSO	0	27	DCM (1 equiv.)	61
12	DMF	8	28	0 °C	44
13	HFIP	3	29	40 °C	46
14	EtOH	4	30	100 rpm	44
15	CH_2Cl_2	8	31	700 rpm	47
16	EtOAc	1	32	DABCO (0.2 equiv.)	19

4. Synthesis and Characterisation of Hydrazones

N'-(4-Fluorobenzylidene)acetohydrazide (1a)

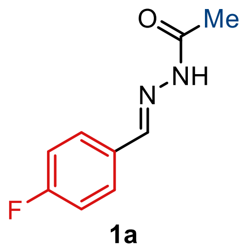

1a

In a 100 mL round-bottom flask equipped with a magnetic stirrer bar, acetohydrazide (1.21 g, 16.3 mmol, 1 equiv.) and 4-fluorobenzaldehyde (1.75 mL, 2.03 g, 16.3 mmol, 1 equiv.) were added, followed by acetonitrile (10 mL). A white-coloured solid started to form upon addition of the aldehyde. The heterogeneous mixture was sonicated for 2 min at room temperature. The reaction mixture was concentrated *in vacuo* and the white-coloured solid was transferred into a 20 mL vial. The solid was further dried in a vacuum oven at 40 °C to afford a mixture of *E*/*Z*-isomers of *N*'-(4-fluorobenzylidene)acetohydrazide (2.78 g, 15.4 mmol, 94%) as a white-coloured solid.

mp: 159 – 166 °C.

^{1}H NMR (400 MHz, DMSO-d_6) δ

Isomer 1: 11.23 (s, 1H), 7.97 (s, 1H), 7.78 – 7.65 (m, 2H), 7.31 – 7.21 (m, 2H), 2.19 (s, 3H)

Isomer 2: 11.35 (s, 0.5H), 8.14 (s, 0.5H), 7.78 – 7.65 (m, 1H), 7.31 – 7.21 (m, 1H), 1.94 (s, 1.5H)

Ratio (isomer 1:isomer 2): 1:0.5 (7.97 ppm:8.14 ppm). Purity = 95%

^{13}C NMR (101 MHz, DMSO-d_6) δ

Isomer 1: 172.4, 163.3 (d, J_{CF} = 245.7 Hz), 141.8, 131.4 (d, J_{CF} = 3.1 Hz), 129.2 (d, J_{CF} = 9.2 Hz), 116.3 (d, J_{CF} = 21.4 Hz), 20.7.

Isomer 2: 166.0, 163.4 (d, J_{CF} = 247.2 Hz), 144.9, 131.5 (d, J_{CF} = 3.1 Hz), 129.5 (d, J_{CF} = 9.2 Hz), 116.3 (d, J_{CF} = 21.4 Hz), 22.1.

^{19}F NMR (376 MHz, DMSO-d_6) δ

Isomer 2: -110.98 (s, 0.5F).

Isomer 1: -111.28 (s, 1F).

LCMS: t_r = 0.74 min (HpH), ES+ (m/z) 180.99 ([M+H]$^+$, 100%), Purity = 100%

FTIR: ν_{max} / cm^{-1} 3312w, 3056m, 2964m 2881w, 1671s, 1611s.

HRMS: m/z Calcd. for $C_9H_{10}FN_2O$ [M+H]$^+$ 181.0771, found 181.0791.

Calcd. for $C_9H_9FN_2ONa$ [M+Na]$^+$ 203.0591, found 203.0608.

Spectroscopic data consistent with literature.[1]

N'-(4-Fluorobenzylidene)pentanehydrazide (1b)

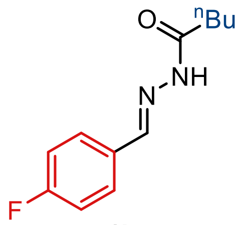

1b

In a 20 mL vial, pentanehydrazide (174 mg, 1.50 mmol, 1 equiv.) and 4-fluorobenzaldehyde (161 µL, 186 mg, 1.50 mmol, 1 equiv.) were added, followed by acetonitrile (5 mL). The heterogeneous mixture was sonicated for 15 min at room temperature then stirred for 3 h. The reaction mixture was transferred to a round-bottom flask and concentrated *in vacuo* to give a yellow-coloured solid. The crude material was liquid loaded with a minimum amount of CH_2Cl_2 onto a 12 g RediSep® silica column, and purified by column chromatography (0 – 70% EtOAc:cyclohexane). The appropriate product-containing fractions were combined and concentrated *in vacuo* to give a white-coloured solid. The solid was further dried in a vacuum oven at 40 °C to afford a mixture of *E*/*Z*-isomers of *N*'-(4-fluorobenzylidene)pentanehydrazide (133 mg, 0.60 mmol, 40%) as a white-coloured solid.

^{1}H NMR (400 MHz, $CDCl_3$) δ

Isomer 1: 9.20 (s, 1H), 7.72 (s, 1H), 7.69 – 7.61 (m, 2H), 7.15 – 7.04 (m, 2H), 2.75 (t, *J* = 7.4 Hz, 2H), 1.72 (dt, *J* = 15.2, 7.5 Hz, 2H), 1.44 (dq, *J* = 14.9, 7.4 Hz, 2H), 0.97 (t, *J* = 7.4 Hz, 3H).

Isomer 2: 8.49 (br s, 0.09H), 8.18 (s, 0.09H), 2.30 (t, *J* = 7.5 Hz, 0.18H). The remaining peaks could not be discerned due to a low concentration.

Ratio (isomer 1:isomer 2): 1:0.09 (7.72 ppm:8.18 ppm). Purity = 95%

^{13}C NMR (101 MHz, $CDCl_3$) δ 176.5, 163.8 (d, J_{CF} = 250.2 Hz), 142.0, 130.2 (d, J_{CF} = 3.1 Hz), 128.9 (d, J_{CF} = 7.6 Hz), 115.9 (d, J_{CF} = 21.4 Hz), 32.5, 26.9, 22.5, 13.9. The peaks corresponding to isomer 2 were not observed due to low concentration.

^{19}F NMR (376 MHz, $CDCl_3$) δ

Isomer 2: -109.44 (s, 0.09F)

Isomer 1: -109.98 (s, 1F)

LCMS: t_r = 1.01 min (HpH), ES+ (m/z) 223.05 ([M+H]$^+$, 100%), ES- (m/z) 221.31 ([M-H]$^-$, 100%), Purity = 100%

FTIR: ν_{max} / cm^{-1} 3221br, m, 2957w, 2931w, 2869w, 1672s.

HRMS: m/z Calcd. for $C_{12}H_{15}FN_2ONa$ [M+Na]$^+$ 245.1061, found 245.1077.

N'-(4-Fluorobenzylidene)-3-methoxypropanehydrazide (1c)

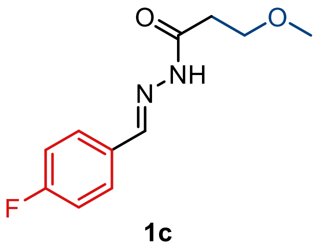

1c

In a 20 mL vial, 3-methoxypropanehydrazide (191 mg, 1.62 mmol, 1 equiv.) and 4-fluorobenzaldehyde (174 µL, 201 mg, 1.62 mmol, 1 equiv.) were added, followed by acetonitrile (5 mL). The heterogeneous mixture was sonicated for 10 min at room temperature. The reaction mixture was transferred to a round-bottom flask and concentrated *in vacuo* to give a yellow-coloured solid. The crude material was liquid loaded with a minimum amount of CH_2Cl_2 onto a 24 g RediSep® silica column, and purified by column chromatography (0 – 100% EtOAc:cyclohexane). The appropriate product-containing fractions were combined and concentrated *in vacuo* to give a white-coloured solid. The solid was further dried in a vacuum oven at 40 °C to afford a mixture of *E*/*Z*-isomers of *N*'-(4-fluorobenzylidene)-3-methoxypropanehydrazide (342 mg, 1.53 mmol, 94%) as a white-coloured solid.

^{1}H NMR (400 MHz, $CDCl_3$) δ

Isomer 1: 9.40 (br s, 1H), 7.75 (s, 1H), 7.68 – 7.61 (m, 2H), 7.13 – 7.05 (m, 2H), 3.80 (t, *J* = 6.6 Hz, 2H), 3.40 (s, 3H), 3.06 (t, *J* = 6.6 Hz, 2H).

Isomer 2: 9.42 (br s, 0.37H), 8.11 (s, 0.37H), 7.74 – 7.70 (m, 0.74H), 7.13 – 7.05 (m, 0.74H) 7.13 – 7.05 (m, 2H), 3.72 (t, *J* = 5.6 Hz, 0.74H), 3.44 (s, 1.11H), 2.61 (t, *J* = 5.6 Hz, 0.74H)

Ratio (isomer 1:isomer 2): 2:0.74 (3.06 ppm:2.61 ppm) = 1:0.37. Purity = 95%

^{13}C NMR (101 MHz, $CDCl_3$) δ

Isomer 1: 173.5, 163.9 (d, J_{CF} = 251.6 Hz), 142.3, 129.9 (d, J_{CF} = 2.9 Hz), 129.0 (d, J_{CF} = 8.8 Hz), 115.9 (d, J_{CF} = 22.0 Hz), 67.9, 58.8, 33.2.

Isomer 2: 167.9, 146.6, 129.5 (d, J_{CF} = 8.8 Hz) 115.8 (br d, J_{CF} = 22.0 Hz), 68.4, 58.9, 36.3. 2C not observed.

^{19}F NMR (376 MHz, $CDCl_3$) δ

Isomer 1: -109.70 (s, 1F)

Isomer 2: -109.48 (s, 0.37F)

LCMS: t_r = 0.77 min (HpH), ES+ (m/z) 224.99 ($[M+H]^+$, 100%), ES- (m/z) 223.11 ($[M-H]^-$, 100%), Purity = 100%

FTIR: ν_{max} / cm^{-1} 3206br,m, 3077w, 3056w, 2983w, 2914w, 1667vs, 1554s.

HRMS: m/z Calcd. for $C_{11}H_{14}FN_2O_2^+$ $[M+H]^+$ 225.1034, found 225.1045.

m/z Calcd. for $C_{11}H_{13}FN_2O_2Na^+$ $[M+H]^+$ 247.0853, found 247.0860.

***N*'-(4-Fluorobenzylidene)formohydrazide (1d)**

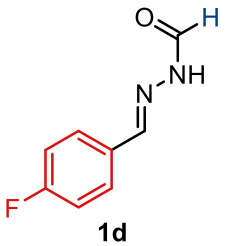

1d

In a 20 mL vial, formohydrazide (90.1 mg, 1.50 mmol, 1 equiv.) and 4-fluorobenzaldehyde (161 µL, 186 mg, 1.50 mmol, 1 equiv.) were added, followed by acetonitrile (5 mL). The heterogeneous mixture was sonicated for 15 min at room temperature. The reaction mixture was stirred for 3 h at room temperature then transferred to a round-bottom flask. Florisil® was added to the flask and the mixture was concentrated *in vacuo* to give a white-coloured solid. The crude material was solid loaded with Florisil® onto a 12 g RediSep® silica column, and purified by column chromatography (0 – 100% EtOAc:cyclohexane). The appropriate product-containing fractions were combined and concentrated *in vacuo* to give a white-coloured solid. The solid was further dried in a vacuum oven at 40 °C to afford a mixture of *E*/*Z*-isomers of *N*'-(4-fluorobenzylidene)formohydrazide (171 mg, 1.03 mmol, 69%) as a white-coloured solid.

mp: 159 – 161 °C.

^{1}H NMR (400 MHz, $CDCl_3$) δ

Isomer 1: 9.48 (br d, *J* = 9.0 Hz, 1H), 8.79 (d, *J* = 10.3 Hz, 1H), 7.83 (s, 1H), 7.71 – 7.61 (m, 2H), 7.17 – 7.04 (m, 2H).

Isomer 2: 8.43 (br s, 0.07H), 8.31 (s, 0.07H), 8.31 (s, 0.07H), 7.76 – 7.71 (m, 0.14H). The remaining peaks could not be discerned due to overlapping peaks and low concentration.

Ratio (isomer 1:isomer 2): 1:0.07 (7.83 ppm:8.31 ppm). Purity = 95%

^{13}C NMR (101 MHz, $CDCl_3$) δ 165.1, 162.8, 144.6, 129.5 (d, J_{CF} = 2.9 Hz), 129.1 (d, J_{CF} = 8.8 Hz), 116.0 (d, J_{CF} = 22.0 Hz). 1C not observed. The peaks corresponding to isomer 2 were not observed due to low concentration.

^{19}F NMR (376 MHz, $CDCl_3$) δ

Isomer 2: -108.61 (s, 0.07F)

Isomer 1: -109.25 (s, 1F)

LCMS: t_r = 0.74 min (HpH), ES+ (m/z) 166.98 ($[M+H]^+$, 100%), ES- (m/z) 165.21 ($[M-H]^-$, 100%), Purity = 100%.

FTIR: ν_{max} / cm^{-1} 3182w, 3060m, 2955m, 2897m, 1696vs, 1616m.

HRMS: m/z Calcd. for $C_8H_8FN_2O^+$ $[M+H]^+$ 167.0615, found 167.0625.

N'-(4-Fluorobenzylidene)-2-(2-oxoazepan-1-yl)acetohydrazide (1e)

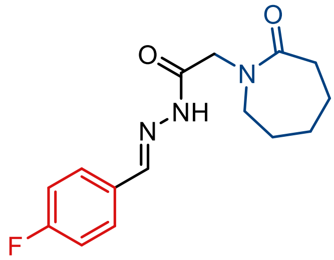

1e

The synthesis of **1e** was conducted following the general procedure P1 using 2-(2-oxoazepan-1-yl)acetohydrazide (278 mg, 1.50 mmol, 1 equiv.) and 4-fluorobenzaldehyde (162 µL, 187 mg, 1.50 mmol, 1 equiv.). The reaction mixture was filtered, washed with EtOH, and dried under vacuum to afford a mixture of *E*/*Z*-isomers of *N*'-(4-fluorobenzylidene)-2-(2-oxoazepan-1-yl)acetohydrazide (305 mg, 1.05 mmol, 70%) as a white-coloured solid.

^{1}H NMR (400 MHz, DMSO-d$_6$) δ

Isomer 1: 11.40 (s, 1H), 7.96 (s, 1H), 7.79 – 7.66 (m, 2H), 7.33 – 7.18 (m, 2H), 4.47 (s, 2H), 3.46 – 3.38 (m, 2H), 2.49 – 2.44 (m, 2H), 1.72 – 1.62 (m, 4H), 1.61 – 1.52 (m, 2H).

Isomer 2: 11.39 (br s, 0.39H), 8.20 (s, 0.39H), 7.79 – 7.66 (m, 0.78H), 7.33 – 7.18 (m, 0.78H), 4.04 (s, 0.78H), 3.46 – 3.38 (m, 0.78H), 2.49 – 2.44 (m, 0.78H), 1.72 – 1.62 (m, 1.56H), 1.61 – 1.52 (m, 0.78H).

Ratio (isomer 1:isomer 2): 1:0.39 (7.96 ppm:8.20 ppm). Purity = 95%

^{13}C NMR (101 MHz, DMSO-d$_6$) δ 175.6, 175.5, 170.6, 165.8, 163.5 (d, J_{CF} = 247.2 Hz), 163.4 (d, J_{CF} = 248.7 Hz), 145.8, 142.4, 131.4 (d, J_{CF} = 3.1 Hz), 131.2 (d, J_{CF} = 3.1 Hz), 129.6 (d, J_{CF} = 9.2 Hz), 129.4 (d, J_{CF} = 9.2 Hz), 116.3 (d, J_{CF} = 22.9 Hz), 50.8, 50.7, 50.2, 36.9, 36.8, 29.8, 29.8, 28.1, 28.0, 23.5, 23.4. ^{13}C NMR data corresponds to a mixture of isomers. 2C not observed.

^{19}F NMR (376 MHz, DMSO-d$_6$) δ

Isomer 2: -110.78 (s, 0.39F)

Isomer 1: -111.04 (s, 1F)

LCMS: t_r = 0.86 min (HpH), ES+ (m/z) 291.99 ([M+H]$^+$, 100%), ES- (m/z) 290.12 ([M-H]$^-$, 100%), Purity = 100%.

FTIR: ν_{max} / cm^{-1} 3065w, 2931m, 1694s, 1683s, 1627s.

HRMS: m/z Calcd. for $C_{15}H_{19}FN_3O_2^+$ [M+H]$^+$ 292.1456, found 292.1467.

m/z Calcd. for $C_{15}H_{18}FN_3O_2Na^+$ [M+Na]$^+$ 314.1287, found 314.1287.

3-(1*H*-Benzo[*d*]imidazol-1-yl)-*N*'-(4-fluorobenzylidene)propanehydrazide (1f)

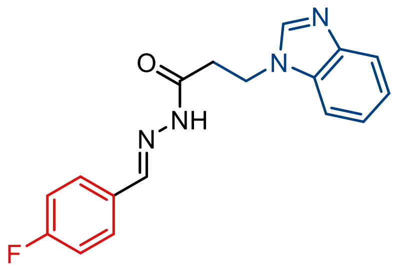

1f

The synthesis of **1f** was conducted following the general procedure P1 using 3-(1*H*-benzo[*d*]imidazol-1-yl)propanehydrazide (306 mg, 1.50 mmol, 1 equiv.) and 4-fluorobenzaldehyde (162 µL, 187 mg, 1.50 mmol, 1 equiv.). The reaction mixture was filtered, washed with EtOH and cyclohexane, and dried under vacuum to afford a mixture of *E*/*Z*-isomers of 3-(1*H*-benzo[*d*]imidazol-1-yl)-*N*'-(4-fluorobenzylidene)propanehydrazide (430 mg, 1.30 mmol, 86%, 93% purity) as a beige-coloured solid.

^{1}H NMR (400 MHz, DMSO-d$_6$) δ

Isomer 1: 11.41 (br s, 1H), 8.22 (s, 1H), 7.93 (s, 1H), 7.75 – 7.58 (m, 4H), 7.32 – 7.16 (m, 4H), 4.57 (br t, *J* = 6.7 Hz, 2H), 3.22 (br t, *J* = 6.7 Hz, 2H).

Isomer 2: 11.41 (br s, 0.5H), 8.17 (s, 0.5H), 8.09 (s, 0.5H), 7.75 – 7.58 (m, 2H), 7.32 – 7.16 (m, 2H), 4.57 (br t, *J* = 6.6 Hz, 1H), 2.81 (br t, *J* = 6.6 Hz, 1H).

Ratio (isomer 1:isomer 2): 1:0.5 (8.22 ppm:8.17 ppm). Purity = 93%

^{13}C NMR (101 MHz, DMSO-d$_6$) δ

Isomer 1: 172.4, 164.6, 144.7, 143.9, 142.6, 131.1 (d, J_{CF} = 3.1 Hz), 129.4 (d, J_{CF} = 7.6 Hz), 122.7, 121.9, 119.9, 116.2 (d, J_{CF} = 21.4 Hz), 110.8, 40.4, 33.0. 1C not observed.

Isomer 2: 166.6, 162.1, 145.8, 144.6, 134.1, 131.2, 129.7 (d, J_{CF} = 9.2 Hz), 122.8, 121.9, 119.9, 116.3 (d, J_{CF} = 21.4 Hz), 110.9, 40.7, 34.9. 1C not observed.

^{19}F NMR (376 MHz, DMSO-d$_6$) δ

Isomer 1: -111.03 (s, 1F)

Isomer 2: -110.68 (s, 1F)

LCMS: t_r = 0.90 min (HpH), ES+ (m/z) 310.95 ([M+H]$^+$, 100%), Purity = 98%

FTIR: ν_{max} / cm^{-1} 3196w, 3078w, 2946w, 2821w, 1671vs.

HRMS: m/z Calcd. for $C_{17}H_{16}FN_4O^+$ [M+H]$^+$ 311.1303, found 311.1313.

N'-(4-Fluorobenzylidene)cyclopropanecarbohydrazide (1g)

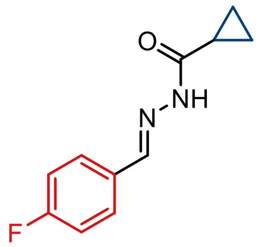

1g

In a 20 mL vial, cyclopropanecarbohydrazide (150 mg, 1.50 mmol, 1 equiv.) and 4-fluorobenzaldehyde (161 µL, 186 mg, 1.50 mmol, 1 equiv.) were added, followed by acetonitrile (5 mL). The reaction mixture was sonicated for 15 min at room temperature. The solvent was evaporated in a blowdown at 40 °C to give a white-coloured solid which was further dried in a vacuum oven at 40 °C to afford *E*/*Z*-isomers of *N*'-(4-fluorobenzylidene)cyclopropanecarbohydrazide (305 mg, 1.48 mmol, 87%, 88% purity) as a white-coloured solid.

^{1}H NMR (400 MHz, $CDCl_3$) δ 9.04 (br s, 1H), 7.74 (s, 1H), 7.67 (br dd, *J* = 8.6, 5.4 Hz, 2H), 7.09 (t, *J* = 8.6 Hz, 2H), 2.75 – 2.64 (m, 1H), 1.18 – 1.11 (m, 2H), 1.00 – 0.91 (m, 2H). Purity = 88%.

^{13}C NMR (101 MHz, $CDCl_3$) δ 176.9, 163.9 (d, J_{CF} = 250.2 Hz), 142.6, 130.5 (d, J_{CF} = 2.9 Hz), 129.0 (d, J_{CF} = 8.1 Hz), 116.0 (d, J_{CF} = 22.0 Hz), 10.5, 9.0.

^{19}F NMR (376 MHz, $CDCl_3$) δ

Isomer 1: -109.60 (s, 0.12F)

Isomer 2: -110.03 (s, 1F)

LCMS: t_r = 0.86 min (HpH), ES+ (m/z) 207.05 ($[M+H]^+$, 100%), ES- (m/z) 205.27 ($[M-H]^-$, 100%), Purity = 100%

FTIR: ν_{max} / cm^{-1} 3184w, 3072w, 3011w, 2957w, 1657s.

HRMS: m/z Calcd. for $C_{22}H_{22}F_2N_4O_2Na^+$ $[2M+Na]^+$ 435.1603, found 435.1606.

(1*S*,2*S*)-*N*'-(4-Fluorobenzylidene)-2-phenylcyclopropane-1-carbohydrazide (1h)

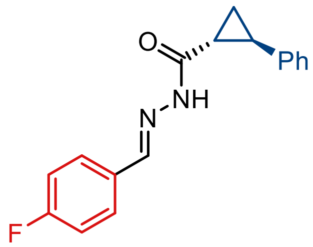

1h

The synthesis of **1h** was conducted following the general procedure P1 using (1*S*,2*S*)-2-phenylcyclopropane-1-carbohydrazide (264 mg, 1.50 mmol, 1 equiv.) and 4-fluorobenzaldehyde (162 µL, 187 mg, 1.50 mmol, 1 equiv.). The reaction mixture was filtered, washed with EtOH, and dried under vacuum to afford a mixture of *E*/*Z*-isomers of (1*S*,2*S*)-*N*'-(4-fluorobenzylidene)-2-phenylcyclopropane-1-carbohydrazide (266 mg, 0.94 mmol, 63%) as a white-coloured solid.

^{1}H NMR (400 MHz, DMSO-d$_6$) δ 11.66 (s, 0.5H), 11.47 (s, 0.5H), 8.15 (s, 0.5H), 8.03 (s, 0.5H), 7.78 – 7.64 (m, 2H), 7.33 – 7.15 (m, 7H), 2.94 – 2.87 (m, 1H), 2.45 (ddd, *J* = 9.2, 6.2, 4.3 Hz, 0.5H), 2.39 – 2.31 (m, 0.5H), 1.97 – 1.88 (m, 0.5H), 1.56 – 1.45 (m, 1H), 1.44 – 1.32 (m, 1H). ^{1}H NMR data corresponds to a mixture of isomers.

Ratio (isomer 1:isomer 2): 1:1 (8.15 ppm:8.03 ppm). Purity = 95%

^{13}C NMR (101 MHz, DMSO-d$_6$) δ 173.3, 168.1, 163.5 (d, J_{CF} = 247.2 Hz), 163.3 (d, J_{CF} = 247.2 Hz), 145.1, 142.6, 141.3, 141.1, 131.4 (d, J_{CF} = 2.9 Hz), 131.3 (d, J_{CF} = 2.9 Hz), 129.6 (d, J_{CF} = 8.8 Hz), 129.3 (d, J_{CF} = 8.8 Hz), 128.9, 128.8, 126.6, 126.5, 126.4, 116.4 (d, J_{CF} = 3.7 Hz), 116.2 (d, J_{CF} = 3.7 Hz), 25.9, 25.5, 25.2, 22.3, 17.1, 15.8. ^{13}C NMR data corresponds to a mixture of isomers. 1C not observed.

^{19}F NMR (376 MHz, DMSO-d$_6$) δ

Isomer 1: -110.89 (s, 0.5F)

Isomer 2: -111.09 (s, 0.5F)

LCMS: t_r = 1.13 min (HpH), ES+ (m/z) 282.97 ([M+H]$^+$, 100%), ES- (m/z) 281.11 ([M-H]$^-$, 100%), Purity = 100%.

FTIR: ν_{max} / cm^{-1} 3180w, 3068w, 2945w, 2845w, 1662s.

HRMS: m/z Calcd. for $C_{17}H_{16}FN_2O^+$ [M+H]$^+$ 283.1241, found 283.1255.

m/z Calcd. for $C_{17}H_{15}FN_2ONa^+$ [M+Na]$^+$ 305.1061, found 305.1072.

3-(2-(4-Fluorobenzylidene)hydrazine-1-carbonyl)azetidine-1-carboxylate (1i)

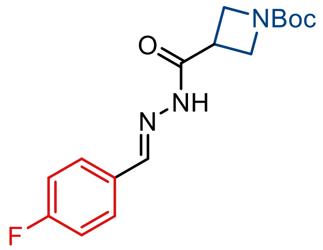

1i

In a 20 mL vial, *tert*-butyl 3-(hydrazinecarbonyl)azetidine-1-carboxylate (323 mg, 1.50 mmol, 1 equiv.) and 4-fluorobenzaldehyde (161 µL, 186 mg, 1.50 mmol, 1 equiv.) were added, followed by acetonitrile (5 mL). The heterogeneous mixture was sonicated for 5 min at room temperature. The reaction mixture was stirred for 3 h at room temperature then transferred to a round-bottom flask. Florisil® was added to the flask and the mixture was concentrated *in vacuo* to give a yellow-coloured solid. The crude material was solid loaded with Florisil® onto a 24 g RediSep® silica column, and purified by column chromatography (0 – 100% EtOAc:cyclohexane). The appropriate product-containing fractions were combined and concentrated *in vacuo* to give a white-coloured solid. The solid was further dried in a vacuum oven at 40 °C to afford a mixture of *E*/*Z*-isomers of 3-(2-(4-fluorobenzylidene)hydrazine-1-carbonyl)azetidine-1-carboxylate (385 mg, 1.20 mmol, 80%) as a white-coloured solid.

^{1}H NMR (400 MHz, $CDCl_3$) δ

Isomer 1: 8.93 (br s, 1H), 7.71 (s, 1H), 7.64 – 7.56 (m, 2H), 7.15 – 7.06 (m, 2H), 4.27 – 4.14 (m, 4H), 3.98 (quin., *J* = 7.9 Hz, 1H), 1.45 (s, 9H).

Isomer 2: 8.21 (s, 0.04H). The remaining peaks could not be discerned due to low concentration.

Ratio (isomer 1:isomer 2): 1:0.04 (7.71 ppm:8.21 ppm). Purity = 95%

^{13}C NMR (101 MHz, $CDCl_3$) δ 174.2, 164.2 (d, J_{CF} = 251.8 Hz), 156.4, 143.4, 129.8 (d, J_{CF} = 3.1 Hz), 129.2 (d, J_{CF} = 7.6 Hz), 116.2 (d, J_{CF} = 22.9 Hz), 79.8, 51.4 (br s), 31.6, 28.6. The peaks corresponding to isomer 2 were not observed due to low concentration.

^{19}F NMR (376 MHz, $CDCl_3$) δ

Isomer 2: -108.79 (s, 0.04F)

Isomer 1: -109.07 (s, 1F)

LCMS: t_r = 1.06 min (HpH), ES+ (m/z) 321.93 ($[M+H]^+$, 5%), ES- (m/z) 320.16 ($[M-H]^-$, 100%), Purity = 100%

FTIR: ν_{max} / cm^{-1} 3216w, 3087w, 2970w, 1691s, 1663vs.

HRMS: m/z Calcd. for $C_{16}H_{20}FN_3O_3Na^+$ $[M+Na]^+$ 344.1381, found 344.1384.

***tert*-Butyl (*S*)-2-(2-(4-fluorobenzylidene)hydrazine-1-carbonyl)pyrrolidine-1-carboxylate (1j)**

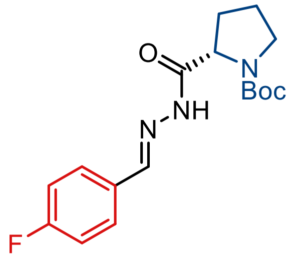

1j

The synthesis of **1j** was conducted following the general procedure P1 using *tert*-butyl (*S*)-2-(hydrazinecarbonyl)pyrrolidine-1-carboxylate (344 mg, 1.50 mmol, 1 equiv.) and 4-fluorobenzaldehyde (162 µL, 187 mg, 1.50 mmol, 1 equiv.). The reaction mixture was concentrated *in vacuo*, liquid loaded with a minimum amount of CH_2Cl_2 onto a 24 g RediSep® silica column and purified by column chromatography (0 – 80% EtOAc:cyclohexane). The appropriate column fractions were combined and concentrated *in vacuo* to afford a mixture of rotamers and *E*/*Z*-isomers of *tert*-butyl (*S*)-2-(2-(4-fluorobenzylidene)hydrazine-1-carbonyl)pyrrolidine-1-carboxylate (497 mg, 1.48 mmol, 99%) as a white-coloured solid.

^{1}H NMR (400 MHz, DMSO-d_6) δ 11.48 – 11.36 (m, 0.7H), 11.36 – 11.19 (m, 0.3H), 8.25 (s, 0.25H), 8.22 (s, 0.19H), 8.15 (s, 0.04H), 7.97 (s, 0.53H), 7.78 – 7.68 (m, 2H), 7.32 – 7.22 (m, 2H), 5.03 (td, *J* = 8.9, 3.4 Hz, 0.44H), 4.22 – 4.02 (m, 0.44H), 3.44 – 3.33 (m, 2.2H), 2.34 – 2.10 (m, 1.32H), 1.93 – 1.74 (m, 2.8H), 1.39 (d, *J* = 3.0 Hz, 3.6H), 1.35 – 1.12 (m, 5.3H). Purity = 95%. ^{1}H NMR data corresponds to a mixture of isomers and rotamers.

^{1}**H NMR** (400 MHz, 120 °C, DMSO-d$_6$) δ 11.06 – 10.75 (m, 1H), 8.18 (br s, 1H), 7.78 – 7.66 (m, 2H), 7.28 – 7.16 (m, 2H), 3.49 – 3.35 (m, 2H), 2.24 (br d, *J* = 4.9 Hz, 1H), 2.16 – 2.08 (m, 1H), 1.99 – 1.78 (m, 3H), 1.38 (s, 9H). 1H not observed.

^{13}**C NMR** (101 MHz, DMSO-d$_6$) δ 173.7, 173.1, 169.1, 168.8, 163.0 (d, J_{CF} = 247.2 Hz), 162.9 (d, J_{CF} = 247.2 Hz), 153.1, 145.5, 141.7, 130.9 (d, J_{CF} = 3.1 Hz), 130.8 (d, J_{CF} = 3.1 Hz), 129.1 (d, J_{CF} = 9.2 Hz), 128.8 (d, J_{CF} = 9.2 Hz), 115.8 (d, J_{CF} = 21.4 Hz), 78.7, 78.6, 78.4, 78.2, 58.9, 58.6, 56.6, 56.3, 46.7, 46.7, 46.5, 46.5, 30.9, 30.1, 30.0, 29.3, 28.1, 28.1, 28.0, 27.9, 24.0, 23.8, 23.4, 23.2, 21.6, 20.2. ^{13}C NMR data corresponds to a mixture of isomers and rotamers.

^{19}**F NMR** (376 MHz, DMSO-d$_6$) -110.75 (s, 0.92F), -110.79 (s, 0.62F), -110.98 (s, 0.27F), -111.03 (s, 1F), -111.06 (s, 0.66F), -111.27 (s, 0.36F). ^{19}F NMR data corresponds to a mixture of isomers and rotamers.

LCMS: t_r = 1.02 min (HpH), ES+ (m/z) 335.97 ([M+H]$^+$, 100%), ES- (m/z) 334.15 ([M-H]$^-$, 100%), Purity = 76%.

FTIR ν_{max} / cm^{-1} 3190w, 3075w, 3051w, 2977w, 2931w, 2882w, 1702vs, 1673vs.

HRMS: m/z Calcd. for $C_{17}H_{22}FN_3O_3Na^+$ [M+Na]$^+$ 358.1537, found 358.1541.

Benzyl (*S*)-(1-(2-(4-fluorobenzylidene)hydrazineyl)-3-hydroxy-1-oxopropan-2-yl)carbamate (1k)

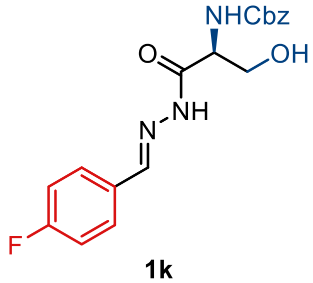

1k

The synthesis of **1k** was conducted following the general procedure P1 using (benzyl (*S*)-(1-hydrazineyl-3-hydroxy-1-oxopropan-2-yl)carbamate (380 mg, 1.50 mmol, 1 equiv.) and 4-fluorobenzaldehyde (162 µL, 187 mg, 1.50 mmol, 1 equiv.). The reaction mixture was filtered, washed with EtOH, dried under vacuum, and further dried in a vacuum oven at 30 °C. The crude material was liquid loaded with DMSO (0.4 mL) onto a 24 g RediSep® silica column and purified by column chromatography (0–100% EtOAc:cyclohexane). The appropriate column fractions were combined and concentrated *in vacuo* to afford a mixture of *E*/*Z*-isomers of benzyl (*S*)-(1-(2-(4-fluorobenzylidene)hydrazineyl)-3-hydroxy-1-oxopropan-2-yl)carbamate (315 mg, 0.88 mmol, 58%) as a white-coloured solid.

^{1}**H NMR** (400 MHz, DMSO-d$_6$) δ 11.48 (br s, 0.5H), 11.40 (br s, 0.5H), 8.24 (s, 0.5H), 7.98 (s, 0.5H), 7.84 – 7.65 (m, 2H), 7.46 – 7.15 (m, 7H), 5.04 (s, 1H), 5.04 (s, 1H), 5.03 – 4.94 (m, 1H), 4.84 (br t, *J* = 5.9 Hz, 0.5H), 4.18 – 4.09 (m, 0.5H), 3.78 – 3.70 (m, 0.5H), 3.69 – 3.55 (m, 2.5H). ^{1}H NMR data corresponds to a mixture of isomers.

Ratio (isomer 1:isomer 2): 1:1 (8.24 ppm:7.98 ppm). Purity = 95%

^{13}**C NMR** (101 MHz, DMSO-d$_6$) δ 171.4, 169.4, 167.0, 163.1 (d, J_{CF} = 247.2 Hz), 163.0 (d, J_{CF} = 247.2 Hz), 156.0, 155.9, 145.9, 142.2, 137.1, 137.0, 137.0, 130.9 (d, J_{CF} = 3.1 Hz), 130.8 (d, J_{CF} = 3.1 Hz), 129.2 (d, J_{CF} = 9.2 Hz), 129.0 (d, J_{CF} = 7.6 Hz), 128.3, 127.8, 127.7, 127.7, 115.9 (d, J_{CF} = 21.4 Hz), 65.6, 65.4, 61.6, 61.1, 56.4, 54.6. ^{13}C NMR data corresponds to a mixture of isomers. 1C not observed.

^{19}F NMR (376 MHz, DMSO-d$_6$) δ

Isomer 1: -110.71 (s, 1F)

Isomer 2: -110.93 (s, 1F)

LCMS: t_r = 0.92 min (HpH), ES+ (m/z) 359.94 ([M+H]$^+$, 100%), ES- (m/z) 358.19 ([M-H]$^-$, 100%), Purity = 92%.

FTIR: $ν_{max}$ / cm^{-1} 3293w, 3205w, 3035w, 2977w, 2956w, 1691s, 1673vs, 1660s.

HRMS: m/z Calcd. for $C_{19}H_{19}FN_3O_4^+$ [M+H]$^+$ 360.1354, found 360.1365.

3-(2-(4-Fluorobenzylidene)hydrazine-1-carbonyl)azetidine-1-carboxylate (1l)

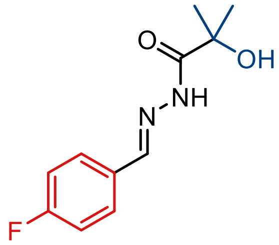

1l

In a 20 mL vial, *tert*-butyl 3-(hydrazinecarbonyl)azetidine-1-carboxylate (323 mg, 1.50 mmol, 1 equiv.) and 4-fluorobenzaldehyde (161 µL, 186 mg, 1.50 mmol, 1 equiv.) were added, followed by acetonitrile (5 mL). The heterogeneous mixture was sonicated for 5 min at room temperature. The reaction mixture was stirred for 3 h at room temperature then transferred to a round-bottom flask. Florisil® was added to the flask and the mixture was concentrated *in vacuo* to give a yellow-coloured solid. The crude material was solid loaded with Florisil® onto a 24 g RediSep® silica column, and purified by column chromatography (0 – 100% EtOAc:cyclohexane). The appropriate product-containing fractions were combined and concentrated *in vacuo* to give a white-coloured solid. The solid was further dried in a vacuum oven at 40 °C to afford a mixture of *E*/*Z*-isomers of 3-(2-(4-fluorobenzylidene)hydrazine-1-carbonyl)azetidine-1-carboxylate (385 mg, 1.20 mmol, 80%) as a white-coloured solid.

^{1}H NMR (400 MHz, CDCl$_3$) δ

Isomer 1: 9.74 (br s, 1H), 8.20 (s, 1H), 7.79 – 7.71 (m, 2H), 7.13 – 7.05 (m, 2H), 1.57 (s, 6H)

Isomer 2: 8.55 (s, 0.08H). The remaining peaks could not be discerned due to low concentration.

Ratio (isomer 1:isomer 2): 1:0.08 (8.20 ppm:8.55 ppm). Purity = 95%

^{13}C NMR (101 MHz, CDCl$_3$) δ 171.8, 164.5 (d, J_{CF} = 244.0 Hz), 129.8 (d, J_{CF} = 9.2 Hz), 116.0 (d, J_{CF} = 22.9 Hz), 74.3, 28.3. 2C not observed. The peaks corresponding to isomer 2 were not observed due to low concentration.

^{19}F NMR (376 MHz, CDCl$_3$) δ

Isomer 2: -108.46 (s, 0.08F)

Isomer 1: -109.22 (s, 1F)

LCMS: t_r = 0.70 min (HpH), ES+ (m/z) 225.08 ($[M+H]^+$, 100%), ES- (m/z) 223.26 ($[M-H]^-$, 100%), Purity = 100%.

FTIR: v_{max} / cm^{-1} 3383 br,w, 3307m, 3072w, 2969w, 2937w, 1654s.

HRMS: m/z Calcd. for $C_{22}H_{26}F_2N_4O_4Na^+$ $[2M+Na]^+$ 471.1814, found 471.1823.

***N*'-(4-Fluorobenzylidene)pivalohydrazide (1m)**

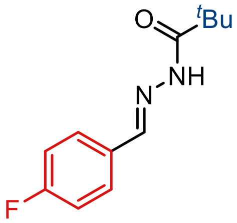

1m

In a 20 mL vial, pivalohydrazide (174 mg, 1.50 mmol, 1 equiv.) and 4-fluorobenzaldehyde (161 µL, 186 mg, 1.50 mmol, 1 equiv.) were added, followed by acetonitrile (5 mL). The heterogeneous mixture was sonicated for 5 min at room temperature. The reaction mixture was sonicated for 15 min at room temperature. The solvent was evaporated in a blowdown at 40 °C to give a white-coloured solid. The solid was further dried in a vacuum oven at 40 °C to afford a mixture of *E*/*Z*-isomers of *N*'-(4-fluorobenzylidene)pivalohydrazide (308 mg, 1.39 mmol, 92%) as a white-coloured solid.

^{1}H NMR (400 MHz, $CDCl_3$) δ

Isomer 1: 8.64 (br s, 1H), 8.32 (br s, 1H), 7.78 – 7.65 (m, 2H), 7.08 (t, *J* = 8.7 Hz, 2H), 1.31 (br s, 9H).

Isomer 2: 8.48 (br d, *J* = 2.7 Hz, 0.05H). The remaining peaks could not be discerned due to low concentration.

Ratio (isomer 1:isomer 2): 1:0.05 (8.20 ppm:8.55 ppm). Purity = 95%

^{13}C NMR (101 MHz, $CDCl_3$) δ

Isomer 1: 174.8, 164.1 (d, J_{CF} = 250.2 Hz), 147.3, 130.2 (d, J_{CF} = 3.1 Hz), 129.6 (d, J_{CF} = 7.6 Hz), 116.0 (d, J_{CF} = 21.4 Hz), 38.8, 27.6.

Isomer 2: 174.5, 38.2, 27.2. 5C not observed.

^{19}F NMR (376 MHz, $CDCl_3$) δ

Isomer 1: -109.52 (s, 1F)

Isomer 2: -110.03 (s, 0.05F)

LCMS: t_r = 0.89 min (HpH), ES+ (m/z) 223.10 ($[M+H]^+$, 100%), ES- (m/z) 221.12 ($[M-H]^-$, 100%), Purity = 100%

FTIR: v_{max} / cm^{-1} 3228w, 3193w, 3050w, 2965m, 2911w, 1646s, 1605s.

HRMS: m/z Calcd. for $C_{24}H_{30}F_2N_4O_2Na^+$ $[2M+Na]^+$ 467.2229, found 467.2234.

N'-(4-Fluorobenzylidene) benzohydrazide (1n)

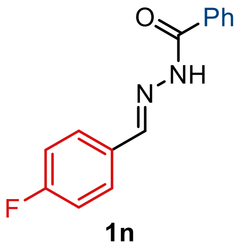

1n

In a flask equipped with a stirrer bar, benzohydrazide (136 mg, 1.00 mmol, 1 equiv.), 4-fluorobenzaldehyde (108 µL, 125 mg, 1.00 mmol, 1 equiv.), acetonitrile (2 mL) and MeOH (1 mL) were added to give a heterogeneous mixture. The reaction mixture was stirred at room temperature for 5 h. Florisil® was added to the reaction mixture and the mixture was concentrated *in vacuo* to give a white-coloured solid. The crude material was solid loaded with Florisil® onto a 24 g RediSep® silica column and purified by column chromatography (0 – 100% EtOAc:cyclohexane). The appropriate fractions were combined and concentrated *in vacuo* to afford (*E*)-*N*'-(4-fluorobenzylidene) benzohydrazide (211 mg, 0.87 mmol, 87%) as a white-coloured solid.

^{1}H NMR (400 MHz, DMSO-d$_6$) δ 11.86 (s, 1H), 8.46 (s, 1H), 7.91 (br d, *J* = 7.4 Hz, 2H), 7.84 – 7.73 (m, 2H), 7.64 – 7.48 (m, 3H), 7.30 (br t, *J* = 8.7 Hz, 2H). Purity = 95%

^{13}C NMR (101 MHz, DMSO-d$_6$) δ 163.1, 163.1 (d, J_{CF} = 247.2 Hz), 146.6, 133.4, 131.7, 130.9 (d, J_{CF} = 3.1 Hz), 129.2 (d, J_{CF} = 9.2 Hz), 128.5, 127.6, 115.9 (d, J_{CF} = 22.9 Hz).

^{19}F NMR (376 MHz, DMSO-d$_6$) δ -110.59 (s, 1F).

LCMS: 0.90 min (Formic), ES+ (m/z) 243.08 ([M+H]$^+$, 100%), ES- (m/z) 241.23 ([M-H]$^-$, 100%), Purity = 99%.

FTIR: ν_{max} / cm^{-1} 3203w, 3180w, 3062w, 3031w, 1639s, 1601s.

HRMS: m/z Calcd. for $C_{28}H_{22}F_2N_4O_2Na^+$ [2M+Na]$^+$ 507.1603, found 507.1617.

Spectroscopic data consistent with literature.[2]

N'-(4-Fluorobenzylidene)-2-iodobenzohydrazide (1o)

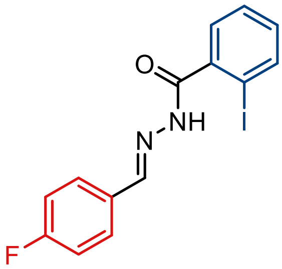

1o

The synthesis of **1o** was conducted following the general procedure P1 using 2-iodobenzohydrazide (393 mg, 1.50 mmol, 1 equiv.) and 4-fluorobenzaldehyde (162 µL, 187 mg, 1.50 mmol, 1 equiv.). The reaction mixture was stirred for 1 h at room temperature then filtered, washed with EtOH, and dried under vacuum to give a white-coloured solid. The solid was further dried in a vacuum oven at 30 °C to

afford a mixture of *E*/*Z*-isomers of *N*'-(4-fluorobenzylidene)-2-iodobenzohydrazide (211 mg, 0.57 mmol, 38%) as an off-white-coloured solid.

^{1}H NMR (400 MHz, DMSO-d$_6$) δ

Isomer 1: 11.85 (br s, 1H), 8.28 (s, 1H), 7.95 (d, *J* = 7.9 Hz, 1H), 7.80 (dd, *J* = 8.7, 5.8 Hz, 2H), 7.56 – 7.39 (m, 2H), 7.37 – 7.16 (m, 3H).

Isomer 2: 11.95 (br s, 1H), 8.07 (s, 1H), 7.90 (d, *J* = 7.9 Hz, 1H), 7.56 – 7.39 (m, 3H), 7.37 – 7.16 (m, 4H).

Ratio (isomer 1:isomer 2): 0.65:0.35 (8.28 ppm:8.07 ppm). Purity = 95%

^{13}C NMR (101 MHz, DMSO-d$_6$) δ 170.8, 164.9, 163.2 (d, J_{CF} = 248.7 Hz), 162.9 (d, J_{CF} = 248.7 Hz), 146.7, 142.7, 142.3, 141.3, 139.2, 138.3, 131.3, 130.7 (d, J_{CF} = 3.1 Hz), 130.3, 129.4 (d, J_{CF} = 7.6 Hz), 128.7 (d, J_{CF} = 9.2 Hz), 128.5, 128.2, 128.1, 127.7 115.9 (d, J_{CF} = 21.4 Hz), 115.8 (d, J_{CF} = 22.9 Hz), 109.5, 94.0, 93.6. ^{13}C NMR data corresponds to a mixture of isomers.

^{19}F NMR (376 MHz, DMSO-d$_6$) δ

Isomer 1: -110.34 (s, 1F)

Isomer 2: -110.82 (s, 0.52F)

LCMS: t_r = 1.04 min (HpH), ES+ (m/z) 368.80 ([M+H]$^+$, 100%), ES- (m/z) 366.99 ([M+H]$^-$, 100%), Purity = 98%.

FTIR: ν_{max} / cm^{-1} 3143w, 3062w, 3047w, 2980w, 1673vs.

HRMS: m/z Calcd. for $C_{14}H_{11}FIN_2O^+$ [M+H]$^+$ 368.9895, found 368.9900.

5-Bromo-2-chloro-*N*'-(4-fluorobenzylidene)benzohydrazide (1p)

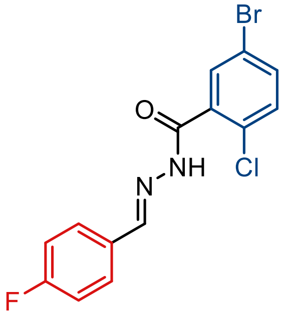

1p

The synthesis of **1p** was conducted following the general procedure P1 using 5-bromo-2-chlorobenzohydrazide (374 mg, 1.50 mmol, 1 equiv.) and 4-fluorobenzaldehyde (162 µL, 187 mg, 1.50 mmol, 1 equiv.). The reaction mixture was filtered, washed with EtOH, and dried under vacuum to give a white-coloured solid. The solid was further dried in a vacuum oven at 30 °C to afford a mixture of *E*/*Z*-isomers of 5-bromo-2-chloro-*N*'-(4-fluorobenzylidene)benzohydrazide (319 mg, 0.90 mmol, 60%) as a white-coloured solid.

^{1}H NMR (400 MHz, DMSO-d$_6$) δ

Isomer 1: 11.98 (s, 1H), 8.28 (s, 1H), 7.84 (d, *J* = 2.4 Hz, 1H), 7.83 – 7.78 (m, 2H), 7.73 (dd, *J* = 8.8, 2.4 Hz, 1H), 7.55 (d, *J* = 8.3 Hz, 1H), 7.35 – 7.28 (m, 2H).

Isomer 2: 12.08 (br s, 1H), 8.08 (s, 0.66H), 7.71 – 7.66 (m, 1.32H), 7.52 (dd, J = 8.3, 1.0 Hz, 0.66H), 7.47 – 7.42 (m, 1.32H), 7.25 – 7.18 (m, 1.32H).

Ratio (isomer 1:isomer 2): 1:0.66 (8.28 ppm:8.08 ppm). Purity = 95%

^{13}C NMR (101 MHz, DMSO-d$_6$) δ 167.0, 163.3 (d, J_{CF} = 247.2 Hz), 163.0 (d, J_{CF} = 247.2 Hz), 160.9, 147.3, 143.5, 137.9, 136.9, 134.1, 133.3, 131.7, 131.1, 131.0, 130.5 (d, J_{CF} = 3.1 Hz), 130.4 (d, J_{CF} = 3.1 Hz), 129.9, 129.5 (d, J_{CF} = 9.2 Hz), 129.2, 128.8 (d, J_{CF} = 7.6 Hz), 120.0, 120.0, 115.9 (d, J_{CF} = 22.9 Hz), 115.9 (d, J_{CF} = 22.9 Hz). 1C not observed.

^{19}F NMR (376 MHz, DMSO-d$_6$) δ

Isomer 1: -110.12 (s, 1F)

Isomer 2: -110.53 (s, 0.66F)

LCMS: t_r = 1.14 min (HpH), ES+ (m/z) 354.76 and 356.76 ([M+H]$^+$, 100%), ES- (m/z) 352.96 and 354.92 ([M-H]$^-$, 100%), Purity = 99%

FTIR: ν_{max} / cm^{-1} 3180w, 3074w, 2957w, 2862w, 1673vs.

HRMS: m/z Calcd. for $C_{14}H_{10}BrClFN_2O^+$ [M+H]$^+$ 354.9644, found 354.9649.

m/z Calcd. for $C_{14}H_{10}BrClFIN_2O^+$ [M+H]$^+$ 356.9624, found 356.9627.

***N*'-(4-Fluorobenzylidene)-4-nitrobenzohydrazide (1q)**

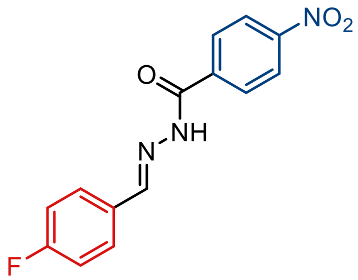

1q

In a 20 mL vial, 4-nitrobenzohydrazide (272 mg, 1.50 mmol, 1 equiv.) and 4-fluorobenzaldehyde (161 µL, 186 mg, 1.50 mmol, 1 equiv.) were added, followed by acetonitrile (2.5 mL) and EtOH (2.5 mL). The heterogeneous mixture was sonicated for 10 min at room temperature. HCl in 1,4-dioxane (4 M, 100 µL, 0.4 mmol, 0.27 equiv.) was added and the reaction mixture was stirred for 3 h at room temperature. The reaction mixture was filtered, washed with acetonitrile, and dried under vacuum to give a yellow-coloured solid. The solid was further dried in a vacuum oven at 40 °C to afford a mixture of *E*/*Z*-isomers of *N*'-(4-fluorobenzylidene)-4-nitrobenzohydrazide (313 mg, 1.09 mmol, 73%) as a yellow-coloured solid.

^{1}H NMR (400 MHz, DMSO-d$_6$) δ

Isomer 1: 12.15 (s, 1H), 8.48 (s, 1H), 8.38 (d, J = 8.6 Hz, 2H), 8.15 (d, J = 8.9 Hz, 2H), 7.82 (dd, J = 8.6, 5.7 Hz, 2H), 7.32 (t, J = 8.9 Hz, 2H)

Isomer 2: 12.04 (br s, 0.14H), 8.34 (s, 0.14H), 8.08 (d, J = 8.6 Hz, 0.28H), 7.99 (br d, J = 8.6 Hz, 0.28H), 7.59 (br t, J = 6.4 Hz, 0.28H), 7.24 (br t, J = 9.0 Hz, 0.28H)

Ratio (isomer 1:isomer 2): 1:0.14 (8.48 ppm:8.34 ppm). Purity = 95%

^{13}C NMR (101 MHz, DMSO-d$_6$) δ 163.3 (d, J_{CF} = 248.7 Hz), 161.5, 149.3, 147.8, 139.0, 130.7 (d, J_{CF} = 3.1 Hz), 129.4 (d, J_{CF} = 7.6 Hz), 129.1, 123.6, 115.9 (d, J_{CF} = 21.4 Hz).

^{19}F NMR (376 MHz, DMSO-d$_6$) δ -110.12 (s, 1F)

LCMS: t_r = 0.97 min (HpH), ES+ (m/z) 287.85 ([M+H]$^+$, 100%), ES- (m/z) 286.08 ([M-H]$^-$, 100%), Purity = 94%

FTIR: $ν_{max}$ / cm^{-1} 3257w, 3185w, 3067w, 2995w, 2846w, 1651s, 1601vs, 1521vs, 1508vs, 1348s.

HRMS: m/z Calcd. for $C_{14}H_{11}FN_3O_3^+$ [M+H]$^+$ 288.0779, found 288.0791.

3-(2-(4-Fluorobenzylidene)hydrazine-1-carbonyl)-*N*,*N*-dimethylbenzene sulfonamide (1r)

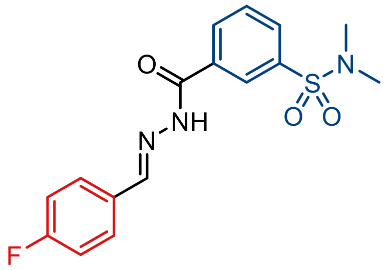

1r

The synthesis of **1r** was conducted following the general procedure P1 using 3-(hydrazinecarbonyl)-*N*,*N*-dimethylbenzenesulfonamide (365 mg, 1.50 mmol, 1 equiv.) and 4-fluorobenzaldehyde (162 µL, 187 mg, 1.50 mmol, 1 equiv.). The reaction mixture was filtered, washed with EtOH, and dried under vacuum to give a white-coloured solid. The solid was further dried in a vacuum oven at 30 °C to afford a mixture of *E*/*Z*-isomers of 3-(2-(4-fluorobenzylidene)hydrazine-1-carbonyl)-*N*,*N*-dimethylbenzene sulfonamide (473 mg, 1.35 mmol, 90%) as a white-coloured solid.

^{1}H NMR (400 MHz, DMSO-d$_6$) δ

Isomer 1: 12.09 (br s, 1H), 8.50 (s, 1H), 8.30 – 8.23 (m, 2H), 7.96 (br d, *J* = 7.8 Hz, 1H), 7.86 – 7.78 (m, 3H), 7.32 (br t, *J* = 8.8 Hz, 2H), 2.66 (s, 6H)

Isomer 2: 8.22 – 8.17 (m, 0.33H), 8.16 – 8.10 (m, 0.33H), 7.67 – 7.57 (m, 0.33H), 7.27 – 7.19 (m, 0.33H). The remaining peaks could not be discerned due to overlap.

Ratio (isomer 1:isomer 2): 1:0.33 (8.50 ppm:8.20 ppm). Purity = 95%

^{13}C NMR (101 MHz, CDCl$_3$) δ 163.2 (d, J_{CF} = 247.2 Hz), 161.6, 147.6, 135.3, 134.3, 132.1, 130.7, 130.4, 129.8, 129.4 (d, J_{CF} = 7.6 Hz), 126.3, 115.9 (d, J_{CF} = 22.9 Hz), 37.6.

^{19}F NMR (376 MHz, DMSO-d$_6$) δ

Isomer 2: -110.52 (s, 0.12F)

Isomer 1: -110.25 (s, 1F)

LCMS: t_r = 0.95 min (HpH), ES+ (m/z) 349.91 ([M+H]$^+$, 100%), ES- (m/z) 348.06 ([M-H]$^-$, 100%), Purity = 100%

FTIR: ν_{max} / cm^{-1} 3195w, 3061w, 2979w, 1651s.

HRMS: m/z Calcd. for $C_{16}H_{17}FN_3O_3S^+$ $[M+H]^+$ 350.0969, found 350.0972.

***N*'-(4-Fluorobenzylidene)isonicotinohydrazide (1s)**

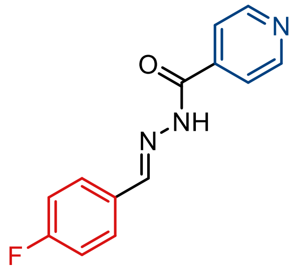

1s

In a 20 mL vial, isonicotinohydrazide (205.7 mg, 1.50 mmol, 1 equiv.) and 4-fluorobenzaldehyde (161 µL, 186 mg, 1.50 mmol, 1 equiv.) were added, followed by EtOH (5 mL). HCl in 1,4-dioxane (4 M, 5 µL, 20 µmol, 0.01 equiv.) was added and the reaction mixture was stirred for 1 h at room temperature. The reaction mixture was filtered, washed with EtOH, and dried under vacuum to give a yellow-coloured solid. The solid was further dried in a vacuum oven at 40 °C to afford a mixture of *E*/*Z*-isomers of *N*'-(4-fluorobenzylidene)isonicotinohydrazide (306 mg, 1.26 mmol, 84%) as a yellow-coloured solid.

^{1}H NMR (400 MHz, DMSO-d_6) δ

Isomer 1: 12.09 (s, 1H), 8.85 – 8.76 (m, 2H), 8.48 (s, 1H), 7.87 – 7.78 (m, 4H), 7.31 (t, *J* = 8.9 Hz, 2H).

Isomer 2: 12.03 (br s, 0.16H), 8.74 (br d, *J* = 4.7 Hz, 0.32H), 8.10 (s, 0.16H), 7.67 (br d, *J* = 4.9 Hz, 0.32H), 7.61 – 7.54 (m, 0.32H), 7.26 – 7.20 (m, 0.32H)

Ratio (isomer 1:isomer 2) = 1:0.16 (8.47 ppm:8.10 ppm). Purity = 95%

^{13}C NMR (101 MHz, DMSO-d_6) δ

Isomer 1: 163.3 (d, J_{CF} = 248.7 Hz), 161.6, 150.3, 147.9, 140.5, 130.6 (d, J_{CF} = 3.1 Hz), 129.5 (d, J_{CF} = 9.2 Hz), 121.5, 116.0 (d, J_{CF} = 22.9 Hz).

Isomer 2: 149.4, 143.6, 129.0 (d, J_{CF} = 7.6 Hz), 123.12. 5C not observed.

^{19}F NMR (376 MHz, DMSO-d_6) δ

Isomer 1: -110.11 (s, 1F)

Isomer 2: -110.55 (s, 0.16F)

LCMS: t_r = 0.75 min (HpH), ES+ (m/z) 243.98 ($[M+H]^+$, 100%), ES- (m/z) 242.12 ($[M-H]^-$, 100%), Purity = 100%.

FTIR: ν_{max} / cm^{-1} 3471br,w, 3254br,w, 3043w, 2922w, 2846w, 1655vs, 1551s, 1293vs.

HRMS: m/z Calcd. for $C_{14}H_{11}FN_3O_3^+$ $[M+H]^+$ 244.0881, found 244.0894.

Spectroscopic data consistent with literature.[3]

N'-(4-Fluorobenzylidene)-1H-indole-3-carbohydrazide (1t)

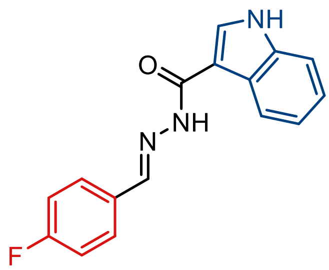

1t

In a 20 mL vial, 1*H*-indole-3-carbohydrazide (263 mg, 1.50 mmol, 1 equiv.) and 4-fluorobenzaldehyde (161 µL, 186 mg, 1.50 mmol, 1 equiv.) were added, followed by acetonitrile (5 mL). The heterogeneous mixture was sonicated for 10 min at room temperature. HCl in 1,4-dioxane (4 M, 0.1 mL, 0.40 mmol, 0.27 equiv.) was added and the reaction mixture was stirred for 6 h at room temperature. The reaction mixture was transferred to a 100 mL flask, Florisil® was added, and the mixture was concentrated *in vacuo* to give a yellow-coloured solid. The crude material was solid loaded with Florisil® onto a 12 g RediSep® silica column, and purified by column chromatography (0 – 100% EtOAc:cyclohexane). The appropriate fractions were combined and concentrated *in vacuo* to give a white-coloured solid. The solid was further dried in a vacuum oven at 40 °C to afford *N*'-(4-fluorobenzylidene)-1*H*-indole-3-carbohydrazide (298 mg, 1.06 mmol, 71%) as an off-white-coloured solid.

^{1}H NMR (600 MHz, DMSO-d$_6$) δ 11.73 (br s, 1H), 11.39 (br s, 1H), 8.21 (br d, *J* = 4.8 Hz, 1H), 8.46 – 8.15 (m, 1H), 7.81 – 7.74 (m, 2H), 7.48 (d, *J* = 7.7 Hz, 1H), 7.32 – 7.27 (m, 2H), 7.21 – 7.17 (m, 1H), 7.17 – 7.13 (m, 1H). Purity = 95%.

^{13}C NMR (151 MHz, DMSO-d$_6$) δ 162.8 (d, J_{CF} = 247.1 Hz), 161.0 (br s), 143.5 (br s), 135.9 (br s), 131.4 (d, J_{CF} = 2.8 Hz), 128.8 (d, J_{CF} = 8.8 Hz), 126.6, 122.2, 121.1 (br s), 120.7, 115.9 (d, J_{CF} = 22.1 Hz), 111.9, 108.5 (br s). 1C not observed.

^{19}F NMR (376 MHz, DMSO-d$_6$) δ -111.45 (s, 1F)

LCMS: t_r = 0.93 min (HpH), ES+ (m/z) 281.94 ([M+H]$^+$, 100%), ES- (m/z) 280.11 ([M-H]$^-$, 100%), Purity = 99%

FTIR: ν_{max} / cm^{-1} 3357w, 3143br,m, 3115m, 3068m, 3043m, 2997w, 1644s.

HRMS: m/z Calcd. for $C_{16}H_{13}FN_3O^+$ [M+H]$^+$ 282.1037, found 282.1047.

Spectroscopic data consistent with literature.[4]

N'-(4-Fluorobenzylidene)-1-methyl-1H-pyrazole-3-carbohydrazide (1u)

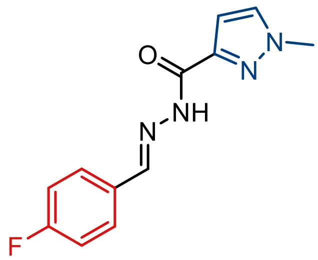

1u

The synthesis of **1u** was conducted following the general procedure P1 using 1-methyl-1*H*-pyrazole-3-carbohydrazide (210 mg, 1.50 mmol, 1 equiv.) and 4-fluorobenzaldehyde (162 µL, 187 mg, 1.50 mmol, 1 equiv.). The reaction mixture was filtered, washed with EtOH, and dried under vacuum to afford *N*'-(4-fluorobenzylidene)-1-methyl-1*H*-pyrazole-3-carbohydrazide (61.3 mg, 0.22 mmol, 14%, 87% purity) as a yellow-coloured solid.

^{1}H NMR (400 MHz, $CDCl_3$) δ 9.86 (s, 1H), 8.22 (s, 1H), 7.80 – 7.74 (m, 2H), 7.41 (d, *J* = 2.2 Hz, 1H), 7.12 – 7.06 (m, 2H), 6.93 (d, *J* = 2.5 Hz, 1H), 3.96 (s, 3H). Purity = 87%

^{13}C NMR (101 MHz, $CDCl_3$) δ 178.2, 162.1 (d, J_{CF} = 253.3 Hz), 157.9, 146.1, 132.0, 130.5, 129.6 (d, J_{CF} = 7.6 Hz), 115.8 (d, J_{CF} = 21.4 Hz), 107.9, 39.4.

^{19}F NMR (376 MHz, $CDCl_3$) δ -109.66 (s, 1F).

LCMS: t_r = 0.78 min (HpH), ES+ (m/z) 246.97 ([M+H]$^+$, 100%), ES- (m/z) 245.19 ([M+H]$^-$, 100%), Purity = 84%

FTIR: v_{max} / cm^{-1} 3059w, 3039w, 3015w, 2938w, 1631s.

HRMS: m/z Calcd. for $C_{12}H_{11}FN_4ONa^+$ [M+Na]$^+$ 269.0809, found 269.0821

***N*'-(4-Fluorobenzylidene)-3,5-dimethoxybenzohydrazide (1v)**

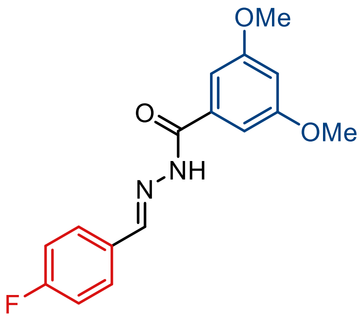

1v

The synthesis of **1v** was conducted following the general procedure P1 using 3,5-dimethoxybenzohydrazide (294 mg, 1.50 mmol, 1 equiv.) and 4-fluorobenzaldehyde (162 µL, 187 mg, 1.50 mmol, 1 equiv.). The reaction mixture was filtered, washed with EtOH, and dried under vacuum to afford *N*'-(4-fluorobenzylidene)-3,5-dimethoxybenzohydrazide (436 mg, 1.44 mmol, 96%) as a white-coloured solid.

^{1}H NMR (400 MHz, DMSO-d_6) δ 11.75 (s, 1H), 8.46 (s, 1H), 7.79 (br dd, *J* = 8.6, 5.8 Hz, 2H), 7.30 (t, *J* = 8.6 Hz, 2H), 7.06 (d, *J* = 2.1 Hz, 2H), 6.72 (t, *J* = 2.1 Hz, 1H). Purity = 95%.

^{13}C NMR (101 MHz, DMSO-d_6) δ 163.1 (d, J_{CF} = 247.2 Hz), 162.6, 160.4, 146.8, 135.4, 130.9 (d, J_{CF} = 3.1 Hz), 129.2 (d, J_{CF} = 9.2 Hz), 115.9 (d, J_{CF} = 21.4 Hz), 105.5, 103.5, 55.5.

^{19}F NMR (376 MHz, DMSO-d_6) δ -110.57 (s, 1F).

LCMS: t_r = 1.00 min (HpH), ES+ (m/z) 302.95 ([M+H]$^+$, 100%), ES- (m/z) 301.05 ([M+H]$^-$, 100%), Purity = 99%

FTIR: v_{max} / cm^{-1} 3229w, 3080w, 3064w, 3001w, 2971w, 2939w, 1645m, 1603s.

HRMS: m/z Calcd. for $C_{16}H_{16}FN_2O_3^+$ [M+H]$^+$ 303.1139, found 303.1149.

_N_'-(4-Fluorobenzylidene)-2-(methylsulfonyl)acetohydrazide (1w)

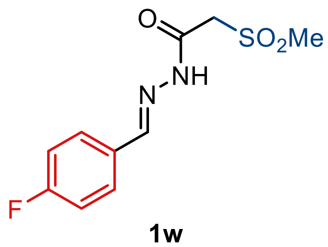

1w

The synthesis of **1w** was conducted following the general procedure P1 using 2-(methylsulfonyl)acetohydrazide (228 mg, 1.50 mmol, 1 equiv.) and 4-fluorobenzaldehyde (162 µL, 187 mg, 1.50 mmol, 1 equiv.). The reaction mixture was filtered, washed with EtOH, and dried under vacuum to afford *E*/*Z*-isomers of *N*'-(4-fluorobenzylidene)-2-(methylsulfonyl)acetohydrazide (327 mg, 1.27 mmol, 84%) as a white-coloured solid.

^{1}H NMR (400 MHz, DMSO-d$_6$) δ

Isomer 1: 11.82 (br s, 1H), 8.04 (s, 1H), 7.79 (dd, *J* = 8.2, 5.8 Hz, 2H), 7.29 (q, *J* = 8.9 Hz, 2H), 4.68 (s, 2H), 3.16 (s, 3H).

Isomer 2: 9.42 (br s, 1H), 8.21 (s, 0.85H), 8.04 (s, 1H), 7.79 (dd, *J* = 8.2, 5.8 Hz, 1.7H), 7.29 (q, *J* = 8.9 Hz, 1.7H), 4.19 (s, 2H), 3.16 (s, 2.55H).

Ratio (isomer 1:isomer 2): 1:0.85 (8.04 ppm:8.21 ppm). Purity = 95%

^{13}C NMR (101 MHz, DMSO-d$_6$) δ 164.1, 163.3 (d, J_{CF} = 247.2 Hz), 163.1 (d, J_{CF} = 248.7 Hz), 158.4, 147.1, 143.4, 130.4 (d, J_{CF} = 3.1 Hz), 129.5 (d, J_{CF} = 7.6 Hz), 129.3 (d, J_{CF} = 9.2 Hz), 115.9 (br d, J_{CF} = 22.9 Hz), 115.9 (br d, J_{CF} = 21.4 Hz), 58.9, 57.9, 56.0, 42.1, 41.6. ^{13}C NMR data corresponds to a mixture of isomers.

^{19}F NMR (376 MHz, DMSO-d$_6$) δ

Isomer 2: -110.02 (s, 0.85F)

Isomer 1: -110.43 (s, 1F)

LCMS: t_r = 0.70 min (HpH), ES+ (m/z) 258.95 ([M+H]$^+$, 100%), ES- (m/z) 257.04 ([M+H]$^-$, 100%), Purity = 100%

FTIR: v_{max} / cm^{-1} 3232w, 3047w, 2991w, 2937w, 2845w, 1667s.

HRMS: m/z Calcd. for $C_{10}H_{12}FN_2O_3S^+$ [M+H]$^+$ 259.0561, found 259.0547.

N'-(4-Fluorobenzylidene)morpholine-4-carbohydrazide (1x)

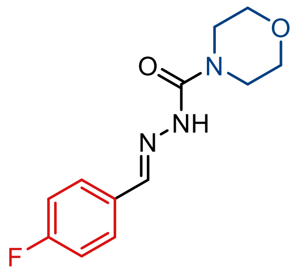

1x

In a round-bottom flask equipped with a stirrer bar, morpholine-4-carbohydrazide (145 mg, 1.00 mmol, 1 equiv.), 4-fluorobenzaldehyde (108 µL, 125 mg, 1.00 mmol, 1 equiv.), acetonitrile (2 mL) and EtOH (2 mL) were added to give a heterogeneous mixture. The reaction mixture was stirred at room temperature for 3 h. Florisil® was added to the flask and the mixture was concentrated *in vacuo* to give a white-coloured solid. The reaction mixture was loaded with Florisil® onto a 24 g RediSep® silica column and purified by column chromatography (0 – 100% EtOAc:cyclohexane). The appropriate fractions were combined and concentrated *in vacuo* to afford *N*'-(4-fluorobenzylidene)morpholine-4-carbohydrazide (176 mg, 0.70 mmol, 70%) as a white-coloured solid.

^{1}H NMR (400 MHz, $CDCl_3$) δ 7.73 (s, 1H), 7.69 (s, 1H), 7.62 – 7.56 (m, 2H), 7.12 – 7.03 (m, 2H), 3.80 – 3.73 (m, 4H), 3.69 – 3.63 (m, 4H). Purity = 95%

^{13}C NMR (101 MHz, $CDCl_3$) δ 163.6 (d, J_{CF} = 248.7 Hz), 156.4, 141.7, 130.3 (d, J_{CF} = 4.6 Hz), 128.7 (d, J_{CF} = 9.2 Hz), 115.9 (br d, J_{CF} = 22.9 Hz), 66.7, 45.7.

^{19}F NMR (376 MHz, $CDCl_3$) δ -110.33 (s, 1F).

LCMS: t_r = 0.67 min (Formic), ES+ (m/z) 252.01 ($[M+H]^+$, 100%), ES- (m/z) 250.11 ($[M-H]^-$, 100%), Purity = 83%

FTIR: ν_{max} / cm^{-1} 3399br,w, 3203br,w, 3029w, 2960w, 2910w, 2855w, 1628s, 1602s.

HRMS: m/z Calcd. for $C_{24}H_{28}F_2N_6O_4Na^+$ $[2M+Na]^+$ 525.2032, found 525.2052.

2-(4-Fluorobenzylidene)hydrazine-1-carbothioamide (1y)

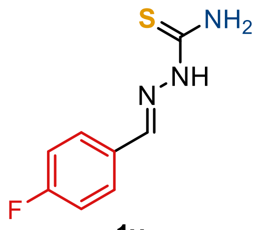

1y

In a round-bottom flask equipped with a magnetic stirrer bar, thiosemicarbazide (137 mg, 1.50 mmol, 1 equiv.), 4-fluorobenzaldehyde (169 µL, 195 mg, 1.57 mmol, 1.05 equiv.), and EtOH (5 mL) were added. The reaction mixture was stirred at room temperature for 8 h. The reaction mixture was filtered, washed with ethanol, and dried under vacuum to give a white-coloured solid. The solid was transferred to a 20 mL vial and further dried in a vacuum oven at 40 °C to afford (*E*)-2-(4-fluorobenzylidene)hydrazine-1-carbothioamide (283 mg, 1.44 mmol, 96%) as a white-coloured solid.

^{1}H NMR (400 MHz, DMSO-d$_6$) δ 11.40 (br s, 1H), 8.17 (br s, 1H), 8.04 (s, 1H), 8.01 (s, 1H), 7.91 – 7.84 (m, 2H), 7.28 – 7.19 (m, 2H). Purity = 95%

^{13}C NMR (101 MHz, DMSO-d$_6$) δ 178.0, 163.0 (d, J_{CF} = 247.2 Hz), 141.0, 130.8 (d, J_{CF} = 3.1 Hz), 129.5 (d, J_{CF} = 7.6 Hz), 115.6 (d, J_{CF} = 22.9 Hz).

^{19}F NMR (376 MHz, DMSO-d$_6$) δ -110.94 (s, 1F).

LCMS: t_r = 0.80 min (HpH), ES+ (m/z) 198.01 ([M+H]$^+$, 100%), Purity = 94%.

FTIR: v_{max} / cm^{-1} 3388m, 3232m, 3152br, m, 3020w, 2986w, 1599s, 1505s.

HRMS: m/z Calcd. for $C_8H_9FN_3S^+$ [M+H]$^+$ 198.0496, found 198.0515.

Spectroscopic data consistent with literature.[5]

***tert*-Butyl (*E*)-2-(4-fluorobenzylidene)hydrazine-1-carboxylate (1z)**

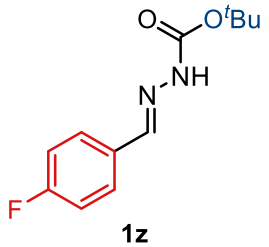

1z

In a round-bottom flask equipped with a stirrer bar, *tert*-butyl hydrazinecarboxylate (668 mg, 5.05 mmol, 1 equiv.), 4-fluorobenzaldehyde (570 µL, 656 m, 5.28 mmol, 1.04 equiv.), and MeOH (10 mL) were added to give a heterogeneous mixture. The reaction mixture was stirred at room temperature for 30 min. The reaction mixture was transferred to a 20 mL vial and the solvent was evaporated in a blowdown unit to give a white-coloured solid. The solid was further dried in a vacuum oven at 40 °C to afford *tert*-butyl (*E*)-2-(4-fluorobenzylidene)hydrazine-1-carboxylate (1.20 g, 5.03 mmol, 99%) as a white-coloured solid.

^{1}H NMR (400 MHz, $CDCl_3$) δ 7.83 (br s, 1H), 7.82 (br s, 1H), 7.70 – 7.63 (m, 2H), 7.09 – 7.02 (m, 2H), 1.54 (s, 9H).

^{13}C NMR (101 MHz, $CDCl_3$) δ 163.9 (d, J_{CF} = 250.2 Hz), 152.7, 142.6 (br s), 130.4 (d, J_{CF} = 2.9 Hz), 129.1 (d, J_{CF} = 8.1 Hz), 115.9 (d, J_{CF} = 22.0 Hz), 81.7 (br s), 28.4.

^{19}F NMR (376 MHz, $CDCl_3$) δ -110.49 (s, 1F).

LCMS: t_r = 1.06 min (HpH), ES+ (m/z) 261.02 ([M+Na]$^+$, 50%), ES- (m/z) 237.33 ([M-H]$^-$, 100%), Purity = 100%.

FTIR: v_{max} / cm^{-1} 3231br,w, 3018w, 2976w, 1698s.

HRMS: m/z Calcd. for $C_{12}H_{15}FN_2O_2Na^+$ [M+Na]$^+$ 261.1010, found 261.1027.

tert-Butyl (*E*)-4-((2-benzoylhydrazineylidene)methyl)piperidine-1-carboxylate (3a)

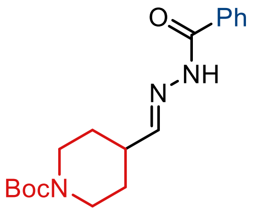

3a

The synthesis of **3a** was conducted following the general procedure P2 using *tert*-butyl 4-formylpiperidine-1-carboxylate (213 mg, 1.00 mmol, 1 equiv.), benzohydrazide (136 mg, 1.00 mmol, 1 equiv.), and MeOH (5 mL). The reaction mixture was transferred to a round-bottom flask and concentrated *in vacuo*. The crude material was liquid loaded with a minimum amount of CH_2Cl_2 onto a 12 g RediSep® silica column, and purified by column chromatography (0 – 40% EtOAc:cyclohexane). The appropriate fractions were combined and concentrated *in vacuo* to afford *tert*-butyl (*E*)-4-((2-benzoylhydrazineylidene)methyl)piperidine-1-carboxylate (164 mg, 0.50 mmol, 49%) as a white-coloured solid.

^{1}H NMR (400 MHz, $CDCl_3$) δ 9.17 (br s, 1H), 7.87 – 7.75 (m, 2H), 7.56 – 7.47 (m, 2H), 7.46 – 7.39 (m, 2H), 4.19 – 4.01 (m, 2H), 2.79 (br t, *J* = 10.3 Hz, 2H), 2.65 – 2.51 (m, 1H), 1.80 (br d, *J* = 11.8 Hz, 2H), 1.70 – 1.58 (m, 2H), 1.45 (s, 9H). Purity = 95%.

^{13}C NMR (101 MHz, $CDCl_3$) δ 164.2 (br s), 154.9, 154.3 (br s), 133.3 (br s), 132.1 (br s), 128.9 (br s), 127.3 (br s), 79.8, 43.4 (br s), 39.4 (br s), 29.3 (br s), 28.6.

LCMS: t_r = 1.00 min (HpH), ES+ (m/z) 232.12 ([M-Boc+2H]$^+$, 100%), ES- (m/z) 330.30 ([M-H]$^-$, 100%), Purity = 98%.

FTIR: ν_{max} / cm^{-1} 3262m, 3058w, 2987w, 2940w, 2852w, 1679s, 1657s.

HRMS: m/z Calcd. for $C_{18}H_{25}N_3O_3Na^+$ [M+Na]$^+$ 354.1788, found 354.1798.

(*E*)-*N*'-(Cyclopropylmethylene)benzohydrazide (3b)

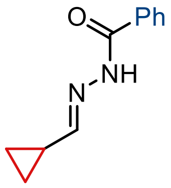

3b

The synthesis of **3b** was conducted following the general procedure P2 using cyclopropanecarbaldehyde (54.8 µL, 51.4 mg, 0.73 mmol, 1 equiv.), benzohydrazide (136 mg, 1.00 mmol, 1.4 equiv.), and MeOH (5 mL). The reaction mixture was transferred to a round-bottom flask and concentrated *in vacuo*. The crude material was liquid loaded with a minimum amount of CH_2Cl_2 onto a 12 g RediSep® silica column, and purified by column chromatography (0 – 45% EtOAc:cyclohexane). The appropriate fractions were combined and concentrated *in vacuo* to afford (*E*)-*N*'-(cyclopropylmethylene)benzohydrazide (43.2 mg, 0.23 mmol, 31%) as a white-coloured solid.

^{1}H NMR (400 MHz, $CDCl_3$) δ 8.60 (br s, 1H), 7.93 – 7.68 (m, 2H), 7.58 – 7.50 (m, 2H), 7.49 – 7.40 (m, 2H), 2.17 (m, 3H), 1.96 (br s, 2H). Purity = 95%.

^{13}C NMR (101 MHz, $CDCl_3$) δ 156.3, 132.0, 128.9, 127.3, 127.0, 25.7, 16.6. 1C not observed.

LCMS: t_r = 0.70 min (HpH), ES+ (m/z) 189.09 ($[M+H]^+$, 100%), ES- (m/z) 187.08 ($[M-H]^-$, 100%), Purity = 100%.

FTIR: v_{max} / cm^{-1} 3189w, 3010w, 2952w, 1655s.

HRMS: m/z Calcd. for $C_{11}H_{12}N_2ONa^+$ $[M+Na]^+$ 211.0842, found 211.0857.

(*E*)-*N*'-(3-(Benzo[*d*][1,3]dioxol-5-yl)-2-methylpropylidene)benzohydrazide (3c)

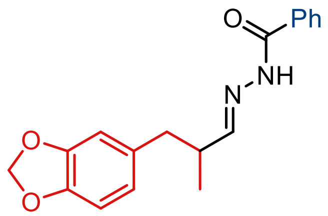

3c

The synthesis of **3c** was conducted following the general procedure P2 using 3-(benzo[*d*][1,3]dioxol-5-yl)-2-methylpropanal (166 µL, 192 mg, 1 equiv.), benzohydrazide (136 mg, 1.00 mmol, 1 equiv.), and MeOH (5 mL). The reaction mixture was transferred to a round-bottom flask and concentrated *in vacuo*. The crude material was liquid loaded with a minimum amount of CH_2Cl_2 onto a 12 g RediSep® silica column, and purified by column chromatography (0 – 50% EtOAc:cyclohexane). The appropriate fractions were combined and concentrated *in vacuo* to afford (*E*)-*N*'-(3-(benzo[*d*][1,3]dioxol-5-yl)-2-methylpropylidene) benzohydrazide (281 mg, 0.90 mmol, 90%) as a white-coloured solid.

^{1}H NMR (400 MHz, $CDCl_3$) δ 9.23 (br s, 1H), 7.79 (br d, *J* = 7.1 Hz, 2H), 7.55 – 7.45 (m, *J* = 7.4, 7.4 Hz, 2H), 7.44 – 7.37 (m, 2H), 6.71 (d, *J* = 7.9 Hz, 1H), 6.64 (s, 1H), 6.59 (br d, *J* = 7.9 Hz, 1H), 5.90 (s, 2H), 2.81 (br d, *J* = 8.9 Hz, 2H), 2.62 – 2.53 (m, 1H), 1.09 (br d, *J* = 6.4 Hz, 3H). Purity = 95%.

^{13}C NMR (101 MHz, $CDCl_3$) δ 164.2, 156.1, 147.7, 146.1, 133.0, 132.0, 128.8, 127.4, 122.2, 109.6, 108.3, 101.0, 40.5, 38.6, 17.4. 1C not observed.

LCMS: t_r = 1.03 min (HpH), ES+ (m/z) 311.09 ($[M+H]^+$, 10%), ES- (m/z) 309.20 ($[M-H]^-$, 100%), Purity = 100%.

FTIR: v_{max} / cm^{-1} 3195w, 3031w, 2961w, 2867w, 1644s.

HRMS: m/z Calcd. for $C_{18}H_{18}N_2O_3Na^+$ $[M+Na]^+$ 333.1210, found 333.1220.

(*E*)-*N*'-Benzylidenebenzohydrazide (3d)

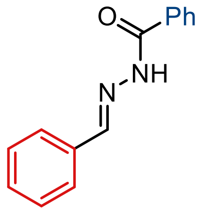

3d

The synthesis of **3d** was conducted following the general procedure P2 using benzaldehyde (102 µL, 106 mg, 1.00 mmol, 1 equiv.), benzohydrazide (136 mg, 1.00 mmol, 1 equiv.), and MeOH (5 mL). The reaction mixture was transferred to a round-bottom flask and concentrated *in vacuo*. The crude material was liquid loaded with a minimum amount of CH_2Cl_2 onto a 12 g RediSep® silica column, and purified by column chromatography (0 – 30% EtOAc:cyclohexane). The appropriate fractions were combined and concentrated *in vacuo* to afford (*E*)-*N*'-benzylidenebenzohydrazide (120 mg, 0.53 mmol, 53%) as a white-coloured solid.

^{1}H NMR (400 MHz, DMSO-d$_6$) δ 11.84 (s, 1H), 8.47 (s, 1H), 7.92 (br d, *J* = 7.4 Hz, 2H), 7.74 (br d, *J* = 6.2 Hz, 2H), 7.63 – 7.50 (m, 3H), 7.50 – 7.42 (m, *J* = 6.6 Hz, 3H). Purity = 95%.

^{13}C NMR (101 MHz, DMSO-d$_6$) δ 163.1, 147.8, 134.3, 133.4, 131.7, 130.0, 128.8, 128.4, 127.6, 127.1.

LCMS: t_r = 0.91 min (HpH), ES+ (m/z) 225.09 ([M+H]$^+$, 100%), ES- (m/z) 223.25 ([M-H]$^-$, 100%), Purity = 99%.

FTIR: v_{max} / cm^{-1} 3178w, 3059w, 3028w, 1639s.

HRMS: m/z Calcd. for $C_{14}H_{12}N_2ONa^+$ [M+Na]$^+$ 247.0842, found 247.0855.

Spectroscopic data consistent with literature.[2]

(*E*)-*N*'-(4-Methylbenzylidene)benzohydrazide (3e)

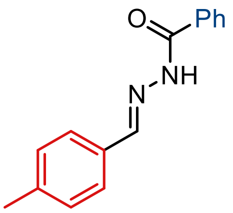

3e

The synthesis of **3e** was conducted following the general procedure P2 using 4-methylbenzaldehyde 118 µL, 120 mg, 1.00 mmol, 1 equiv.), benzohydrazide (136 mg, 1.00 mmol, 1 equiv.), and MeOH (5 mL). The reaction mixture was transferred to a round-bottom flask and concentrated *in vacuo*. The crude material was liquid loaded with a minimum amount of CH_2Cl_2 onto a 12 g RediSep® silica column, and purified by column chromatography (0 – 30% EtOAc:cyclohexane). The appropriate fractions were combined and concentrated *in vacuo* to afford (*E*)-*N*'-(4-methylbenzylidene)benzohydrazide (208 mg g, 0.87 mmol, 87%) as a white-coloured solid.

^{1}H NMR (400 MHz, CDCl$_3$) δ 9.07 (br s, 1H), 8.29 (br s, 1H), 7.86 (br s, 2H), 7.73 – 7.60 (m, 2H), 7.58 – 7.51 (m, 1H), 7.51 – 7.44 (m, 2H), 7.21 (br d, *J* = 7.8 Hz, 2H), 2.38 (s, 3H). Purity = 95%.

^{13}C NMR (101 MHz, CDCl$_3$) δ 164.5, 149.1, 140.9, 133.5, 132.0, 131.1, 129.5, 128.8, 127.9, 127.5, 21.7.

LCMS: t_r = 0.99 min (HpH), ES+ (m/z) 239.16 ([M+H]$^+$, 100%), ES- (m/z) 237.21 ([M-H]$^-$, 100%), Purity = 100%.

FTIR: v_{max} / cm^{-1} 3199w, 3028w, 1640s.

HRMS: m/z Calcd. for $C_{15}H_{14}N_2ONa^+$ [M+Na]$^+$ 261.0998, found 261.1012.

Spectroscopic data consistent with literature.[6]

N'-([1,1'-Biphenyl]-4-ylmethylene)acetohydrazide (3f)

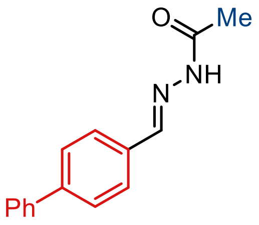

3f

In a round-bottom flask, acetohydrazide (264 mg, 3.56 mmol, 1 equiv.), [1,1'-biphenyl]-4-carbaldehyde (651 mg, 3.57 mmol, 1 equiv.), and acetonitrile (7 mL) were added. The reaction mixture was stirred for 6 h at room temperature. The heterogeneous mixture was filtered, washed with acetonitrile, dried under vacuum, and further dried in a vacuum oven at 40 °C to afford a mixture of *E*/*Z*-isomers of *N*'-([1,1'-biphenyl]-4-ylmethylene)acetohydrazide (661 mg, 2.77 mmol, 78%) as a white-coloured solid.

mp: 201 – 203 °C

^{1}H NMR (400 MHz, $CDCl_3$) δ

Isomer 1: 8.83 (br s, 1H), 7.77 (s, 1H), 7.75 – 7.71 (m, 2H), 7.67 – 7.60 (m, 4H), 7.49 – 7.43 (m, 2H), 7.41 – 7.35 (m, 1H), 2.41 (s, 3H).

Isomer 2: 8.49 (br s, *J* = 0.7 Hz, 1H), 8.17 (s, 1H). The remaining peaks could not be discerned due to a low concentration.

Ratio (isomer 1:isomer 2): 1:0.10 (8.84 ppm:8.18 ppm). Purity = 95%

^{13}C NMR (101 MHz, $CDCl_3$) δ 172.9, 142.8, 140.0, 132.7, 129.1, 128.0, 127.7, 127.6, 127.2, 126.8, 20.6.

LCMS: t_r = 1.03 min (HpH), ES+ (m/z) 239.00 ($[M+H]^+$, 100%), ES- (m/z) 237.13 ($[M-H]^-$, 100%), Purity = 100%

FTIR: ν_{max} / cm^{-1} 3182w, 3060m, 3032m, 2957m, 1671s, 1608m.

HRMS: m/z Calcd. for $C_{15}H_{15}N_2O$ $[M+H]^+$ 239.1189, found 239.1199.

(*E*)-*N*'-(2-Nitrobenzylidene)benzohydrazide (3g)

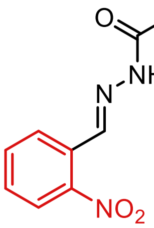

3g

The synthesis of **3g** was conducted following the general procedure P2 using 2-nitrobenzaldehyde (151 mg, 1.00 mmol, 1 equiv.), benzohydrazide (136 mg, 1.00 mmol, 1 equiv.), and MeOH (5 mL). The solvent was evaporated in a blowdown at 40 °C and further dried in a vacuum oven at 40 °C to afford (*E*)-*N*'-(2-nitrobenzylidene)benzohydrazide (265 mg, 0.98 mmol, 98%) as a yellow-coloured solid.

^{1}H NMR (400 MHz, DMSO-d$_6$) δ 12.20 (br s, 1H), 8.89 (br s, 1H), 8.14 (br d, *J* = 7.4 Hz, 1H), 8.08 (br d, *J* = 8.1 Hz, 1H), 7.95 (br d, *J* = 7.4 Hz, 2H), 7.83 (br t, *J* = 7.3 Hz, 1H), 7.69 (br t, *J* = 7.5 Hz, 1H), 7.64 – 7.58 (m, 1H), 7.57 – 7.50 (m, 2H). Purity = 95%.

^{13}C NMR (101 MHz, DMSO-d$_6$) δ 163.3, 148.2, 142.9, 133.7, 133.0, 132.0, 130.6, 128.7, 128.5, 127.9, 127.7, 124.6.

LCMS: t_r = 0.96 min (HpH), ES+ (m/z) 270.00 ([M+H]$^+$, 100%), ES- (m/z) 268.25 ([M-H]$^-$, 100%), Purity = 100%.

FTIR: ν_{max} / cm^{-1} 3161w, 3005w, 2848w, 1644s, 1523s, 1343s.

HRMS: m/z Calcd. for $C_{14}H_{12}N_3O_3^+$ [M+H]$^+$ 270.0873, found 270.0892.

Spectroscopic data consistent with literature.[2]

(*E*)-*N*'-(4-Cyanobenzylidene)benzohydrazide (3h)

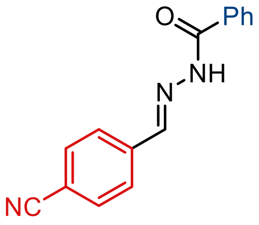

3h

To a 20 mL vial, 4-formylbenzonitrile (131 mg, 1.00 mmol, 1 equiv.), benzohydrazide (136 mg, 1.00 mmol, 1 equiv.), and MeOH (5 mL). The solvent was evaporated in a blowdown at 40 °C and further dried in a vacuum oven at 40 °C to afford (*E*)-*N*'-(4-cyanobenzylidene)benzohydrazide (243 mg, 0.98 mmol, 98%) as an off-white-coloured solid.

^{1}H NMR (400 MHz, DMSO-d$_6$) δ 12.07 (br s, 1H), 8.61 – 8.42 (m, 1H), 7.97 – 7.86 (m, 6H), 7.65 – 7.58 (m, 1H), 7.57 – 7.51 (m, 2H). Purity = 95%

^{13}C NMR (101 MHz, DMSO-d$_6$) δ 163.3, 145.8, 138.8, 133.1, 132.7, 131.9, 128.5, 127.6, 127.6, 118.6, 111.9.

LCMS: t_r = 0.84 min (HpH), ES+ (m/z) 250.07 ([M+H]$^+$, 100%), ES- (m/z) 248.19 ([M-H]$^-$, 100%), Purity = 98%

FTIR: ν_{max} / cm^{-1} 3235br,w, 3060w, 3029w, 2222w, 1659s, 1542s.

HRMS: m/z Calcd. For $C_{15}H_{12}N_3O^+$ [M+H]$^+$ 250.0975, found 250.0988.

Spectroscopic data consistent with literature.[7]

(*E*)-*N*'-((1*H*-Indol-7-yl)methylene)benzohydrazide (3k)

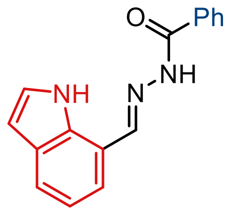

3k

The synthesis of **3k** was conducted following the general procedure P2 using 1*H*-indole-7-carbaldehyde (145 mg, 1.00 mmol, 1 equiv.), benzohydrazide (136 mg, 1.00 mmol, 1 equiv.), and MeOH (5 mL). The solvent was evaporated in a blowdown at 40 °C and further dried in a vacuum oven at 40 °C to afford (*E*)-*N*'-((1*H*-indol-7-yl)methylene)benzohydrazide (260 mg, 0.99 mmol, 99%) as a white-coloured solid.

^{1}H NMR (400 MHz, DMSO-d_6) δ 12.12 (s, 1H), 10.84 (br s, 1H), 8.70 (s, 1H), 8.00 (br d, *J* = 7.5 Hz, 2H), 7.72 (br d, *J* = 7.9 Hz, 1H), 7.66 – 7.51 (m, 4H), 7.38 (br d, *J* = 7.1 Hz, 1H), 7.15 (br t, *J* = 7.5 Hz, 1H), 6.59 (br s, 1H). Purity = 95%.

^{13}C NMR (101 MHz, DMSO-d_6) δ 163.1, 148.3, 133.1, 132.0, 131.9, 128.5, 128.2, 127.6, 126.3, 124.1, 122.9, 119.2, 117.8, 102.0.

LCMS: t_r = 1.06 min (HpH), ES+ (m/z) 264.05 ([M+H]$^+$, 100%), ES- (m/z) 262.23 ([M-H]$^-$, 100%), Purity = 100%.

FTIR: ν_{max} / cm^{-1} 3481w, 3393w, 3291w, 1646s.

HRMS: m/z Calcd. for $C_{16}H_{14}N_3O$ [M+H]$^+$ 264.1131, found 264.1147.

Spectroscopic data consistent with literature.[8]

(*E*)-*N*'-((4-Methylthiophen-2-yl)methylene)benzohydrazide (3l)

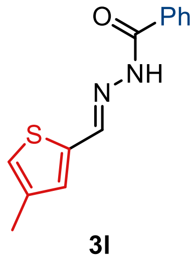

3l

The synthesis of **3l** was conducted following the general procedure P2 using 4-methylthiophene-2-carbaldehyde (123 µL, 126 mg, 1.00 mmol, 1 equiv.), benzohydrazide (136 mg, 1.00 mmol, 1 equiv.), and MeOH (5 mL). The reaction mixture was transferred to a round-bottom flask and concentrated *in vacuo*. The crude material was liquid loaded with a minimum amount of CH_2Cl_2 onto a 12 g RediSep® silica column, and purified by column chromatography (0 – 30% EtOAc:cyclohexane). The appropriate fractions were combined and concentrated *in vacuo* to afford (*E*)-*N*'-((4-methylthiophen-2-yl)methylene)benzohydrazide (123 mg, 0.50 mmol, 50%) as a brown-coloured solid.

^{1}H NMR (400 MHz, $CDCl_3$) δ 10.85 (br s, 1H), 8.76 (br s, 1H), 7.93 (br d, *J* = 6.9 Hz, 2H), 7.51 – 7.41 (m, 1H), 7.41 – 7.30 (m, 2H), 6.96 (s, 1H), 6.79 (s, 1H), 2.11 (s, 3H). Purity = 95%.

^{13}C NMR (101 MHz, $CDCl_3$) δ 164.9, 144.6, 138.7, 138.0, 133.4, 133.0, 132.0, 128.7, 127.7, 124.5, 15.6.

LCMS: t_r = 0.96 min (HpH), ES+ (m/z) 245.05 ($[M+H]^+$, 100%), ES- (m/z) 243.24 ($[M-H]^-$, 100%), Purity = 100%.

FTIR: ν_{max} / cm^{-1} 3214w, 3060w, 2924w, 1635s.

HRMS: m/z Calcd. for $C_{13}H_{13}N_2OS^+$ $[M+H]^+$ 245.0743, found 245.0755.

***tert*-Butyl (*E*)-3-((2-benzoylhydrazineylidene)methyl)-1*H*-pyrrolo[2,3-*b*]pyridine-1-carboxylate (3m)**

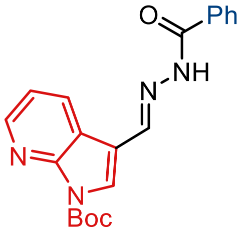

3m

The synthesis of **3m** was conducted following the general procedure P2 using *tert*-butyl 3-formyl-1*H*-pyrrolo[2,3-*b*]pyridine-1-carboxylate (246 mg, 1.00 mmol, 1 equiv.), benzohydrazide (136 mg, 1.00 mmol, 1 equiv.), and MeOH (5 mL). The reaction mixture was transferred to a round-bottom flask and concentrated *in vacuo*. The crude material was liquid loaded with a minimum amount of CH_2Cl_2 onto a 12 g RediSep® silica column, and purified by column chromatography (0 – 60% EtOAc:cyclohexane). The appropriate fractions were combined and concentrated *in vacuo* to afford *tert*-butyl (*E*)-3-((2-benzoylhydrazineylidene)methyl)-1*H*-pyrrolo[2,3-*b*]pyridine-1-carboxylate (334 mg, 0.92 mmol, 92%) as a pale yellow-coloured solid.

^{1}H NMR (400 MHz, DMSO-d_6) δ 11.93 (s, 1H), 8.74 (br d, *J* = 7.6 Hz, 1H), 8.59 (s, 1H), 8.53 – 8.44 (m, 1H), 8.28 (s, 1H), 7.94 (br d, *J* = 7.1 Hz, 2H), 7.63 – 7.57 (m, 1H), 7.57 – 7.50 (m, 2H), 7.43 (br dd, *J* = 7.8, 4.8 Hz, 1H), 1.63 (s, 9H). Purity = 95%.

^{13}C NMR (101 MHz, DMSO-d_6) δ 163.1, 147.9, 147.3, 145.7, 142.5, 133.5, 131.6, 131.2, 129.8, 128.4, 127.6, 119.6, 119.6, 114.2, 84.1, 27.7.

LCMS: t_r = 1.06 min (HpH), ES+ (m/z) 365.16 ($[M+H]^+$, 35%), ES- (m/z) 363.28 ($[M-H]^-$, 100%), Purity = 100%.

FTIR: ν_{max} / cm^{-1} 3233w, 3049w, 2979w, 1731m, 1648s.

HRMS: m/z Calcd. for $C_{20}H_{20}N_4O_3Na^+$ $[M+Na]^+$ 387.1428, found 387.1424.

(*E*)-*N*'-((1*H*-Benzo[*d*][1,2,3]triazol-5-yl)methylene) (3n)

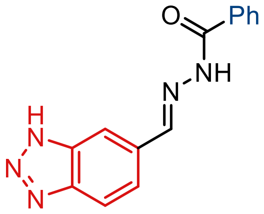

3n

The synthesis of **3n** was conducted following the general procedure P2 using 1*H*-benzo[*d*][1,2,3]triazole-5-carbaldehyde (147 mg, 1.00 mmol, 1 equiv.), benzohydrazide (136 mg, 1.00 mmol, 1 equiv.), and MeOH (5 mL). The reaction mixture was filtered, washed with CH_2Cl_2, dried under vacuum, and further dried in a vacuum oven at 40 °C to afford (*E*)-*N*'-((1*H*-benzo[*d*][1,2,3]triazol-5-yl)methylene)benzohydrazide (134 mg, 0.50 mmol, 50%) as a beige-coloured solid.

^{1}H NMR (600 MHz, MeOD-d$_4$) δ 8.52 (s, 1H), 8.23 (s, 1H), 8.17 (br d, *J* = 8.4 Hz, 1H), 7.96 (d, *J* = 7.3 Hz, 2H), 7.90 (br d, *J* = 8.8 Hz, 1H), 7.62 (t, *J* = 7.3 Hz, 1H), 7.56 – 7.52 (m, 2H). Purity = 95%. 2H not observed.

^{13}C NMR (151 MHz, MeOD-d$_4$) δ 167.2, 149.9, 134.1, 133.8, 133.4, 129.8, 128.8, 126.0 (br s), 111.4. 3C not observed.

LCMS: t_r = 0.58 min (HpH), ES+ (m/z) 266.04 ([M+H]$^+$, 100%), ES- (m/z) 264.47 ([M-H]$^-$, 100%), Purity = 100%.

FTIR: ν_{max} / cm^{-1} 3030w, 2847w, 1645s.

HRMS: m/z Calcd. for $C_{14}H_{12}N_5O^+$ [M+H]$^+$ 266.1036, found 266.1050.

(*E*)-*N*'-((4-Morpholino-2-phenylthieno[3,2-*d*]pyrimidin-6-yl)methylene)benzohydrazide (3o)

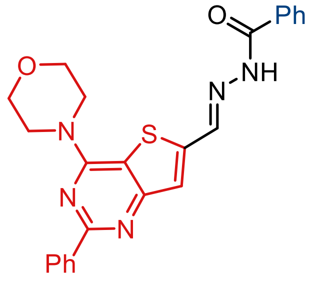

3o

The synthesis of **3o** was conducted following the general procedure P2 using 4-morpholino-2-phenylthieno[3,2-*d*]pyrimidine-6-carbaldehyde (326 mg, 1.00 mmol, 1 equiv.), benzohydrazide (136 mg, 1.00 mmol, 1 equiv.), and MeOH (5 mL). The reaction mixture was filtered, washed with MeOH, dried under vacuum, and further dried in a vacuum oven at 40 °C to afford (*E*)-*N*'-((4-morpholino-2-phenylthieno[3,2-*d*]pyrimidin-6-yl)methylene) benzohydrazide (385 mg, 0.87 mmol, 87%) as a yellow-coloured solid.

^{1}H NMR (400 MHz, DMSO-d$_6$) δ 12.17 (br s, 1H), 8.82 (br s, 1H), 8.40 (br d, *J* = 3.7 Hz, 2H), 7.99 – 7.80 (m, 3H), 7.67 – 7.58 (m, 1H), 7.58 – 7.45 (m, 5H), 4.02 (br s, 4H), 3.90 – 3.75 (m, 4H). Purity = 95%.

^{13}C NMR (101 MHz, DMSO-d$_6$) δ 163.2 (br s), 162.0, 159.4, 157.6, 144.7, 141.7 (br s), 137.8, 133.0, 132.0 (br s), 130.1, 128.5 (br s), 128.3, 127.7, 127.4 (br s), 112.6, 66.0, 46.0. 1C not observed.

LCMS: t_r = 1.23 min (HpH), ES+ (m/z) 444.16 ([M+H]$^+$, 100%), ES- (m/z) 442.29 ([M-H]$^-$, 80%), Purity = 100%.

FTIR: ν_{max} / cm^{-1} 3187w, 3065w, 2849w, 1645s.

HRMS: m/z Calcd. for $C_{24}H_{22}N_5O_2S^+$ [M+H]$^+$ 444.1489, found 444.1492.

(3-Chloro-4-cyano-2-methylphenyl)-*D*-threonine (3p')

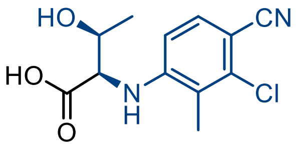

3p'

In a round-bottom flask, 2-chloro-4-fluoro-3-methylbenzonitrile (500 mg, 2.94 mmol, 1 equiv.), *D*-threonine (589 mg, 4.94 mmol, 1.7 equiv.), potassium carbonate (139 mg, 1 mmol, 0.3 equiv.), and DMSO (6 mL). The reaction mixture was stirred at 75 °C for 70 h. The reaction mixture was transferred to a 20 mL microwave vial. DIPEA (1 mL, 742 mg, 5.74 mmol, 2 equiv.), and DMSO (6 mL) were added to the round-bottom flask to wash any residual material into the microwave vial and the vial was crimp-sealed. The reaction mixture was stirred under microwave irradiation at 120 °C for 30 min, then at 140 °C for 3 h. DIPEA (0.5 mL, 371 mg, 2.87 mmol, 1 equiv.) was added to the reaction mixture which was stirred under microwave irradiation at 150 °C for 3 h. The reaction mixture was diluted with 10% aqueous citric acid solution (60 mL) and the mixture was extracted with EtOAc (60 mL). The aqueous layer was diluted with saturated aqueous ammonium chloride solution (60 mL) and the mixture was extracted with EtOAc (100 mL). The organic layers were combined, dried over anhydrous Na_2SO_4, filtered, and concentrated *in vacuo* to give an oil. The oil was dissolved in a minimum amount of CH_2Cl_2 and liquid loaded onto a 24 g RediSep® silica column and purified by column chromatography (0 – 100% EtOAc:cyclohexane). The appropriate fractions were combined and concentrated *in vacuo* to give an orange-coloured oil. The oil was further dried in a vacuum oven at 40 °C to afford (3-chloro-4-cyano-2-methylphenyl)-*D*-threonine (206 mg, 0.77 mmol, 26%) as an orange-coloured solid.

mp: 154 – 162 °C

^{1}H NMR (400 MHz, acetone-*d$_6$*) δ 7.48 (d, *J* = 8.7 Hz, 1H), 6.70 (d, *J* = 8.6 Hz, 1H), 5.35 (br d, *J* = 8.7 Hz, 1H), 4.48 (qd, *J* = 6.4, 3.0 Hz, 1H), 4.25 (dd, *J* = 8.7, 3.0 Hz, 1H), 2.34 (s, 3H), 1.34 (d, *J* = 6.4 Hz, 3H). Purity = 95%

^{13}C NMR (101 MHz, acetone-*d$_6$*) δ 172.6, 151.8, 136.2, 133.6, 121.9, 118.3, 109.7, 101.1, 68.3, 62.1, 21.0, 13.8.

LCMS: t_r = 0.60 min (HpH), ES+ (m/z) 268.99 ([M+H]$^+$, 65%), ES- (m/z) 267.17 ([M+H]$^-$, 100%), Purity = 97%.

FTIR: ν_{max} / cm^{-1} 3388m, 3089w, 2990w, 2923w, 2226m, 1723s, 1593vs.

HRMS: m/z Calcd. for $C_{12}H_{14}ClN_2O_3$ [M+H]$^+$ 269.0687, found 269.0704.

Spectroscopic data consistent with literature.[9]

2-((3-Chloro-4-cyano-2-methylphenyl)amino)-*N*'-(4-cyanobenzylidene)-3-hydroxybutanehydrazide (3p)

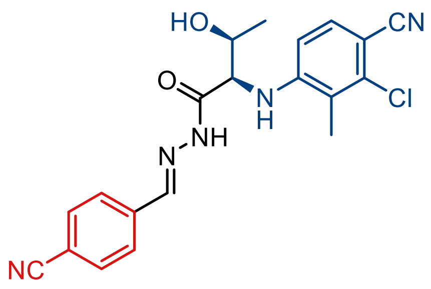

3p

To a 5 mL vial, (3-chloro-4-cyano-2-methylphenyl)-*D*-threonine (150 mg, 0.56 mmol, 1 equiv.), *tert*-butyl hydrazinecarboxylate (81.1 mg, 0.61 mmol, 1.1 equiv.), HATU (254 mg, 0.67 mmol, 1.2 equiv.), DIPEA (292 µL, 217 mg, 1.68 mmol, 3 equiv.), and anhydrous DMF (2 mL) were added. The reaction mixture was stirred for 14.5 h at room temperature. An additional portion of HATU (255 mg, 0.67 mmol, 1.2 equiv.) and *tert*-butyl hydrazinecarboxylate (20.8 mg, 0.16 mmol, 0.28 equiv.) were added and the reaction mixture was stirred for 1 h at room temperature.

HCl in dioxane (4 M, 2 mL, 292 mg, 8 mmol, 14 equiv.) was added and the reaction mixture was stirred for 2 h at room temperature. An additional portion of HCl in dioxane (4 M, 4 mL, 583 mg, 16 mmol, 29 equiv.) was added and the reaction mixture was stirred for 18 h at room temperature.

4-formylbenzonitrile (129 mg, 0.99 mmol, 1.8 equiv.) was added and the reaction mixture was stirred for 1 h at room temperature. The reaction mixture was neutralised with saturated aqueous $NaHCO_3$ solution (40 mL), transferred to a separating funnel, and extracted with EtOAc (50 mL × 2). The organic phases were combined, dried over anhydrous Na_2SO_4, filtered, and concentrated *in vacuo*.

The crude mixture was liquid loaded with a minimum amount of CH_2Cl_2 onto a 12 g RediSep® silica column and purified by column chromatography (0 – 100% EtOAc:cyclohexane). The product-containing fractions were combined and concentrated *in vacuo* to give an oil which was re-purified. The oil was dissolved in MeOH (0.5 mL) and purified by reverse phase prep HPLC (15 – 70% MeCN:10 mM aqueous ammonium bicarbonate). The appropriate fractions were combined and concentrated *in vacuo* to give a yellow oil. The oil was transferred into a 4 mL vial and further dried in a vacuum oven at 40 °C to afford a mixture of isomers of 2-((3-chloro-4-cyano-2-methylphenyl)amino)-*N*'-(4-cyanobenzylidene)-3-hydroxybutanehydrazide (101 mg, 0.26 mmol, 46%, 94% purity) as a yellow-coloured gum.

^{1}H NMR (400 MHz, DMSO-d_6) δ 11.85 (br s, 0.48H), 11.66 (br s, 0.52H), 8.33 (s, 0.52H), 8.08 (s, 0.48H), 7.91 (s, 1H), 7.91 – 7.83 (m, 3H), 7.57 (d, *J* = 8.6 Hz, 0.52H), 7.52 (d, *J* = 8.9 Hz, 0.48H), 6.55 (t, *J* = 9.0 Hz, 1H), 5.64 (t, *J* = 8.0 Hz, 1H), 5.45 (br s, 0.48H), 5.23 (d, *J* = 6.4 Hz, 0.48H), 4.96 (dd, *J* = 9.0, 3.8 Hz, 0.48H), 4.30 – 4.15 (m, 1H), 3.99 (dd, *J* = 7.8, 4.1 Hz, 0.52H), 2.33 (s, 1.56H), 2.30 (s, 1.44H), 1.25 (d, *J* = 6.4 Hz, 1.44H), 1.21 (d, *J* = 6.4 Hz, 1.56H). Purity = 94%. ^{1}H NMR data corresponds to multiple isomers.

^{1}H NMR (400 MHz, 120 °C, DMSO-d_6) δ 11.31 (br s, 1H), 8.30 (br s, 1H), 7.89 – 7.80 (m, 4H), 7.48 (d, *J* = 8.6 Hz, 1H), 6.64 (d, *J* = 8.8 Hz, 1H), 5.53 (br d, *J* = 8.1 Hz, 1H), 4.94 (br s, 1H), 4.30 – 4.20 (m, 1H), 2.33 (s, 3H), 1.26 (d, *J* = 6.4 Hz, 3H). 1H not observed.

^{13}C NMR (101 MHz, DMSO-d$_6$) δ 172.3, 168.1, 151.4, 150.9, 146.3, 142.5, 139.0, 138.9, 135.5, 133.5, 133.4, 133.2, 133.2, 128.1, 128.0, 121.5, 121.0, 119.1, 119.1, 118.4, 118.3, 112.5, 112.3, 109.1, 109.0, 99.3, 98.9, 67.2, 67.1, 63.1, 59.5, 21.4, 21.1, 14.3, 14.3. ^{13}C NMR data corresponds to multiple isomers.

LCMS: t_r = 1.03 (HpH), ES+ (m/z) 396.18 ([M+H]$^+$, 100%), ES- (m/z) 394.23 ([M+H]$^-$, 100%), Purity = 97%

FTIR: ν_{max} / cm^{-1} 3397br,w, 3209br,w, 3042w, 2974w, 2924w, 2226m, 1682m, 1592s.

HRMS: m/z Calcd. for $C_{20}H_{19}ClN_5O_2$ [M+H]$^+$ 396.1222, found 396.1227.

m/z Calcd. for $C_{20}H_{18}ClN_5NaO_2$ [M+Na]$^+$ 418.1041, found 418.1042.

***N*'-((1*E*,2*E*)-3-Phenylallylidene)benzohydrazide (3q)**

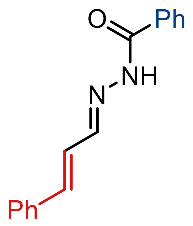

3q

The synthesis of **3q** was conducted following the general procedure P2 using cinnamaldehyde (126 µL, 132 mg, 1.00 mmol, 1 equiv.), benzohydrazide (136 mg, 1.00 mmol, 1 equiv.), and MeOH (5 mL). The solvent was evaporated in a blowdown at 40 °C and further dried in a vacuum oven at 40 °C to afford *N*'-((1*E*,2*E*)-3-phenylallylidene)benzohydrazide (236 mg, 0.94 mmol, 94%) as a white-coloured solid.

^{1}H NMR (400 MHz, DMSO-d$_6$) δ 11.74 (s, 1H), 8.25 (br d, *J* = 6.4 Hz, 1H), 7.90 (br d, *J* = 7.4 Hz, 2H), 7.63 (br d, *J* = 7.4 Hz, 2H), 7.58 (br d, *J* = 7.1 Hz, 1H), 7.55 – 7.48 (m, 2H), 7.43 – 7.37 (m, 2H), 7.36 – 7.29 (m, 1H), 7.14 – 7.01 (m, 2H). Purity = 95%.

^{13}C NMR (101 MHz, DMSO-d$_6$) δ 163.0, 149.7, 139.0, 135.9, 133.4, 131.7, 128.8, 128.4, 127.6, 127.1, 125.7. 1C not observed.

LCMS: t_r = 0.99 min (HpH), ES+ (m/z) 251.07 ([M+H]$^+$, 100%), ES- (m/z) 249.51 ([M-H]$^-$, 100%), Purity = 100%.

FTIR: ν_{max} / cm^{-1} 3261w, 3060w, 3033w, 1644s, 1623s.

HRMS: m/z Calcd. for $C_{16}H_{15}N_2O^+$ [M+H]$^+$ 251.1179, found 251.1194.

Spectroscopic data consistent with literature.[10]

***N*'-((3,4-Dihydro-2*H*-pyran-5-yl)methylene)benzohydrazide (3r)**

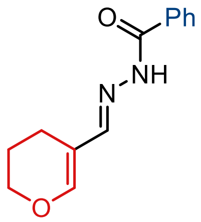

3r

The synthesis of **3r** was conducted following the general procedure P2 using 3,4-dihydro-2*H*-pyran-5-carbaldehyde (112 mg, 1.00 mmol, 1 equiv.), benzohydrazide (136 mg, 1.00 mmol, 1 equiv.), and MeOH (5 mL). The reaction mixture was filtered, washed with MeOH, dried under vacuum, and further dried in a vacuum oven at 40 °C to afford *N*'-((3,4-dihydro-2*H*-pyran-5-yl)methylene)benzohydrazide (96.4 mg, 0.39 mmol, 39%, 93% purity) as a pale yellow-coloured solid.

^{1}H NMR (400 MHz, $CDCl_3$) δ 9.08 (br s, 1H), 7.89 – 7.70 (m, *J* = 9.6 Hz, 3H), 7.50 (br t, *J* = 7.1 Hz, 1H), 7.46 – 7.39 (m, 2H), 6.82 (s, 1H), 4.10 (t, *J* = 5.6 Hz, 2H), 2.43 (t, *J* = 5.6 Hz, 2H), 1.91 (quin., *J* = 5.6 Hz, 2H). Purity = 93%.

^{13}C NMR (101 MHz, $CDCl_3$) δ 164.0, 153.0, 150.4, 133.9, 131.8, 128.8, 127.3, 113.1, 67.6, 21.3, 18.9.

LCMS: t_r = 0.78 min (HpH), ES+ (m/z) 231.09 ($[M+H]^+$, 100%), ES- (m/z) 229.36 ($[M-H]^-$, 50%), Purity = 100%.

FTIR: v_{max} / cm^{-1} 3222w, 2973w, 2850w, 1639m, 1618s.

HRMS: m/z Calcd. for $C_{13}H_{15}N_2O_2^+$ $[M+H]^+$ 231.1128, found 231.1144.

(*E*)-*N*'-(4-(Methylsulfonyl)benzylidene)benzohydrazide (3s)

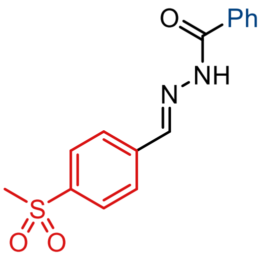

3s

The synthesis of **3s** was conducted following the general procedure P2 mg, 1.00 mmol, 1 equiv.), benzohydrazide (136 mg, 1.00 mmol, 1 equiv.), and MeOH (5 mL). The reaction mixture was filtered, washed with CH_2Cl_2, dried under vacuum, and further dried in a vacuum oven at 40 °C to afford (*E*)-*N*'-(4-(methylsulfonyl)benzylidene)benzohydrazide (274 mg, 0.91 mmol, 91%) as a white-coloured solid.

^{1}H NMR (400 MHz, DMSO-d_6) δ 12.08 (br s, 1H), 8.54 (br s, 1H), 8.00 (br s, 4H), 7.93 (br d, *J* = 7.1 Hz, 2H), 7.64 – 7.58 (m, 1H), 7.58 – 7.51 (m, 2H), 3.26 (s, 3H). Purity = 95%.

^{13}C NMR (101 MHz, DMSO-d_6) δ 163.3, 145.8), 141.4, 139.1, 133.1, 131.9, 128.5, 127.7, 127.5, 43.4. 1C not observed.

LCMS: t_r = 0.79 min (HpH), ES+ (m/z) 303.06 ($[M+H]^+$, 100%), ES- (m/z) 301.07 ($[M-H]^-$, 100%), Purity = 100%.

FTIR: v_{max} / cm^{-1} 3307m, 3019w, 2924w, 1668s.

HRMS: m/z Calcd. for $C_{15}H_{15}N_2O_3S^+$ $[M+H]^+$ 303.0798, found 303.0810.

Spectroscopic data consistent with literature.[11]

(*E*)-4-((2-Benzoylhydrazineylidene)methyl)phenyl acetate (3t)

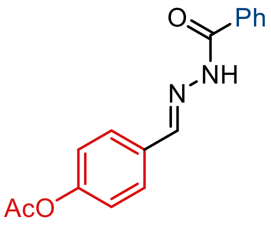

3t

The synthesis of **3t** was conducted following the general procedure P2 using 4-formylphenyl acetate (141 µL, 164 mg, 1.00 mmol, 1 equiv.), benzohydrazide (136 mg, 1.00 mmol, 1 equiv.), and MeOH (5 mL). The solvent was evaporated in a blowdown at 40 °C and further dried in a vacuum oven at 40 °C to afford (*E*)-4-((2-benzoylhydrazineylidene)methyl)phenyl acetate (223 mg, 0.79 mmol, 79%) as a white-coloured solid.

^{1}H NMR (400 MHz, DMSO-d$_6$) δ 11.74 (s, 1H), 8.25 (br d, *J* = 6.4 Hz, 1H), 7.90 (br d, *J* = 7.4 Hz, 2H), 7.63 (br d, *J* = 7.4 Hz, 2H), 7.58 (br d, *J* = 7.1 Hz, 1H), 7.55 – 7.48 (m, 2H), 7.43 – 7.37 (m, 2H), 7.36 – 7.29 (m, 1H), 7.14 – 7.01 (m, 2H). Purity = 95%.

^{13}C NMR (101 MHz, CDCl$_3$) δ 169.4, 164.6, 152.3, 147.9, 133.2, 132.2, 131.6, 129.0, 128.8, 127.6, 122.0, 21.2.

LCMS: t_r = 0.90 min (HpH), ES+ (m/z) 283.09 ([M+H]$^+$, 100%), ES- (m/z) 281.17 ([M-H]$^-$, 100%), Purity = 100%.

FTIR: ν_{max} / cm^{-1} 3263w, 3062w, 1758s, 1652s.

HRMS: m/z Calcd. for $C_{16}H_{15}N_2O_3^+$ [M+H]$^+$ 283.1077, found 283.1091.

(*E*)-*N*'-(3,5-Difluoro-4-(4,4,5,5-tetramethyl-1,3,2-dioxaborolan-2-yl)benzylidene)benzohydrazide (3u)

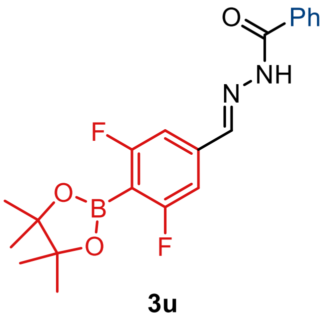

3u

The synthesis of **3u** was conducted following the general procedure P2 using 3,5-difluoro-4-(4,4,5,5-tetramethyl-1,3,2-dioxaborolan-2-yl)benzaldehyde (268 mg, 1.00 mmol, 1 equiv.), benzohydrazide (136 mg, 1.00 mmol, 1 equiv.), and MeOH (5 mL). The solvent was evaporated in a blowdown at 40 °C and further dried in a vacuum oven at 40 °C to afford (*E*)-*N*'-(3,5-difluoro-4-(4,4,5,5-tetramethyl-1,3,2-dioxaborolan-2-yl)benzylidene)benzohydrazide (384 mg, 0.99 mmol, 99%) as a white-coloured solid.

^{1}H NMR (400 MHz, DMSO-d$_6$) 12.12 (br s, 1H), 8.43 (br s, 1H), 7.92 (br d, *J* = 7.1 Hz, 2H), 7.66 – 7.58 (m, 1H), 7.57 – 7.50 (m, 2H), 7.40 (br d, *J* = 7.4 Hz, 2H), 1.32 (s, 12H). Purity = 95%.

^{13}C NMR (101 MHz, DMSO-d$_6$) δ 165.9 (dd, J_{CF} = 250.2, 13.7 Hz), 163.4, 144.8, 140.6 (br t, J_{CF} = 10.7 Hz), 133.1, 132.0, 128.5, 127.7, 109.4 (br d, J_{CF} = 27.5 Hz), 84.1, 24.5. 1C not observed.

^{19}F NMR (376 MHz, CDCl$_3$) δ -99.75 (s, 2F).

LCMS: t_r = 0.76 min (HpH), ES+ (m/z) 387.21 ([M+H]$^+$, 100%), ES- (m/z) 384.72 ([M-H]$^-$, 85%), % area = 17%. Boronic acid: t_r = 0.51 min (HpH), ES+ (m/z) 304.99 ([M+H]$^+$, 100%), ES- (m/z) 303.24 ([M-H]$^-$, 100%), % area = 72%. Protodeboronated product: t_r = 1.00 min (HpH), ES+ (m/z) 261.04 ([M+H]$^+$, 100%), ES- (m/z) 259.10 ([M-H]$^-$, 100%), % area = 11%.

FTIR: ν_{max} / cm^{-1} 3207w, 2978m, 2933w, 1630s.

HRMS: m/z Calcd. for $C_{14}H_{12}BF_2N_2O_3^+$ [M+H-C_6H_{12}]$^+$ 305.0904, found 305.0913. Mass corresponds to the boronic acid.

5. Synthesis and Characterisation of Oxadiazoles

2-(4-Fluorophenyl)-5-methyl-1,3,4-oxadiazole (2a)

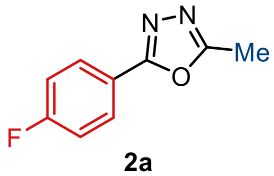

2a

The synthesis of **2a** was conducted following the general procedure P4, using *N*'-(4-fluorobenzylidene)acetohydrazide (45.1 mg, 0.25 mmol, 1 equiv.). The reaction mixture was concentrated *in vacuo,* liquid loaded with a minimum amount of CH_2Cl_2 onto a 12 g RediSep® silica column, and purified by column chromatography (0 – 40% EtOAc:cyclohexane). The appropriate fractions were combined and concentrated *in vacuo* to afford 2-(4-fluorophenyl)-5-methyl-1,3,4-oxadiazole (28.5 mg, 0.16 mmol, 64%) as an off-white-coloured solid.

^{1}H NMR (400 MHz, CDCl$_3$) δ 8.12 – 7.95 (m, 2H), 7.23 – 7.14 (m, 2H), 2.61 (s, 3H). Purity = 95%

^{13}C NMR (101 MHz, CDCl$_3$) δ 164.8 (d, J_{CF} = 253.3 Hz), 164.3, 163.8, 129.1 (d, J_{CF} = 9.2 Hz), 120.5 (d, J_{CF} = 3.1 Hz), 116.5 (d, J_{CF} = 21.4 Hz), 11.2.

^{19}F NMR (376 MHz, CDCl$_3$) δ -107.27 (s, 1F).

LCMS: t_r = 0.82 min (HpH), ES+ (m/z) 179.01 ([M+H]$^+$, 100%), Purity = 100%

FTIR: ν_{max} / cm^{-1} 3072w, 2932w, 1604s.

HRMS: m/z Calcd. for $C_9H_8FN_2O^+$ [M+H]$^+$ 179.0615, found 179.0626.

Spectroscopic data consistent with literature.[12,13]

<u>One-pot reaction:</u>

The synthesis of **2a** was conducted following the general procedure P5, using acetohydrazide (18.5 mg, 0.25 mmol, 1 equiv.), 4-fluorobenzaldehyde (26.8 µL, 31.0 mg, 0.25 mmol, 1 equiv.), and MeOH (3.6 mL). For the hydrazone formation step, the reaction mixture was stirred at 45 °C for 30 min. Prior to

electrolysis, electrolyte (1 equiv.) and DABCO (1 equiv.) were added and following electrolysis, the reaction mixture was concentrated *in vacuo*, liquid loaded with a minimum amount of CH_2Cl_2 onto a 12 g RediSep® silica column, and purified by column chromatography (0 – 50% EtOAc:cyclohexane). The appropriate column fractions were combined and concentrated *in vacuo* to give a white-coloured solid. The solid was further dried in a vacuum oven at 40 °C to afford 2-(4-fluorophenyl)-5-methyl-1,3,4-oxadiazole (18.7 mg, 10 mmol, 42%) as an off-white-coloured solid.

Scale-up (1 mmol):

To a 20 mL ElectraSyn 2.0 reaction vessel containing a stirrer bar, *N*'-(4-fluorobenzylidene)acetohydrazide (180 mg, 1 mmol, 1 equiv.), tetraethylammonium tetrafluoroborate (217 mg, 1 mmol, 1 equiv.), and DABCO (112 mg, 1 mmol, 1 equiv.) were added, followed by acetonitrile (14.4 mL). The reaction mixture was electrolysed under a constant current of 7 mA at room temperature with a graphite anode and platinum foil cathode, stirring at 400 rpm until a total charge of 3 F mol^{-1} had been passed. The electrodes were rinsed with methanol into the reaction mixture and the reaction mixture concentrated *in vacuo* to give a yellow solid. The crude material was liquid loaded with a minimum amount of CH_2Cl_2 onto a 12 g RediSep® silica column, and purified by column chromatography (0 – 40% EtOAc:cyclohexane). The appropriate column fractions were combined and concentrated *in vacuo* to give a white-coloured solid. The solid was further dried in a vacuum oven at 40 °C to afford 2-(4-fluorophenyl)-5-methyl-1,3,4-oxadiazole (105 mg, 0.56 mmol, 59%) as an off-white-coloured solid.

Scale-up (1 g):

To a 20 mL ElectraSyn 2.0 reaction vessel containing a stirrer bar, *N*'-(4-fluorobenzylidene)acetohydrazide (1.00 g, 5.55 mmol, 1 equiv.), tetraethylammonium tetrafluoroborate (1.21 g, 5.55 mmol, 1 equiv.), and DABCO (624 mg, 5.55 mmol, 1 equiv.) were added, followed by acetonitrile (16 mL). The reaction mixture was electrolysed under a constant current of 10.6 mA at room temperature with a graphite anode and platinum foil cathode, stirring at 800 rpm until a total charge of 4.5 F mol^{-1} had been passed. The electrodes were rinsed with methanol into a round-bottom flask and combined with the reaction mixture. The mixture was concentrated *in vacuo* to give a yellow solid. The crude material was liquid loaded with a minimum amount of CH_2Cl_2 onto a 12 g RediSep® silica column, and purified by column chromatography (0 – 50% EtOAc:cyclohexane). The appropriate column fractions were combined and concentrated *in vacuo* to give a yellow-coloured solid. The solid was further dried in a vacuum oven at 40 °C to afford 2-(4-fluorophenyl)-5-methyl-1,3,4-oxadiazole (401 mg, 1.9 mmol, 34%, 85% Purity) as a yellow-coloured solid.

2-Butyl-5-(4-fluorophenyl)-1,3,4-oxadiazole (2b)

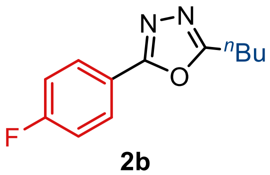

2b

The synthesis of **2b** was conducted following the general procedure P4, using *N*'-(4-fluorobenzylidene)pentanehydrazide (55.6 mg, 0.25 mmol, 1 equiv.). The reaction mixture was concentrated *in vacuo,* liquid loaded with a minimum amount of CH_2Cl_2 onto a 12 g RediSep® silica

column, and purified by column chromatography (0 – 30% EtOAc:cyclohexane). The appropriate fractions were combined and concentrated *in vacuo* to give a white-coloured solid. The solid was further dried in a vacuum oven at 40 °C to afford 2-butyl-5-(4-fluorophenyl)-1,3,4-oxadiazole (35.1 mg, 0.16 mmol, 64%) as a white-coloured, low melting point solid.

mp: 39 – 42 °C

^{1}H NMR (400 MHz, $CDCl_3$) δ 8.10 – 8.03 (m, 2H), 7.25 – 7.18 (m, 2H), 2.95 (t, *J* = 7.6 Hz, 2H), 1.91 – 1.80 (m, 2H), 1.49 (dq, *J* = 15.0, 7.5 Hz, 2H), 1.00 (t, *J* = 7.3 Hz, 3H). Purity = 95%

^{13}C NMR (101 MHz, $CDCl_3$) δ 167.2, 164.8 (d, J_{CF} = 253.3 Hz), 164.0, 129.2 (d, J_{CF} = 9.2 Hz), 120.6 (d, J_{CF} = 3.1 Hz), 116.5 (d, J_{CF} = 21.4 Hz), 28.8, 25.3, 22.3, 13.7.

^{19}F NMR (376 MHz, $CDCl_3$) δ -107.38 (s, 1F).

LCMS: t_r = 1.14 min (HpH), ES+ (m/z) 221.04 ($[M+H]^+$, 100%), Purity = 99%

FTIR: ν_{max} / cm^{-1} 3073w, 2979m, 2933w, 1604m.

HRMS: m/z Calcd. for $C_{12}H_{14}FN_2O^+$ $[M+H]^+$ 221.1085, found 221.1100.

2-(4-Fluorophenyl)-5-(2-methoxyethyl)-1,3,4-oxadiazole (2c)

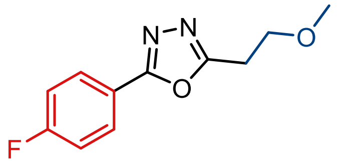

2c

The synthesis of **2c** was conducted following the general procedure P4, using *N*'-(4-fluorobenzylidene)-3-methoxypropanehydrazide (56.1 mg, 0.25 mmol, 1 equiv.). The reaction mixture was concentrated *in vacuo,* liquid loaded with a minimum amount of CH_2Cl_2 onto a 12 g RediSep® silica column, and purified by column chromatography (0 – 30% EtOAc:cyclohexane). The appropriate fractions were combined and concentrated *in vacuo* to give an oil. The oil was further dried in a vacuum oven at 40 °C to afford 2-(4-fluorophenyl)-5-(2-methoxyethyl)-1,3,4-oxadiazole (27.4 mg, 0.12 mmol, 49%) as a yellow-coloured oil.

^{1}H NMR (400 MHz, $CDCl_3$) δ 8.08 – 8.01 (m, 2H), 7.23 – 7.15 (m, 2H), 3.85 (t, *J* = 6.5 Hz, 2H), 3.40 (s, 3H), 3.20 (t, *J* = 6.5 Hz, 2H). Purity = 95%

^{13}C NMR (101 MHz, $CDCl_3$) δ 164.9, 164.9 (d, J_{CF} = 253.3 Hz), 164.3, 129.3 (d, J_{CF} = 9.2 Hz), 120.5 (d, J_{CF} = 4.6 Hz), 116.5 (d, J_{CF} = 22.9 Hz), 68.8, 59.0, 26.6.

^{19}F NMR (376 MHz, $CDCl_3$) δ -107.19 (s, 1F).

LCMS: t_r = 0.85 min (HpH), ES+ (m/z) 222.96 ($[M+H]^+$, 100%), Purity = 99%

FTIR: ν_{max} / cm^{-1} 3107w, 2923m, 1609m.

HRMS: m/z Calcd. for $C_{11}H_{12}FN_2O_2^+$ $[M+H]^+$ 223.0877, found 223.0892.

2-(4-Fluorophenyl)-1,3,4-oxadiazole (2d)

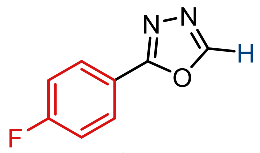

2d

The synthesis of **2d** was conducted following the general procedure P4, using *N*'-(4-fluorobenzylidene)formohydrazide (41.6 mg, 0.25 mmol, 1 equiv.). The reaction mixture was concentrated *in vacuo,* liquid loaded with a minimum amount of CH_2Cl_2 onto a 12 g RediSep® silica column, and purified by column chromatography (0 – 40% EtOAc:cyclohexane). The appropriate fractions were combined and concentrated *in vacuo* to give a white-coloured solid. The solid was further dried in a vacuum oven at 40 °C to afford 2-(4-fluorophenyl)-1,3,4-oxadiazole (13.1 mg, 0.80 mmol, 32%) as a white-coloured solid.

^{1}H NMR (400 MHz, $CDCl_3$) δ 8.45 (s, 1H), 8.13 – 8.06 (m, 2H), 7.25 – 7.19 (m, 2H). Purity = 95%

^{13}C NMR (101 MHz, $CDCl_3$) δ 166.4, 164.2, 152.7, 129.6 (d, J_{CF} = 9.5 Hz), 120.0 (d, J_{CF} = 2.9 Hz), 116.7 (d, J_{CF} = 22.7 Hz). Ipso J_{CF} coupling not observed.

^{19}F NMR (376 MHz, $CDCl_3$) δ -106.38 (s, 1F).

LCMS: t_r = 0.76 min (HpH), ES+ (m/z) 164.78 ($[M+H]^+$, 10%), Purity = 95%

FTIR: ν_{max} / cm^{-1} 3111w, 2919w, 1607s.

HRMS: m/z Calcd. for $C_8H_6FN_2O^+$ $[M+H]^+$ 165.0459, found 165.0472.

Spectroscopic data consistent with literature.[14]

1-((5-(4-Fluorophenyl)-1,3,4-oxadiazol-2-yl)methyl)azepan-2-one (2e)

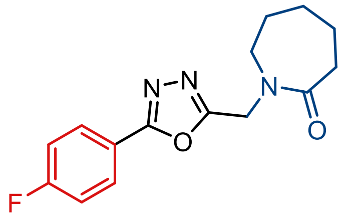

2e

The synthesis of **2e** was conducted following the general procedure P4, using *N*'-(4-fluorobenzylidene)-2-(2-oxoazepan-1-yl)acetohydrazide (72.9 mg, 0.25 mmol, 1 equiv.), acetonitrile (2.4 mL), and MeOH (1.2 mL). The reaction mixture was concentrated *in vacuo,* liquid loaded with a minimum amount of CH_2Cl_2 onto a 12 g RediSep® silica column, and purified by column chromatography (0 – 100% EtOAc:cyclohexane). The appropriate fractions were combined and concentrated *in vacuo* to give a colourless gum. The gum was further dried in a vacuum oven at 40 °C to afford 1-((5-(4-fluorophenyl)-1,3,4-oxadiazol-2-yl)methyl)azepan-2-one (55.1 mg, 0.19 mmol, 76 %) as a colourless gum.

^{1}H NMR (400 MHz, $CDCl_3$) δ 8.06 – 7.94 (m, 2H), 7.20 – 7.10 (m, 2H), 4.85 (s, 2H), 3.52 – 3.43 (m, 2H), 2.63 – 2.54 (m, 2H), 1.73 – 1.65 (m, 4H), 1.65 – 1.57 (m, 2H). Purity = 95%

^{13}C NMR (101 MHz, $CDCl_3$) δ 176.2, 164.7, 164.9 (d, J_{CF} = 253.3 Hz), 163.3, 129.3 (d, J_{CF} = 9.2 Hz), 120.0 (d, J_{CF} = 3.1 Hz), 116.5 (d, J_{CF} = 22.9 Hz), 49.7, 42.0, 36.9, 29.9, 28.1, 23.3.

^{19}F NMR (376 MHz, $CDCl_3$) δ -106.62 (s, 1F)

LCMS: t_r = 0.89 min (HpH), ES+ (m/z) 289.97 ($[M+H]^+$, 100%), Purity = 99%

FTIR: ν_{max} / cm^{-1} 3067w, 2933m, 1650s, 1611m.

HRMS: m/z Calcd. for $C_{15}H_{16}FN_3O_2Na^+$ $[M+Na]^+$ 312.1119, found 312.1127.

2-(2-(1*H*-Benzo[*d*]imidazol-1-yl)ethyl)-5-(4-fluorophenyl)-1,3,4-oxadiazole (2f)

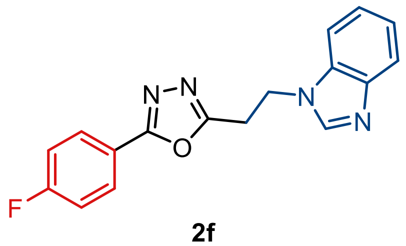

2f

The synthesis of **2f** was conducted following the general procedure P4, 3-(1*H*-benzo[*d*]imidazol-1-yl)-*N*'-(4-fluorobenzylidene)propanehydrazide (77.6 mg, 0.25 mmol, 1 equiv.), acetonitrile (1.2 mL), and MeOH (2.4 mL). The reaction mixture was concentrated *in vacuo,* liquid loaded with a minimum amount of CH_2Cl_2 onto a 12 g RediSep® silica column, and purified by column chromatography (0 – 100% EtOAc:cyclohexane). The appropriate fractions were combined and concentrated *in vacuo* to give a white-coloured solid. The solid was further dried in a vacuum oven at 40 °C to afford 2-(2-(1*H*-benzo[*d*]imidazol-1-yl)ethyl)-5-(4-fluorophenyl)-1,3,4-oxadiazole (48.7 mg, 0.16 mmol, 63%) as a white-coloured solid.

^{1}H NMR (400 MHz, $CDCl_3$) δ 8.03 (s, 1H), 7.92 – 7.86 (m, 2H), 7.85 – 7.80 (m, 1H), 7.44 – 7.38 (m, 1H), 7.34 – 7.27 (m, 2H), 7.20 – 7.13 (m, 2H), 4.77 (t, *J* = 6.7 Hz, 2H), 3.49 (t, *J* = 6.7 Hz, 2H). Purity = 95%

^{13}C NMR (101 MHz, $CDCl_3$) δ 164.5, 164.8 (d, J_{CF} = 254.8 Hz), 163.4, 143.8, 143.2, 133.3, 129.1 (d, J_{CF} = 9.2 Hz), 123.4, 122.6, 120.6, 119.7 (d, J_{CF} = 3.1 Hz), 116.4 (d, J_{CF} = 22.9 Hz), 109.2, 41.5, 26.5.

^{19}F NMR (376 MHz, $CDCl_3$) δ -106.47 (s, 1F).

LCMS: t_r = 0.91 min (HpH), ES+ (m/z) 308.93 ($[M+H]^+$, 100%), Purity = 93%.

FTIR: ν_{max} / cm^{-1} 3064w, 2939w, 1611m.

HRMS: m/z Calcd. for $C_{17}H_{14}FN_4O^+$ $[M+H]^+$ 309.1146, found 309.1157.

2-Cyclopropyl-5-(4-fluorophenyl)-1,3,4-oxadiazole (2g)

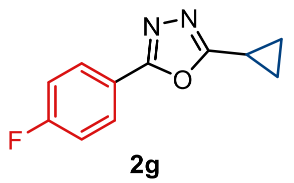

2g

The synthesis of **2g** was conducted following the general procedure P4, using *N*'-(4-fluorobenzylidene)cyclopropanecarbohydrazide (51.6 mg, 0.25 mmol, 1 equiv.). The reaction mixture

was concentrated *in vacuo,* liquid loaded with a minimum amount of CH_2Cl_2 onto a 12 g RediSep® silica column, and purified by column chromatography (0 – 40% EtOAc:cyclohexane). The appropriate fractions were combined and concentrated *in vacuo* to give a white-coloured solid. The solid was further dried in a vacuum oven at 40 °C to afford 2-cyclopropyl-5-(4-fluorophenyl)-1,3,4-oxadiazole (34.7 mg, 0.16 mmol, 63%, 93% purity) as a white-coloured solid.

mp: 79 – 82 °C

^{1}H NMR (400 MHz, $CDCl_3$) δ 8.05 – 7.96 (m, 2H), 7.22 – 7.13 (m, 2H), 2.26 – 2.17 (m, 1H), 1.22 – 1.17 (m, 4H). Purity = 93%

^{13}C NMR (101 MHz, $CDCl_3$) δ 168.6, 164.7 (d, J_{CF} = 251.8 Hz, 1C), 163.3, 129.0 (d, J_{CF} = 9.2 Hz), 120.6 (d, J_{CF} = 3.1 Hz), 116.4 (d, J_{CF} = 22.9 Hz), 8.6, 6.6.

^{19}F NMR (376 MHz, $CDCl_3$) δ -107.50 (s, 1F).

LCMS: t_r = 0.96 min (HpH), ES+ (m/z) 205.04 ($[M+H]^+$, 100%), Purity = 100%.

FTIR: ν_{max} / cm^{-1} 3046w, 2921m, 2851w, 1603s.

HRMS: m/z Calcd. for $C_{11}H_{10}FN_2O^+$ $[M+H]^+$ 205.0772, found 205.0785.

2-(4-Fluorophenyl)-5-((1*S*,2*S*)-2-phenylcyclopropyl)-1,3,4-oxadiazole (2h)

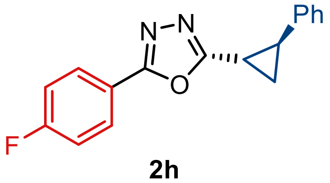

2h

The synthesis of **2h** was conducted following the general procedure P4, using (1*S*,2*S*)-*N*'-(4-fluorobenzylidene)-2-phenylcyclopropane-1-carbohydrazide (70.6 mg, 0.25 mmol, 1 equiv.), acetonitrile (1.2 mL) and MeOH (2.4 mL). The reaction mixture was concentrated *in vacuo,* liquid loaded with a minimum amount of CH_2Cl_2 onto a 12 g RediSep® silica column, and purified by column chromatography (0 – 30% EtOAc:cyclohexane). The appropriate fractions were combined and concentrated *in vacuo* to give a yellow-coloured solid. The solid was further dried in a vacuum oven at 40 °C to afford 2-(4-fluorophenyl)-5-((1*S*,2*S*)-2-phenylcyclopropyl)-1,3,4-oxadiazole (55.0 mg, 0.20 mmol, 78%) as a yellow-coloured solid.

^{1}H NMR (400 MHz, $CDCl_3$) δ 8.07 – 7.95 (m, 2H), 7.35 – 7.29 (m, 2H), 7.27 – 7.21 (m, 1H), 7.20 – 7.13 (m, 4H), 2.71 (ddd, *J* = 9.2, 6.4, 4.7 Hz, 1H), 2.46 (ddd, *J* = 8.8, 5.5, 4.7 Hz, 1H), 1.85 (dt, *J* = 9.2, 5.5 Hz, 1H), 1.65 (ddd, *J* = 8.8, 6.4, 5.2 Hz, 1H). Purity = 95%

^{13}C NMR (101 MHz, $CDCl_3$) δ 167.4, 164.7 (d, J_{CF} = 248.7 Hz), 139.5, 129.0 (d, J_{CF} = 9.2 Hz), 128.8, 126.9, 126.3, 120.5 (d, J_{CF} = 3.1 Hz), 116.4 (d, J_{CF} = 22.9 Hz), 26.9, 17.3, 17.1. 1C not observed.

^{19}F NMR (376 MHz, $CDCl_3$) δ -107.31 (s, 1F).

LCMS: t_r = 1.24 min (HpH), ES+ (m/z) 280.94 ($[M+H]^+$, 100%), Purity = 97%.

FTIR: ν_{max} / cm^{-1} 3030w, 2981w, 1609m.

HRMS: m/z Calcd. for $C_{17}H_{14}FN_2O^+$ $[M+H]^+$ 281.1085, found 281.1103.

tert-Butyl 3-(5-(4-fluorophenyl)-1,3,4-oxadiazol-2-yl)azetidine-1-carboxylate (2i)

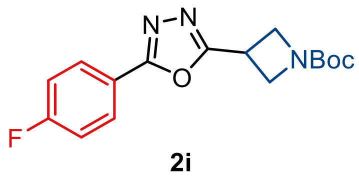

2i

The synthesis of **2i** was conducted following the general procedure P4, using *tert*-butyl 3-(2-(4-fluorobenzylidene)hydrazine-1-carbonyl)azetidine-1-carboxylate (80.4 mg, 0.25 mmol, 1 equiv.), acetonitrile (2.4 mL), and MeOH (1.2 mL). The reaction mixture was concentrated *in vacuo,* liquid loaded with a minimum amount of CH_2Cl_2 onto a 12 g RediSep® silica column, and purified by column chromatography (0 – 40% EtOAc:cyclohexane). The appropriate fractions were combined and concentrated *in vacuo* to give a white-coloured solid. The solid was further dried in a vacuum oven at 40 °C to afford *tert*-butyl 3-(5-(4-fluorophenyl)-1,3,4-oxadiazol-2-yl)azetidine-1-carboxylate (33.9 mg, 0.11 mmol, 42%) as a white-coloured solid.

^{1}H NMR (400 MHz, $CDCl_3$) δ 8.11 – 8.02 (m, 2H), 7.24 – 7.18 (m, 2H), 4.37 (t, *J* = 8.9 Hz, 2H), 4.30 (dd, *J* = 8.9, 6.2 Hz, 2H), 4.07 (tt, *J* = 8.9, 6.2 Hz, 1H), 1.47 (s, 9H). Purity = 95%

^{13}C NMR (101 MHz, $CDCl_3$) δ 166.3, 164.7, 165.0 (d, J_{CF} = 249.9 Hz), 156.1, 129.4 (d, J_{CF} = 9.2 Hz), 120.1 (d, J_{CF} = 4.6 Hz), 116.6 (d, J_{CF} = 22.9 Hz), 80.4, 53.1, 28.5, 25.1.

^{19}F NMR (376 MHz, $CDCl_3$) δ -106.47 (s, 1F).

LCMS: t_r = 1.11 min (HpH), ES+ (m/z) 319.92 ($[M+H]^+$, 10%), Purity = 100%.

FTIR: ν_{max} / cm^{-1} 3075w, 2920w, 1685s, 1606m.

HRMS: m/z Calcd. for $C_{16}H_{18}FN_3O_3Na^+$ $[M+Na]^+$ 342.1224, found 342.1230.

tert-Butyl (S)-2-(5-(4-fluorophenyl)-1,3,4-oxadiazol-2-yl)pyrrolidine-1-carboxylate (2j)

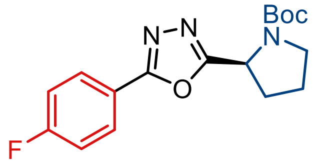

2j

The synthesis of **2j** was conducted following the general procedure P4, using *tert*-butyl (*S*)-2-(2-(4-fluorobenzylidene)hydrazine-1-carbonyl)pyrrolidine-1-carboxylate (83.9 mg, 0.25 mmol, 1 equiv.), acetonitrile (1.2 mL), MeOH (2.4 mL), and a stir rate of 1000 rpm. The reaction mixture was electrolysed until a total charge of 5 F mol^{-1} had been passed. The reaction mixture was concentrated *in vacuo,* liquid loaded with a minimum amount of CH_2Cl_2 onto a 12 g RediSep® silica column, and purified by column chromatography (0 – 50% EtOAc:cyclohexane). The appropriate fractions were combined and concentrated *in vacuo* to give a white-coloured solid. The solid was further dried in a vacuum oven at 40 °C to afford a mixture of rotamers of *tert*-butyl (*S*)-2-(5-(4-fluorophenyl)-1,3,4-oxadiazol-2-yl)pyrrolidine-1-carboxylate (65.9 mg, 20.0 mmol, 79%) as a white-coloured solid.

^{1}H NMR (400 MHz, DMSO-d$_6$) δ 8.10 – 7.98 (m, 2H), 7.45 (t, *J* = 8.9 Hz, 2H), 5.14 – 5.01 (m, 1H), 3.56 – 3.48 (m, 1H), 3.41 (dt, *J* = 10.3, 7.3 Hz, 1H), 2.43 – 2.25 (m, 1H), 2.18 – 1.88 (m, 3H), 1.39 (br s, 3.5H), 1.20 (s, 5.5H). Purity = 95%

^{13}C NMR (101 MHz, DMSO-d$_6$) δ 167.0 (d, J_{CF} = 235.0 Hz), 163.3, 153.2, 129.6 (d, J_{CF} = 9.2 Hz), 120.5 (br s), 117.2 (d, J_{CF} = 22.9 Hz), 79.8, 79.6 (br s), 52.8 (br s), 46.9 (br s), 46.7, 32.1 (br s), 31.1 (br s), 28.5 (br s), 28.3, 24.3 (br s), 23.7 (br s). ^{13}C NMR data corresponds to two rotamers. 9C not observed.

^{19}F NMR (376 MHz, DMSO-d$_6$) δ -107.41 (s, 1F), -107.51 (s, 0.58 F). ^{19}F NMR data corresponds to two rotamers.

LCMS: t_r = 1.14 min (HpH), ES+ (m/z) 334.01 ([M+H]$^+$, 20%), Purity = 100%

FTIR: ν_{max} / cm^{-1} 3069w, 2982w, 2874w, 1687s, 1615m.

HRMS: m/z Calcd. for $C_{17}H_{20}FN_3O_3Na^+$ [M+Na]$^+$ 356.1381, found 356.1387.

Benzyl (*S*)-(1-(5-(4-fluorophenyl)-1,3,4-oxadiazol-2-yl)-2-hydroxyethyl) carbamate (2k)

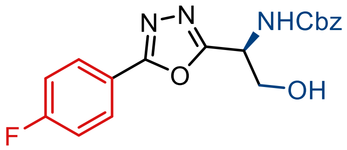

2k

The synthesis of **2k** was conducted following the general procedure P4, using benzyl (*S*)-(1-(2-(4-fluorobenzylidene)hydrazineyl)-3-hydroxy-1-oxopropan-2-yl)carbamate (89.8 mg, 0.25 mmol, 1 equiv.), acetonitrile (1.2 mL), and MeOH (2.4 mL). The reaction mixture was concentrated *in vacuo,* liquid loaded with a minimum amount of CH_2Cl_2 onto a 12 g RediSep® silica column, and purified by column chromatography (0 – 60% EtOAc:cyclohexane). The appropriate fractions were combined and concentrated *in vacuo* to give a yellow-coloured solid. The solid was further dried in a vacuum oven at 40 °C to afford benzyl (*S*)-(1-(5-(4-fluorophenyl)-1,3,4-oxadiazol-2-yl)-2-hydroxyethyl) carbamate (35.7 mg, 0.10 mmol, 40%) as a yellow-coloured solid.

^{1}H NMR (400 MHz, CDCl$_3$) δ 8.00 (br d, *J* = 5.6 Hz, 2H), 7.40 – 7.30 (m, 5H), 7.18 (t, *J* = 8.6 Hz, 2H), 5.90 – 5.79 (m, 1H), 5.26 – 5.14 (m, 3H), 4.32 – 4.22 (m, 1H), 4.04 (ddd, *J* = 11.7, 8.1, 4.2 Hz, 1H), 2.86 – 2.75 (m, 1H). Purity = 95%.

^{13}C NMR (101 MHz, CDCl$_3$) δ 165.2, 164.7, 165.0 (d, J_{CF} = 254.8 Hz), 156.2, 136.1, 129.4 (d, J_{CF} = 9.2 Hz), 128.7, 128.4, 128.3, 119.7 (d, J_{CF} = 3.1 Hz), 116.5 (d, J_{CF} = 22.9 Hz), 67.5, 63.0, 49.7.

^{19}F NMR (376 MHz, CDCl$_3$) δ -106.18 (s, 1F)

LCMS: t_r = 0.97 min (HpH), ES+ (m/z) 357.93 ([M+H]$^+$, 100%), Purity = 100%.

FTIR: ν_{max} / cm^{-1} 3308br,w, 3034w, 2950w, 1709s, 1611m.

HRMS: m/z Calcd. for $C_{18}H_{17}FN_3O_4^+$ [M+H]$^+$ 358.1198, found 358.1206.

2-(5-(4-Fluorophenyl)-1,3,4-oxadiazol-2-yl)propan-2-ol (2l)

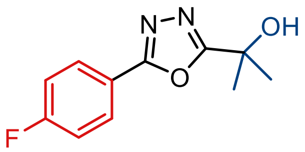

2l

The synthesis of **2l** was conducted following the general procedure P4, using *N*'-(4-fluorobenzylidene)-2-hydroxy-2-methylpropanehydrazide (56.1 mg, 0.25 mmol, 1 equiv.). The reaction mixture was concentrated *in vacuo,* liquid loaded with a minimum amount of CH_2Cl_2 onto a 12 g RediSep® silica column, and purified by column chromatography (0 – 40% EtOAc:cyclohexane). The appropriate fractions were combined and concentrated *in vacuo* to give a white-coloured solid. The solid was further dried in a vacuum oven at 40 °C to afford 2-(5-(4-fluorophenyl)-1,3,4-oxadiazol-2-yl)propan-2-ol (41.6 mg, 0.19 mmol, 75%) as a white-coloured solid.

^{1}H NMR (400 MHz, $CDCl_3$) δ 8.11 – 8.02 (m, 2H), 7.25 – 7.16 (m, 2H), 2.65 (s, 1H), 1.77 (s, 6H). Purity = 95%.

^{13}C NMR (101 MHz, $CDCl_3$) δ 170.5, 165.0 (d, J_{CF} = 253.1 Hz), 164.4, 129.4 (d, J_{CF} = 8.8 Hz), 120.3 (d, J_{CF} = 3.7 Hz), 116.6 (d, J_{CF} = 22.7 Hz), 68.6, 28.6.

^{19}F NMR (376 MHz, $CDCl_3$) δ -106.69 (s, 1F).

LCMS: t_r = 0.79 min (HpH), ES+ (m/z) 223.03 ([M+H]$^+$, 100%), Purity = 100%.

FTIR: ν_{max} / cm^{-1} 3340br,m, 3069w, 2991w, 1602m.

HRMS: m/z Calcd. for $C_{11}H_{12}FN_2O_2^+$ [M+H]$^+$ 223.0877, found 223.0890.

2-(*tert*-Butyl)-5-(4-fluorophenyl)-1,3,4-oxadiazole (2m)

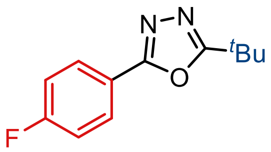

2m

The synthesis of **2m** was conducted following the general procedure P4, using *N*'-(4-fluorobenzylidene)pivalohydrazide (55.6 mg, 0.25 mmol, 1 equiv.). The reaction mixture was concentrated *in vacuo,* liquid loaded with a minimum amount of CH_2Cl_2 onto a 12 g RediSep® silica column, and purified by column chromatography (0 – 30% EtOAc:cyclohexane). The appropriate fractions were combined and concentrated *in vacuo* to give a white-coloured solid. The solid was further dried in a vacuum oven at 40 °C to afford 2-(*tert*-butyl)-5-(4-fluorophenyl)-1,3,4-oxadiazole (30.7 mg, 0.14 mmol, 56%) as a white-coloured solid.

mp: 78 – 81 °C

^{1}H NMR (400 MHz, $CDCl_3$) δ 8.09 – 8.00 (m, 2H), 7.23 – 7.15 (m, 2H), 1.49 (s, 9H). Purity = 95%.

^{13}C NMR (101 MHz, $CDCl_3$) δ 173.3, 164.8 (d, J_{CF} = 252.4 Hz), 164.0, 129.2 (d, J_{CF} = 8.8 Hz), 120.8 (d, J_{CF}= 3.7 Hz), 116.4 (d, J_{CF} = 22.0 Hz), 32.7, 28.4.

^{19}**F NMR** (376 MHz, $CDCl_3$) δ -107.49 (s, 1F).

LCMS: t_r = 1.11 min (HpH), ES+ (m/z) 221.04 ($[M+H]^+$, 100%), Purity = 100%.

FTIR: ν_{max} / cm^{-1} 3084w, 2976m, 1605m.

HRMS: m/z Calcd. for $C_{12}H_{14}FN_2O^+$ $[M+H]^+$ 221.1085, found 221.1090.

2-(4-Fluorophenyl)-5-phenyl-1,3,4-oxadiazole (2n)

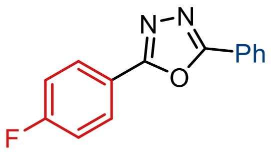

2n

The synthesis of **2n** was conducted following the general procedure P4, using *N*'-(4-fluorobenzylidene)benzohydrazide (60.6 mg, 0.25 mmol, 1 equiv.). The reaction mixture was concentrated *in vacuo,* liquid loaded with a minimum amount of CH_2Cl_2 onto a 12 g RediSep® silica column, and purified by column chromatography (0 – 40% EtOAc:cyclohexane). The appropriate fractions were combined and concentrated *in vacuo* to give a white-coloured solid. The solid was further dried in a vacuum oven at 40 °C to afford 2-(4-fluorophenyl)-5-phenyl-1,3,4-oxadiazole (28.3 mg, 0.12 mmol, 47%) as a white-coloured solid.

^{1}**H NMR** (400 MHz, $CDCl_3$) δ 8.20 – 8.09 (m, 4H), 7.60 – 7.50 (m, 3H), 7.27 – 7.20 (m, 2H). Purity = 95%.

^{13}**C NMR** (101 MHz, $CDCl_3$) δ 165.0 (d, J_{CF} = 253.3 Hz), 164.8, 163.9, 132.0, 129.4 (d, J_{CF} = 9.2 Hz), 129.3, 127.1, 124.0, 120.4 (d, J_{CF} = 4.6 Hz), 116.6 (d, J_{CF} = 21.4 Hz).

^{19}**F NMR** (376 MHz, $CDCl_3$) δ -106.85 (s, 1F).

LCMS: t_r = 1.17 min (HpH), ES+ (m/z) 241.03 ($[M+H]^+$, 100%), Purity = 100%.

FTIR: ν_{max} / cm^{-1} 3064w, 1606m.

HRMS: m/z Calcd. for $C_{14}H_{10}FN_2O^+$ $[M+H]^+$ 241.0772, found 241.0786.

Spectroscopic data consistent with literature.[13]

<u>One-pot reaction:</u>

The synthesis of **2n** was conducted following the general procedure P5, using benzohydrazide (34.1 mg, 0.25 mmol, 1 equiv.), 4-fluorobenzaldehyde (26.9 µL, 31.1 mg, 0.25 mmol, 1 equiv.), and MeOH (3.6 mL). For the hydrazone formation step, the reaction mixture was stirred at 45 °C for 30 min. Prior to electrolysis, electrolyte (1 equiv.) and DABCO (1 equiv.) were added, and the reaction mixture was electrolysed until a total charge of 3.5 F mol^{-1} had been passed. Following electrolysis, the reaction mixture was concentrated *in vacuo*, liquid loaded with a minimum amount of CH_2Cl_2 onto a 12 g RediSep® silica column, and purified by column chromatography (0 – 40% EtOAc:cyclohexane). The appropriate column fractions were combined and concentrated *in vacuo* to give a white-coloured solid. The solid was further dried in a vacuum oven at 40 °C to afford 2-(4-fluorophenyl)-5-phenyl-1,3,4-oxadiazole (36.6 mg, 0.15 mmol, 61%) as an off-white-coloured solid.

2-(4-Fluorophenyl)-5-(2-iodophenyl)-1,3,4-oxadiazole (2o)

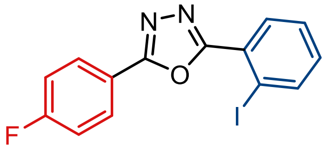

2o

The synthesis of **2o** was conducted following the general procedure P4, using *N*'-(4-fluorobenzylidene)-2-iodobenzohydrazide (92.1 mg, 0.25 mmol, 1 equiv.). The reaction mixture was concentrated *in vacuo,* liquid loaded with a minimum amount of CH_2Cl_2 onto a 12 g RediSep® silica column, and purified by column chromatography (0 – 40% EtOAc:cyclohexane). The appropriate fractions were combined and concentrated *in vacuo* to give a white-coloured solid. The solid was further dried in a vacuum oven at 40 °C to afford 2-(4-fluorophenyl)-5-(2-iodophenyl)-1,3,4-oxadiazole (58.2 mg, 0.15 mmol, 58%, 92% purity) as a white-coloured solid.

^{1}H NMR (400 MHz, $CDCl_3$) δ 8.23 – 8.18 (m, 2H), 8.11 (d, *J* = 7.9 Hz, 1H), 7.99 (dd, *J* = 7.9, 1.5 Hz, 1H), 7.60 – 7.51 (m, 1H), 7.30 – 7.22 (m, 3H). Purity = 92% (impurity corresponds to the des-iodide product **2n**).

^{13}C NMR (101 MHz, $CDCl_3$) δ 165.0 (d, J_{CF} = 253.3 Hz), 164.4, 164.3, 141.6, 132.6, 131.6, 129.5 (d, J_{CF} = 9.2 Hz), 128.4, 127.0, 120.2 (d, J_{CF} = 4.6 Hz), 116.6 (d, J_{CF} = 22.9 Hz), 94.0.

^{19}F NMR (376 MHz, $CDCl_3$) δ -106.49 (s, 1F). Purity = 93% (impurity at -106.83 (s, 0.11F) corresponds to the des-iodide product **2n**).

LCMS: t_r = 1.29 min (HpH), ES+ (m/z) 366.78 ([M+H]$^+$, 100%), Purity = 80%

Des-iodo side product **2n**: t_r = 1.17 min (HpH), ES+ (m/z) 240.95 ([M+H]$^+$, 100%), Area % = 20%

FTIR: ν_{max} / cm^{-1} 3066w, 1609m.

HRMS: m/z Calcd. for $C_{14}H_9FIN_2O^+$ [M+H]$^+$ 366.9738, found 366.9742.

2-(5-Bromo-2-chlorophenyl)-5-(4-fluorophenyl)-1,3,4-oxadiazole (2p)

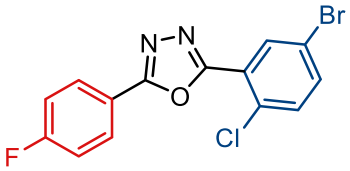

2p

The synthesis of **2p** was conducted following the general procedure P4, using 5-bromo-2-chloro-*N*'-(4-fluorobenzylidene)benzohydrazide (88.9 mg, 0.25 mmol, 1 equiv.), acetonitrile (1.2 mL), MeOH (2.4 mL), and a stir rate of 800 rpm. The reaction mixture was concentrated *in vacuo,* liquid loaded with a minimum amount of CH_2Cl_2 onto a 12 g RediSep® silica column, and purified by column chromatography (0 – 20% EtOAc:cyclohexane). The appropriate fractions were combined and concentrated *in vacuo* to give a white-coloured solid. The solid was further dried in a vacuum oven at 40 °C to afford 2-(5-bromo-2-chlorophenyl)-5-(4-fluorophenyl)-1,3,4-oxadiazole (53.0 mg, 0.15 mmol, 60%) as an off-white-coloured solid.

^{1}**H NMR** (400 MHz, $CDCl_3$) δ 8.25 (d, *J* = 2.5 Hz, 1H), 8.19 – 8.12 (m, 2H), 7.60 (dd, *J* = 8.6, 2.5 Hz, 1H), 7.45 (d, *J* = 8.6 Hz, 1H), 7.26 – 7.20 (m, 2H). Purity = 95%

^{13}**C NMR** (101 MHz, $CDCl_3$) δ 166.4, 163.9, 163.3 (d, J_{CF} = 268.6 Hz), 135.5, 133.7, 132.9, 132.1, 129.6 (d, J_{CF} = 7.6 Hz), 124.8, 120.9, 120.0 (d, J_{CF} = 3.1 Hz), 116.7 (d, J_{CF} = 22.9 Hz).

^{19}**F NMR** (376 MHz, $CDCl_3$) δ -106.11 (s, 1F).

LCMS: t_r = 1.37 min (HpH), ES+ (m/z) 352.74 ($[M+H]^+$, 100%), 354.71 ($[M+H]^+$, 100%), Purity = 98%

Des-chloro side product: t_r = 1.32 min (HpH), ES+ (m/z) 318.82 and 320.77 ($[M+H]^+$, 100%), Area % = 2%

FTIR: ν_{max} / cm^{-1} 3081w, 1610m.

HRMS: m/z Calcd. for $C_{14}H_8BrClFN_2O^+$ $[M+H]^+$ 352.9487, found 352.9497.

2-(4-Fluorophenyl)-5-(4-nitrophenyl)-1,3,4-oxadiazole (2q)

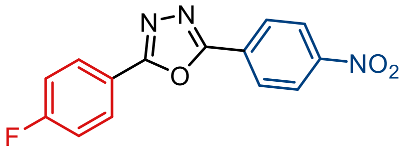

2q

The synthesis of **2q** was conducted following the general procedure P4, using *N*'-(4-fluorobenzylidene)-4-nitrobenzohydrazide (71.9 mg, 0.25 mmol, 1 equiv.) and a stir rate of 800 rpm. The reaction mixture was concentrated *in vacuo,* liquid loaded with a minimum amount of CH_2Cl_2 onto a 12 g RediSep® silica column, and purified by column chromatography (0 – 45% EtOAc:cyclohexane). The appropriate fractions were combined and concentrated *in vacuo* to give a yellow-coloured solid. The solid was further dried in a vacuum oven at 40 °C to afford 2-(4-fluorophenyl)-5-(4-nitrophenyl)-1,3,4-oxadiazole (34.9 mg, 0.12 mmol, 49 %) as a yellow-coloured solid.

^{1}**H NMR** (400 MHz, DMSO-d_6) δ 8.49 – 8.44 (m, 2H), 8.43 – 8.38 (m, 2H), 8.28 – 8.22 (m, 2H), 7.55 – 7.48 (m, 2H). Purity = 95%

^{13}**C NMR** (101 MHz, DMSO-d_6) δ 164.4 (d, J_{CF} = 253.3 Hz), 164.1, 162.8, 149.2, 129.7 (d, J_{CF} = 9.2 Hz), 128.9, 128.1, 124.6, 119.8 (d, J_{CF} = 3.1 Hz), 116.8 (d, J_{CF} = 22.9 Hz).

^{19}**F NMR** (376 MHz, DMSO-d_6) δ -106.73 (s, 1F)

LCMS: t_r = 1.18 min (HpH), ES+ (m/z) 285.88 ($[M+H]^+$, 50%), Purity = 98%

FTIR: ν_{max} / cm^{-1} 3108w, 1607m, 1494s, 1342s.

HRMS: m/z Calcd. for $C_{14}H_9FN_3O_3^+$ $[M+H]^+$ 286.0622, found 286.0630.

Spectroscopic data consistent with literature.[15]

3-(5-(4-Fluorophenyl)-1,3,4-oxadiazol-2-yl)-*N*,*N*-dimethylbenzenesulfonamide (2r)

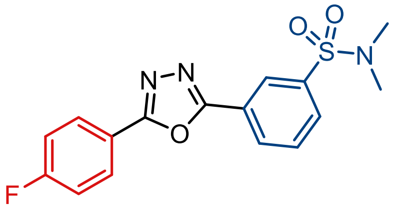

2r

The synthesis of **2r** was conducted following the general procedure P4, using 3-(2-(4-fluorobenzylidene)hydrazine-1-carbonyl)-*N*,*N*-dimethylbenzenesulfonamide (87.4 mg, 0.25 mmol, 1 equiv.), acetonitrile (1.2 mL), and MeOH (2.4 mL). The reaction mixture was concentrated *in vacuo,* liquid loaded with a minimum amount of CH_2Cl_2 onto a 12 g RediSep® silica column, and purified by column chromatography (0 – 100% EtOAc + 1% Et_3N:cyclohexane). The appropriate fractions were combined and concentrated *in vacuo* to give a white-coloured solid. The solid was further dried in a vacuum oven at 40 °C to afford 3-(5-(4-fluorophenyl)-1,3,4-oxadiazol-2-yl)-*N*,*N*-dimethylbenzenesulfonamide (43.8 mg, 0.13 mmol, 50%) as a white-coloured solid.

^{1}H NMR (400 MHz, $CDCl_3$) δ 8.49 (t, *J* = 1.5 Hz, 1H), 8.41 (dt, *J* = 7.8, 1.5 Hz, 1H), 8.22 – 8.15 (m, 2H), 7.99 – 7.95 (m, 1H), 7.76 (t, *J* = 7.8 Hz, 1H), 7.30 – 7.23 (m, 2H), 2.80 (s, 6H). Purity = 95%

^{13}C NMR (101 MHz, $CDCl_3$) δ 165.2 (d, J_{CF} = 254.8 Hz), 164.5, 163.4, 137.6, 130.9, 130.6, 130.2, 129.6 (d, J_{CF} = 9.2 Hz), 125.8, 125.2, 120.0 (d, J_{CF} = 4.6 Hz), 116.7 (d, J_{CF} = 21.4 Hz), 38.1.

^{19}F NMR (376 MHz, $CDCl_3$) δ -106.01 (s, 1F).

LCMS: t_r = 1.12 min (HpH), ES+ (m/z) 347.86 ([M+H]$^+$, 100%), Purity = 100%.

FTIR: ν_{max} / cm^{-1} 3075w, 2923w, 1609m.

HRMS: m/z Calcd. for $C_{16}H_{15}FN_3O_3S^+$ [M+H]$^+$ 348.0813, found 348.0819.

2-(4-Fluorophenyl)-5-(pyridin-4-yl)-1,3,4-oxadiazole (2s)

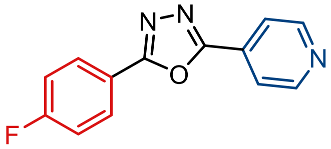

2s

The synthesis of **2s** was conducted following the general procedure P4, using *N*'-(4-fluorobenzylidene)isonicotinohydrazide (60.9 mg, 0.25 mmol, 1 equiv.), acetonitrile (2.4 mL), and MeOH (1.2 mL). The reaction mixture was concentrated *in vacuo,* liquid loaded with a minimum amount of CH_2Cl_2 onto a 12 g RediSep® silica column and purified by column chromatography (0 – 40% EtOAc:cyclohexane). The appropriate column fractions were combined and concentrated *in vacuo* to give a white-coloured solid. The solid was further dried in a vacuum oven at 40 °C to afford 2-(4-fluorophenyl)-5-(pyridin-4-yl)-1,3,4-oxadiazole (34.4 mg, 0.14 mmol, 57%) as an off-white-coloured solid.

^{1}H NMR (400 MHz, $CDCl_3$) δ 8.89 – 8.82 (m, 2H), 8.22 – 8.14 (m, 2H), 8.01 – 7.96 (m, 2H), 7.30 – 7.22 (m, 2H). Purity = 95%

^{13}C NMR (101 MHz, $CDCl_3$) δ 165.3 (d, J_{CF} = 253.3 Hz), 164.8, 163.0, 151.1, 131.1, 129.7 (d, J_{CF} = 9.2 Hz), 120.4, 119.9 (d, J_{CF} = 3.1 Hz), 116.8 (d, J_{CF} = 22.9 Hz).

^{19}F NMR (376 MHz, $CDCl_3$) δ -105.69 (s, 1F).

LCMS: t_r = 0.93 min (HpH), ES+ (m/z) 241.98 ([M+H]$^+$, 100%), Purity = 100%.

FTIR: ν_{max} / cm^{-1} 3056w, 1605m.

HRMS: m/z Calcd. for $C_{13}H_9FN_3O^+$ [M+H]$^+$ 242.0724, found 242.0730.

2-(4-Fluorophenyl)-5-(1*H*-indol-3-yl)-1,3,4-oxadiazole (2t)

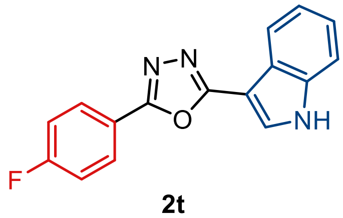

2t

The synthesis of **2t** was conducted following the general procedure P4, using *N*'-(4-fluorobenzylidene)-1*H*-indole-3-carbohydrazide (70.4 mg, 0.25 mmol, 1 equiv.), acetonitrile (2.4 mL), and MeOH (1.2 mL). The reaction mixture was concentrated *in vacuo,* liquid loaded with a minimum amount of CH_2Cl_2 onto a 12 g RediSep® silica column and purified by column chromatography (0 – 60% EtOAc:cyclohexane). The appropriate column fractions were combined and concentrated *in vacuo* to give a brown-coloured solid. The solid was further dried in a vacuum oven at 40 °C to afford 2-(4-fluorophenyl)-5-(1*H*-indol-3-yl)-1,3,4-oxadiazole (18.6 mg, 63 µmol, 25%, 95% purity) as beige-coloured solid.

^{1}H NMR (400 MHz, DMSO-d_6) δ 12.08 (br s, 1H), 8.31 (d, *J* = 2.5 Hz, 1H), 8.23 – 8.13 (m, 3H), 7.60 – 7.53 (m, 1H), 7.53 – 7.45 (m, 2H), 7.32 – 7.24 (m, 2H). Purity = 95%

^{13}C NMR (101 MHz, DMSO-d_6) δ 163.9 (d, J_{CF} = 250.2 Hz), 162.0, 161.1, 136.5, 129.0 (d, J_{CF} = 9.2 Hz), 128.6, 124.1, 122.9, 121.3, 120.4 (d, J_{CF} = 3.1 Hz), 120.2, 116.6 (d, J_{CF} = 21.4 Hz), 112.5, 99.3.

^{19}F NMR (376 MHz, DMSO-d_6) δ -108.16 (s, 1F).

LCMS: t_r = 1.12 min (HpH), ES+ (m/z) 280.03([M+H]$^+$, 100%), ES- (m/z) 278.24 ([M-H]$^-$, 100%), Purity = 100%.

FTIR: ν_{max} / cm^{-1} 3265br,m, 3134w, 3068w, 1602s.

HRMS: m/z Calcd. for $C_{16}H_{11}FN_3O^+$ [M+H]$^+$ 280.0881, found 280.0894.

2-(4-Fluorophenyl)-5-(1-methyl-1*H*-pyrazol-3-yl)-1,3,4-oxadiazole (2u)

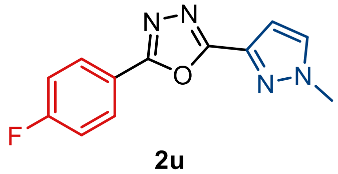

2u

The synthesis of **2u** was conducted following the general procedure P4, using *N*'-(4-fluorobenzylidene)-1-methyl-1*H*-pyrazole-3-carbohydrazide (59.4 mg, 88% purity, 0.21 mmol, 1 equiv.), tetraethylammonium tetrafluoroborate (46.6 mg, 0.21 mmol, 1 equiv.), DABCO (24.2 mg, 0.22 mmol, 1 equiv.), acetonitrile (1.2 mL), and MeOH (2.2 mL). The reaction mixture was concentrated *in vacuo,* liquid loaded with a minimum amount of CH_2Cl_2 onto a 12 g RediSep® silica column and purified by column chromatography (0 – 100% EtOAc + 1% Et_3N:cyclohexane). The appropriate column fractions were combined and concentrated *in vacuo* to give a white-coloured solid. The solid was further dried in a vacuum oven at 40 °C to afford 2-(4-fluorophenyl)-5-(1-methyl-1*H*-pyrazol-3-yl)-1,3,4-oxadiazole (30.0 mg, 0.12 mmol, 58%) as a white-coloured solid.

^{1}H NMR (400 MHz, $CDCl_3$) δ 8.27 – 8.10 (m, 2H), 7.50 (d, *J* = 2.4 Hz, 1H), 7.24 – 7.17 (m, 2H), 6.98 (d, *J* = 2.4 Hz, 1H), 4.17 – 3.92 (m, 3H). Purity = 95%

^{13}C NMR (101 MHz, $CDCl_3$) δ 164.9 (d, J_{CF} = 259.4 Hz), 160.4, 144.3, 137.5, 132.1, 129.6 (d, J_{CF} = 9.2 Hz), 120.3 (d, J_{CF} = 3.1 Hz), 116.5 (d, J_{CF} = 21.4 Hz), 107.2, 39.8.

^{19}F NMR (376 MHz, $CDCl_3$) δ -107.03 (s, 1F)

LCMS: t_r = 0.88 min (HpH), ES+ (m/z) 244.96 ($[M+H]^+$, 100%), Purity = 98%.

FTIR: $ν_{max}$ / cm^{-1} 3112w, 2950w, 1605s.

HRMS: m/z Calcd. for $C_{12}H_{10}FN_4O^+$ $[M+H]^+$ 245.0833, found 245.0848.

2-(3,5-Dimethoxyphenyl)-5-(4-fluorophenyl)-1,3,4-oxadiazole (2v)

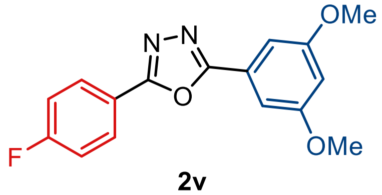

2v

The synthesis of **2v** was conducted following the general procedure P4, using *N*'-(4-fluorobenzylidene)-3,5-dimethoxybenzohydrazide (75.6 mg, 0.25 mmol, 1 equiv.), acetonitrile (2.4 mL), and MeOH (1.2 mL). The reaction mixture was concentrated *in vacuo,* liquid loaded with a minimum amount of CH_2Cl_2 onto a 12 g RediSep® silica column and purified by column chromatography (0 – 40% EtOAc:cyclohexane). The appropriate column fractions were combined and concentrated *in vacuo* to give a white-coloured solid. The solid was further dried in a vacuum oven at 40 °C to afford 2-(3,5-dimethoxyphenyl)-5-(4-fluorophenyl)-1,3,4-oxadiazole (61.9 mg, 0.21 mmol, 82%) as a white-coloured solid.

^{1}H NMR (400 MHz, $CDCl_3$) δ 8.19 – 8.09 (m, 2H), 7.28 – 7.18 (m, 4H), 6.63 (t, *J* = 2.2 Hz, 1H), 3.88 (s, 6H). Purity = 95%

^{13}C NMR (101 MHz, $CDCl_3$) δ 165.0 (d, J_{CF} = 253.3 Hz), 164.7, 163.9, 161.4, 129.4 (d, J_{CF} = 9.2 Hz), 125.4, 120.4 (d, J_{CF} = 4.6 Hz), 116.6 (d, J_{CF} = 22.9 Hz), 104.9, 104.4, 55.8.

^{19}F NMR (376 MHz, $CDCl_3$) δ -106.79 (s, 1F).

LCMS: t_r = 1.23 min (HpH), ES+ (m/z) 300.93 ($[M+H]^+$, 100%), Purity = 100%

FTIR: v_{max} / cm^{-1} 3052w, 2947w, 1593s.

HRMS: m/z Calcd. for $C_{16}H_{14}FN_2O_3^+$ $[M+H]^+$ 301.0983, found 301.0997.

2-(4-Fluorophenyl)-5-((methylsulfonyl)methyl)-1,3,4-oxadiazole 2w

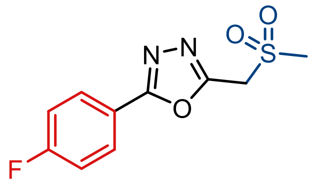

2w

The synthesis of **2w** was conducted following the general procedure P4, using *N*'-(4-fluorobenzylidene)-2-(methylsulfonyl)acetohydrazide (64.6 mg, 0.25 mmol, 1 equiv.), acetonitrile (1.2 mL), MeOH (2.4 mL), and a total charge of 4 F mol^{-1}. The reaction mixture was concentrated *in vacuo,* liquid loaded with a minimum amount of CH_2Cl_2 onto a 12 g RediSep® silica column and purified by column chromatography (0 – 100% EtOAc:cyclohexane). The appropriate column fractions were combined and concentrated *in vacuo* to give a brown-coloured solid. The solid was dissolved in a minimum amount of MeOH and purified by reverse phase prep HPLC (10 – 55% MeCN:10 mM aqueous ammonium bicarbonate solution). The appropriate fractions were combined and concentrated *in vacuo* to give a brown-coloured solid. The solid was further dried in a vacuum oven at 40 °C to afford 2-(4-fluorophenyl)-5-((methylsulfonyl)methyl)-1,3,4-oxadiazole (5.2 mg, 20 µmol, 8%) as a brown-coloured solid.

^{1}H NMR (400 MHz, $CDCl_3$) δ 8.13 – 8.05 (m, 2H), 7.25 – 7.18 (m, 2H), 4.62 (d, J = 0.7 Hz, 2H), 3.15 (s, 3H). Purity = 95%

^{13}C NMR (101 MHz, $CDCl_3$) δ 165.9, 165.4 (d, J_{CF} = 254.8 Hz), 157.3, 129.7 (d, J_{CF} = 9.2 Hz), 119.5 (d, J_{CF} = 3.1 Hz), 116.8 (d, J_{CF} = 22.9 Hz), 51.3, 40.6.

^{19}F NMR (376 MHz, $CDCl_3$) δ -105.41 (s, 1F).

LCMS: t_r = 0.75 min (HpH), ES+ (m/z) 256.90 ($[M+H]^+$, 100%), Purity = 100%

FTIR: v_{max} / cm^{-1} 3000m, 2927m, 1607s.

HRMS: m/z Calcd. for $C_{10}H_{10}FN_2O_3S^+$ $[M+H]^+$ 257.0391, found 257.0404.

4-(5-(4-Fluorophenyl)-1,3,4-oxadiazol-2-yl)morpholine (2x)

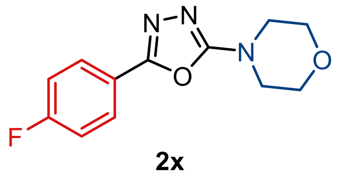

2x

The synthesis of **2x** was conducted following the general procedure P4, using *N*'-(4-fluorobenzylidene)morpholine-4-carbohydrazide (62.9 mg, 0.25 mmol, 1 equiv.). The reaction mixture was concentrated *in vacuo,* liquid loaded with a minimum amount of CH_2Cl_2 onto a 12 g RediSep® silica column and purified by column chromatography (40 – 100% EtOAc:cyclohexane). The appropriate column fractions were combined and concentrated *in vacuo* to give a white-coloured solid. The solid

was further dried in a vacuum oven at 40 °C to afford 4-(5-(4-fluorophenyl)-1,3,4-oxadiazol-2-yl)morpholine (44.8 mg, 0.18 mmol, 72%) as a white-coloured solid.

^{1}H NMR (400 MHz, $CDCl_3$) δ 7.95 – 7.87 (m, 2H), 7.18 – 7.11 (m, 2H), 3.86 – 3.80 (m, 4H), 3.60 – 3.56 (m, 4H). Purity = 95%

^{13}C NMR (101 MHz, $CDCl_3$) δ 164.3 (d, J_{CF} = 251.6 Hz), 164.2, 159.0, 128.1 (d, J_{CF} = 8.8 Hz), 121.0 (d, J_{CF} = 3.7 Hz), 116.3 (d_{CF}, J = 22.0 Hz), 66.1, 46.4.

^{19}F NMR (376 MHz, $CDCl_3$) δ -108.64 (s, 1F).

LCMS: t_r = 0.84 min (HpH), ES+ (m/z) 249.99 ($[M+H]^-$, 100%), Purity = 100%.

FTIR: ν_{max} / cm^{-1} 3068w, 2865w, 1608s.

HRMS: m/z Calcd. for $C_{12}H_{13}FN_3O_2^+$ $[M+H]^+$ 250.1001, found 250.0986.

Spectroscopic data consistent with literature.[13]

5-(4-Fluorophenyl)-1,3,4-thiadiazol-2-amine (2y)

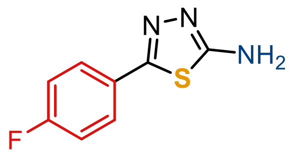

2y

The synthesis of **2y** was conducted following the general procedure P4, using 2-(4-fluorobenzylidene)hydrazine-1-carbothioamide (49.4 mg, 0.25 mmol, 1 equiv.). The reaction mixture was concentrated *in vacuo,* liquid loaded with a minimum amount of CH_2Cl_2 onto a 12 g RediSep® silica column and purified by column chromatography (0 – 100% EtOAc:cyclohexane). The appropriate column fractions were combined and concentrated *in vacuo* to give an off-white-coloured solid. The solid was further dried in a vacuum oven at 40 °C to afford 5-(4-fluorophenyl)-1,3,4-thiadiazol-2-amine (18.5 mg, 95 µmol, 38%) as an off-white-coloured solid.

^{1}H NMR (400 MHz, DMSO-d_6) δ 7.83 – 7.76 (m, 2H), 7.38 (s, 2H), 7.34 – 7.26 (m, 2H). Purity = 95%

^{13}C NMR (101 MHz, DMSO-d_6) δ 168.5, 162.6 (d, J_{CF} = 247.2 Hz), 155.2, 128.4 (d, J_{CF} = 7.6 Hz), 127.6 (d, J_{CF} = 3.1 Hz), 116.1 (d, J_{CF} = 22.9 Hz).

^{19}F NMR (376 MHz, DMSO-d_6) δ -111.47 (s, 1F).

LCMS: t_r = 0.67 min (HpH), ES+ (m/z) 195.91 ($[M+H]^+$, 100%), ES- (m/z) 194.05 ($[M-H]^-$, 100%), Purity = 98%.

FTIR: ν_{max} / cm^{-1} 3244w, 3084m, 2937w, 1613m.

HRMS: m/z Calcd. for $C_{12}H_{13}FN_3O_2^+$ $[M+H]^+$ 196.0339, found 196.0352.

Spectroscopic data consistent with literature.[16]

5-(4-Fluorophenyl)-1,3,4-oxadiazol-2(3*H*)-one (2z')

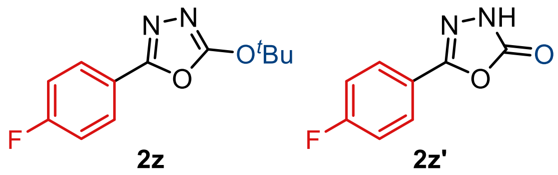

The synthesis of **2z'** was conducted following the general procedure P4, using 2-(4-fluorobenzylidene)hydrazine-1-carboxylate (59.6 mg, 0.25 mmol, 1 equiv.). Following the electrolysis, HCl in IPA (5–6 N, 100 µL, 18.2 mg, 0.5 mmol, 2 equiv.) was added slowly and the reaction mixture was stirred at room temperature for 1 h. The reaction mixture was concentrated *in vacuo,* liquid loaded with a minimum amount of CH_2Cl_2 onto a 12 g RediSep® silica column and purified by column chromatography (0 – 40% EtOAc:cyclohexane). The appropriate column fractions were combined and concentrated *in vacuo* to give a white-coloured solid. The solid was further dried in a vacuum oven at 40 °C to afford 5-(4-fluorophenyl)-1,3,4-oxadiazol-2(3*H*)-one (29.3 mg, 0.16 mmol, 65%) as a white-coloured solid.

^{1}H NMR (400 MHz, MeOD-d$_4$) δ 7.98 – 7.82 (m, 2H), 7.33 – 7.22 (m, 2H). 1H not observed. Purity = 95%

^{13}C NMR (101 MHz, MeOD-d$_4$) δ 166.0 (d, J_{CF} = 251.8 Hz), 156.9, 155.5, 129.1 (d, J_{CF} = 9.2 Hz), 122.1 (d, J_{CF} = 4.6 Hz), 117.3 (d, J_{CF} = 22.9 Hz).

^{19}F NMR (376 MHz, MeOD-d$_4$) δ -109.92 (s, 1F).

LCMS: t_r = 0.51 min (HpH), ES- (m/z) 178.92 ([M-H]$^-$, 30%), Purity = 95%

FTIR: $ν_{max}$ / cm^{-1} 3076m, 1841s, 1511s.

HRMS: No molecular ions corresponding to **2z'** were observed.

Spectroscopic data consistent with literature.[17]

***tert*-butyl 4-(5-Phenyl-1,3,4-oxadiazol-2-yl)piperidine-1-carboxylate (4a)**

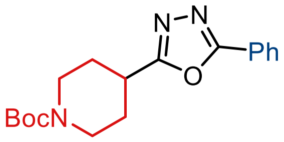

4a

The synthesis of **4a** was conducted following the general procedure P4, using *tert*-butyl 4-((2-benzoylhydrazineylidene)methyl)piperidine-1-carboxylate (92.6 mg, 0.28 mmol, 1.1 equiv.), tetraethylammonium tetrafluoroborate (54.4 mg, 0.25 mmol, 1 equiv.), and DABCO (28.1 mg, 0.25 mmol, 1 equiv.). The reaction mixture was concentrated *in vacuo*, liquid loaded with a minimum amount of CH_2Cl_2 onto a 12 g RediSep® silica column, and purified by column chromatography (0 – 50% EtOAc:cyclohexane). The appropriate column fractions were combined and concentrated *in vacuo* to give a white-coloured solid. The solid was further dried in a vacuum oven at 40 °C to afford *tert*-butyl 4-(5-phenyl-1,3,4-oxadiazol-2-yl)piperidine-1-carboxylate (51.3 mg, 0.16 mmol, 62%) as a white-coloured solid.

^{1}H NMR (400 MHz, $CDCl_3$) δ 8.12 – 7.95 (m, 2H), 7.60 – 7.41 (m, 3H), 4.22 – 4.03 (m, 2H), 3.16 (tt, *J* = 11.0, 3.8 Hz, 1H), 2.99 (br t, *J* = 11.8 Hz, 2H), 2.11 (br dd, *J* = 13.4, 3.0 Hz, 2H), 1.88 (dtd, *J* = 13.4, 11.0, 4.2 Hz, 2H), 1.47 (s, 9H). Purity = 95%

^{13}C NMR (101 MHz, $CDCl_3$) δ 168.5, 164.9, 154.8, 131.8, 129.2, 126.9, 124.1, 80.0, 43.2 (br s), 33.7, 29.2, 28.6.

LCMS: t_r = 1.20 min (HpH), ES+ (m/z) 330.28 ($[M+H]^+$, 10%), Purity = 100%.

FTIR: ν_{max} / cm^{-1} 3065w, 2982w, 2851w, 1683s.

HRMS: m/z Calcd. for $C_{18}H_{23}N_3ONa^+$ $[M+Na]^+$ 352.1632, found 352.1640.

2-Cyclopropyl-5-phenyl-1,3,4-oxadiazole (4b)

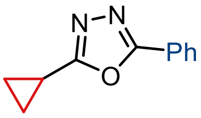

4b

The synthesis of **4b** was conducted following the general procedure P4, using *N*'-(cyclopropylmethylene)benzohydrazide (31.0 mg, 0.16 mmol, 0.66 equiv.), tetraethylammonium tetrafluoroborate (54.4 mg, 0.25 mmol, 1.5 equiv.), and DABCO (28.1 mg, 0.25 mmol, 1.5 equiv.). The reaction mixture was concentrated *in vacuo*, liquid loaded with a minimum amount of CH_2Cl_2 onto a 12 g RediSep® silica column, and purified by column chromatography (0 – 45% EtOAc:cyclohexane). The appropriate column fractions were combined and concentrated *in vacuo* to give an oil. The solid was further dried in a vacuum oven at 40 °C to afford 2-cyclopropyl-5-phenyl-1,3,4-oxadiazole (22.0 mg, 0.12 mmol, 72%) as a colourless oil.

^{1}H NMR (400 MHz, $CDCl_3$) δ 8.06 – 7.98 (m, 2H), 7.57 – 7.45 (m, 3H), 2.29 – 2.21 (m, 1H), 1.26 – 1.19 (m, 4H). Purity = 95%.

^{13}C NMR (101 MHz, $CDCl_3$) δ 168.5, 164.1, 131.5, 129.1, 126.8, 124.3, 8.6, 6.6.

LCMS: t_r = 0.96 min (HpH), ES+ (m/z) 187.18 ($[M+H]^+$, 100%), Purity = 100%.

FTIR: ν_{max} / cm^{-1} 3063w, 2926w, 1573s.

HRMS: m/z Calcd. for $C_{11}H_{11}N_2O^+$ $[M+H]^+$ 187.0866, found 187.0871.

Spectroscopic data consistent with literature.[18]

2-(1-(Benzo[*d*][1,3]dioxol-5-yl)propan-2-yl)-5-phenyl-1,3,4-oxadiazole (4c)

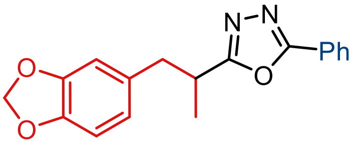

4c

The synthesis of **4c** was conducted following the general procedure P4, using *N*'-(3-(benzo[*d*][1,3]dioxol-5-yl)-2-methylpropylidene)benzohydrazide (77.6 mg, 0.25 mmol, 1 equiv.). The reaction mixture was concentrated *in vacuo*, liquid loaded with a minimum amount of CH_2Cl_2 onto a

12 g RediSep® silica column, and purified by column chromatography (0 – 40% EtOAc:cyclohexane). The appropriate column fractions were combined and concentrated *in vacuo* to give an oil. The solid was further dried in a vacuum oven at 35 °C to afford 2-(1-(benzo[*d*][1,3]dioxol-5-yl)propan-2-yl)-5-phenyl-1,3,4-oxadiazole (20.2 mg, 66 µmol, 26%) as a colourless gum.

^{1}H NMR (400 MHz, $CDCl_3$) δ 8.09 – 7.95 (m, 2H), 7.56 – 7.45 (m, 3H), 6.71 (d, *J* = 8.1 Hz, 1H), 6.67 (d, *J* = 1.5 Hz, 1H), 6.62 (dd, *J* = 7.8, 1.7 Hz, 1H), 5.91 (q, *J* = 1.5 Hz, 2H), 3.45 – 3.33 (m, 1H), 3.17 (dd, *J* = 13.7, 6.8 Hz, 1H), 2.86 (dd, *J* = 13.9, 8.1 Hz, 1H), 1.41 (d, *J* = 6.8 Hz, 3H). Purity = 95%.

^{13}C NMR (101 MHz, $CDCl_3$) δ 169.7, 164.7, 147.9, 146.4, 132.3, 131.6, 129.1, 126.9, 124.2, 122.2, 109.5, 108.4, 101.0, 40.7, 34.0, 17.8.

LCMS: t_r = 1.22 min (HpH), ES+ (m/z) 309.15 ($[M+H]^+$, 100%), Purity = 100%.

FTIR: ν_{max} / cm^{-1} 3067w, 2975w, 2882w.

HRMS: m/z Calcd. for $C_{18}H_{17}N_2O_3^+$ $[M+H]^+$ 309.1234, found 309.1243.

2,5-Diphenyl-1,3,4-oxadiazole (4d)

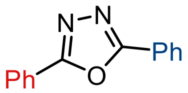

4d

The synthesis of **4d** was conducted following the general procedure P4, using *N*'-benzylidenebenzohydrazide (56.1 mg, 0.25 mmol, 1 equiv.). The reaction mixture was concentrated *in vacuo*, liquid loaded with a minimum amount of CH_2Cl_2 onto a 12 g RediSep® silica column, and purified by column chromatography (0 – 40% EtOAc:cyclohexane). The appropriate column fractions were combined and concentrated *in vacuo* to give a white-coloured solid. The solid was further dried in a vacuum oven at 40 °C to afford 2,5-diphenyl-1,3,4-oxadiazole (43.3 mg, 0.19 mmol, 78%) as a white-coloured solid.

^{1}H NMR (400 MHz, $CDCl_3$) δ 8.20 – 8.10 (m, 4H), 7.60 – 7.51 (m, 6H). Purity = 95%.

^{13}C NMR (101 MHz, $CDCl_3$) δ 164.8, 131.9, 129.2, 127.1, 124.1.

LCMS: t_r = 1.18 min (HpH), ES+ (m/z) 223.10 ($[M+H]^+$, 100%), Purity = 99%.

FTIR: ν_{max} / cm^{-1} 3058w, 1605w.

HRMS: m/z Calcd. for $C_{14}H_{11}N_2O^+$ $[M+H]^+$ 223.0866, found 223.0882.

Spectroscopic data consistent with literature.[19]

<u>One-pot reaction:</u>

The synthesis of **4d** was conducted following the general procedure P5, using benzohydrazide (34.1 mg, 0.25 mmol, 1 equiv.), benzaldehyde (25.5 µL, 26.6 mg, 0.25 mmol, 1 equiv.), and MeOH (3.6 mL). For the hydrazone formation step, the reaction mixture was stirred at 50 °C for 2.5 h. Prior to electrolysis, electrolyte (1 equiv.) and DABCO (1 equiv.) were added and following electrolysis, the reaction mixture was concentrated *in vacuo*, liquid loaded with a minimum amount of CH_2Cl_2 onto a 12 g RediSep® silica column, and purified by column chromatography (0 – 40% EtOAc:cyclohexane).

The appropriate column fractions were combined and concentrated *in vacuo* to give a white-coloured solid. The solid was further dried in a vacuum oven at 40 °C to afford 2,5-diphenyl-1,3,4-oxadiazole (35.3 mg, 0.16 mmol, 63%) as an off-white-coloured solid.

2-Phenyl-5-(*p*-tolyl)-1,3,4-oxadiazole (4e)

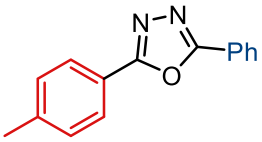

4e

The synthesis of **4e** was conducted following the general procedure P4, using *N*'-(4-methylbenzylidene)benzohydrazide (59.6 mg, 0.25 mmol, 1 equiv.). The reaction mixture was concentrated *in vacuo*, liquid loaded with a minimum amount of CH_2Cl_2 onto a 12 g RediSep® silica column, and purified by column chromatography (0 – 40% EtOAc:cyclohexane). The appropriate column fractions were combined and concentrated *in vacuo* to give a white-coloured solid. The solid was further dried in a vacuum oven at 40 °C to afford 2-phenyl-5-(*p*-tolyl)-1,3,4-oxadiazole (49.1 mg, 0.22 mmol, 83%) as an off-white-coloured solid.

^{1}H NMR (400 MHz, $CDCl_3$) δ 8.17 – 8.10 (m, 2H), 8.06 – 8.01 (m, 2H), 7.59 – 7.50 (m, 3H), 7.34 (d, *J* = 7.8 Hz, 2H), 2.45 (s, 3H). Purity = 95%

^{13}C NMR (101 MHz, $CDCl_3$) δ 164.9, 164.5, 146.4, 142.4, 131.7, 129.9, 129.2, 127.1, 124.2, 121.4, 21.8.

LCMS: t_r = 1.27 min (HpH), ES+ (m/z) 237.17 ($[M+H]^+$, 100%), Purity = 98%.

FTIR: ν_{max} / cm^{-1} 3062w, 2921w, 1611m.

HRMS: m/z Calcd. for $C_{15}H_{13}N_2O^+$ $[M+H]^+$ 237.1022, found 237.1037.

Spectroscopic data consistent with literature.[19]

<u>Synthesis of **4e** using Singh's methodology</u>[20]

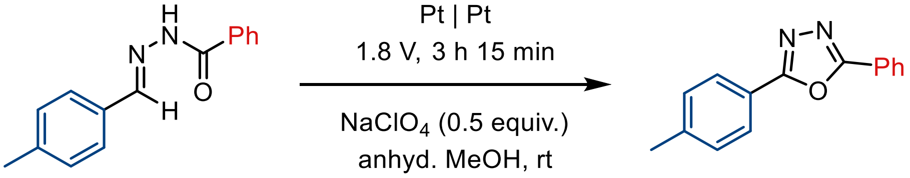

3e (0.42 mmol) **4e**, 14%

To a 5 mL ElectraSyn 2.0 reaction vessel containing a stirrer bar, *N*'-(4-methylbenzylidene)benzohydrazide (100 mg, 0.42 mmol, 1 equiv.) and sodium perchlorate (25.7 mg, 0.21 mmol, 0.5 equiv.) were added followed by anhydrous MeOH (3.5 mL). The reference electrode was filled with 3 M KCl (aq) and allowed to sit in a 3 M KCl (aq) solution for 30 min before conducting the electrolysis. The reaction mixture was electrolysed under a constant potential of 1.8 V mA at room temperature with platinum foil electrodes, stirring at 400 rpm for 195 min (3 h 15 min). The electrodes were rinsed with methanol into the reaction mixture. The crude material was liquid loaded with a minimum amount of CH_2Cl_2 onto a 12 g RediSep silica column and purified by silica column chromatography (0 – 40% EtOAc:cyclohexane) over 16 min. The appropriate column fractions were

combined and concentrated *in vacuo* to afford 2-phenyl-5-(*p*-tolyl)-1,3,4-oxadiazole (22.6 mg, 0.06 mmol, 14%, 60% purity by NMR) as an orange-coloured solid and the starting material *N*'-(4-methylbenzylidene)benzohydrazide (41.3 mg, 0.17 mmol, 41%) was recovered as a white-coloured solid.

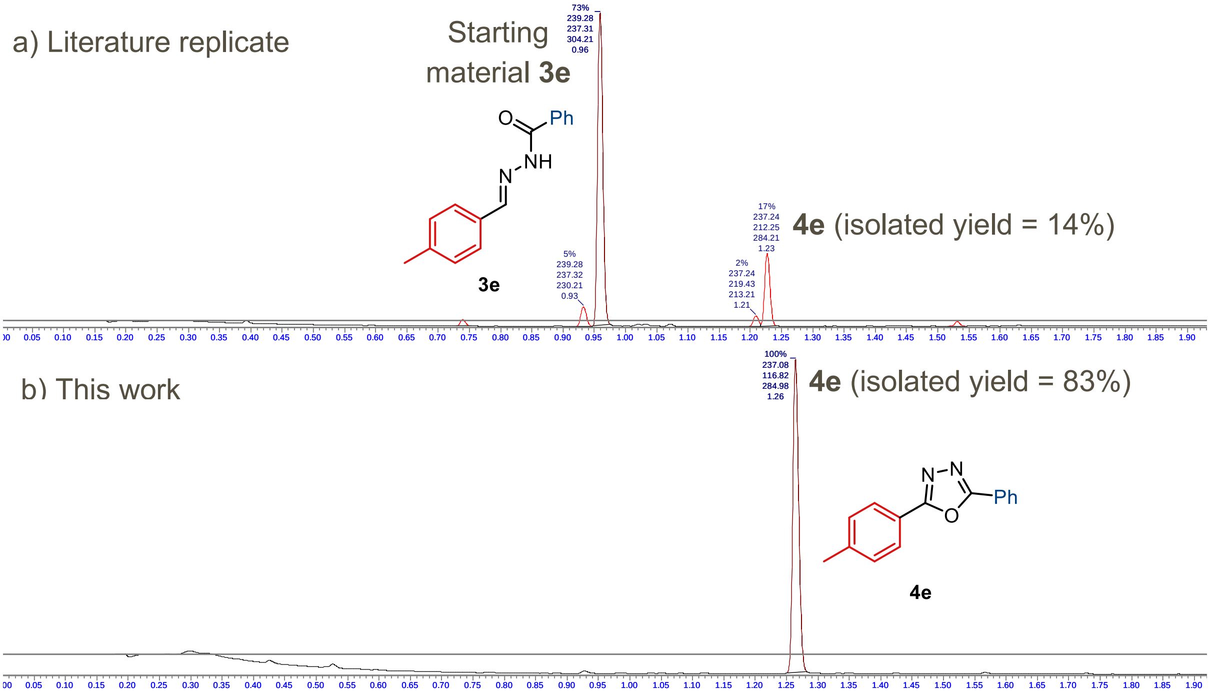

Figure S1. LCMS spectra of the reaction mixtures for a) the replicate of Singh's methodology[20] and b) this work.

2-([1,1'-Biphenyl]-4-yl)-5-methyl-1,3,4-oxadiazole (4f)

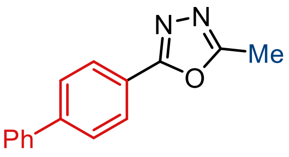

4f

The synthesis of **4f** was conducted following the general procedure P4, a 10 mL ElectraSyn 2.0 reaction vessel, tetraethylammonium tetrafluoroborate (108.6 mg, 0.5 mmol, 1 equiv.), *N*'-([1,1'-biphenyl]-4-ylmethylene)acetohydrazide (119.2 mg, 0.5 mmol, 1 equiv.), DABCO (56.2 mg, 0.5 mmol, 1 equiv.), HFIP (68.5 µL, 109.3 mg, 0.65 mmol, 1.3 equiv.), and acetonitrile (7 mL). The reaction mixture was concentrated *in vacuo*, liquid loaded with a minimum amount of CH_2Cl_2 onto a 12 g RediSep® silica column, and purified by column chromatography (0 – 60% EtOAc:cyclohexane). The appropriate column fractions were combined and concentrated *in vacuo* to give a white-coloured solid. The solid was further dried in a vacuum oven at 40 °C to afford 2-([1,1'-biphenyl]-4-yl)-5-methyl-1,3,4-oxadiazole (66.6 mg, 0.28 mmol, 56%) as an off-white-coloured solid.

^{1}H NMR (400 MHz, $CDCl_3$) δ 8.17 – 8.08 (m, 2H), 7.80 – 7.71 (m, 2H), 7.69 – 7.63 (m, 2H), 7.54 – 7.46 (m, 2H), 7.46 – 7.40 (m, 1H), 2.66 (s, 3H). Purity = 95%

^{13}C NMR (101 MHz, $CDCl_3$) δ 164.9, 163.8, 144.4, 140.0, 129.1, 128.3, 127.8, 127.3, 127.3, 122.9, 11.3.

LCMS: t_r = 1.13 min (HpH), ES+ (m/z) 237.10 ([M+H]$^+$, 100%), Purity = 100%

FTIR: v_{max} / cm^{-1} 3059w, 3033w, 2929w.

HRMS: m/z Calcd. for $C_{15}H_{13}N_2O^+$ [M+H]$^+$ 237.1022, found 237.1027.

2-(2-Nitrophenyl)-5-phenyl-1,3,4-oxadiazole (4g)

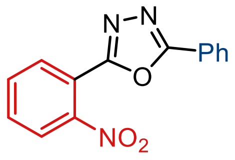

4g

The synthesis of **4g** was conducted following the general procedure P4, using *N*'-(2-nitrobenzylidene)benzohydrazide (67.4 mg, 0.25 mmol, 1 equiv.). The reaction mixture was concentrated *in vacuo*, liquid loaded with a minimum amount of CH_2Cl_2 onto a 12 g RediSep® silica column, and purified by column chromatography (0 – 40% EtOAc:cyclohexane). The appropriate column fractions were combined and concentrated *in vacuo* to give a yellow-coloured solid. The solid was further dried in a vacuum oven at 40 °C to afford 2-(2-nitrophenyl)-5-phenyl-1,3,4-oxadiazole (35.9 mg, 0.13 mmol 54%) as a yellow-coloured solid.

^{1}H NMR (400 MHz, $CDCl_3$) δ 8.12 – 8.01 (m, 4H), 7.83 – 7.72 (m, 2H), 7.60 – 7.49 (m, 3H). Purity = 95%

^{13}C NMR (101 MHz, $CDCl_3$) δ 165.9, 161.4, 148.4, 133.2, 132.6, 132.3, 131.8, 129.3, 127.3, 124.8, 123.5, 118.8.

LCMS: t_r = 1.13 min (HpH), ES+ (m/z) 268.09 ([M+H]$^+$, 100%), Purity = 100%.

FTIR: v_{max} / cm^{-1} 3062w, 2921w, 1610m.

HRMS: m/z Calcd. for $C_{14}H_{10}N_3O_3^+$ [M+H]$^+$ 268.0717, found 268.0732.

m/z Calcd. for $C_{14}H_9N_3O_3Na^+$ [M+Na]$^+$ 290.0536, found 290.0547.

Spectroscopic data consistent with literature.[10]

4-(5-Phenyl-1,3,4-oxadiazol-2-yl)benzonitrile (4h)

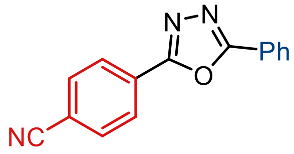

4h

The synthesis of **4h** was conducted following the general procedure P4, using *N*'-(4-cyanobenzylidene)benzohydrazide (62.4 mg, 0.25 mmol, 1 equiv.). The reaction mixture was concentrated *in vacuo*, liquid loaded with a minimum amount of CH_2Cl_2 onto a 12 g RediSep® silica column, and purified by column chromatography (0 – 40% EtOAc:cyclohexane). The appropriate column fractions were combined and concentrated *in vacuo* to give a white-coloured solid. The solid was further dried in a vacuum oven at 40 °C to afford 4-(5-phenyl-1,3,4-oxadiazol-2-yl)benzonitrile (38.8 mg, 0.16 mmol, 63%) as a white-coloured solid.

¹H NMR (400 MHz, $CDCl_3$) δ 8.30 – 8.23 (m, 2H), 8.18 – 8.13 (m, 2H), 7.87 – 7.81 (m, 2H), 7.63 – 7.53 (m, 3H). Purity = 95%

¹³C NMR (101 MHz, $CDCl_3$) δ 165.5, 163.2, 133.0, 132.4, 129.4, 128.0, 127.5, 127.3, 123.6, 118.0, 115.4.

LCMS: t_r = 1.13 min (HpH), ES+ (m/z) 248.02 ($[M+H]^+$, 100%), Purity = 100%

FTIR: ν_{max} / cm^{-1} 3056w, 2231m.

HRMS: m/z Calcd. for $C_{15}H_{10}N_3O^+$ $[M+H]^+$ 248.0818, found 248.0830.

Spectroscopic data consistent with literature.[21]

2-(5-Phenyl-1,3,4-oxadiazol-2-yl)phenol (4i)

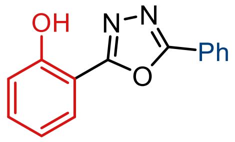

4i

The synthesis of **4i** was conducted following the general procedure P4, using *N*'-(2-hydroxybenzylidene)benzohydrazide (60.1 mg, 0.25 mmol, 1 equiv.). The reaction mixture was concentrated *in vacuo*, liquid loaded with a minimum amount of CH_2Cl_2 onto a 12 g RediSep® silica column, and purified by column chromatography (0 – 40% EtOAc:cyclohexane). The appropriate column fractions were combined and concentrated *in vacuo* to give a white-coloured solid. The solid was further dried in a vacuum oven at 40 °C to afford 2-(5-phenyl-1,3,4-oxadiazol-2-yl)phenol (23.2 mg, 97 µmol, 39%) as a white-coloured solid.

¹H NMR (400 MHz, $CDCl_3$) δ 10.19 (s, 1H), 8.19 – 8.12 (m, 2H), 7.88 (dd, *J* = 7.9, 2.0 Hz, 1H), 7.63 – 7.53 (m, 3H), 7.47 (ddd, *J* = 8.5, 7.1, 2.0 Hz, 1H), 7.16 (dd, *J* = 8.5, 1.0 Hz, 1H), 7.08 – 7.02 (m, 1H). Purity = 95%

¹³C NMR (101 MHz, $CDCl_3$) δ 164.4, 163.4, 157.9, 133.8, 132.3, 129.4, 127.2, 126.7, 123.5, 120.1, 117.8, 108.3.

LCMS: t_r = 1.23 min (HpH), ES+ (m/z) 238.99 ($[M+H]^+$, 100%), ES- (m/z) 237.14 ($[M+H]^-$, 100%), Purity = 100%.

FTIR: ν_{max} / cm^{-1} 3167m, 3063w.

HRMS: m/z Calcd. for $C_{14}H_{11}N_2O_2^+$ $[M+H]^+$ 239.0815, found 239.0828.

Spectroscopic data consistent with literature.[22]

2-(5-(Pyridin-4-yl)-1,3,4-oxadiazol-2-yl)phenol (4j)

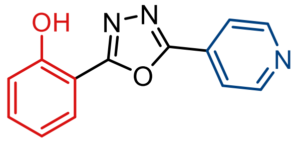

4j

The synthesis of **4j** was conducted following the general procedure P4, using *N*'-(2-hydroxybenzylidene)isonicotinohydrazide (60.3 mg, 0.25 mmol, 1 equiv.), acetonitrile (2.4 mL), and MeOH (1.2 mL). The reaction mixture was concentrated *in vacuo*, liquid loaded with a minimum amount of CH_2Cl_2 onto a 12 g RediSep® silica column, and purified by column chromatography (0 – 45% EtOAc:cyclohexane). The appropriate column fractions were combined and concentrated *in vacuo* to give an off-white-coloured solid. The solid was further dried in a vacuum oven at 40 °C to afford 2-(5-(pyridin-4-yl)-1,3,4-oxadiazol-2-yl)phenol (13.3 mg, 56 µmol, 22%) as an off-white-coloured solid.

^{1}H NMR (400 MHz, $CDCl_3$) δ 10.06 (s, 1H), 8.94 – 8.82 (m, 2H), 8.04 – 7.97 (m, 2H), 7.88 (dd, *J* = 7.9, 1.5 Hz, 1H), 7.50 (ddd, *J* = 8.6, 7.2, 1.7 Hz, 1H), 7.17 (dd, *J* = 8.6, 0.7 Hz, 1H), 7.10 – 7.04 (m, 1H). Purity = 95%

^{13}C NMR (101 MHz, $CDCl_3$) δ 165.3, 161.6, 158.1, 151.2, 134.5, 130.6, 126.8, 120.5, 120.3, 118.0, 107.7.

LCMS: t_r = 0.86 min (HpH), ES+ (m/z) 239.97 ($[M+H]^+$, 100%), ES- (m/z) 238.10 ($[M+H]^-$, 100%), Purity = 100%

FTIR: ν_{max} / cm^{-1} 3156w, 3041w, 1624m.

HRMS: m/z Calcd. for $C_{13}H_{10}N_3O_2^+$ $[M+H]^+$ 240.0768, found 240.0784.

2-(1*H*-Indol-7-yl)-5-phenyl-1,3,4-oxadiazole (4k)

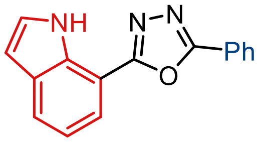

4k

The synthesis of **4k** was conducted following the general procedure P4, using *N*'-((1*H*-indol-7-yl)methylene)benzohydrazide (65.9 mg, 0.25 mmol, 1 equiv.). The reaction mixture was concentrated *in vacuo*, liquid loaded with a minimum amount of CH_2Cl_2 onto a 12 g RediSep® silica column, and purified by column chromatography (0 – 40% EtOAc:cyclohexane). The appropriate column fractions were combined and concentrated *in vacuo* to give an off-white-coloured solid. The solid was further dried in a vacuum oven at 40 °C to afford 2-(1*H*-indol-7-yl)-5-phenyl-1,3,4-oxadiazole (30.9 mg, 0.12 mmol, 47%) as an off-white-coloured solid.

^{1}H NMR (400 MHz, $CDCl_3$) δ 10.21 (br s, 1H), 8.25 – 8.15 (m, 2H), 7.93 – 7.84 (m, 2H), 7.61 – 7.53 (m, 3H), 7.44 – 7.41 (m, 1H), 7.27 (t, *J* = 7.7 Hz, 1H), 6.68 (dd, *J* = 3.2, 2.2 Hz, 1H). Purity = 95%

^{13}C NMR (101 MHz, $CDCl_3$) δ 164.3, 163.8, 133.3, 131.9, 129.3, 129.0, 127.2, 125.8, 125.1, 124.0, 120.5, 119.6, 106.8, 103.1.

LCMS: t_r = 1.33 min (HpH), ES+ (m/z) 262.06 ($[M+H]^+$, 100%), ES- (m/z) 260.23 ($[M-H]^-$, 100%), Purity = 100%.

FTIR: ν_{max} / cm^{-1} 3295br,m, 3057w.

HRMS: m/z Calcd. for $C_{16}H_{12}N_3O^+$ $[M+H]^+$ 262.0975, found 262.0988.

2-(4-Methylthiophen-2-yl)-5-phenyl-1,3,4-oxadiazole (4l)

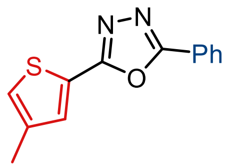

4l

The synthesis of **4l** was conducted following the general procedure P4, using *N*'-((4-methylthiophen-2-yl)methylene)benzohydrazide (61.1 mg, 0.25 mmol, 1 equiv.). The reaction mixture was concentrated *in vacuo*, liquid loaded with a minimum amount of CH_2Cl_2 onto a 12 g RediSep® silica column, and purified by column chromatography (0 – 40% EtOAc:cyclohexane). The appropriate column fractions were combined and concentrated *in vacuo* to give an off-white-coloured solid. The solid was further dried in a vacuum oven at 40 °C to afford 2-(4-methylthiophen-2-yl)-5-phenyl-1,3,4-oxadiazole (32.9 mg, 0.14 mmol, 54%) as an off-white-coloured solid.

^{1}H NMR (400 MHz, $CDCl_3$) δ 8.15 – 8.07 (m, 2H), 7.65 (d, *J* = 1.0 Hz, 1H), 7.58 – 7.49 (m, 3H), 7.17 – 7.13 (m, 1H), 2.35 (d, *J* = 0.7 Hz, 3H). Purity = 95%

^{13}C NMR (101 MHz, $CDCl_3$) δ 164.1, 161.1, 139.1, 131.9, 131.8, 129.2, 127.1, 125.9, 124.9, 123.9, 15.7.

LCMS: t_r = 1.24 min (HpH), ES+ (m/z) 243.08 ($[M+H]^+$, 100%), Purity = 100%.

FTIR: ν_{max} / cm^{-1} 3070w, 2928w.

HRMS: m/z Calcd. for $C_{16}H_{12}N_3O^+$ $[M+H]^+$ 243.0587, found 243.0599.

***tert*-Butyl 3-(5-phenyl-1,3,4-oxadiazol-2-yl)-1*H*-pyrrolo[2,3-*b*]pyridine-1-carboxylate (4m)**

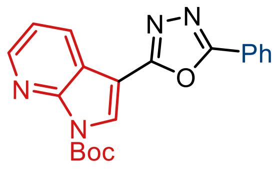

4m

The synthesis of **4m** was conducted following the general procedure P4, using *N*'-((1*H*-benzo[*d*][1,2,3]triazol-5-yl)methylene)benzohydrazide (66.4 mg, 0.25 mmol, 1 equiv.). The reaction mixture was concentrated *in vacuo*, liquid loaded with a minimum amount of CH_2Cl_2 onto a 12 g RediSep® silica column, and purified by column chromatography (0 – 50% EtOAc:cyclohexane). The appropriate column fractions were combined and concentrated *in vacuo* to give an off-white-coloured solid. The solid was further dried in a vacuum oven at 40 °C to afford *tert*-butyl 3-(5-phenyl-1,3,4-oxadiazol-2-yl)-1*H*-pyrrolo[2,3-*b*]pyridine-1-carboxylate (34.1 mg, 94 µmol, 38%) as an off-white-coloured solid.

^{1}H NMR (400 MHz, $CDCl_3$) δ 8.67 (dd, *J* = 7.9, 2.0 Hz, 1H), 8.65 – 8.63 (m, 1H), 8.38 (s, 1H), 8.20 – 8.13 (m, 2H), 7.60 – 7.52 (m, 3H), 7.40 (dd, *J* = 7.9, 4.9 Hz, 1H), 1.74 (s, 9H). Purity = 95%

^{13}C NMR (101 MHz, $CDCl_3$) δ 163.9, 160.4, 148.3, 147.3, 146.9, 132.0, 130.6, 129.3, 127.4, 127.1, 123.9, 120.0, 119.7, 103.6, 85.8, 28.3.

LCMS: t_r = 1.32 min (HpH), ES+ (m/z) 363.22 ($[M+H]^+$, 20%), Purity = 100%.

FTIR: ν_{max} / cm^{-1} 3005w, 2942w, 1598s.

HRMS: m/z Calcd. for $C_{40}H_{36}N_8O_6Na^+$ $[2M+Na]^+$ 747.2650, found 747.2648.

2-(1*H*-Benzo[*d*][1,2,3]triazol-5-yl)-5-phenyl-1,3,4-oxadiazole (4n)

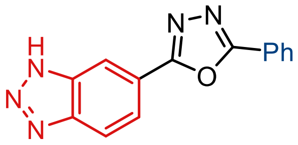

4n

The synthesis of **4n** was conducted following the general procedure P4, using *N*'-((1*H*-benzo[*d*][1,2,3]triazol-5-yl)methylene)benzohydrazide (66.4 mg, 0.25 mmol, 1 equiv.), acetonitrile (3.6 mL), and MeOH (0.1 mL). The reaction mixture was concentrated *in vacuo*, liquid loaded with a minimum amount of CH_2Cl_2 onto a 12 g RediSep® silica column, and purified by column chromatography (0 – 100% EtOAc + 1% Et_3N:cyclohexane). The appropriate column fractions were combined and concentrated *in vacuo* to give yellow-coloured solid. The solid was further dried in a vacuum oven at 40 °C to afford 2-(1*H*-benzo[*d*][1,2,3]triazol-5-yl)-5-phenyl-1,3,4-oxadiazole (15.5 mg, 53 µmol, 21%, 90% purity) as a yellow-coloured solid.

^{1}H NMR (600 MHz, MeOD-d_4) δ 8.72 (s, 1H), 8.27 (dd, *J* = 8.8, 1.5 Hz, 1H), 8.22 – 8.18 (m, 2H), 8.06 (d, *J* = 8.4 Hz, 1H), 7.66 – 7.60 (m, 3H). NH not observed. Purity = 90%

^{13}C NMR (151 MHz, MeOD-d_4) δ 166.5, 166.1, 133.4, 132.9, 130.5, 129.5, 128.6, 128.1, 125.6, 124.8, 122.1, 116.4 (br s).

LCMS: t_r = 0.70 min (HpH), ES+ (m/z) 264.04 ($[M+H]^+$, 100%), ES- (m/z) 262.24 ($[M-H]^-$, 100%), Purity = 100%.

FTIR: ν_{max} / cm^{-1} 3099w, 3060w, 2919w.

HRMS: m/z Calcd. for $C_{14}H_{10}N_5O^+$ $[M+H]^+$ 264.0880 found 264.0896.

4-(2-Phenyl-6-(5-phenyl-1,3,4-oxadiazol-2-yl)thieno[3,2-*d*]pyrimidin-4-yl)morpholine (4o)

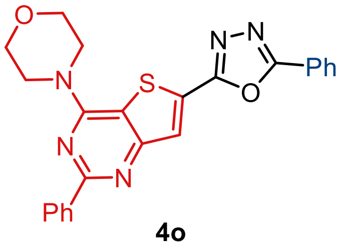

4o

The synthesis of **4o** was conducted following the general procedure P4, using *N*'-((4-morpholino-2-phenylthieno[3,2-*d*]pyrimidin-6-yl)methylene)benzohydrazide (111 mg, 0.25 mmol, 1 equiv.), a stir rate of 800 rpm, and a total charge of 7 F mol^{-1}. The reaction mixture was concentrated *in vacuo*, liquid loaded with a minimum amount of CH_2Cl_2 onto a 12 g RediSep® silica column, and purified by column chromatography (0 – 30% EtOAc:cyclohexane). The appropriate column fractions were combined and concentrated *in vacuo* to give a yellow-coloured solid. The solid was further dried in a vacuum oven at

40 °C to afford 4-(2-phenyl-6-(5-phenyl-1,3,4-oxadiazol-2-yl)thieno[3,2-*d*]pyrimidin-4-yl)morpholine (27.0 mg, 61 µmol, 24%) as a yellow-coloured solid.

^{1}H NMR (400 MHz, $CDCl_3$) δ 8.51 – 8.41 (m, 2H), 8.19 – 8.13 (m, 2H), 8.15 (s, 1H), 7.59 (s, 3H), 7.48 (d, *J* = 2.0 Hz, 3H), 4.16 – 4.08 (m, 4H), 3.96 – 3.91 (m, 4H). Purity = 95%

^{13}C NMR (101 MHz, $CDCl_3$) δ 165.4, 162.3, 161.6, 160.4, 158.4, 138.2, 132.5, 130.5, 130.0, 129.4, 128.6, 128.4, 127.4, 127.3, 123.4, 114.6, 66.9, 46.7.

LCMS: t_r = 1.51 min (HpH), ES+ (m/z) 442.16 ($[M+H]^+$, 100%), Purity = 98%.

FTIR: ν_{max} / cm^{-1} 3041w, 2969w, 2856w, 1522s.

HRMS: m/z Calcd. for $C_{24}H_{20}N_5O_2S^+$ $[M+H]^+$ 442.1332, found 442.1341.

2-Chloro-4-(((1*R*,2*S*)-1-(5-(4-cyanophenyl)-1,3,4-oxadiazol-2-yl)-2-hydroxypropyl)amino)-3-methylbenzonitrile (4p)

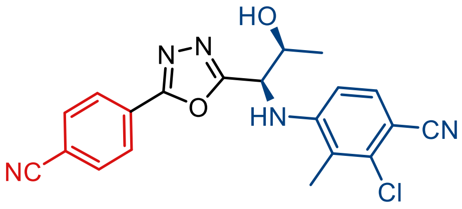

4p, Vosilasarm

The synthesis of **4p** was conducted following the general procedure P4, using (2*R*,3*S*)-2-((3-chloro-4-cyano-2-methylphenyl)amino)-*N*'-(4-cyanobenzylidene)-3-hydroxybutanehydrazide (96.5 mg, 0.24 mmol, 1 equiv.), tetraethylammonium tetrafluoroborate (53.0 mg, 0.24 mmol, 1 equiv.), and DABCO (27.4 mg, 0.24 mmol, 1 equiv.). The reaction mixture was concentrated *in vacuo*, liquid loaded with a minimum amount of CH_2Cl_2 onto a 12 g RediSep® silica column, and purified by column chromatography (0 – 75% EtOAc:cyclohexane). The appropriate column fractions were combined and concentrated *in vacuo* to give a beige-coloured gum. The gum was purified by reverse phase prep HPLC (15 – 65% MeCN:10 mM aqueous ammonium bicarbonate). The appropriate fractions were combined and concentrated *in vacuo* to give a gum which was further dried in a vacuum oven at 40 °C to afford 2-chloro-4-(((1*R*,2*S*)-1-(5-(4-cyanophenyl)-1,3,4-oxadiazol-2-yl)-2-hydroxypropyl)amino)-3-methylbenzonitrile (39.6 mg, 93 µmol, 38%, 92% purity) as a colourless gum.

^{1}H NMR (400 MHz, $CDCl_3$) δ 8.12 – 8.07 (m, 2H), 7.82 – 7.77 (m, 2H), 7.41 (d, *J* = 8.6 Hz, 1H), 6.66 (d, *J* = 8.6 Hz, 1H), 5.23 (d, *J* = 8.6 Hz, 1H), 4.78 (dd, *J* = 8.4, 2.8 Hz, 1H), 4.67 – 4.59 (m, 1H), 2.77 (br d, *J* = 2.9 Hz, 1H), 2.36 (s, 3H), 1.44 (d, *J* = 6.4 Hz, 3H). Purity = 92%

^{13}C NMR (101 MHz, $CDCl_3$) δ 166.5, 164.0, 149.0, 137.3, 133.1, 132.9, 127.6, 127.2, 121.9, 117.8, 117.6, 115.9, 108.5, 102.7, 68.3. 54.9, 20.1, 13.9.

LCMS: t_r = 1.08 min (HpH), ES+ (m/z) 394.52 ($[M+H]^+$, 5%), Purity =100%

FTIR: ν_{max} / cm^{-1} 3418br,m, 3093w, 2981w, 2934w, 2228s, 1593vs.

HRMS: m/z Calcd. for $C_{20}H_{17}ClN_5O_2^+$ $[M+H]^+$ 394.1065, found 394.1069.

Spectroscopic data consistent with literature.[9]

(*E*)-2-Phenyl-5-styryl-1,3,4-oxadiazole (4q)

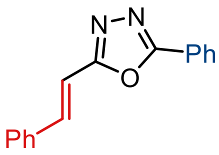

4q

The synthesis of **4q** was conducted following the general procedure P4, using *N*'-((*E*)-3-phenylallylidene)benzohydrazide (62.6 mg, 0.25 mmol, 1 equiv.) and a total charge of 3.5 F mol^{-1}. The reaction mixture was concentrated *in vacuo* and purified by reverse phase prep HPLC (15 – 80% MeCN:10 mM aqueous ammonium bicarbonate). The appropriate fractions were combined, and the solvent was evaporated in a blowdown at 40 °C to give a white-coloured solid. The solid was further dried in a vacuum oven at 40 °C to afford (*E*)-2-phenyl-5-styryl-1,3,4-oxadiazole (10.6 mg, 43 µmol, 17%) as an off-white-coloured solid.

^{1}H NMR (400 MHz, $CDCl_3$) δ 8.18 – 8.08 (m, 2H), 7.68 – 7.50 (m, 6H), 7.48 – 7.39 (m, 3H), 7.12 (d, *J* = 16.5 Hz, 1H). Purity = 95%

^{13}C NMR (101 MHz, $CDCl_3$) δ 164.4, 164.2, 139.1, 134.8, 131.9, 130.1, 129.2, 129.2, 127.6, 127.1, 123.9, 110.1.

LCMS: t_r = 1.27 min (HpH), ES+ (m/z) 249.09 ($[M+H]^+$, 100%), Purity = 98%.

FTIR: ν_{max} / cm^{-1} 3061w, 3028w, 1643m.

HRMS: m/z Calcd. for $C_{16}H_{13}N_2O^+$ $[M+H]^+$ 249.1022, found 249.1037.

Spectroscopic data consistent with literature.[10]

2-(3,4-Dihydro-2*H*-pyran-5-yl)-5-phenyl-1,3,4-oxadiazole (4r)

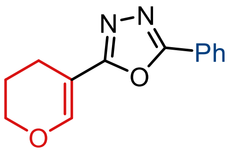

4r

The synthesis of **4r** was conducted following the general procedure P4, using *N*'-((3,4-dihydro-2*H*-pyran-5-yl)methylene)benzohydrazide (57.6 mg, 0.25 mmol, 1 equiv.). The reaction mixture was concentrated *in vacuo*, liquid loaded with a minimum amount of CH_2Cl_2 onto a 12 g RediSep® silica column, and purified by column chromatography (0 – 50% EtOAc:cyclohexane). The appropriate column fractions were combined and concentrated *in vacuo* to give a pale-yellow-coloured solid. The solid was further dried in a vacuum oven at 40 °C to afford 2-(3,4-dihydro-2*H*-pyran-5-yl)-5-phenyl-1,3,4-oxadiazole (10.2 mg, 45 µmol, 18%) as a pale-yellow-coloured solid.

^{1}H NMR (400 MHz, $CDCl_3$) δ 8.08 – 7.99 (m, 2H), 7.56 (s, 1H), 7.54 – 7.46 (m, 3H), 4.23 – 4.10 (m, 2H), 2.59 (br t, *J* = 6.3 Hz, 2H), 2.09 – 2.00 (m, 2H). Purity = 95%

^{13}C NMR (101 MHz, $CDCl_3$) δ 164.8, 163.2, 149.8, 131.4, 129.1, 126.9, 124.4, 100.4, 67.0, 21.4, 19.7.

LCMS: t_r = 1.05 min (HpH), ES+ (m/z) 229.14 ($[M+H]^+$, 100%), Purity = 100%.

FTIR: ν_{max} / cm^{-1} 3069w, 2921w, 1639s.

HRMS: m/z Calcd. for $C_{13}H_{13}N_2O_2^+$ $[M+H]^+$ 229.0972, found 229.0986.

2-(4-(Methylsulfonyl)phenyl)-5-phenyl-1,3,4-oxadiazole (4s)

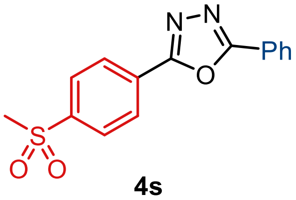

4s

The synthesis of **4s** was conducted following the general procedure P4, using *N*'-(4-(methylsulfonyl)benzylidene)benzohydrazide (75.6 mg, 0.25 mmol, 1 equiv.). The reaction mixture was concentrated *in vacuo*, liquid loaded with a minimum amount of CH_2Cl_2 onto a 12 g RediSep® silica column, and purified by column chromatography (0 – 40% EtOAc:cyclohexane). The appropriate column fractions were combined and concentrated *in vacuo* to give **4s** and **4d** as solids. The solids were further dried in a vacuum oven at 40 °C to afford 2-(4-(methylsulfonyl)phenyl)-5-phenyl-1,3,4-oxadiazole (**4s**, 27.8 mg, 83 µmol, 33%, 90% purity) as an off-white-coloured solid and 2,5-diphenyl-1,3,4-oxadiazole (**4d**, 7.8 mg, 35 µmol, 14%) as a white-coloured solid.

^{1}H NMR (400 MHz, $CDCl_3$) δ 8.41 – 8.32 (m, 2H), 8.20 – 8.11 (m, 4H), 7.65 – 7.54 (m, 3H), 3.12 (s, 3H). Purity = 90%.

^{13}C NMR (101 MHz, $CDCl_3$) δ 165.6, 163.2, 143.3, 132.4, 129.4, 128.9, 128.4, 127.9, 127.3, 123.6, 44.6.

LCMS: t_r = 0.99 min (HpH), ES+ (m/z) 301.11 ($[M+H]^+$, 100%), Purity = 98%.

FTIR: ν_{max} / cm^{-1} 3059w, 3011w, 2930w, 1543m.

HRMS: m/z Calcd. for $C_{15}H_{13}N_2O_3S^+$ $[M+H]^+$ 301.0641, found 301.0651.

Spectroscopic data consistent with literature.[23]

4-(5-Phenyl-1,3,4-oxadiazol-2-yl)phenyl acetate (4t) and 4-(5-phenyl-1,3,4-oxadiazol-2-yl)phenol (4t')

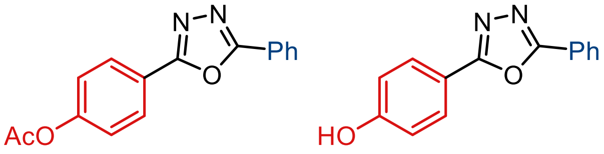

4t **4t'**

The synthesis of **4t** and **4u** was conducted following the general procedure P4, using 4-((2-benzoylhydrazineylidene)methyl)phenyl acetate (70.6 mg, 0.25 mmol, 1 equiv.). The reaction mixture was concentrated *in vacuo*, liquid loaded with a minimum amount of CH_2Cl_2 onto a 12 g RediSep® silica column, and purified by column chromatography (0 – 60% EtOAc:cyclohexane). The appropriate column fractions were combined and concentrated *in vacuo* to give **4t** and **4t'**. The solids were further dried in a vacuum oven at 40 °C to afford 4-(5-phenyl-1,3,4-oxadiazol-2-yl)phenyl acetate (**4t**, 21.2 mg, 76 µmol, 30%) as an off-white-coloured solid and 4-(5-phenyl-1,3,4-oxadiazol-2-yl)phenol (**4t'**, 15.7 mg, 66 µmol, 26%) as a white-coloured solid.

Data for **4t**:

^{1}H NMR (400 MHz, $CDCl_3$) δ 8.21 – 8.11 (m, 4H), 7.59 – 7.50 (m, 3H), 7.32 – 7.27 (m, 2H), 2.34 (s, 3H). Purity = 95%

^{13}C NMR (101 MHz, $CDCl_3$) δ 169.0, 164.8, 164.1, 153.4, 131.9, 129.3, 128.5, 127.1, 124.0, 122.6, 121.7, 21.3.

LCMS: t_r = 1.13 min (HpH), ES+ (m/z) 281.10 ($[M+H]^+$, 100%), Purity = 99%

FTIR: ν_{max} / cm^{-1} 3058w, 2919w, 1752s.

HRMS: m/z Calcd. for $C_{16}H_{13}N_2O_3^+$ $[M+H]^+$ 281.0921, found 281.0932.

Data for **4t'**:

^{1}H NMR (400 MHz, DMSO-d_6) δ 10.32 (br s, 1H), 8.13 – 8.05 (m, 2H), 8.00 – 7.92 (m, 2H), 7.67 – 7.57 (m, 3H), 7.02 – 6.95 (m, 2H). Purity = 95%

^{13}C NMR (101 MHz, DMSO-d_6) δ 164.2, 163.3, 160.8, 131.8, 129.4, 128.7, 126.5, 123.5, 116.1, 114.1.

LCMS: t_r = 0.87 min (HpH), ES+ (m/z) 239.14 ($[M+H]^+$, 100%), ES- (m/z) 237.15 ($[M-H]^-$, 100%), Purity = 100%.

FTIR: ν_{max} / cm^{-1} 3062br,w, 3020w, 1603m.

HRMS: m/z Calcd. for $C_{14}H_{11}N_2O_2^+$ $[M+H]^+$ 239.0815, found 239.0830.

2-(3,5-Difluorophenyl)-5-phenyl-1,3,4-oxadiazole (4u)

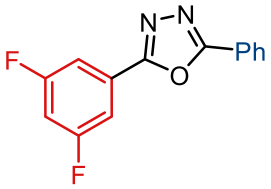

4u

The synthesis of **4u** was conducted following the general procedure P4, using *N*'-(3,5-difluoro-4-(4,4,5,5-tetramethyl-1,3,2-dioxaborolan-2-yl)benzylidene)benzohydrazide (96.6 mg, 0.25 mmol, 1 equiv.). The reaction mixture was concentrated *in vacuo*, liquid loaded with a minimum amount of CH_2Cl_2 onto a 12 g RediSep® silica column, and purified by column chromatography (0 – 40% EtOAc:cyclohexane). The appropriate column fractions were combined and concentrated *in vacuo* to give a white-coloured solid. The solid was further dried in a vacuum oven at 40 °C to afford 2-(3,5-difluorophenyl)-5-phenyl-1,3,4-oxadiazole (36.8 mg, 0.14 mmol, 57%) as a white-coloured solid.

^{1}H NMR (400 MHz, $CDCl_3$) δ 8.23 – 8.04 (m, 2H), 7.75 – 7.63 (m, 2H), 7.62 – 7.51 (m, 3H), 7.02 (tt, *J* = 8.7, 2.4 Hz, 1H). Purity = 95%

^{13}C NMR (101 MHz, $CDCl_3$) δ 165.2, 163.4 (dd, J_{CF} = 250.2, 12.2 Hz), 162.9, 132.3, 129.3, 127.2, 126.7 (t, J_{CF} = 10.7 Hz), 123.5, 110.2 (dd, J_{CF} = 19.8, 7.6 Hz), 107.4 (t, J_{CF} = 25.9 Hz).

^{19}F NMR (376 MHz, $CDCl_3$) δ -107.19 (s, 2F).

LCMS: t_r = 1.27 min (HpH), ES+ (m/z) 259.03 ($[M+H]^+$, 100%), Purity = 100%.

FTIR: ν_{max} / cm^{-1} 3092w, 2919w.

HRMS: m/z Calcd. for $C_{14}H_9F_2N_2O^+$ [M+H]$^+$ 259.0677, found 259.0690.

2-Ethoxy-5-(4-fluorophenyl)-1,3,4-oxadiazole (4v)

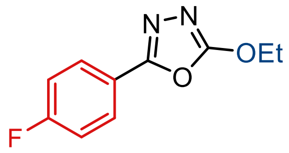

4v

The synthesis of **4v** was conducted following the general procedure P5, using ethyl hydrazinecarboxylate (26.1 mg, 10.25 mmol, 1 equiv.), 4-fluorobenzaldehyde (26.8 µL, 31.0 mg, 0.25 mmol, 1 equiv.), and acetonitrile (3.6 mL). For the hydrazone formation step, the reaction mixture was stirred at 50 °C for 8 h. Prior to electrolysis, electrolyte (1 equiv.) and DABCO (1 equiv.) were added and following electrolysis, the reaction mixture was concentrated *in vacuo*, liquid loaded with a minimum amount of CH_2Cl_2 onto a 12 g RediSep® silica column, and purified by column chromatography (0 – 40% EtOAc:cyclohexane). The appropriate column fractions were combined and concentrated *in vacuo* to give a white-coloured solid. The solid was further dried in a vacuum oven at 40 °C to afford 2-ethoxy-5-(4-fluorophenyl)-1,3,4-oxadiazole (29.6 mg, 0.13 mmol, 52%, 92% purity) as a white-coloured solid.

^{1}H NMR (400 MHz, $CDCl_3$) δ 7.98 – 7.90 (m, 2H), 7.20 – 7.12 (m, 2H), 4.61 (q, *J* = 7.1 Hz, 2H),1.52 (t, *J* = 7.1 Hz, 3H). Purity = 92%

^{13}C NMR (101 MHz, $CDCl_3$) δ 165.9, 164.6 (d, J_{CF} = 250.2 Hz), 159.8, 128.4 (d, J_{CF} = 8.8 Hz), 120.7 (d, J_{CF} = 2.9 Hz), 116.4 (d, J_{CF} = 22.7 Hz), 69.5, 14.5.

^{19}F NMR (376 MHz, $CDCl_3$) δ -107.81 (s, 1F).

LCMS: t_r = 1.00 min (HpH), ES+ (m/z) 208.95 ([M+H]$^+$, 100%), Purity = 90%

FTIR: ν_{max} / cm^{-1} 3110w, 3079w, 2986w, 2968w, 1747m, 1604s.

HRMS: m/z Calcd. for $C_{10}H_{10}FN_2O_2^+$ [M+H]$^+$ 209.0721, found 209.0729.

2-(1-Methyl-1*H*-imidazol-4-yl)-5-phenyl-1,3,4- (4w)

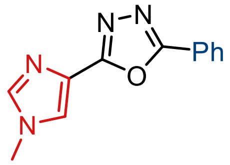

4w

The synthesis of **4w** was conducted following the general procedure P5, using benzohydrazide (34.1 mg, 0.25 mmol, 1 equiv.), 1-methyl-1*H*-imidazole-4-carbaldehyde (27.5 mg, 0.25 mmol, 1 equiv.), and acetonitrile (3.6 mL). For the hydrazone formation step, the reaction mixture was stirred for 30 min at room temperature then for 3 h at 50 °C. Prior to electrolysis, electrolyte (1 equiv.) and DABCO (1 equiv.) were added, and the reaction mixture was electrolysed until a total charge of 3 F mol^{-1} had been passed. Following electrolysis, the reaction mixture was concentrated *in vacuo*, liquid loaded with a

minimum amount of CH_2Cl_2 onto a 12 g RediSep® silica column, and purified by column chromatography (0 – 100% EtOAc:cyclohexane). The appropriate column fractions were combined and concentrated *in vacuo* to give a white-coloured solid. The solid was further dried in a vacuum oven at 40 °C to afford 2-(1-methyl-1*H*-imidazol-4-yl)-5-phenyl-1,3,4-oxadiazole (25.3 mg, 0.11 mmol, 43%, 95% purity) as a beige-coloured solid.

^{1}H NMR (400 MHz, $CDCl_3$) δ 8.20 – 8.10 (m, 2H), 7.74 (d, *J* = 1.0 Hz, 1H), 7.59 (s, 1H), 7.56 – 7.46 (m, 3H), 3.81 (s, 3H). Purity = 95%

^{13}C NMR (101 MHz, $CDCl_3$) δ 164.0, 160.8, 139.3, 131.6, 129.1, 128.0, 127.1, 124.0, 122.6, 34.1.

LCMS: t_r = 0.75 min (HpH), ES+ (m/z) 227.13 ([M+H]$^+$, 100%), Purity = 99%

FTIR: v_{max} / cm^{-1} 3082w, 3067w, 2952w, 1604m.

HRMS: m/z Calcd. for $C_{12}H_{11}N_4O^+$ [M+H]$^+$ 227.0927, found 227.0945.

m/z Calcd. for $C_{24}H_{20}N_8NaO_2^+$ [2M+Na]$^+$ 475.1601, found 475.1611.

2-(2-(Methylthio)pyrimidin-4-yl)-5-phenyl-1,3,4-oxadiazole (4x)

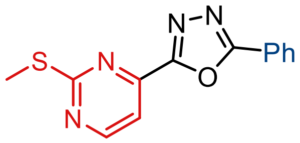

4x

The synthesis of **4x** was conducted following the general procedure P5, using benzohydrazide (34.1 mg, 0.25 mmol, 1 equiv.), 2-(methylthio)pyrimidine-4-carbaldehyde (38.6 mg, 0.25 mmol, 1 equiv.), and acetonitrile (3.6 mL). For the hydrazone formation step, the reaction mixture was stirred for 3 h at 50 °C. Prior to electrolysis, electrolyte (1 equiv.) and DABCO (1 equiv.) were added and following electrolysis, the reaction mixture was concentrated *in vacuo*, purified by reverse phase prep HPLC (20 – 60% MeCN: mM aqueous ammonium bicarbonate). The appropriate column fractions were combined and concentrated *in vacuo* to give a white-coloured solid. The solid was further dried in a vacuum oven at 40 °C to afford 2-(2-(methylthio)pyrimidin-4-yl)-5-phenyl-1,3,4-oxadiazole (30.2 mg, 0.11 mmol, 45%) as an off-white-coloured solid.

^{1}H NMR (400 MHz, $CDCl_3$) δ 8.74 (d, *J* = 4.9 Hz, 1H), 8.21 (dd, *J* = 8.1, 1.5 Hz, 2H), 7.85 (d, *J* = 4.9 Hz, 1H), 7.63 – 7.53 (m, 3H), 2.68 (s, 3H). Purity = 95%

^{13}C NMR (101 MHz, $CDCl_3$) δ 174.4, 166.4, 162.6, 158.7, 150.6, 132.6, 129.3, 127.7, 123.4, 113.9, 14.4.

LCMS: t_r = 1.10 (HpH), ES+ (m/z) 270.93 ([M+H]$^+$, 100%), Purity = 100%

FTIR: v_{max} / cm^{-1} 3071w, 2926w, 2851w, 1574m.

HRMS: m/z Calcd. for $C_{13}H_{11}N_4OS^+$ [M+H]$^+$ 271.0648, found 271.0661.

2-(5-(Furan-2-yl)-1,3,4-oxadiazol-2-yl)-2-methylpropan-1-ol (4y)

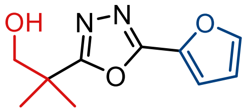

4y

The synthesis of **4y** was conducted following the general procedure P5, using 3-hydroxy-2,2-dimethylpropanal (25.5 mg, 0.25 mmol, 1 equiv.), furan-2-carbohydrazide (31.5 mg, 0.25 mmol, 1 equiv.), and MeOH (1.2 mL). For the hydrazone formation step, the reaction mixture was stirred for 2 h at room temperature. Prior to electrolysis, electrolyte (1 equiv.), DABCO (1 equiv.), and acetonitrile (2.4 mL) were added and following electrolysis, the reaction mixture was concentrated *in vacuo*, liquid loaded with a minimum amount of CH_2Cl_2 onto a 12 g RediSep® silica column, and purified by column chromatography (0 – 60% EtOAc:cyclohexane). The appropriate column fractions were combined and concentrated *in vacuo* to give a yellow-coloured solid. The solid was further dried in a vacuum oven at 40 °C to afford 2-(5-(furan-2-yl)-1,3,4-oxadiazol-2-yl)-2-methylpropan-1-ol (13.6 mg, 65 µmol, 26%) as a yellow-coloured solid.

^{1}H NMR (400 MHz, $CDCl_3$) δ 7.69 – 7.55 (m, 1H), 7.14 (d, *J* = 3.4 Hz, 1H), 6.58 (dd, *J* = 3.4, 1.7 Hz, 1H), 3.81 (br d, *J* = 4.4 Hz, 2H), 2.88 (br t, *J* = 6.4 Hz, 1H), 1.43 (s, 6H). Purity = 95%

^{13}C NMR (101 MHz, $CDCl_3$) δ 170.7, 157.7, 145.7, 139.6, 114.1, 112.2, 69.9, 38.5, 23.2.

LCMS: t_r = 0.69 (HpH), ES+ (m/z) 209.06 ([M+H]$^+$, 100%), Purity = 100%

FTIR: ν_{max} / cm^{-1} 3381br,w, 3130w, 2975w, 2935w, 1640m.

HRMS: m/z Calcd. for $C_{10}H_{13}N_2O_3^+$ [M+H]$^+$ 209.0921, found 209.0926.

4-(5-(2-Methoxyethyl)-1,3,4-oxadiazol-2-yl)-*N*,*N*-dimethylaniline (4z)

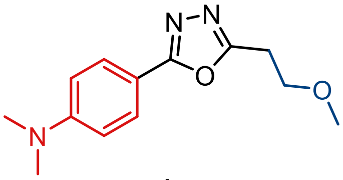

4z

The synthesis of **4z** was conducted following the general procedure P5, using 4-(dimethylamino)benzaldehyde (37.3 mg, 0.25 mmol, 1 equiv.), 3-methoxypropanehydrazide (26.7 µL, 29.6 mg, 0.25 mmol, 1 equiv.), and MeOH (1.2 mL). For the hydrazone formation step, the reaction mixture was stirred for 1 h at room temperature. Prior to electrolysis, electrolyte (1 equiv.), DABCO (1 equiv.), and acetonitrile (2.4 mL) were added and following electrolysis, the reaction mixture was concentrated *in vacuo*, liquid loaded with a minimum amount of CH_2Cl_2 onto a 12 g RediSep® silica column, and purified by column chromatography (0 – 60% EtOAc:cyclohexane). The appropriate column fractions were combined and concentrated *in vacuo* to give a yellow-coloured solid. The solid was further dried in a vacuum oven at 40 °C to 4-(5-(2-methoxyethyl)-1,3,4-oxadiazol-2-yl)-*N*,*N*-dimethylaniline (45.8 mg, 0.19 mmol, 74%) as a yellow-coloured solid.

^{1}H NMR (400 MHz, $CDCl_3$) δ 7.93 – 7.79 (m, 2H), 6.76 – 6.68 (m, 2H), 3.83 (t, *J* = 6.6 Hz, 2H), 3.39 (s, 3H), 3.16 (t, *J* = 6.6 Hz, 2H), 3.04 (s, 6H). Purity = 95%

^{13}C NMR (101 MHz, $CDCl_3$) δ 165.6, 163.5, 152.4, 128.3, 111.7, 111.3, 69.0, 58.9, 40.2, 26.6.

LCMS: t_r = 0.93 (HpH), ES+ (m/z) 248.12 ($[M+H]^+$, 100%), Purity = 97%

FTIR: ν_{max} / cm^{-1} 3045w, 2924w, 2897w, 2814w, 1613s, 1508s.

HRMS: m/z Calcd. for $C_{13}H_{18}N_3O_2^+$ $[M+H]^+$ 248.1394, found 248.1398.

5.1 Unsuccessful substrates

The synthesis of compounds **5a–f** was attempted, following the general procedure P4. According to LCMS analysis of the reaction mixtures, trace or no product was observed and the desired product could not be obtained.

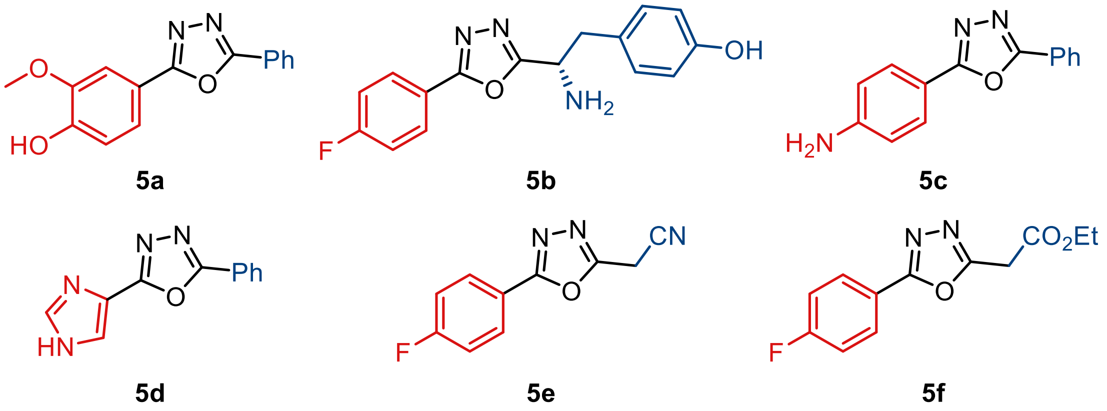

Scheme 1. Unsuccessful substrates.

6. Mechanistic studies

<u>Inert atmosphere</u>

The reaction was conducted inside a glovebox following the general procedure P4, using *N*'-(4-fluorobenzylidene)acetohydrazide (45.1 mg, 0.25 mmol, 1 equiv.) and anhydrous acetonitrile (3.6 mL). Following electrolysis, the reaction mixture was concentrated *in vacuo,* liquid loaded with a minimum amount of CH_2Cl_2 onto a 12 g RediSep® silica column, and purified by column chromatography (0 – 40% EtOAc:cyclohexane). The appropriate fractions were combined and concentrated *in vacuo* to afford 2-(4-fluorophenyl)-5-methyl-1,3,4-oxadiazole (9.9 mg, 49 µmol, 20%, 88% purity) as an off-white-coloured solid.

<u>Oxygen atmosphere</u>

The reaction was conducted following the general procedure P4, using *N*'-(4-fluorobenzylidene)acetohydrazide (45.1 mg, 0.25 mmol, 1 equiv.). The vial was capped, vacuum-purged with nitrogen (×3), and left under a static vacuum. The reaction mixture was placed under an oxygen atmosphere with an oxygen balloon and anhydrous acetonitrile (3.6 mL) was added. Following electrolysis, the reaction mixture was concentrated *in vacuo,* liquid loaded with a minimum amount of CH_2Cl_2 onto a 12 g RediSep® silica column, and purified by column chromatography (0 – 40%

EtOAc:cyclohexane). The appropriate fractions were combined and concentrated *in vacuo* to afford 2-(4-fluorophenyl)-5-methyl-1,3,4-oxadiazole (34.2 mg, 0.19 mmol, 73%, 95% purity) as an off-white-coloured solid.

Acrylate trap

The reaction was conducted following the general procedure P4, using *N*'-(4-fluorobenzylidene)acetohydrazide (22.5 mg, 125 µmol, 1 equiv.), tetraethylammonium tetrafluoroborate (54.3 mg, 250 µmol, 2 equiv.), DABCO (14 mg, 125 µmol, 1 equiv.), HFIP (17.1 µL, 27.3 mg, 162 µmol, 1.3 equiv.), and acetonitrile (3.5 mL). Following electrolysis, the electrodes were rinsed with acetone (1 mL) into the reaction mixture and a solution of 4-fluoroanisole in DMSO-d_6 (0.125 M, 1 mL, 125 µmol, 1 equiv.) was added. The reaction mixture was mixed thoroughly, transferred to an NMR tube (0.5 mL), and analysed by ^{19}F NMR spectroscopy without purification.

TEMPO trap

The reaction was conducted following the general procedure P4, using *N*'-(4-fluorobenzylidene)acetohydrazide (45.1 mg, 0.25 mmol, 1 equiv.) and the addition of TEMPO (39.2 mg, 0.25 mmol, 1 equiv.). Following electrolysis, 1-fluoro-4-methoxybenzene (28.4 µL, 31.6 mg, 0.25 mmol, 1 equiv.) was added and the reaction mixture was thoroughly mixed. An aliquot (0.2 mL) of the reaction mixture was transferred to an NMR tube and DMSO-d_6 (0.26 mL) was added. The contents of the NMR tube were thoroughly mixed and analysed by ^{19}F NMR spectroscopy without purification.

7. Cyclic voltammetry

Cyclic voltammetry was completed on a Zimmer and Peacock Ana Fleuve operating in CV mode. A Zimmer and Peacock platinum chip electrode (product number: A-P-PP-101-N). Cyclic voltammograms were recorded with a step potential of 0.005 V at a scan rate of 100 mV/s. The sample solutions were degassed with N_2 for at least 30 min before analysis.

Solution preparation:

A 0.1 M stock solution of electrolyte was prepared by dissolving tetraethylammonium tetrafluoroborate (1.09 g, 5.0 mmol) in anhydrous acetonitrile (50 mL).

A 10 mM ferrocene reference solution was prepared by dissolving ferrocene (37.2 mg, 0.2 mmol, 0.04 equiv.) in the electrolyte solution (20 mL).

A 10 mM analyte solution of hydrazone was prepared by dissolving *N*'-(4-fluorobenzylidene) acetohydrazide (18.0 mg, 0.1 mmol, 0.02 equiv.) in the electrolyte solution (10 mL).

A 10 mM analyte solution of DABCO was prepared by dissolving DABCO (11.2 mg, 0.1 mmol, 0.02 equiv.) in the electrolyte solution (10 mL).

8. NMR Spectra of Oxadiazole Products

^{1}H NMR (400 MHz, $CDCl_3$)

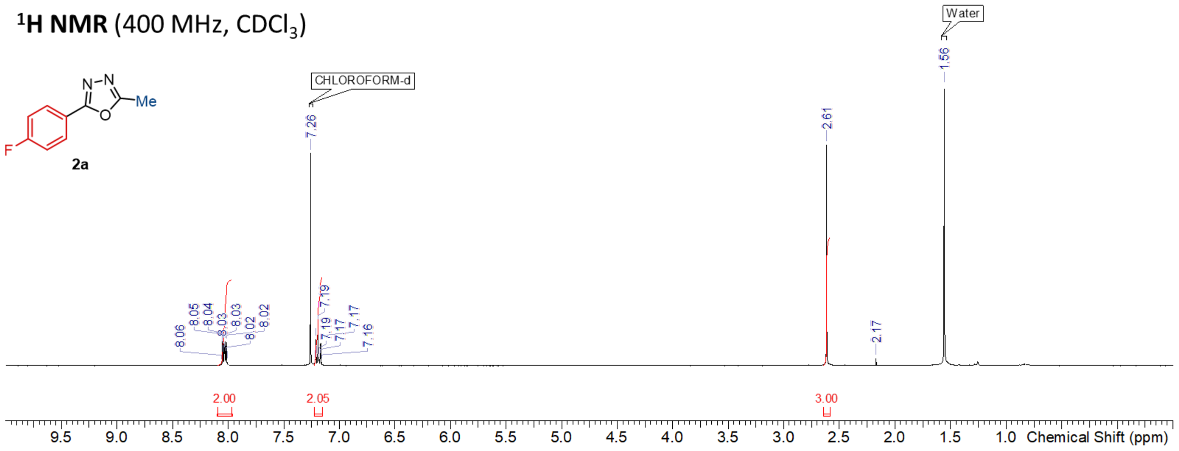

Figure S2. ^{1}H NMR spectrum (400 MHz, $CDCl_3$, 298 K) of **2a**.

^{13}C NMR (101 MHz, $CDCl_3$)

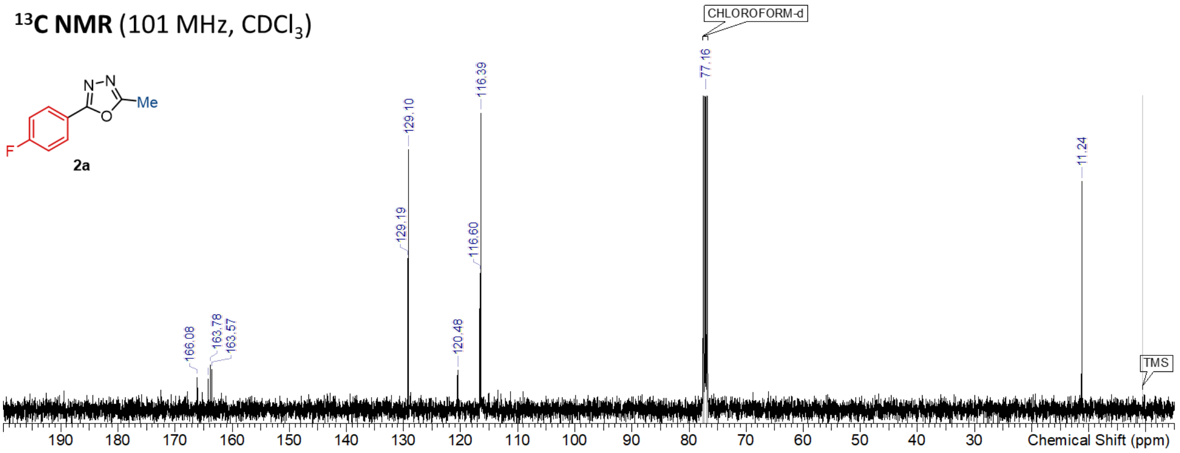

Figure S3. ^{13}C NMR spectrum (101 MHz, $CDCl_3$, 298 K) of **2a**.

^{19}F NMR (376 MHz, $CDCl_3$)

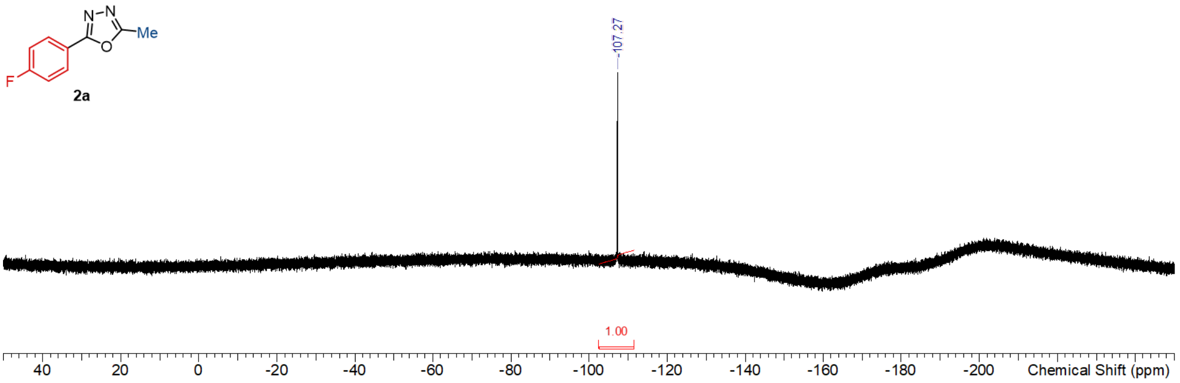

Figure S4. ^{19}F NMR spectrum (376 MHz, $CDCl_3$, 298 K) of **2a**.

^{1}**H NMR** (400 MHz, $CDCl_3$)

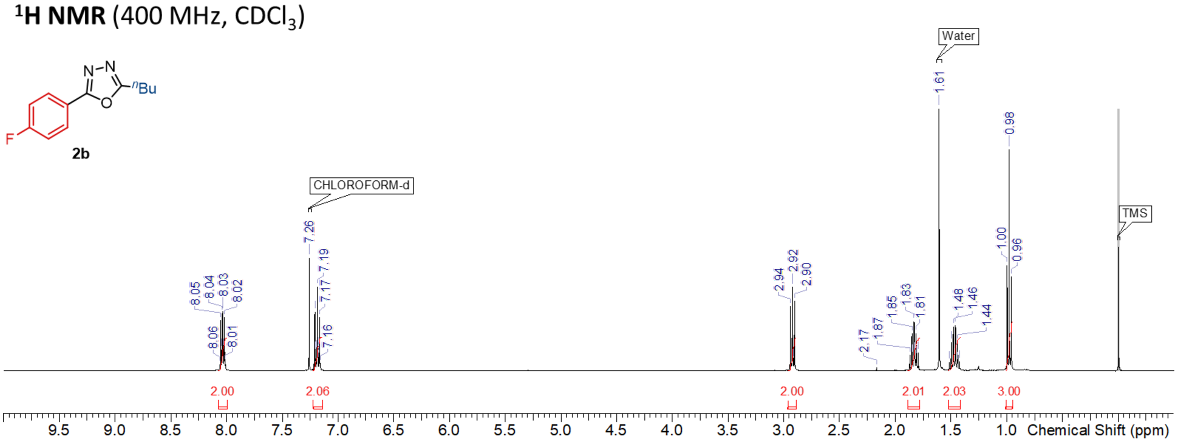

Figure S5. ^{1}H NMR spectrum (400 MHz, $CDCl_3$, 298 K) of **2b**.

^{13}**C NMR** (101 MHz, $CDCl_3$)

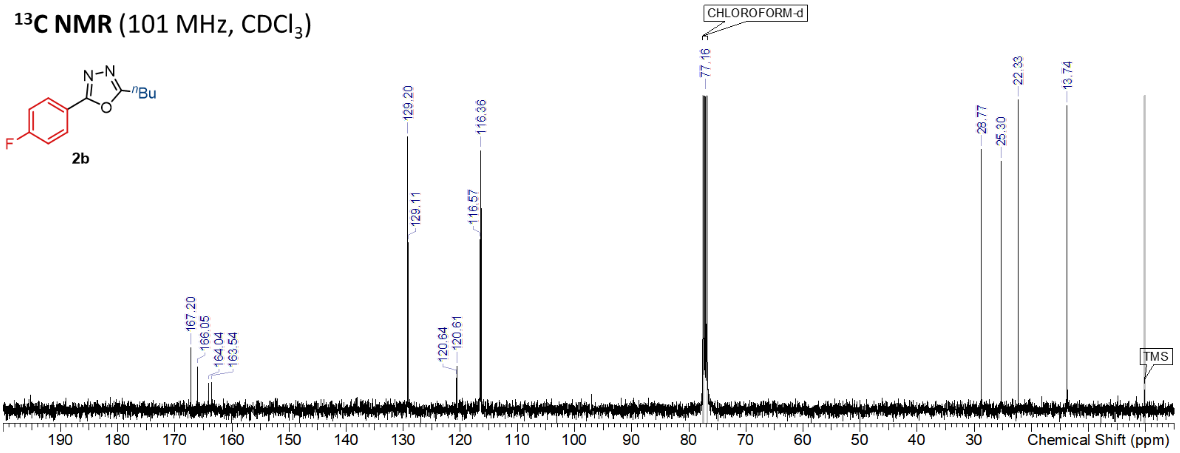

Figure S6. ^{13}C NMR spectrum (101 MHz, $CDCl_3$, 298 K) of **2b**.

^{19}**F NMR** (376 MHz, $CDCl_3$)

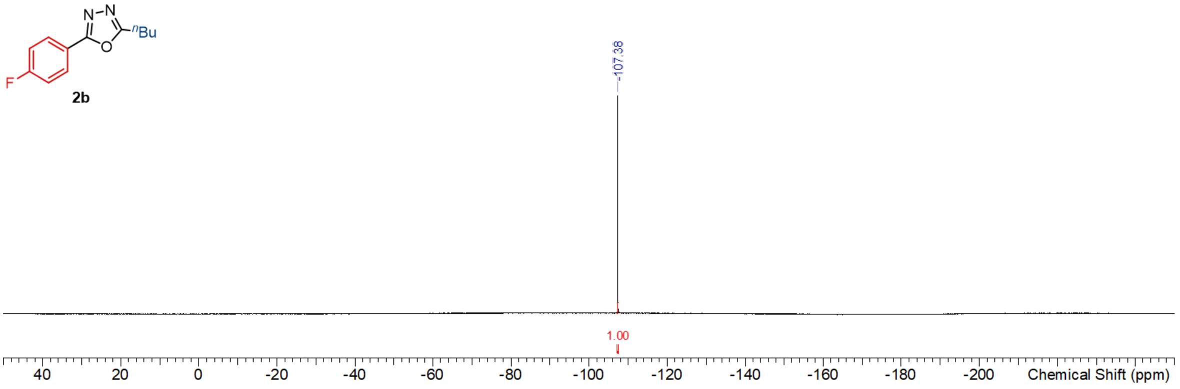

Figure S7. ^{19}F NMR spectrum (376 MHz, $CDCl_3$, 298 K) of **2b**.

^{1}H NMR (400 MHz, $CDCl_3$)

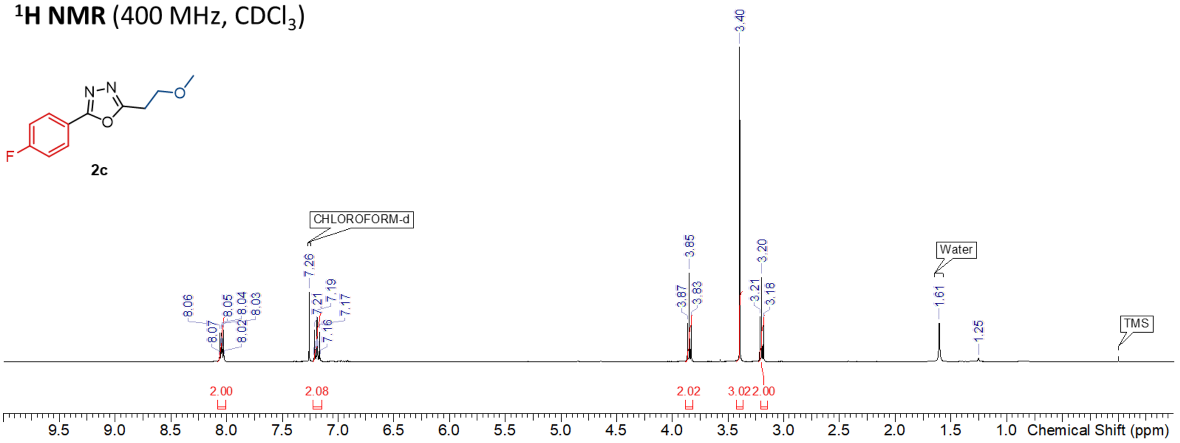

Figure S8. ^{1}H NMR spectrum (400 MHz, $CDCl_3$, 298 K) of **2c**.

^{13}C NMR (101 MHz, $CDCl_3$)

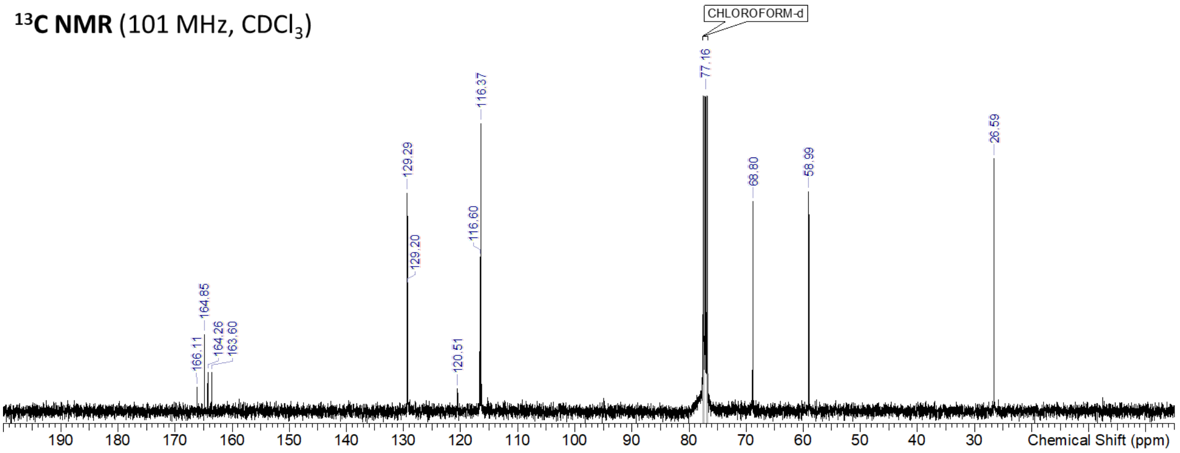

Figure S9. ^{13}C NMR spectrum (101 MHz, $CDCl_3$, 298 K) of **2c**.

^{19}F NMR (376 MHz, $CDCl_3$)

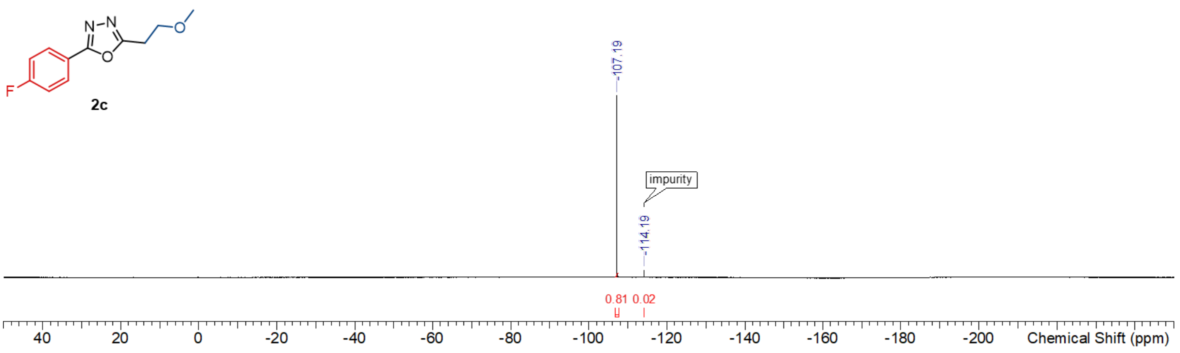

Figure S10. ^{19}F NMR spectrum (376 MHz, $CDCl_3$, 298 K) of **2c**.

^{1}**H NMR** (400 MHz, $CDCl_3$)

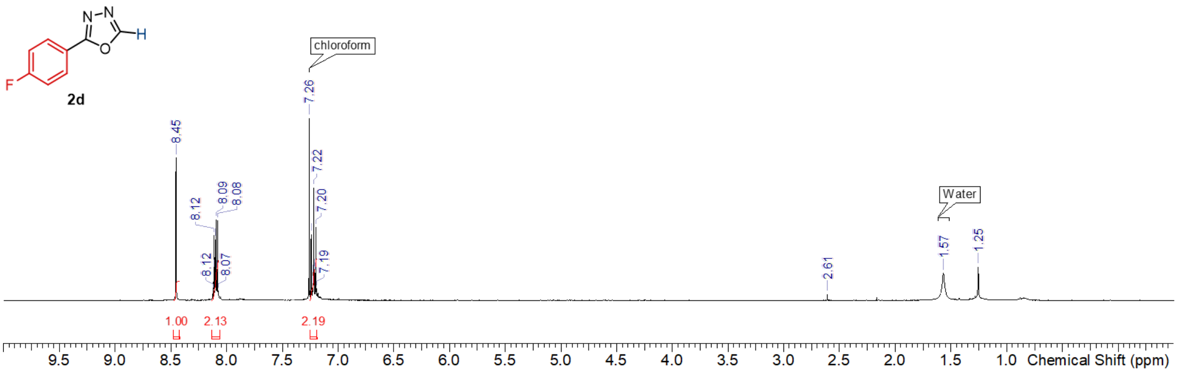

Figure S11. ^{1}H NMR spectrum (400 MHz, $CDCl_3$, 298 K) of **2d**.

^{13}**C NMR** (101 MHz, $CDCl_3$)

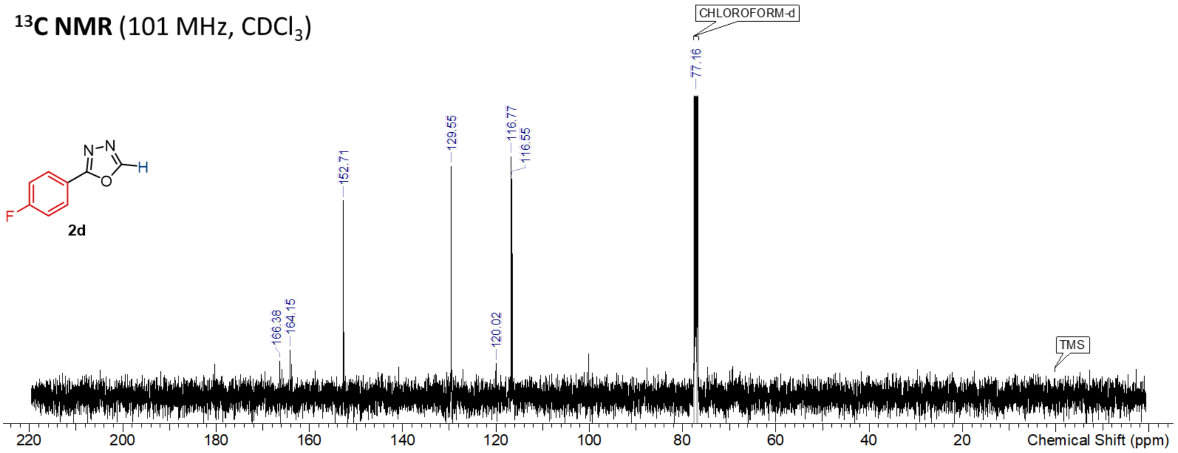

Figure S12. ^{13}C NMR spectrum (101 MHz, $CDCl_3$, 298 K) of **2d**.

^{19}**F NMR** (376 MHz, $CDCl_3$)

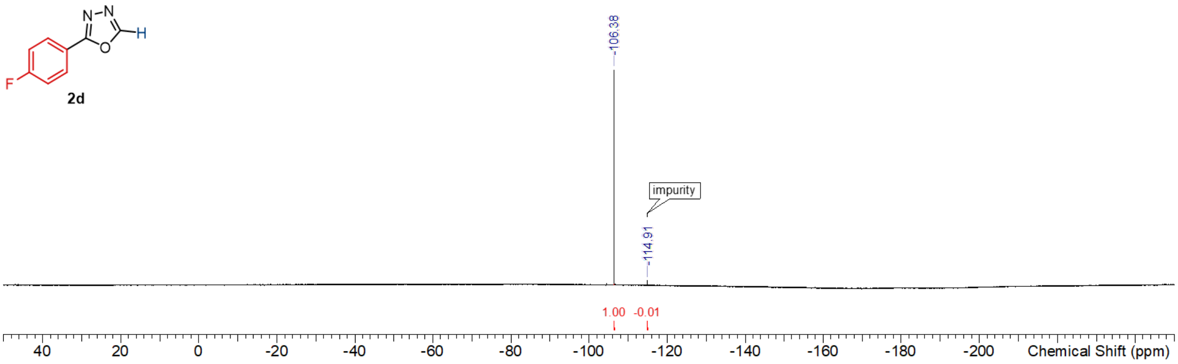

Figure S13. ^{19}F NMR spectrum (376 MHz, $CDCl_3$, 298 K) of **2d.**

[1]**H NMR** (400 MHz, $CDCl_3$)

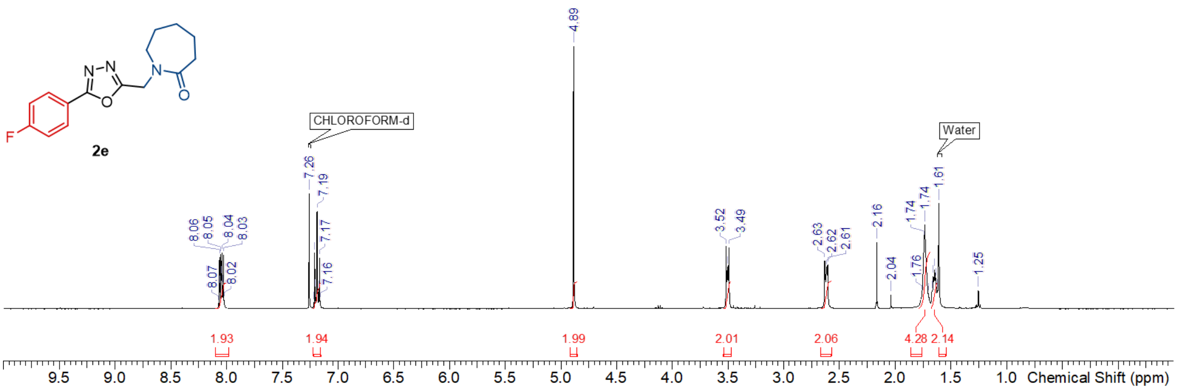

Figure S14. ^{1}H NMR spectrum (400 MHz, $CDCl_3$, 298 K) of **2e**.

[13]**C NMR** (101 MHz, $CDCl_3$)

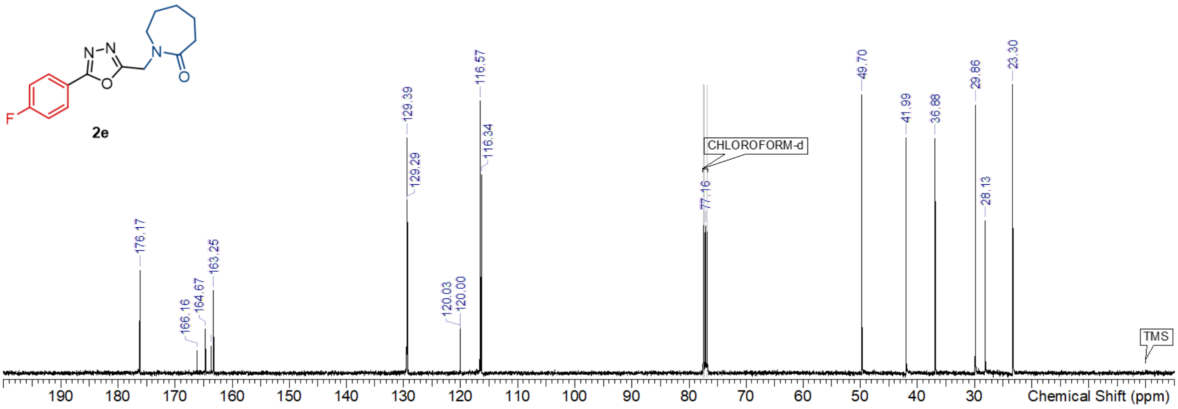

Figure S15. ^{13}C NMR spectrum (101 MHz, $CDCl_3$, 298 K) of **2e**.

[19]**F NMR** (376 MHz, $CDCl_3$)

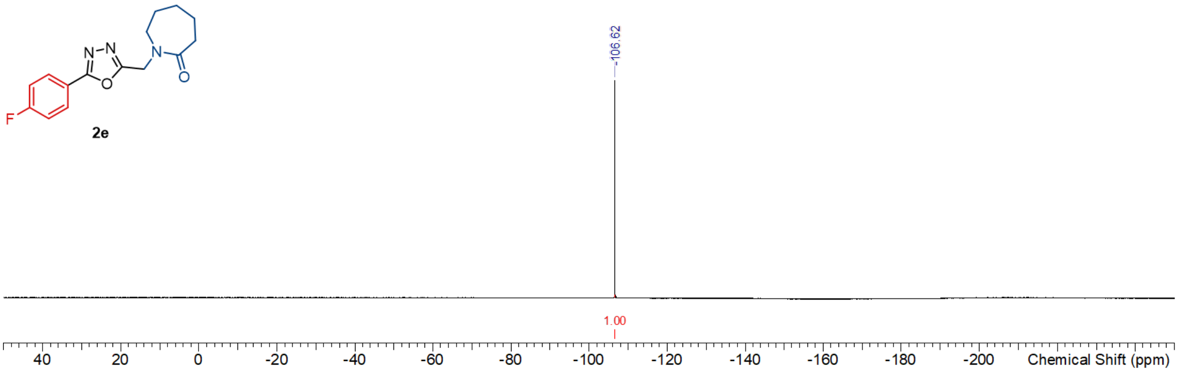

Figure S16. ^{19}F NMR spectrum (376 MHz, $CDCl_3$, 298 K) of **2e.**

^{1}H NMR (400 MHz, $CDCl_3$)

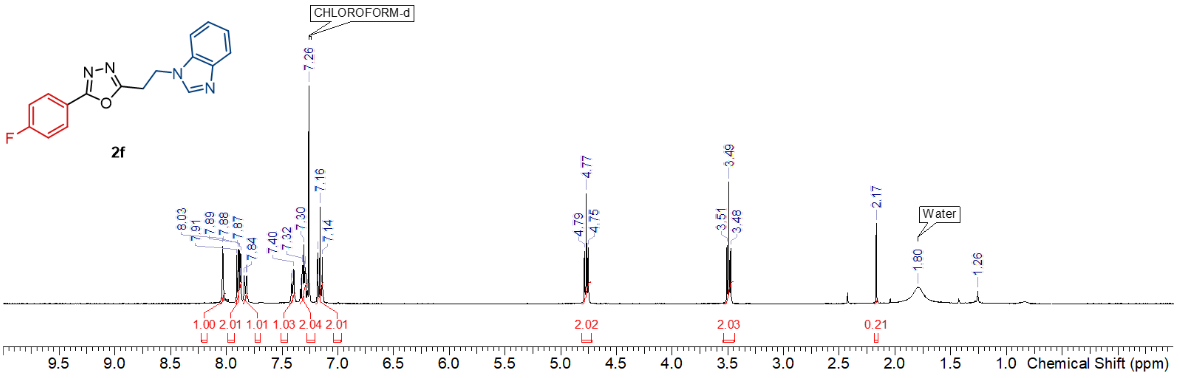

Figure S17. ^{1}H NMR spectrum (400 MHz, $CDCl_3$, 298 K) of **2f**.

^{13}C NMR (101 MHz, $CDCl_3$)

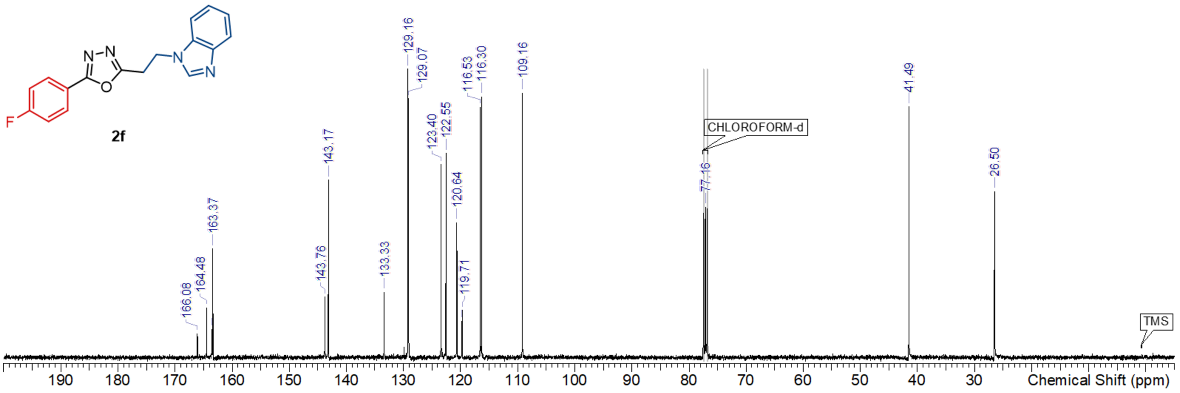

Figure S18. ^{13}C NMR spectrum (101 MHz, $CDCl_3$, 298 K) of **2f**.

^{19}F NMR (376 MHz, $CDCl_3$)

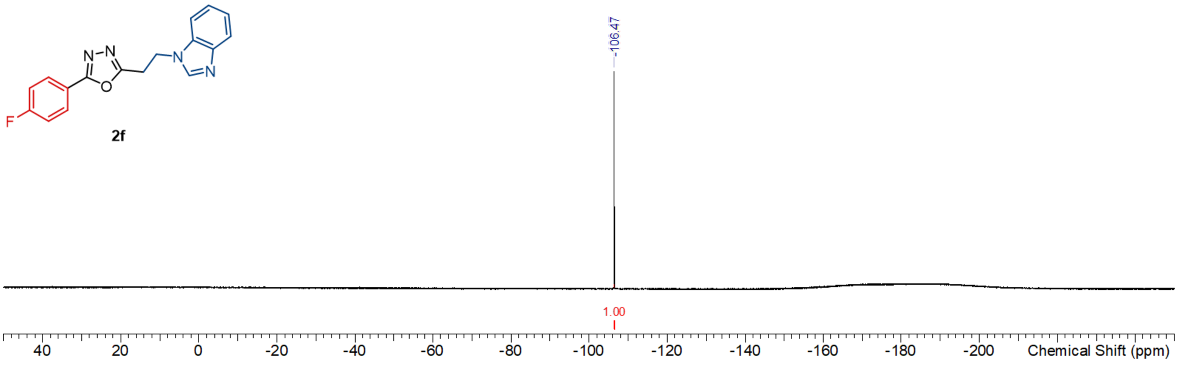

Figure S19. ^{19}F NMR spectrum (376 MHz, $CDCl_3$, 298 K) of **2f**.

^{1}H NMR (400 MHz, $CDCl_3$)

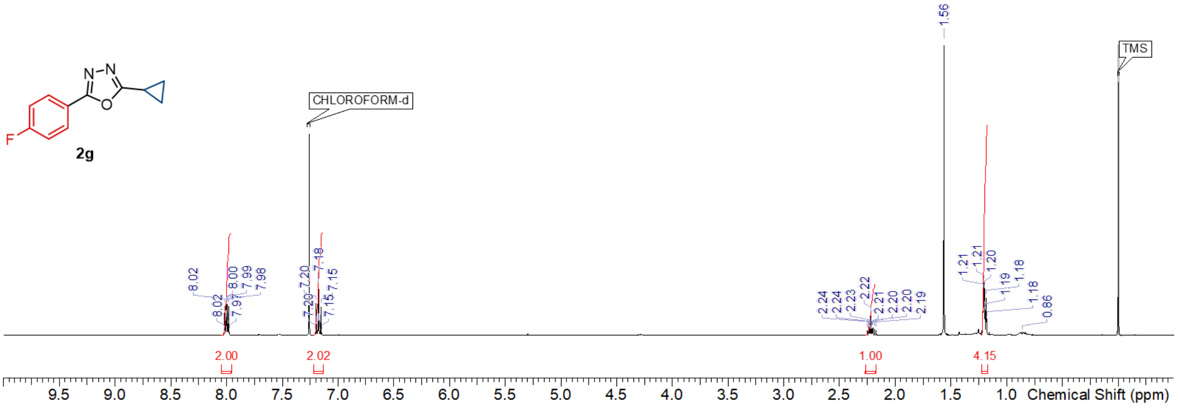

Figure S20. ^{1}H NMR spectrum (400 MHz, $CDCl_3$, 298 K) of **2g**.

^{13}C NMR (101 MHz, $CDCl_3$)

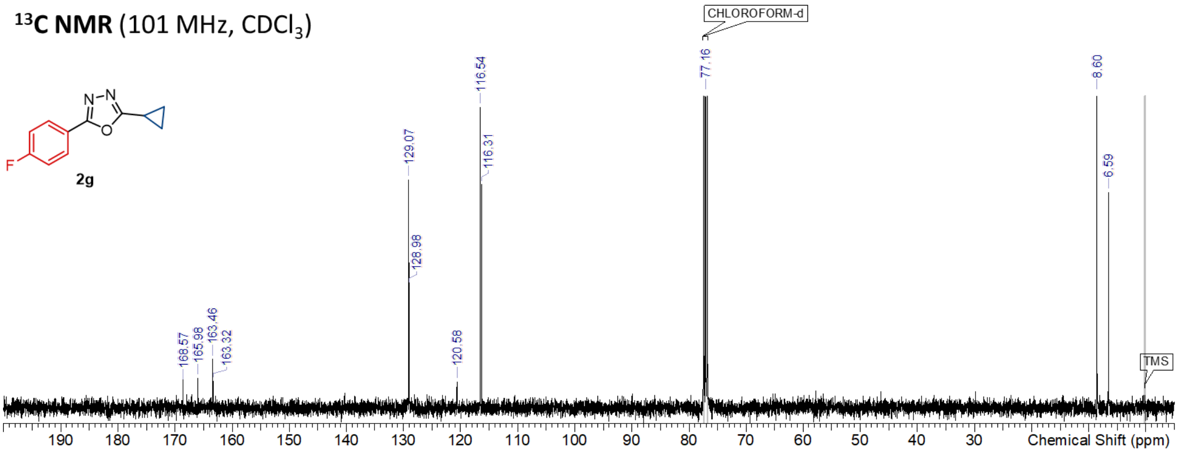

Figure S21. ^{13}C NMR spectrum (101 MHz, $CDCl_3$, 298 K) of **2g**.

^{19}F NMR (376 MHz, $CDCl_3$)

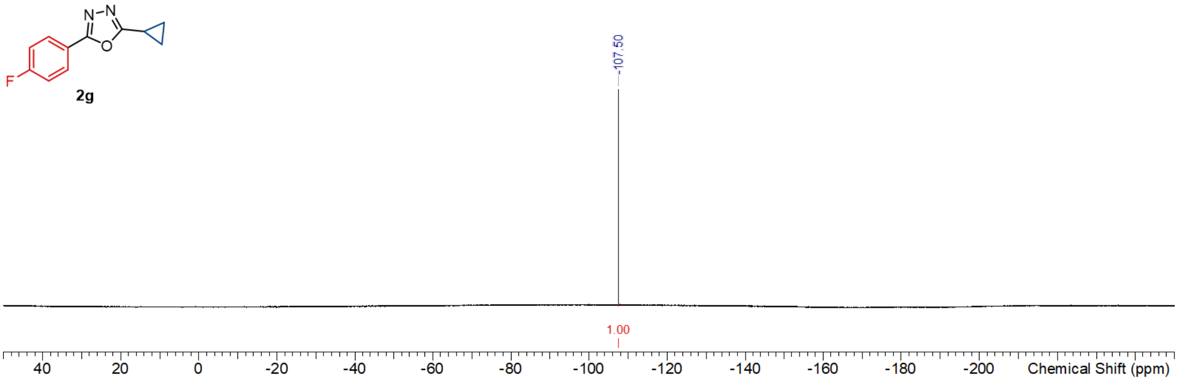

Figure S22. ^{19}F NMR spectrum (376 MHz, $CDCl_3$, 298 K) of **2g.**

^{1}**H NMR** (400 MHz, $CDCl_3$)

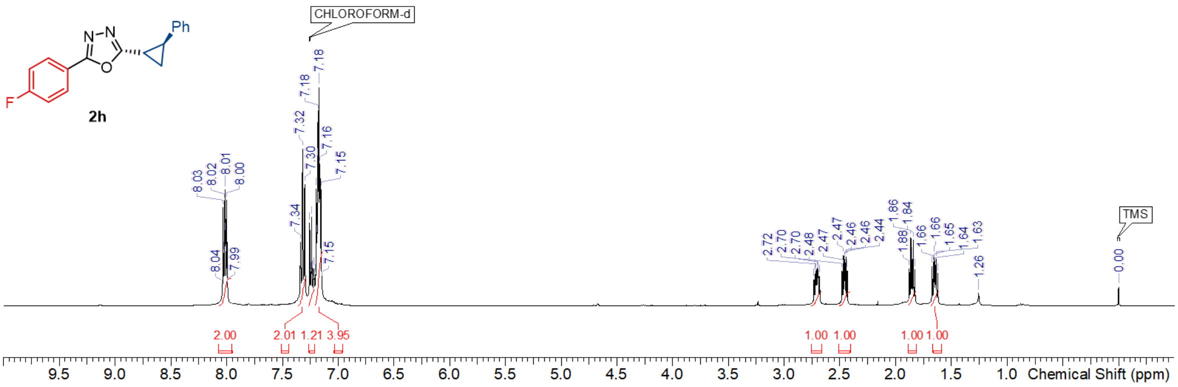

Figure S23. ^{1}H NMR spectrum (400 MHz, $CDCl_3$, 298 K) of **2h**.

^{13}**C NMR** (101 MHz, $CDCl_3$)

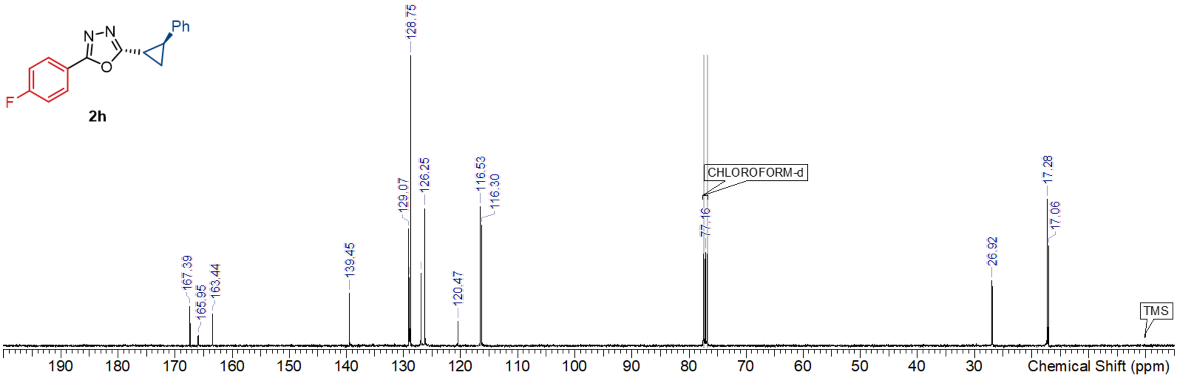

Figure S24. ^{13}C NMR spectrum (101 MHz, $CDCl_3$, 298 K) of **2h**.

^{19}**F NMR** (376 MHz, $CDCl_3$)

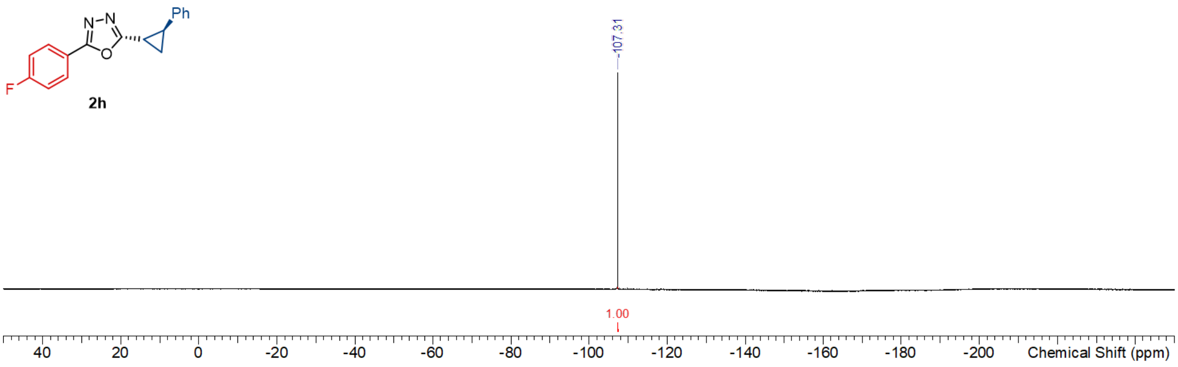

Figure S25. ^{19}F NMR spectrum (376 MHz, $CDCl_3$, 298 K) of **2h**.

^{1}H NMR (400 MHz, $CDCl_3$)

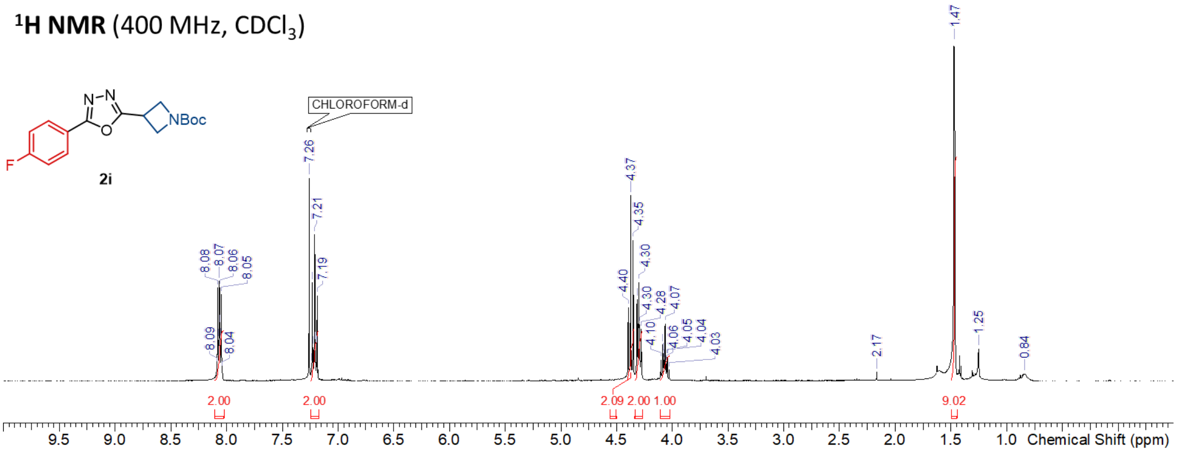

Figure S26. ^{1}H NMR spectrum (400 MHz, $CDCl_3$, 298 K) of **2i**.

^{13}C NMR (101 MHz, $CDCl_3$)

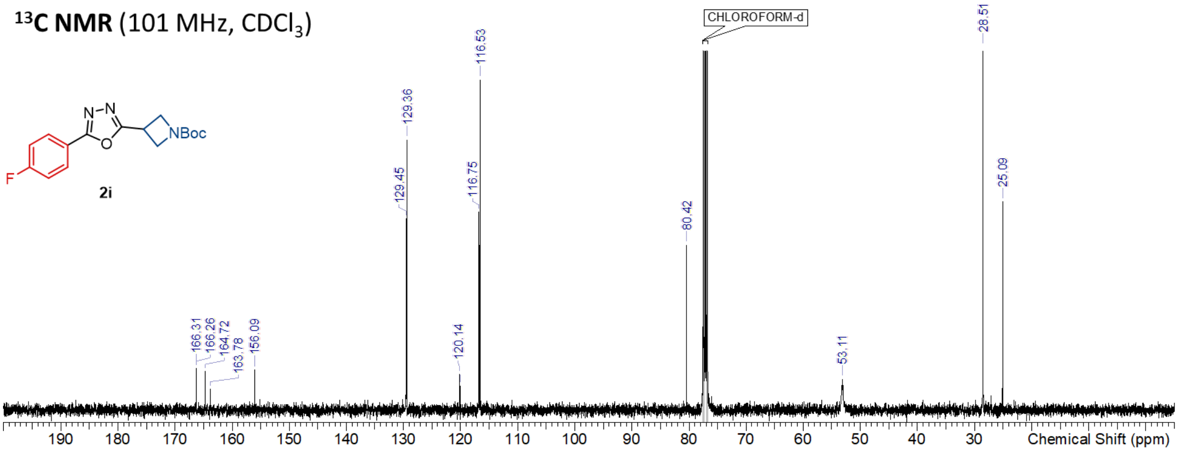

Figure S27. ^{13}C NMR spectrum (101 MHz, $CDCl_3$, 298 K) of **2i**.

^{19}F NMR (376 MHz, $CDCl_3$)

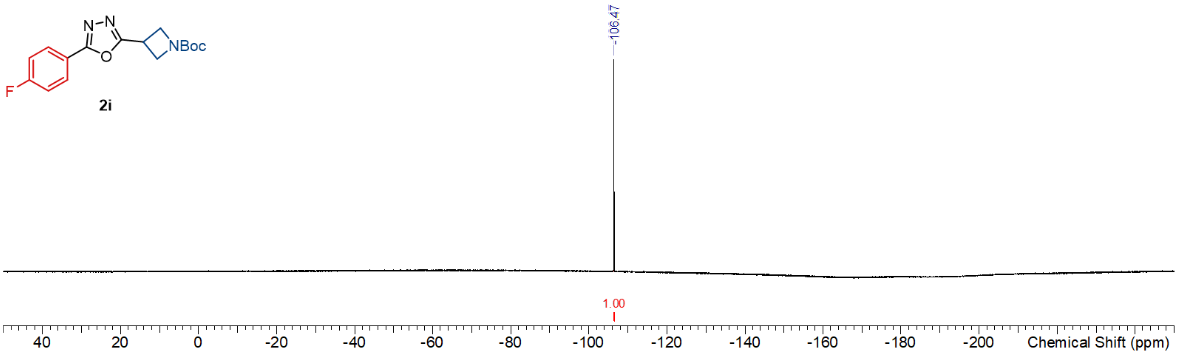

Figure S28. ^{19}F NMR spectrum (376 MHz, $CDCl_3$, 298 K) of **2i**.

^{1}H NMR (400 MHz, DMSO-d_6)

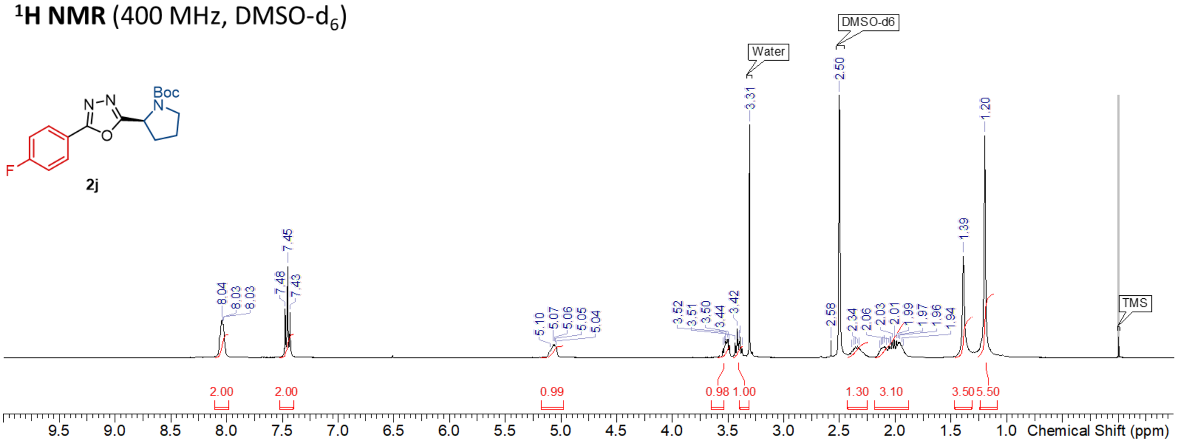

Figure S29. ^{1}H NMR spectrum (400 MHz, DMSO-d_6, 298 K) of **2j**.

^{13}C NMR (101 MHz, DMSO-d_6)

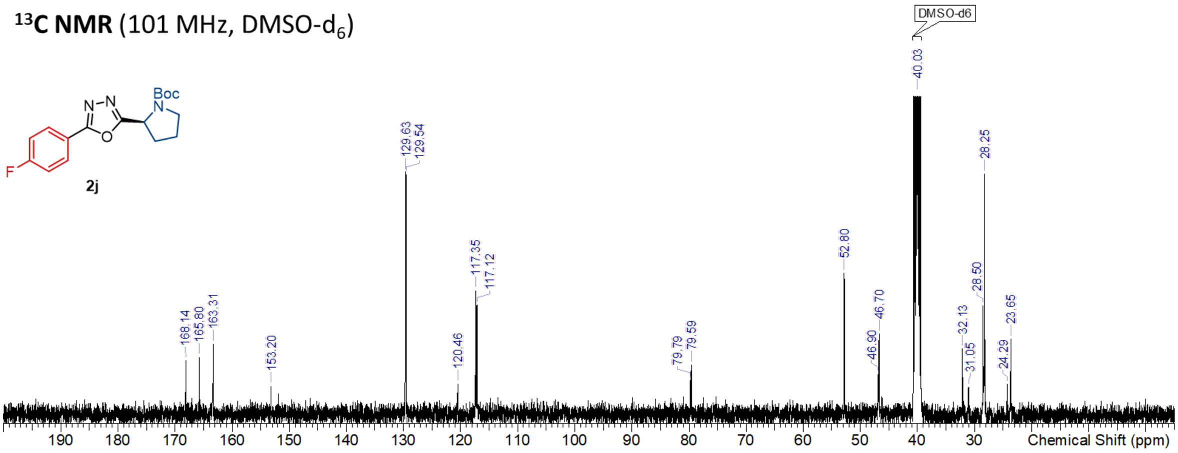

Figure S30. ^{13}C NMR spectrum (101 MHz, DMSO-d_6, 298 K) of **2j**.

^{19}F NMR (376 MHz, DMSO-d_6)

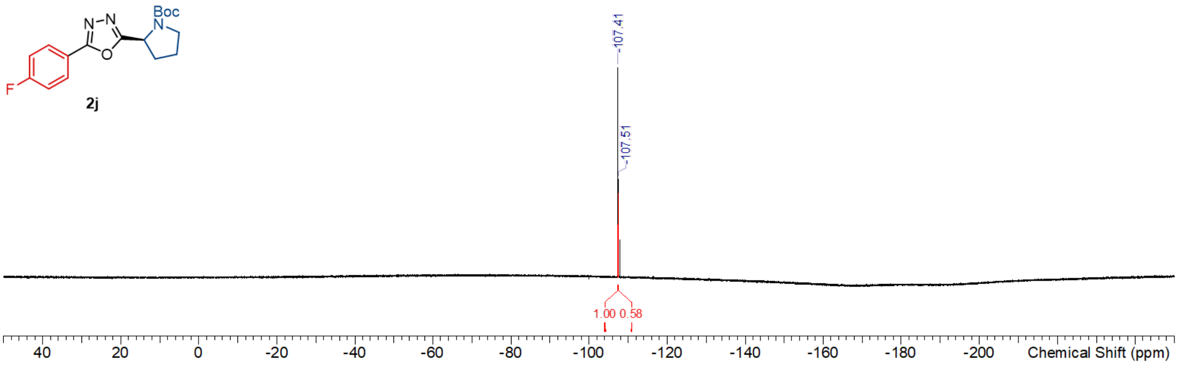

Figure S31. ^{19}F NMR spectrum (376 MHz, DMSO-d_6, 298 K) of **2j**.

^{1}H NMR (400 MHz, $CDCl_3$)

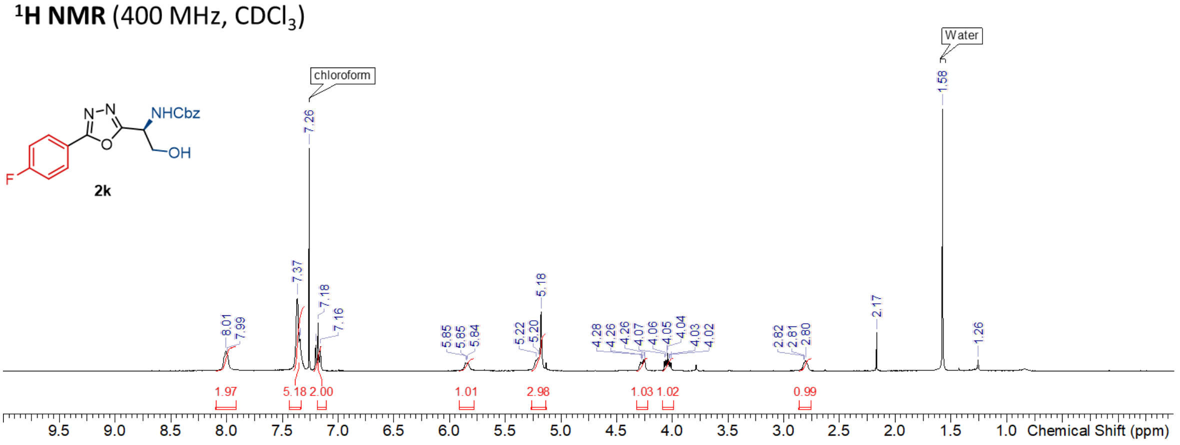

Figure S32. ^{1}H NMR spectrum (400 MHz, $CDCl_3$, 298 K) of **2k**.

^{13}C NMR (101 MHz, $CDCl_3$)

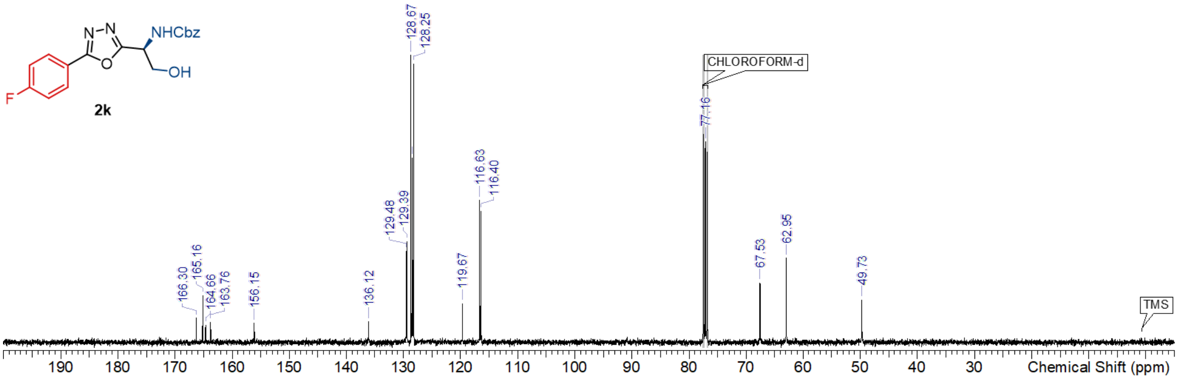

Figure S33. ^{13}C NMR spectrum (101 MHz, $CDCl_3$, 298 K) of **2k**.

^{19}F NMR (376 MHz, $CDCl_3$)

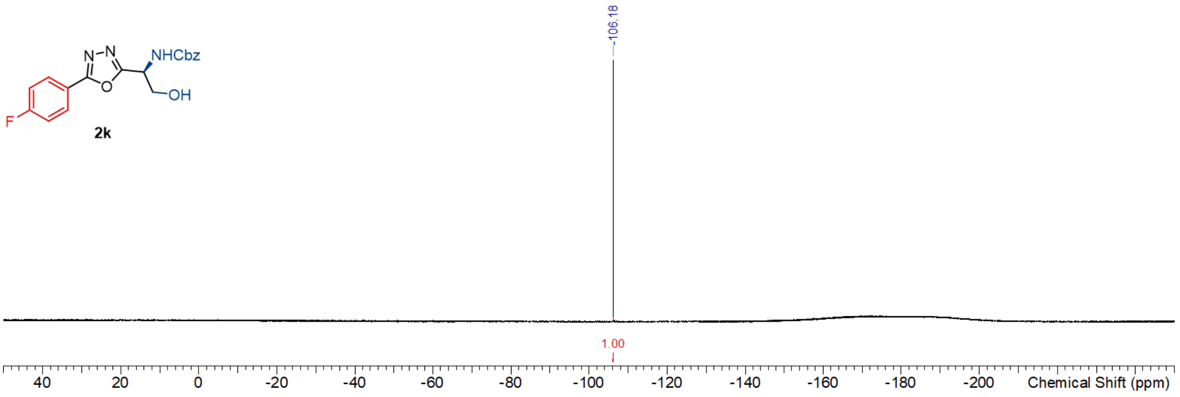

Figure S34. ^{19}F NMR spectrum (376 MHz, $CDCl_3$, 298 K) of **2k**.

^{1}H NMR (400 MHz, $CDCl_3$)

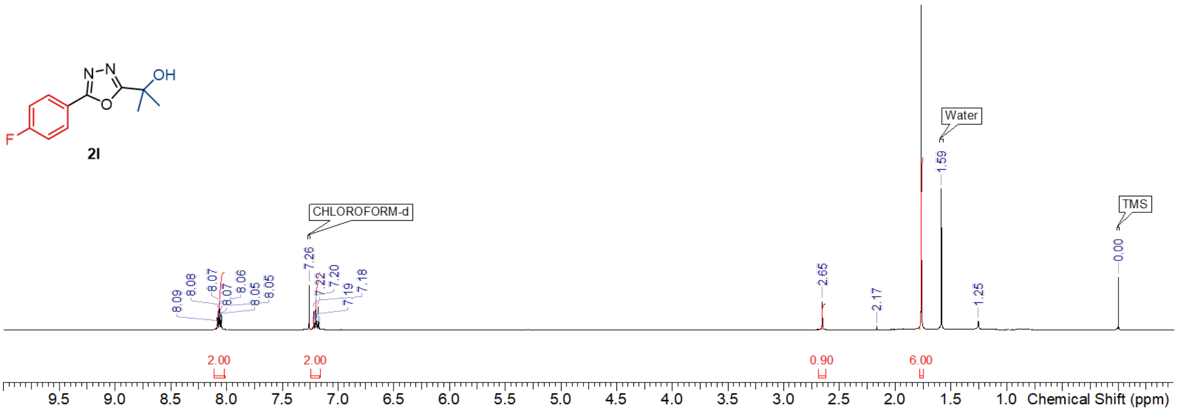

Figure S35. ^{1}H NMR spectrum (400 MHz, $CDCl_3$, 298 K) of **2l**.

^{13}C NMR (101 MHz, $CDCl_3$)

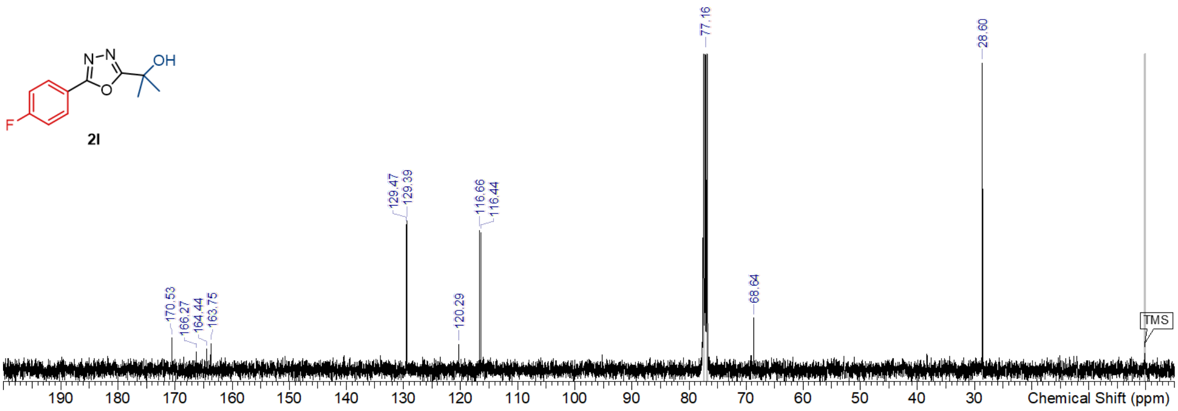

Figure S36. ^{13}C NMR spectrum (101 MHz, $CDCl_3$, 298 K) of **2l**.

^{19}F NMR (376 MHz, $CDCl_3$)

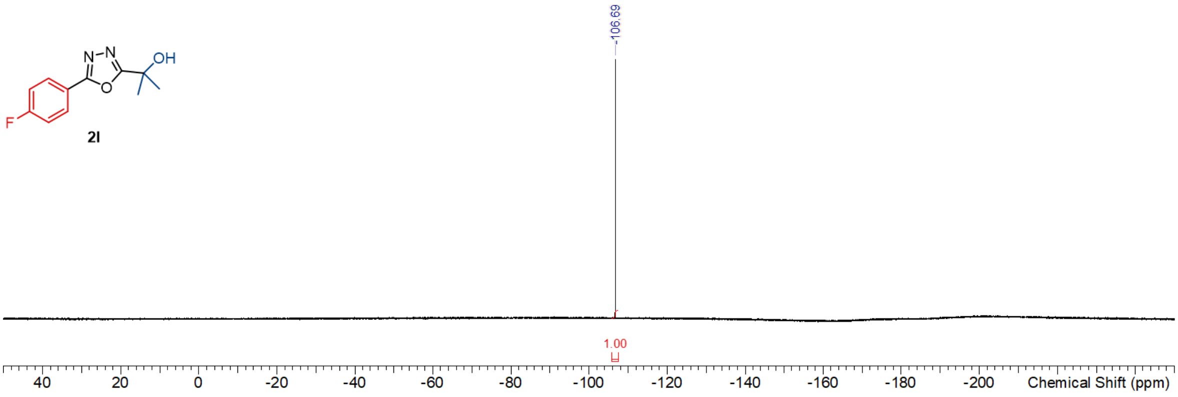

Figure S37. ^{19}F NMR spectrum (376 MHz, $CDCl_3$, 298 K) of **2l**.

^{1}H NMR (400 MHz, $CDCl_3$)

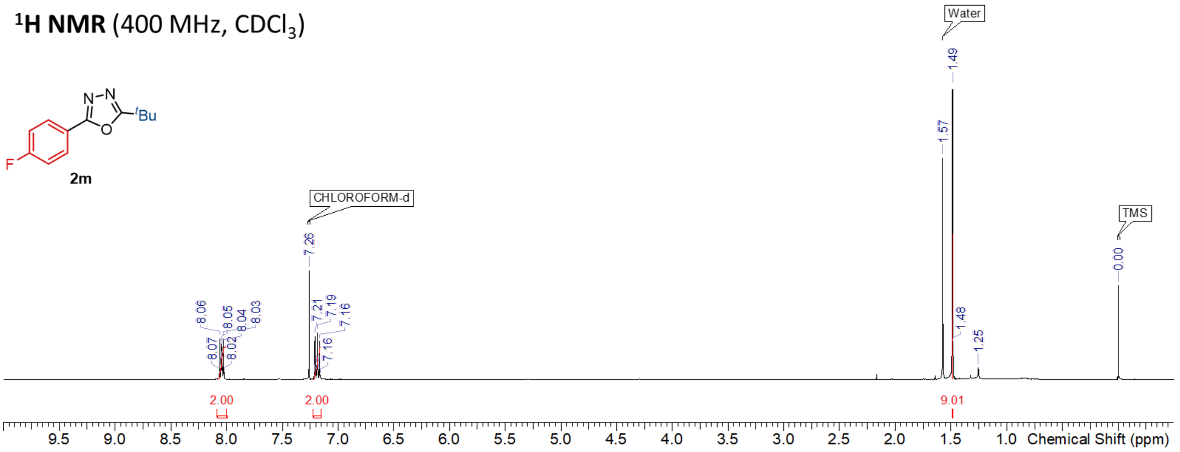

Figure S38. ^{1}H NMR spectrum (400 MHz, $CDCl_3$, 298 K) of **2m**.

^{13}C NMR (101 MHz, $CDCl_3$)

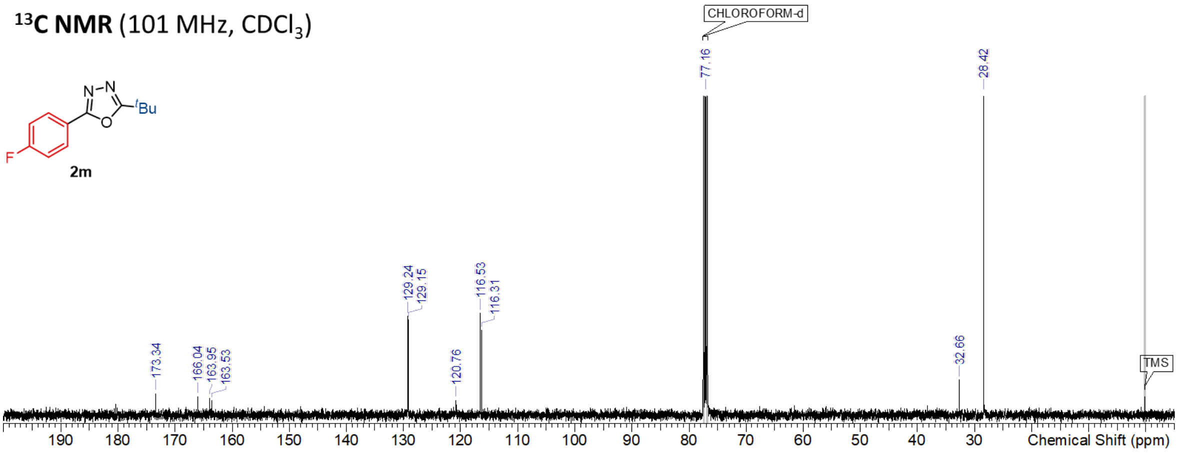

Figure S39. ^{13}C NMR spectrum (101 MHz, $CDCl_3$, 298 K) of **2m**.

^{19}F NMR (376 MHz, $CDCl_3$)

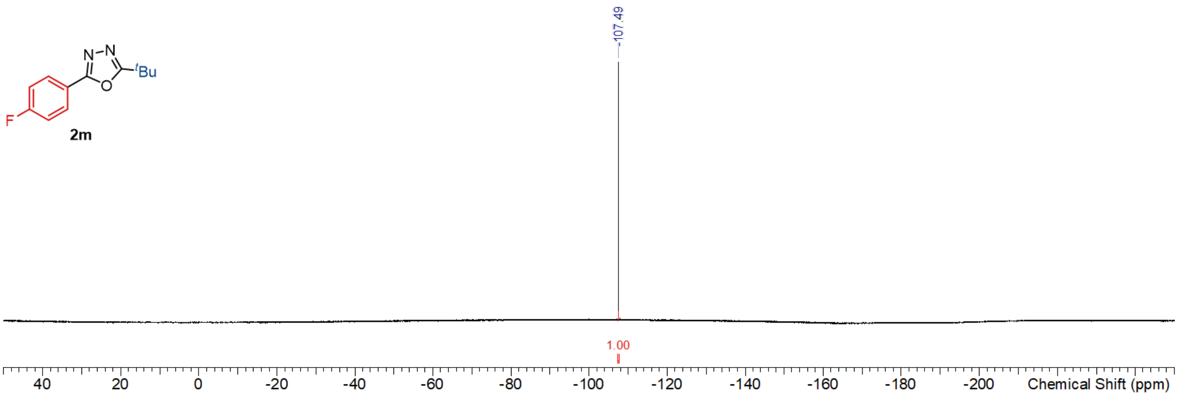

Figure S40. ^{19}F NMR spectrum (376 MHz, $CDCl_3$, 298 K) of **2m**.

^{1}**H NMR** (400 MHz, $CDCl_3$)

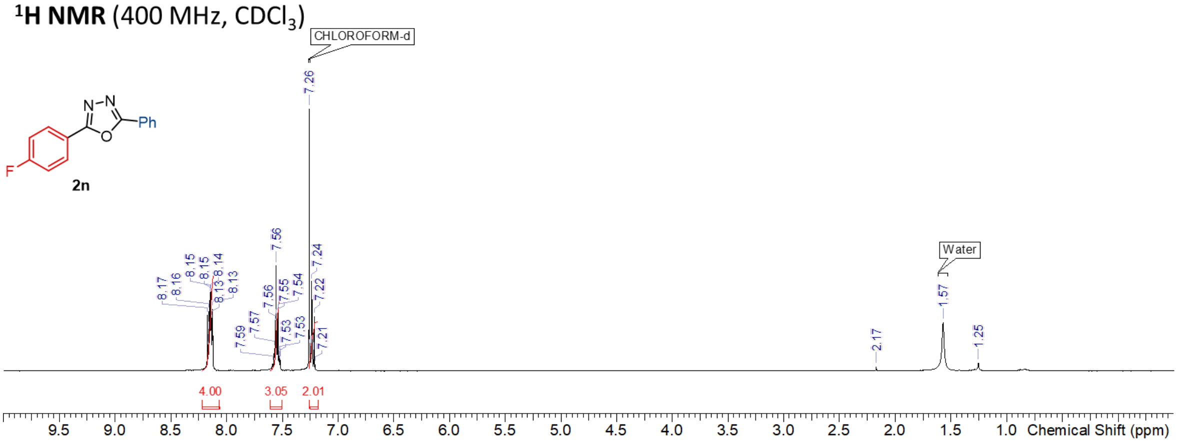

Figure S41. ^{1}H NMR spectrum (400 MHz, $CDCl_3$, 298 K) of **2n**.

^{13}**C NMR** (101 MHz, $CDCl_3$)

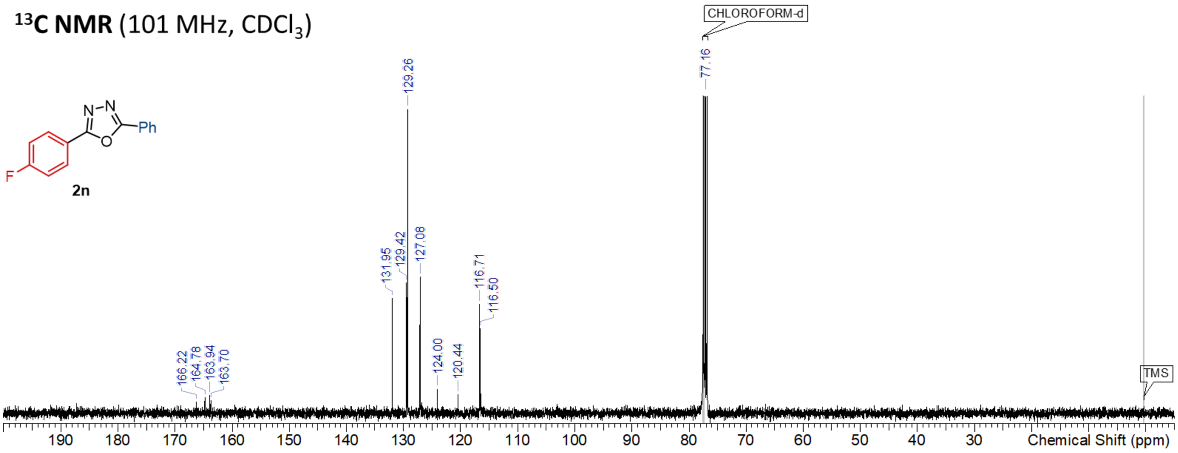

Figure S42. ^{13}C NMR spectrum (101 MHz, $CDCl_3$, 298 K) of **2n**.

^{19}**F NMR** (376 MHz, $CDCl_3$)

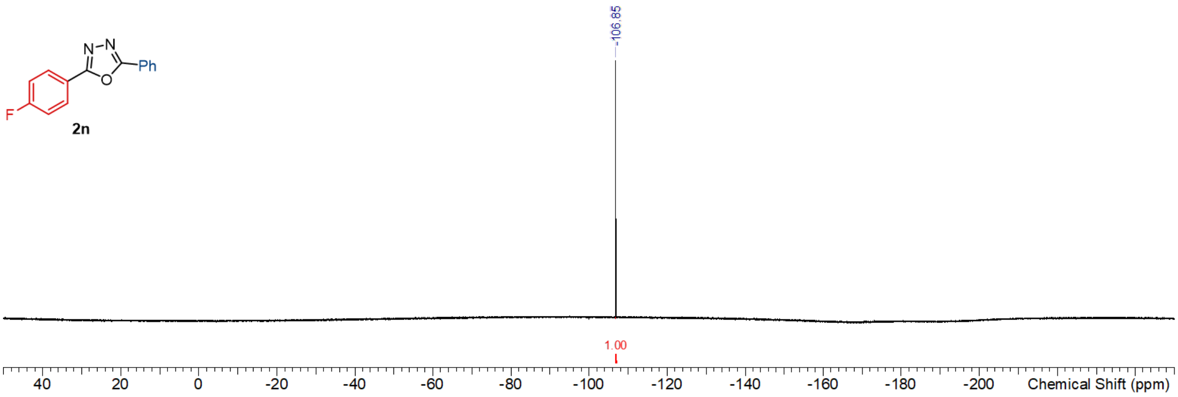

Figure S43. ^{19}F NMR spectrum (376 MHz, $CDCl_3$, 298 K) of **2n**.

^{1}H NMR (400 MHz, $CDCl_3$)

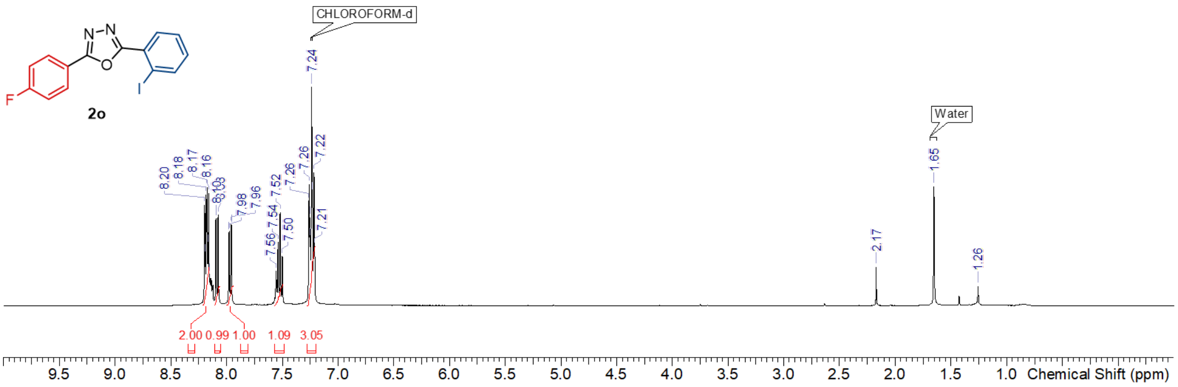

Figure S44. ^{1}H NMR spectrum (400 MHz, $CDCl_3$, 298 K) of **2o**.

^{13}C NMR (101 MHz, $CDCl_3$)

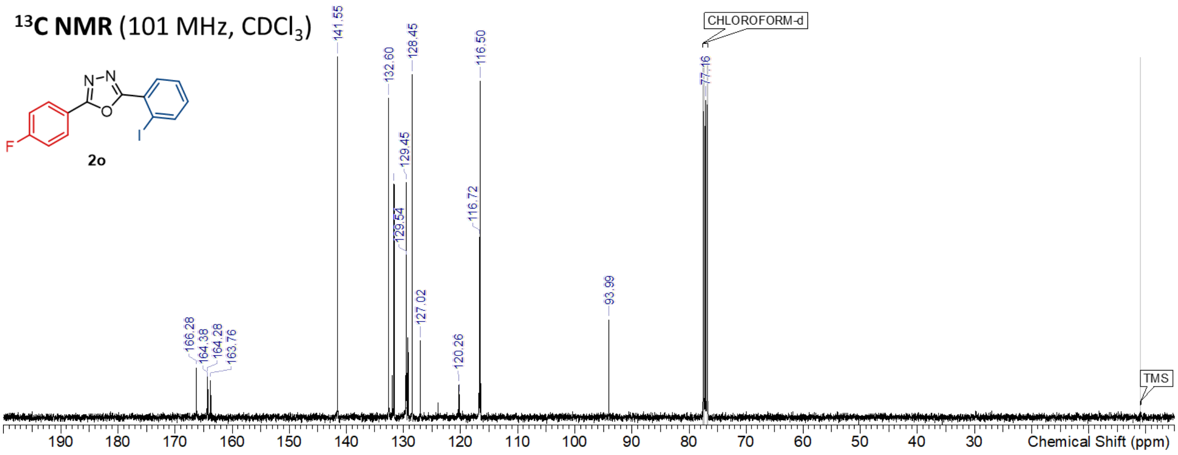

Figure S45. ^{13}C NMR spectrum (101 MHz, $CDCl_3$, 298 K) of **2o**.

^{19}F NMR (376 MHz, $CDCl_3$)

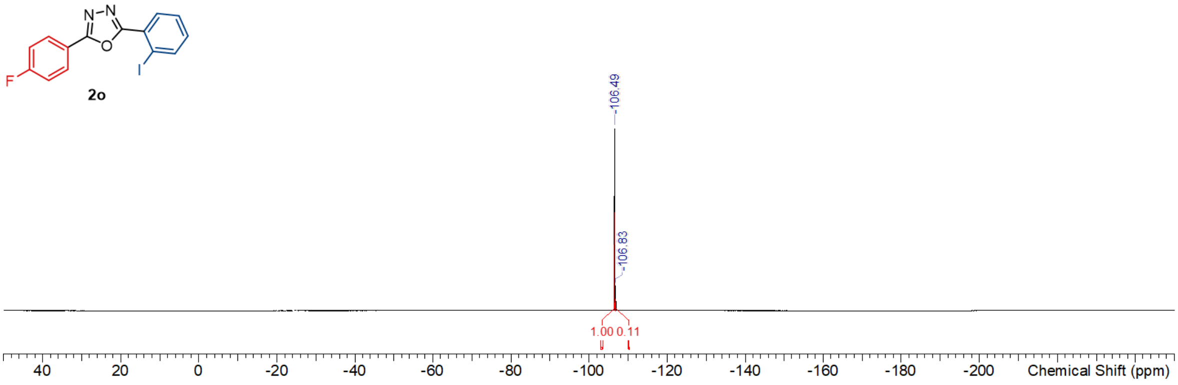

Figure S46. ^{19}F NMR spectrum (376 MHz, $CDCl_3$, 298 K) of **2o**.

^{1}H NMR (400 MHz, $CDCl_3$)

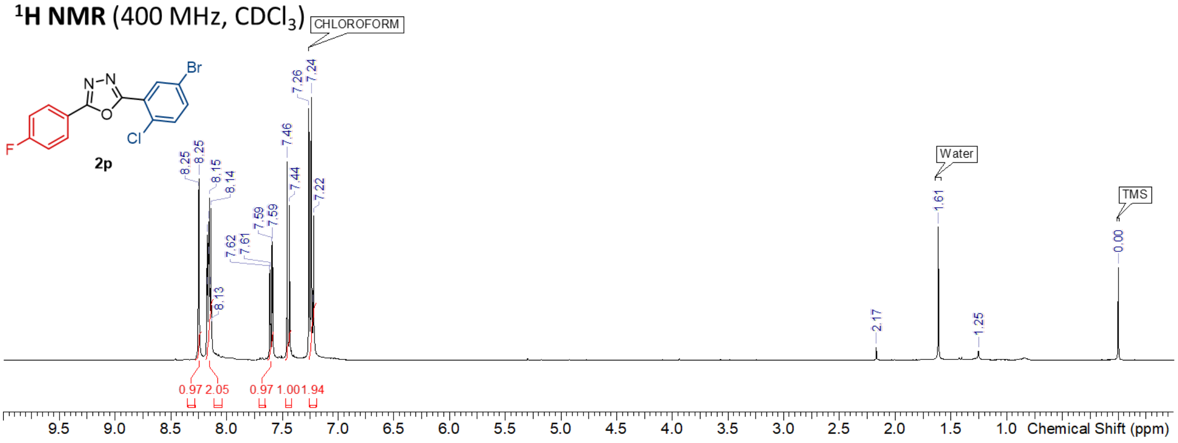

Figure S47. ^{1}H NMR spectrum (400 MHz, $CDCl_3$, 298 K) of **2p**.

^{13}C NMR (101 MHz, $CDCl_3$)

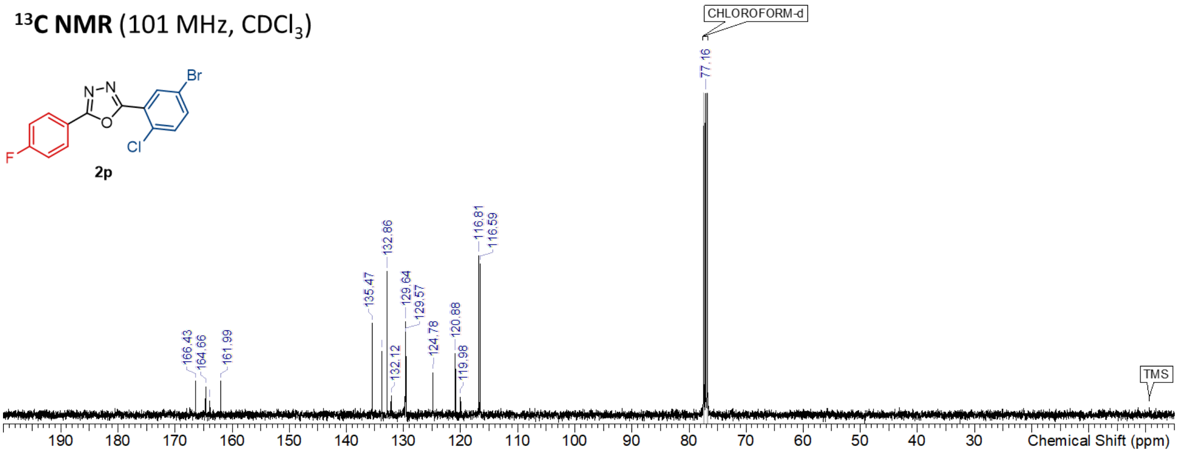

Figure S48. ^{13}C NMR spectrum (101 MHz, $CDCl_3$, 298 K) of **2p**.

^{19}F NMR (376 MHz, $CDCl_3$)

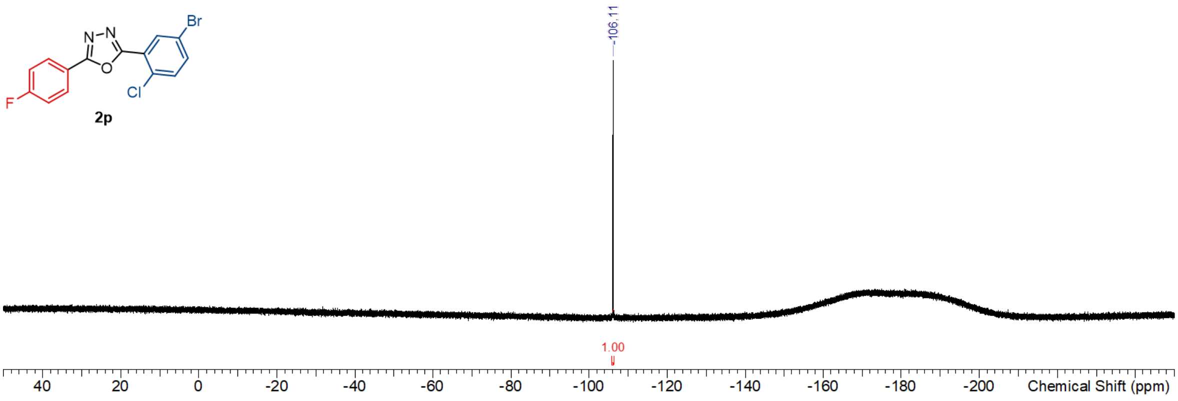

Figure S49. ^{19}F NMR spectrum (376 MHz, $CDCl_3$, 298 K) of **2p**.

^{1}H NMR (400 MHz, DMSO-d_6)

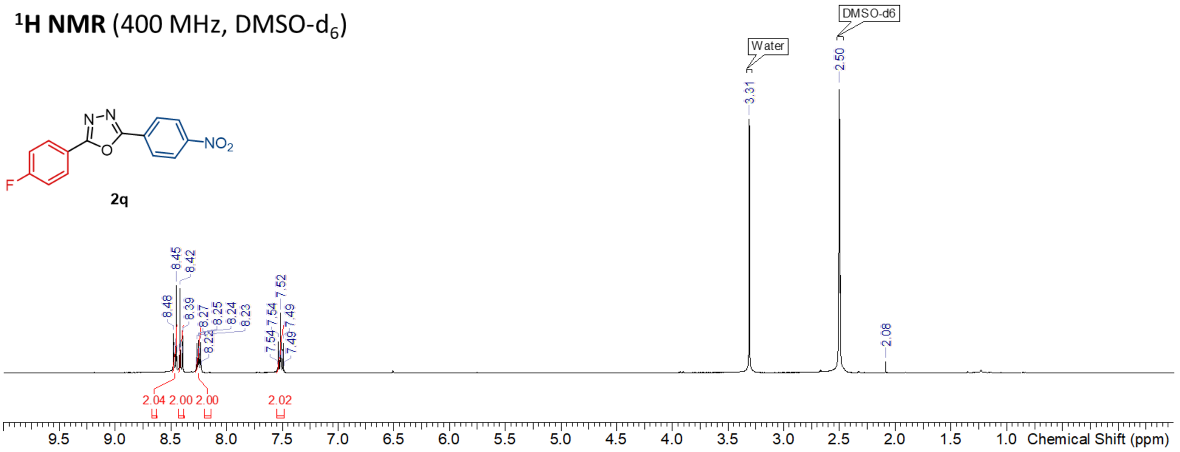

Figure S50. ^{1}H NMR spectrum (400 MHz, DMSO-d_6, 298 K) of **2q**.

^{13}C NMR (101 MHz, DMSO-d_6)

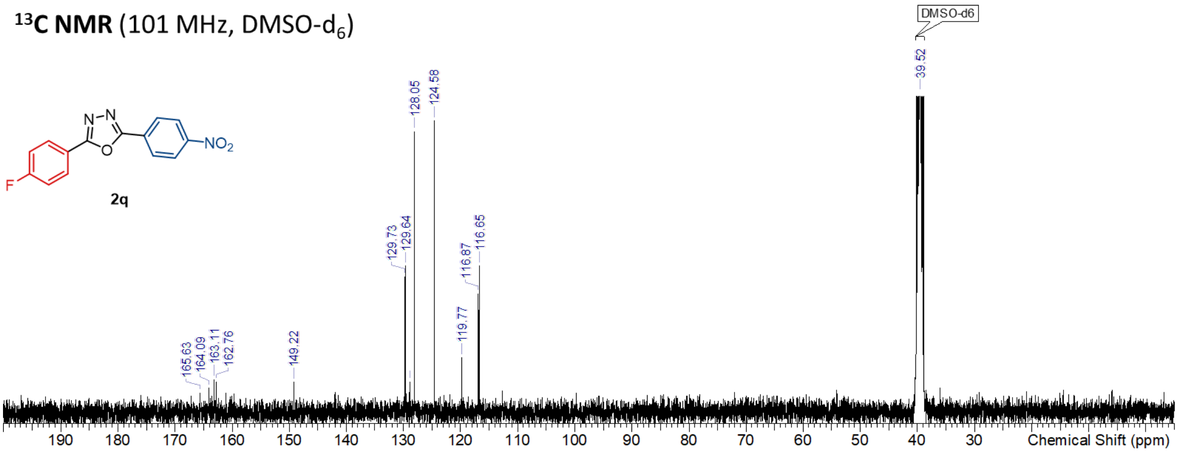

Figure S51. ^{13}C NMR spectrum (101 MHz, DMSO-d_6, 298 K) of **2q**.

^{19}F NMR (376 MHz, DMSO-d_6)

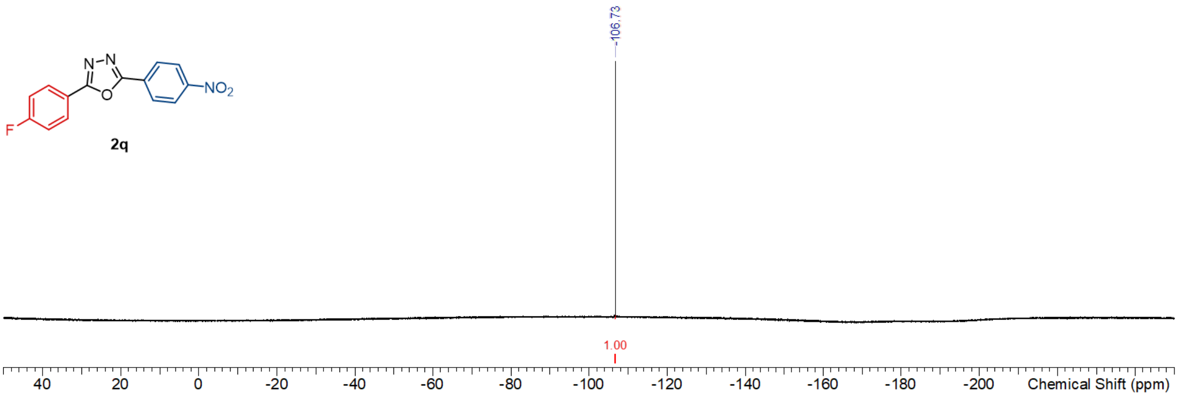

Figure S52. ^{19}F NMR spectrum (376 MHz, DMSO-d_6, 298 K) of **2q**.

^{1}**H NMR** (400 MHz, $CDCl_3$)

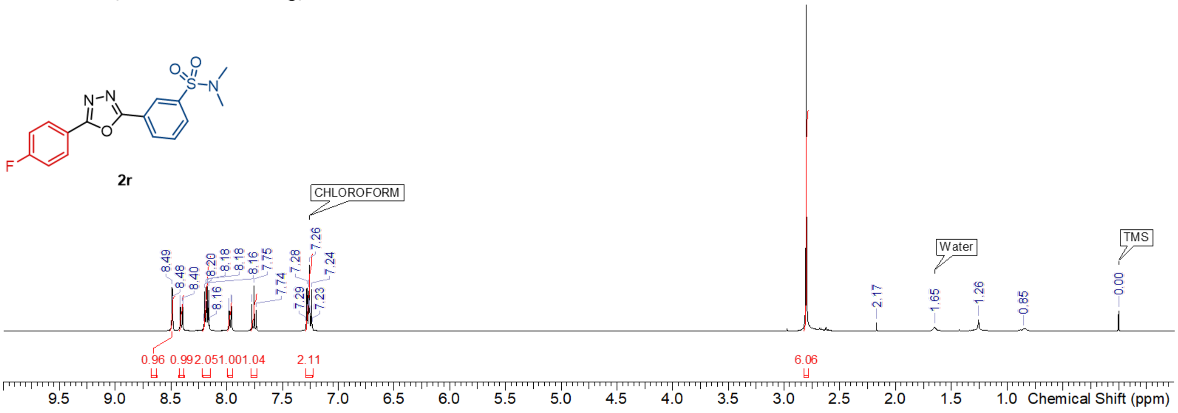

Figure S53. ^{1}H NMR spectrum (400 MHz, $CDCl_3$, 298 K) of **2r**.

^{13}**C NMR** (101 MHz, $CDCl_3$)

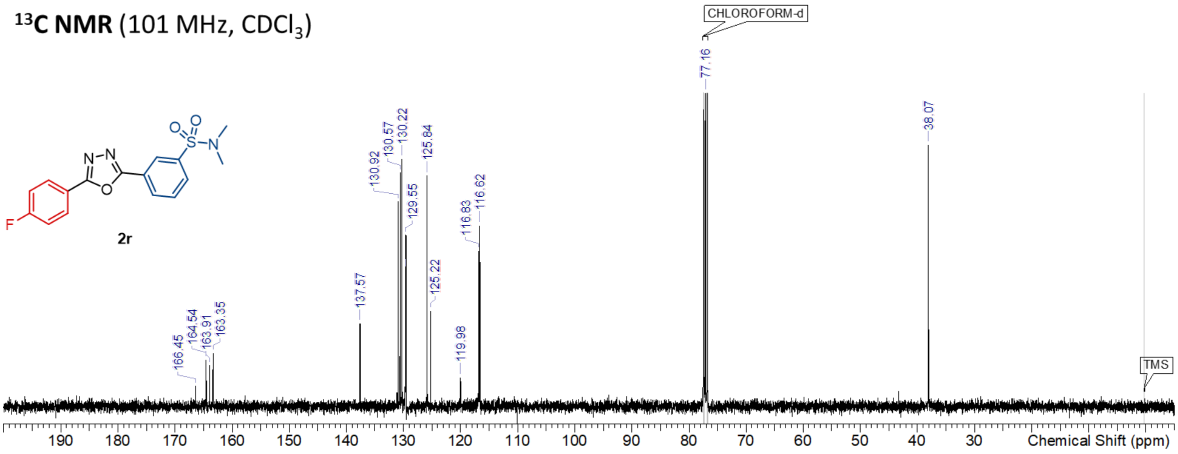

Figure S54. ^{13}C NMR spectrum (101 MHz, $CDCl_3$, 298 K) of **2r**.

^{19}**F NMR** (376 MHz, $CDCl_3$)

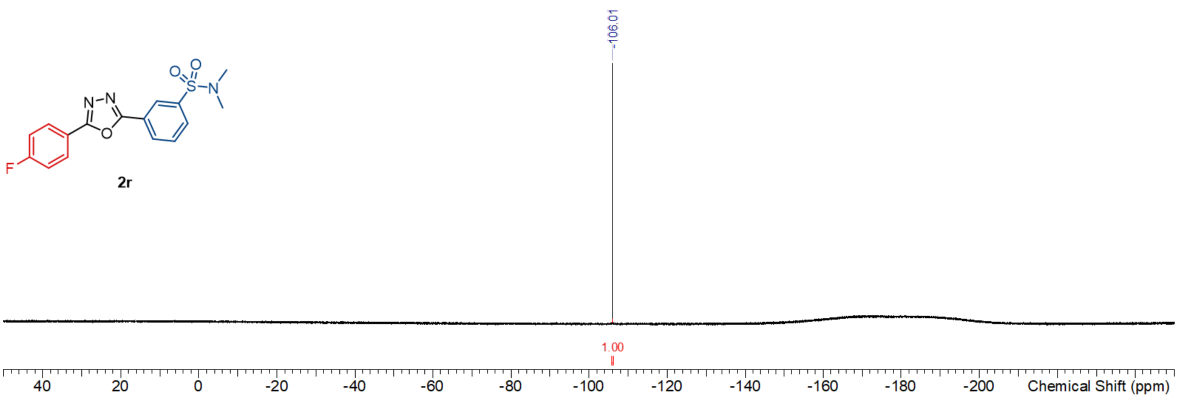

Figure S55. ^{19}F NMR spectrum (376 MHz, $CDCl_3$, 298 K) of **2r**.

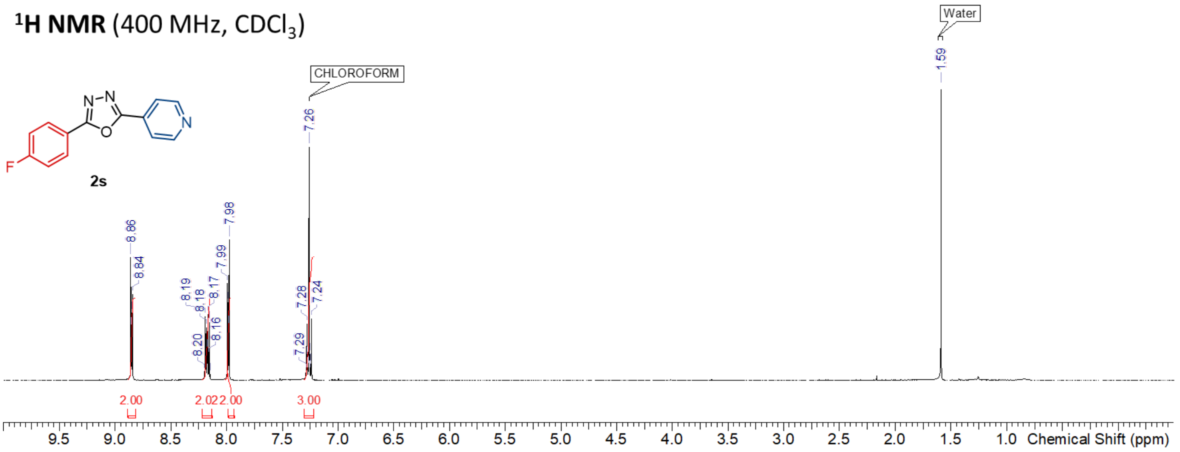

Figure S56. ^{1}H NMR spectrum (400 MHz, $CDCl_3$, 298 K) of **2s**.

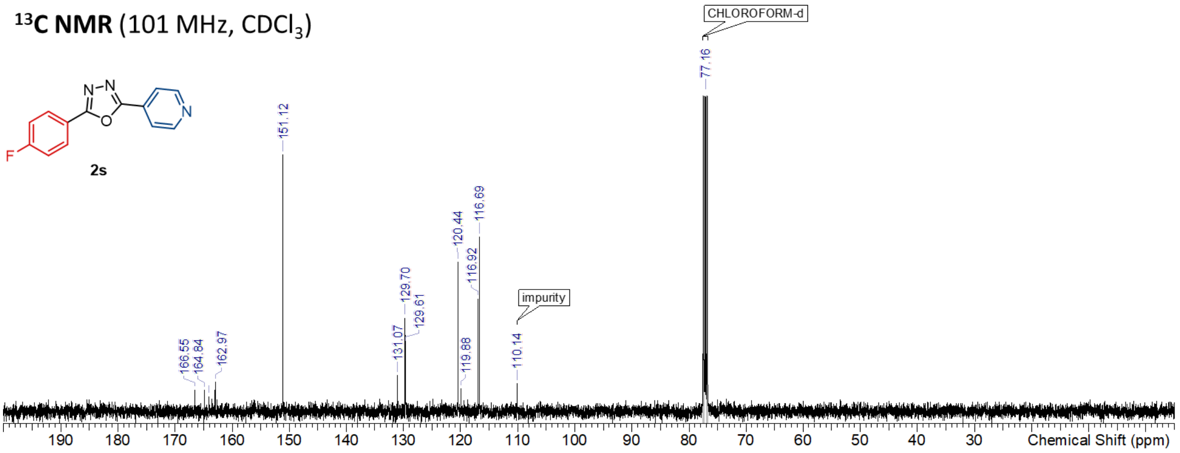

Figure S57. ^{13}C NMR spectrum (101 MHz, $CDCl_3$, 298 K) of **2s**.

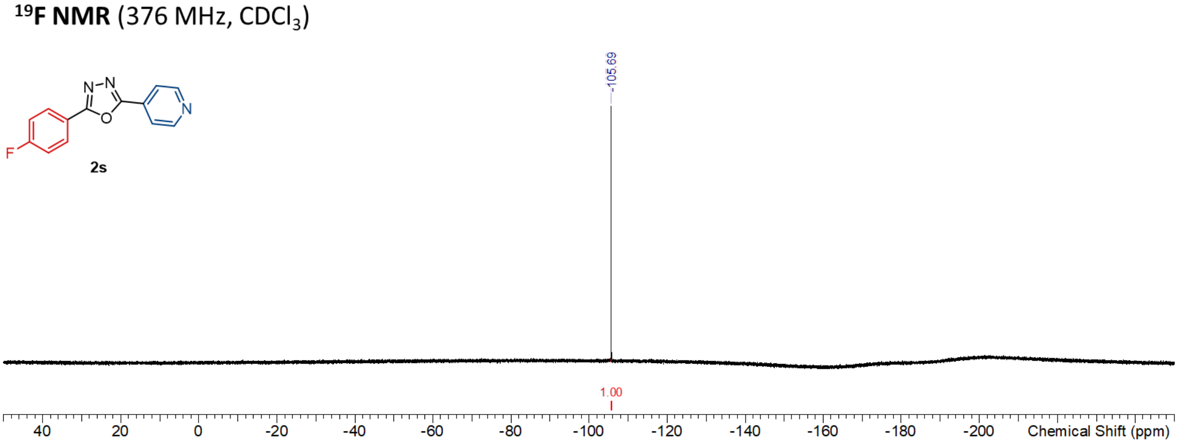

Figure S58. ^{19}F NMR spectrum (376 MHz, $CDCl_3$, 298 K) of **2s**.

¹H NMR (400 MHz, DMSO-d_6)

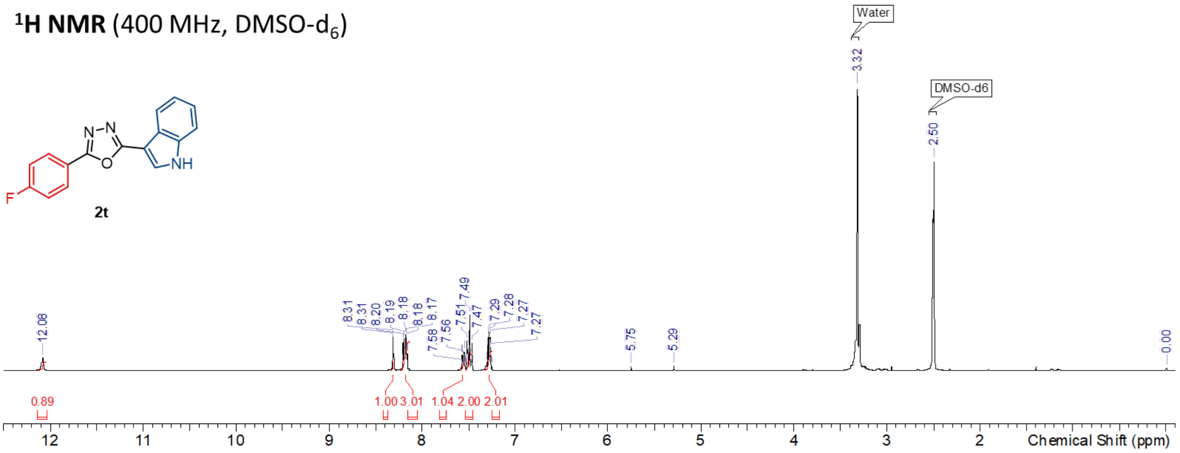

Figure S59. ^{1}H NMR spectrum (400 MHz, DMSO-d_6, 298 K) of **2t**.

¹³C NMR (101 MHz, DMSO-d_6)

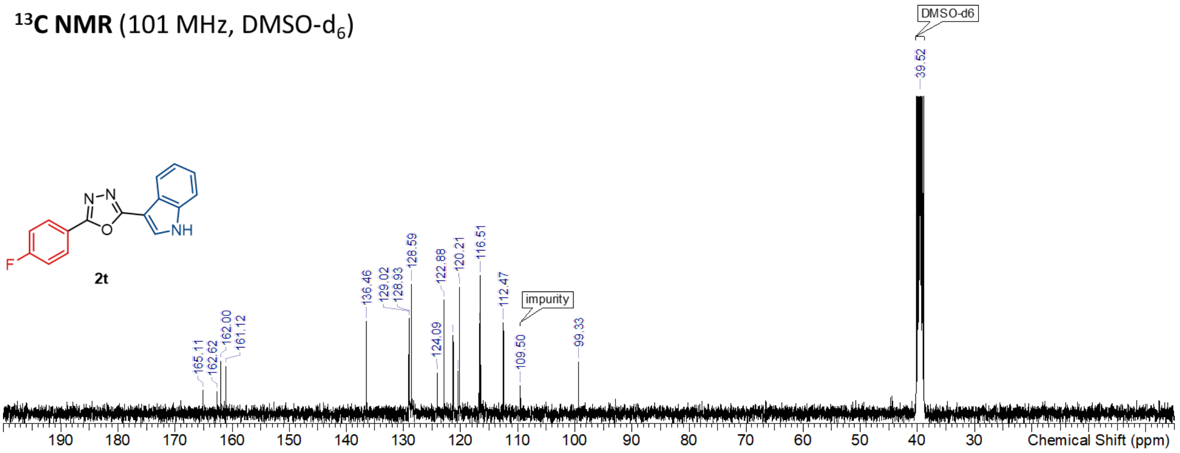

Figure S60. ^{13}C NMR spectrum (101 MHz, DMSO-d_6, 298 K) of **2t**.

¹⁹F NMR (376 MHz, DMSO-d_6)

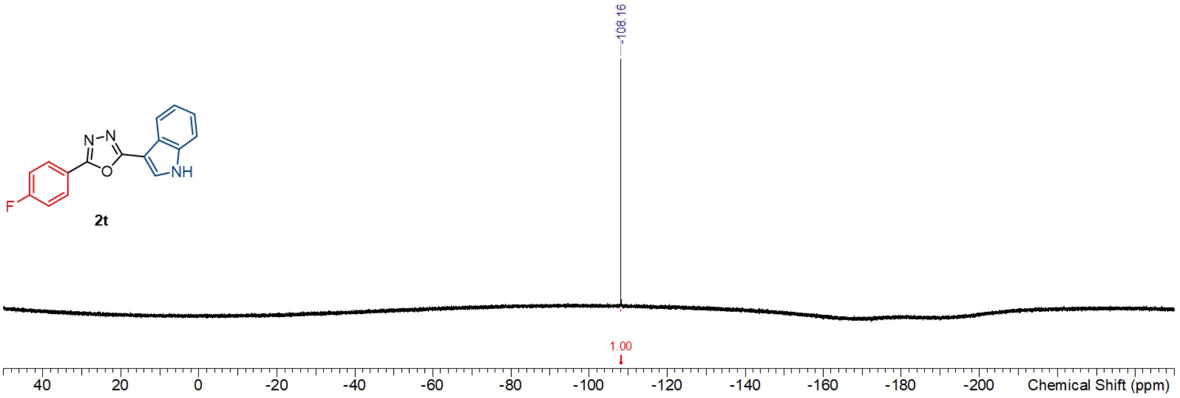

Figure S61. ^{19}F NMR spectrum (376 MHz, DMSO-d_6, 298 K) of **2t**.

^{1}H NMR (400 MHz, $CDCl_3$)

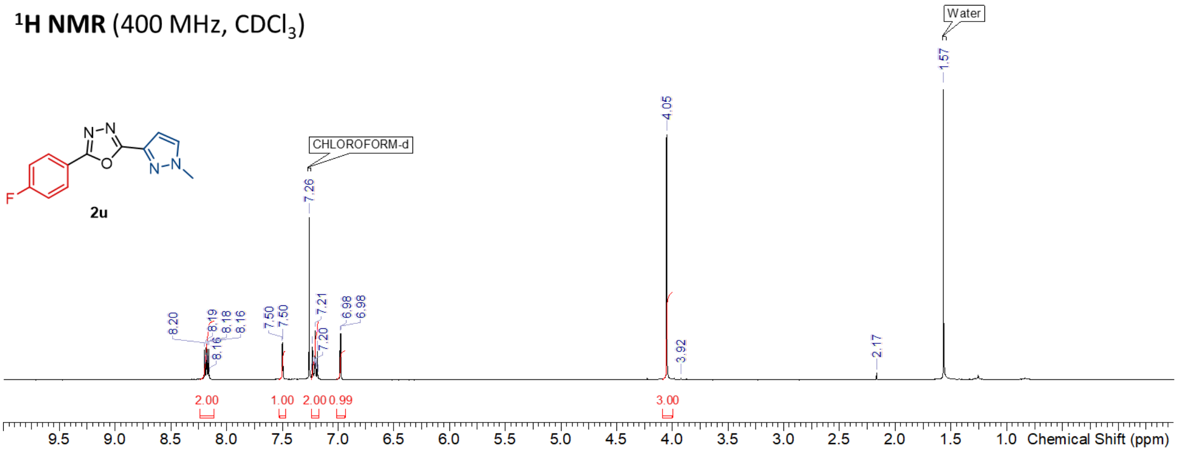

Figure S62. ^{1}H NMR spectrum (400 MHz, $CDCl_3$, 298 K) of **2u**.

^{13}C NMR (101 MHz, $CDCl_3$)

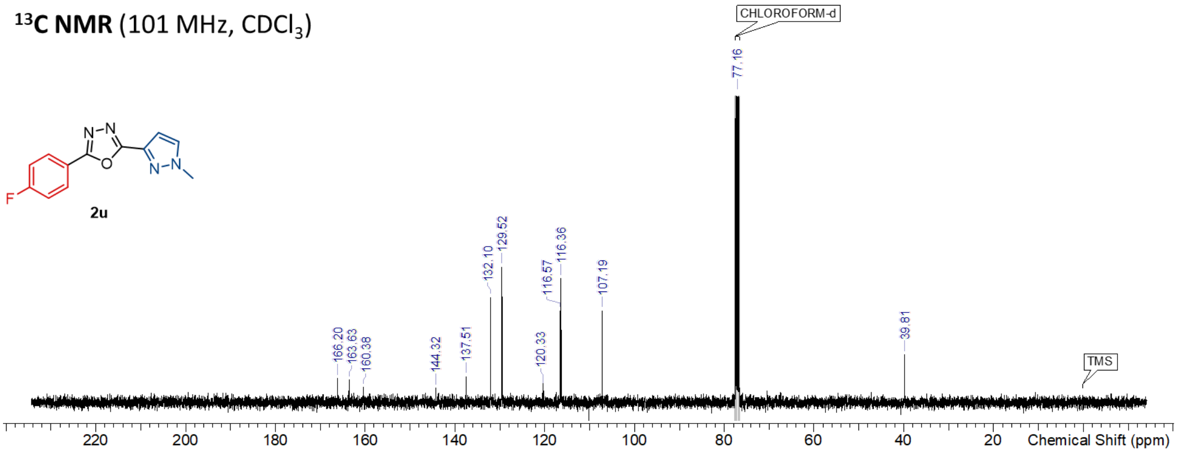

Figure S63. ^{13}C NMR spectrum (101 MHz, $CDCl_3$, 298 K) of **2u**.

^{19}F NMR (376 MHz, $CDCl_3$)

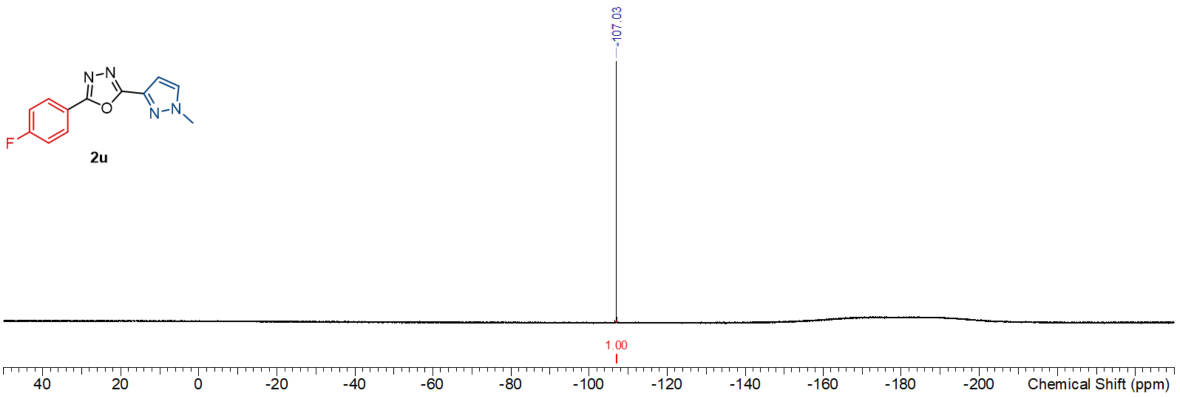

Figure S64. ^{19}F NMR spectrum (376 MHz, $CDCl_3$, 298 K) of **2u**.

[1]**H NMR** (400 MHz, $CDCl_3$)

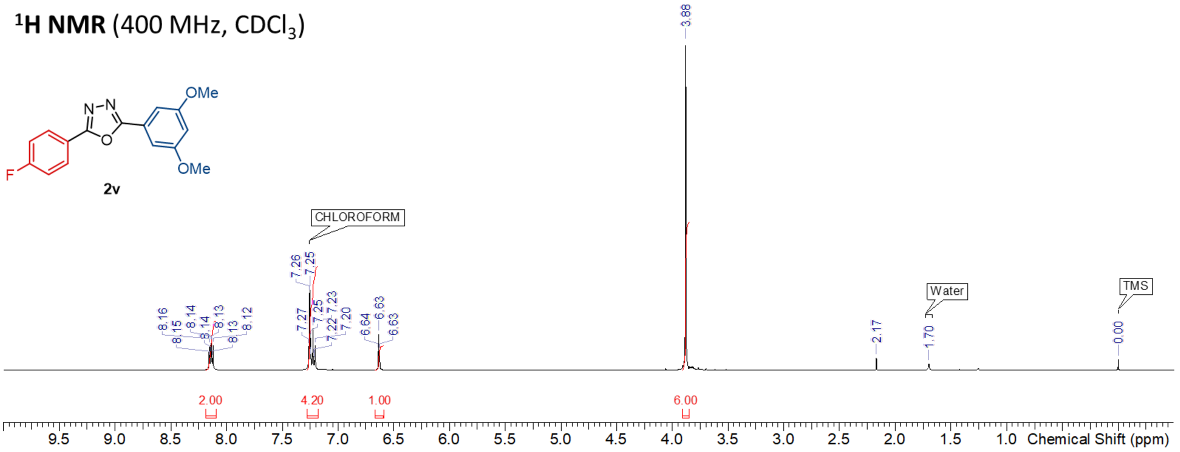

Figure S65. ^{1}H NMR spectrum (400 MHz, $CDCl_3$, 298 K) of **2v**.

[13]**C NMR** (101 MHz, $CDCl_3$)

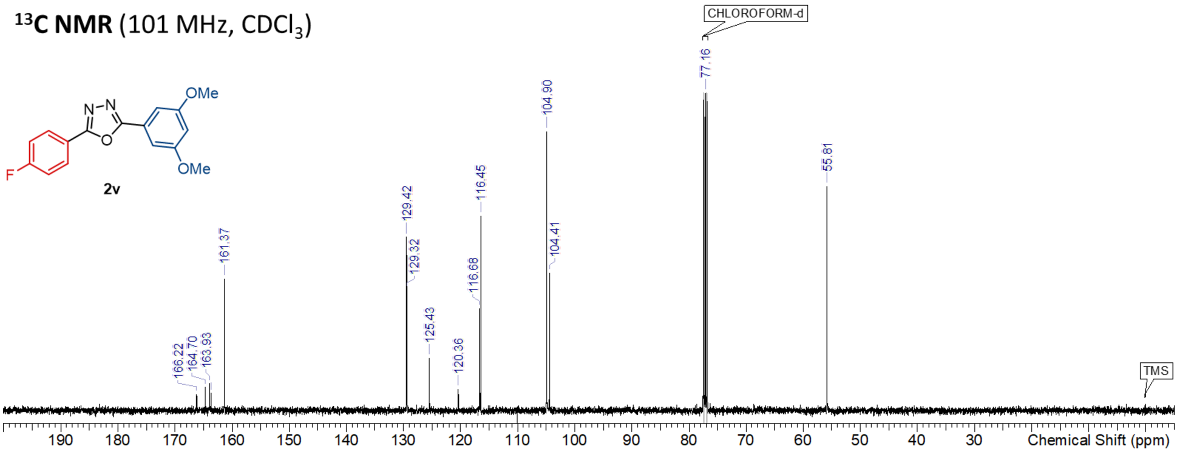

Figure S66. ^{13}C NMR spectrum (101 MHz, $CDCl_3$, 298 K) of **2v**.

[19]**F NMR** (376 MHz, $CDCl_3$)

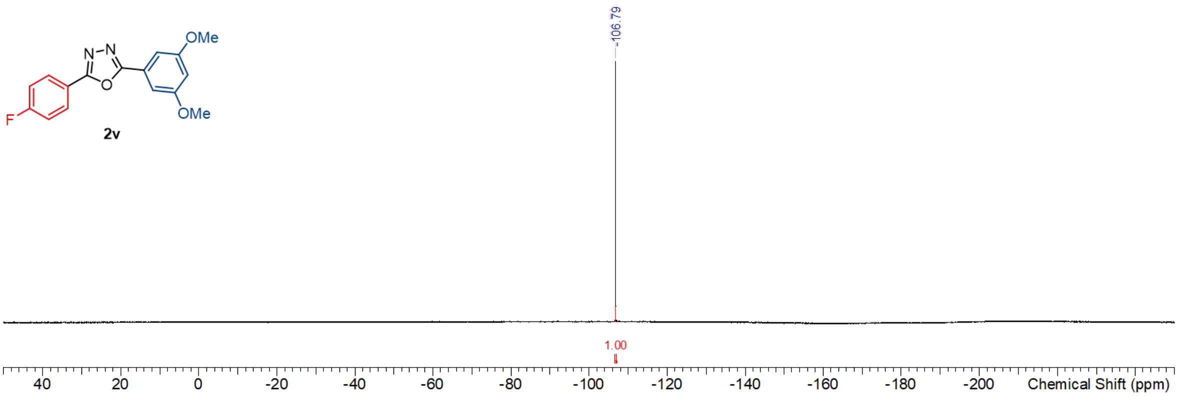

Figure S67. ^{19}F NMR spectrum (376 MHz, $CDCl_3$, 298 K) of **2v**.

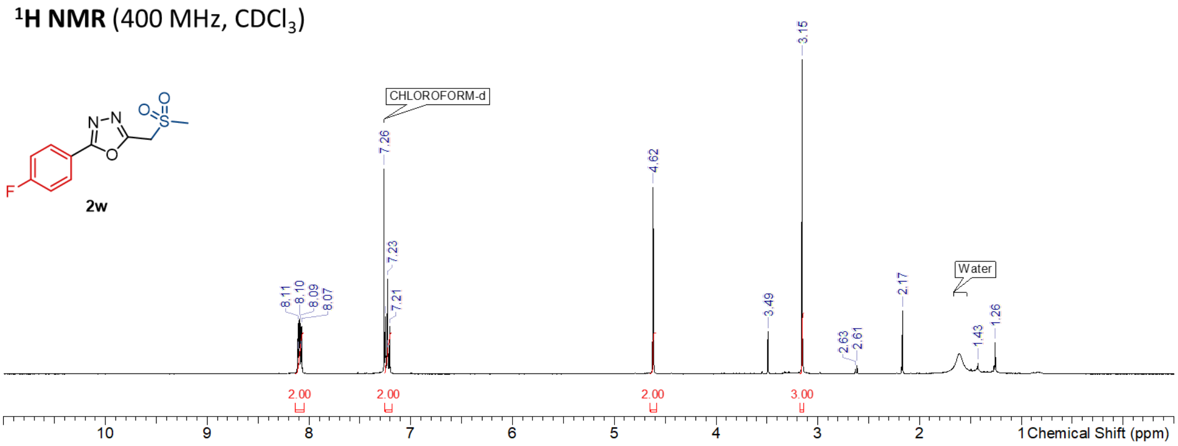

Figure S68. ^{1}H NMR spectrum (400 MHz, CDCl$_3$, 298 K) of **2w**.

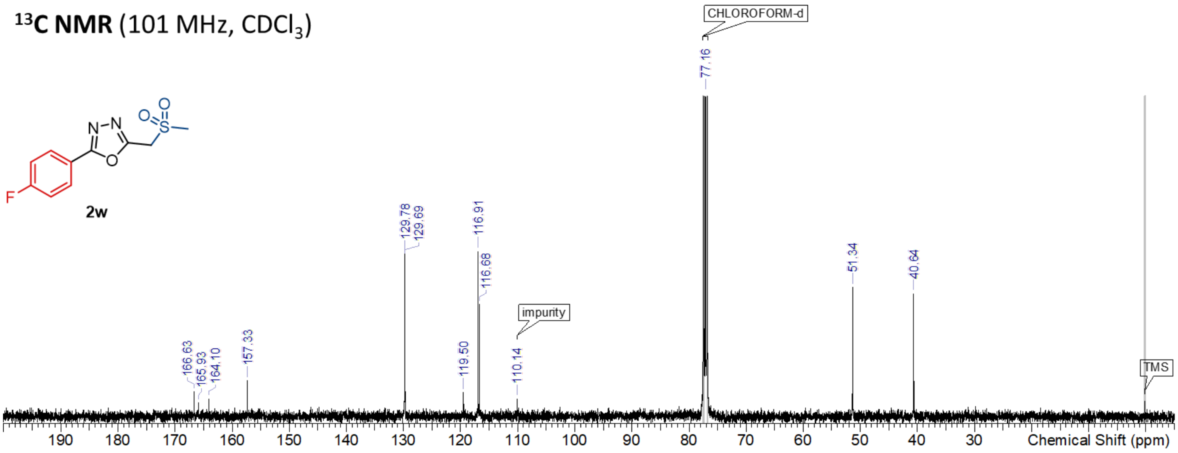

Figure S69. ^{13}C NMR spectrum (101 MHz, CDCl$_3$, 298 K) of **2w**.

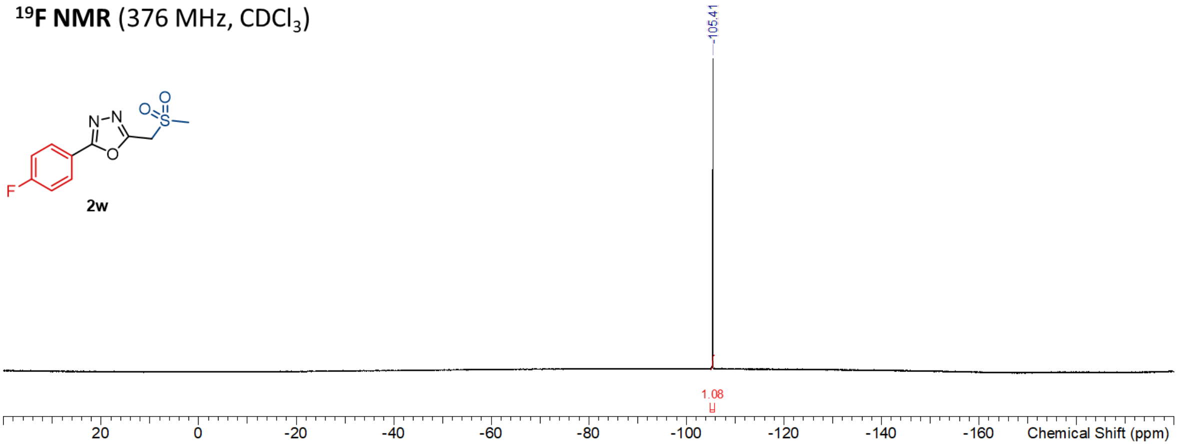

Figure S70. ^{19}F NMR spectrum (376 MHz, CDCl$_3$, 298 K) of **2w**.

^{1}**H NMR** (400 MHz, $CDCl_3$)

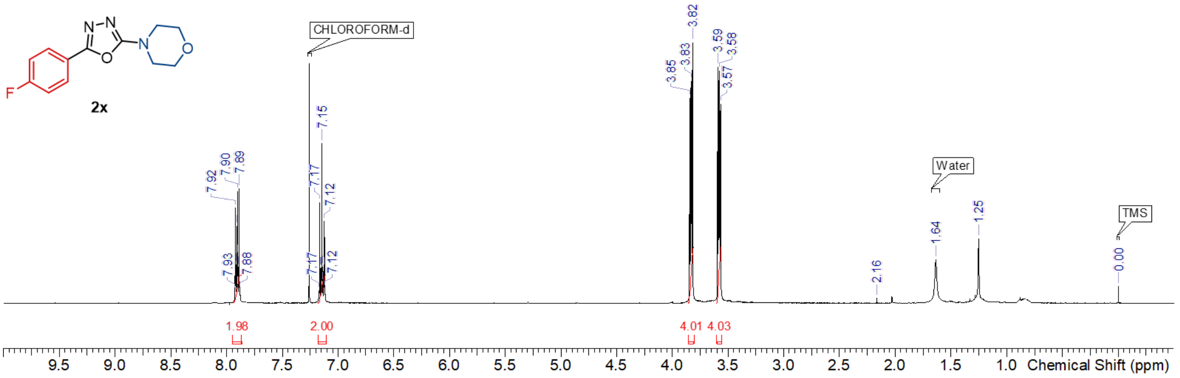

Figure S71. ^{1}H NMR spectrum (400 MHz, $CDCl_3$, 298 K) of **2x**.

^{13}**C NMR** (101 MHz, $CDCl_3$)

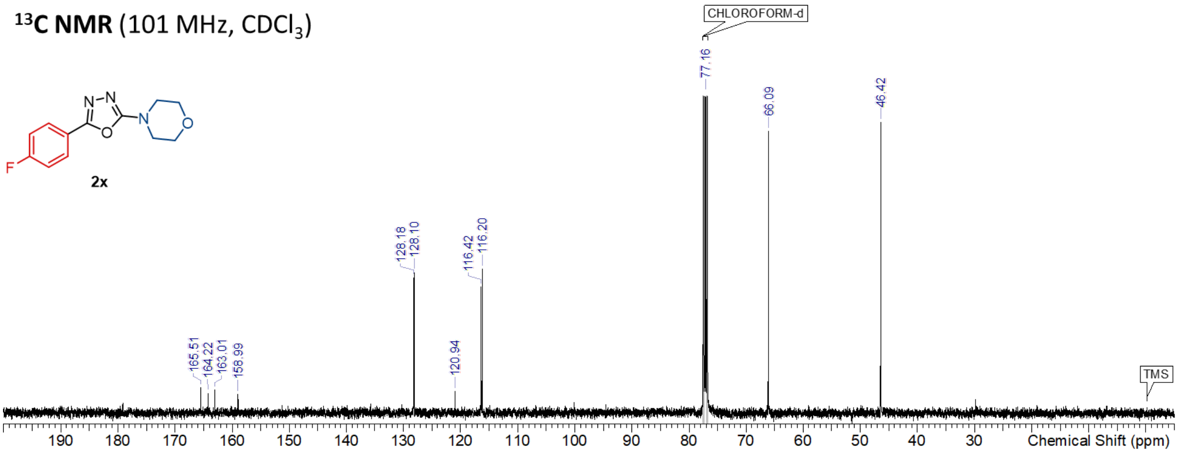

Figure S72. ^{13}C NMR spectrum (101 MHz, $CDCl_3$, 298 K) of **2x**.

^{19}**F NMR** (376 MHz, $CDCl_3$)

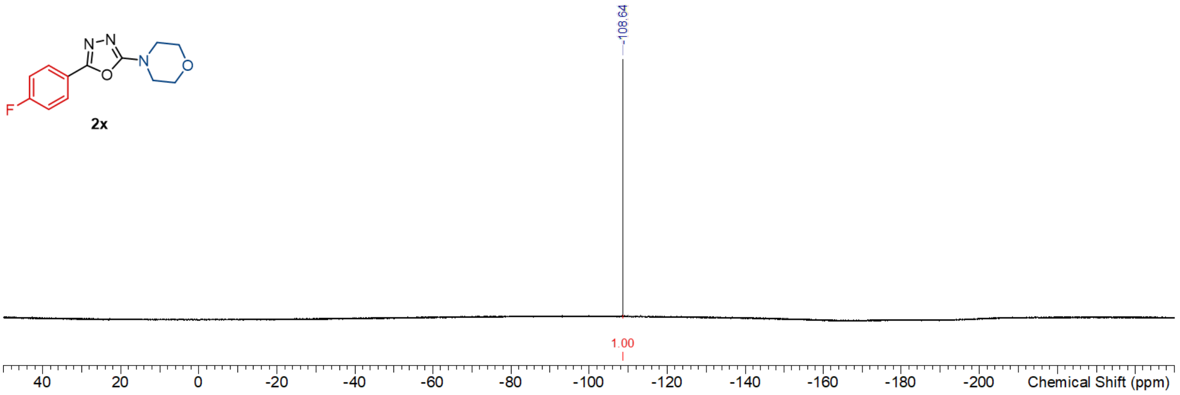

Figure S73. ^{19}F NMR spectrum (376 MHz, $CDCl_3$, 298 K) of **2x**.

^{1}H NMR (400 MHz, DMSO-d_6)

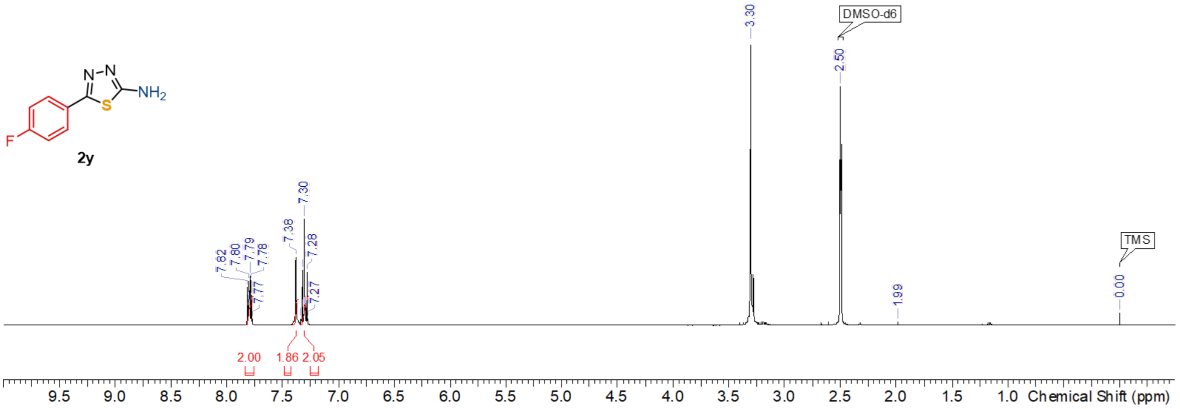

Figure S74. ^{1}H NMR spectrum (400 MHz, DMSO-d_6, 298 K) of **2y**.

^{13}C NMR (101 MHz, DMSO-d_6)

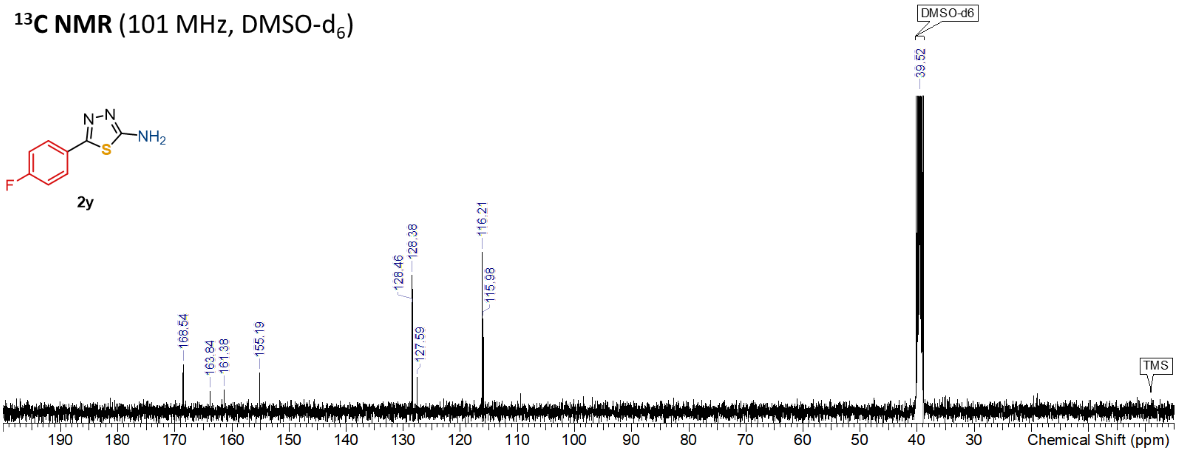

Figure S75. ^{13}C NMR spectrum (101 MHz, DMSO-d_6, 298 K) of **2y**.

^{19}F NMR (376 MHz, DMSO-d_6)

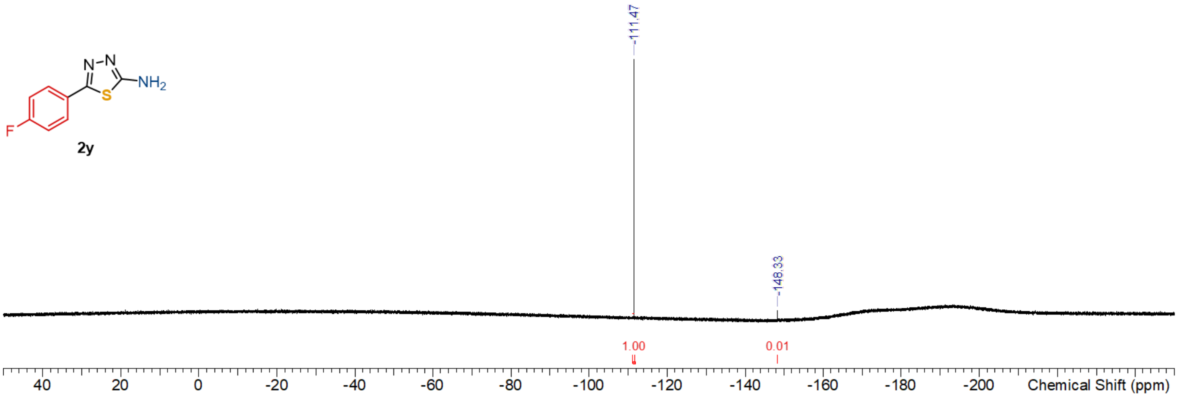

Figure S76. ^{19}F NMR spectrum (376 MHz, DMSO-d_6, 298 K) of **2y**.

^{1}**H NMR** (400 MHz, MeOD-d_4)

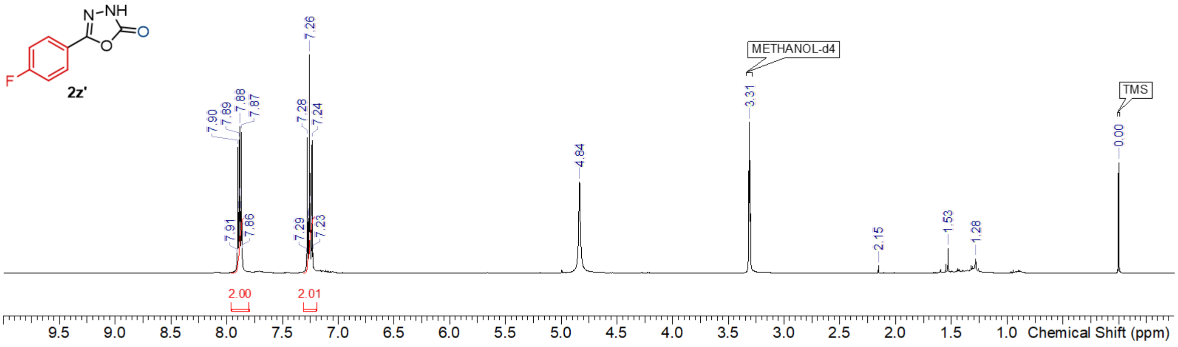

Figure S77. ^{1}H NMR spectrum (400 MHz, MeOD-d_4, 298 K) of **2z'**.

^{13}**C NMR** (101 MHz, MeOD-d_4)

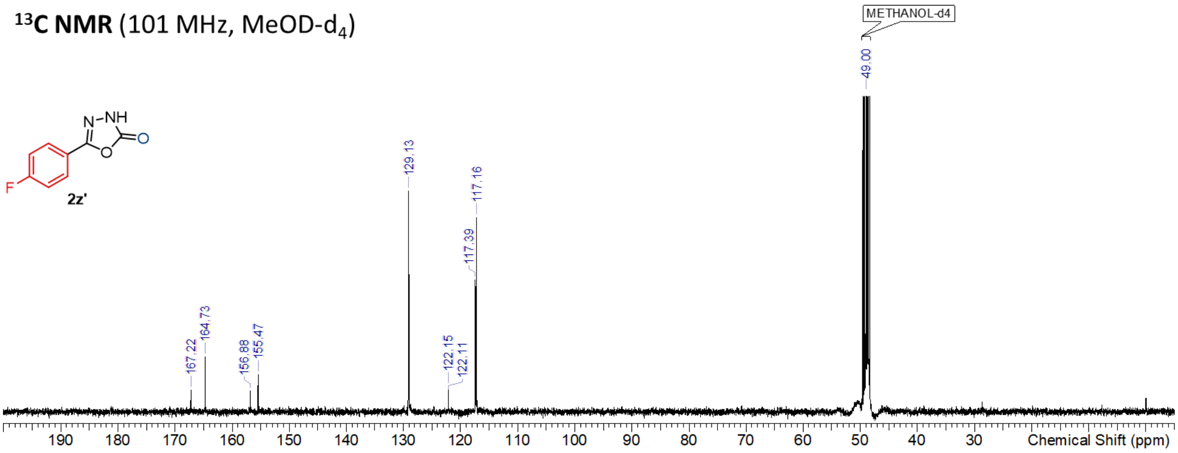

Figure S78. ^{13}C NMR spectrum (101 MHz, MeOD-d_4, 298 K) of **2z'**.

^{19}**F NMR** (376 MHz, MeOD-d_4)

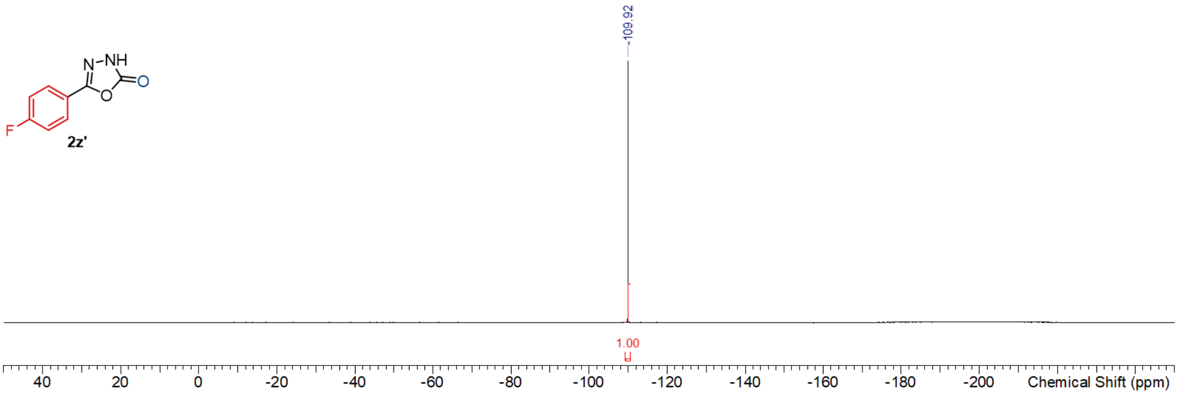

Figure S79. ^{19}F NMR spectrum (376 MHz, MeOD-d_4, 298 K) of **2z'**.

^{1}H NMR (400 MHz, $CDCl_3$)

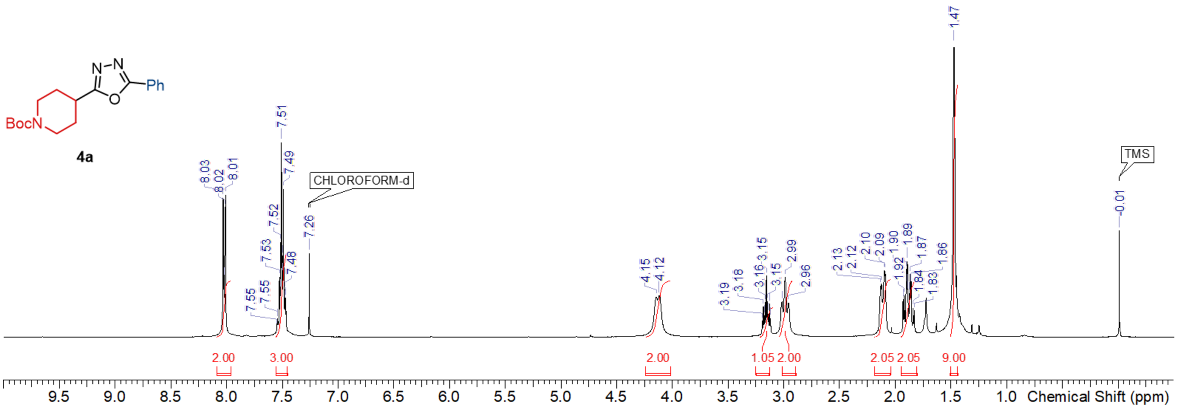

Figure S80. ^{1}H NMR spectrum (400 MHz, $CDCl_3$, 298 K) of **4a**.

^{13}C NMR (101 MHz, $CDCl_3$)

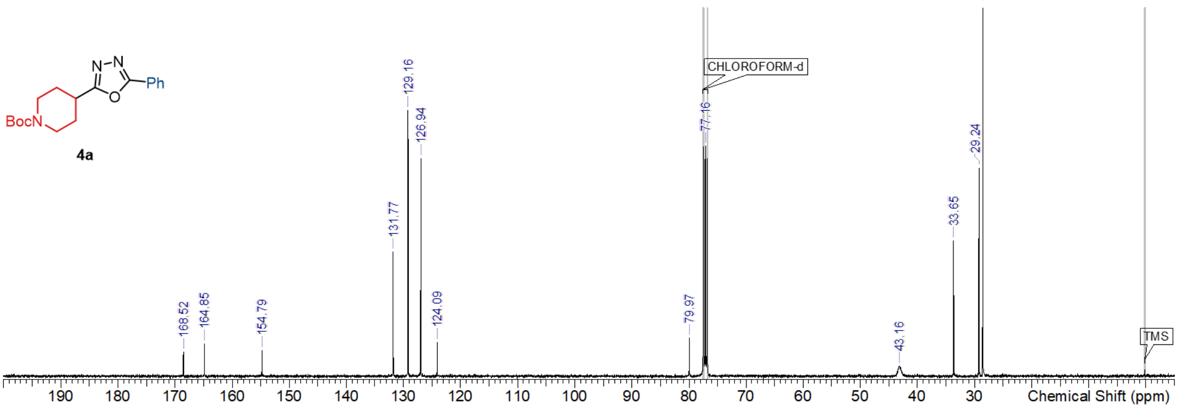

Figure S81. ^{13}C NMR spectrum (101 MHz, $CDCl_3$, 298 K) of **4a**.

^{1}H NMR (400 MHz, $CDCl_3$)

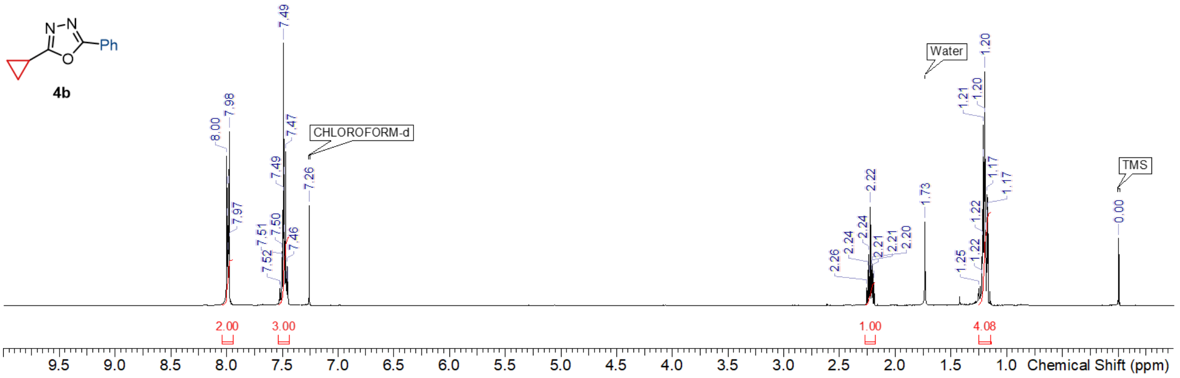

Figure S82. ^{1}H NMR spectrum (400 MHz, $CDCl_3$, 298 K) of **4b**.

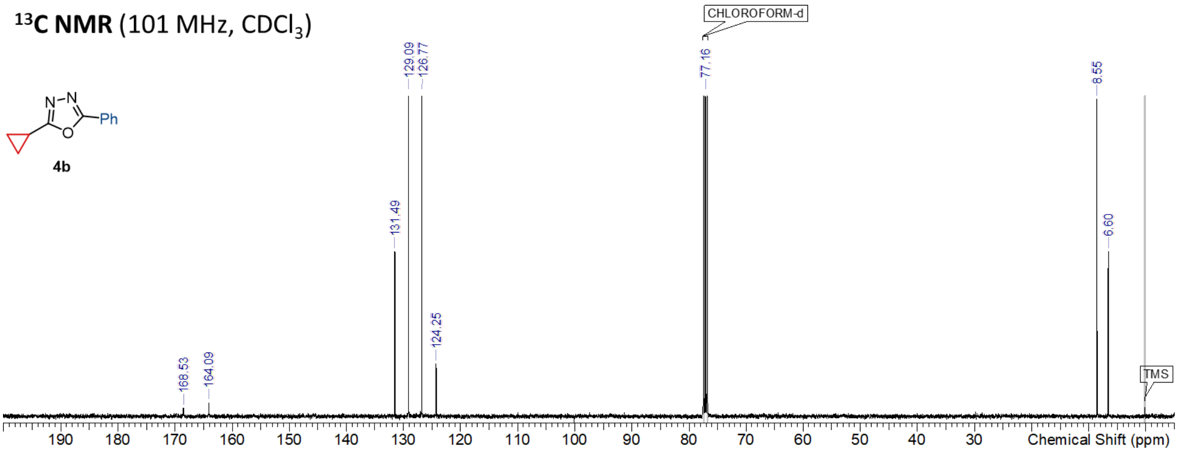

Figure S83. ^{13}C NMR spectrum (101 MHz, $CDCl_3$, 298 K) of **4b**.

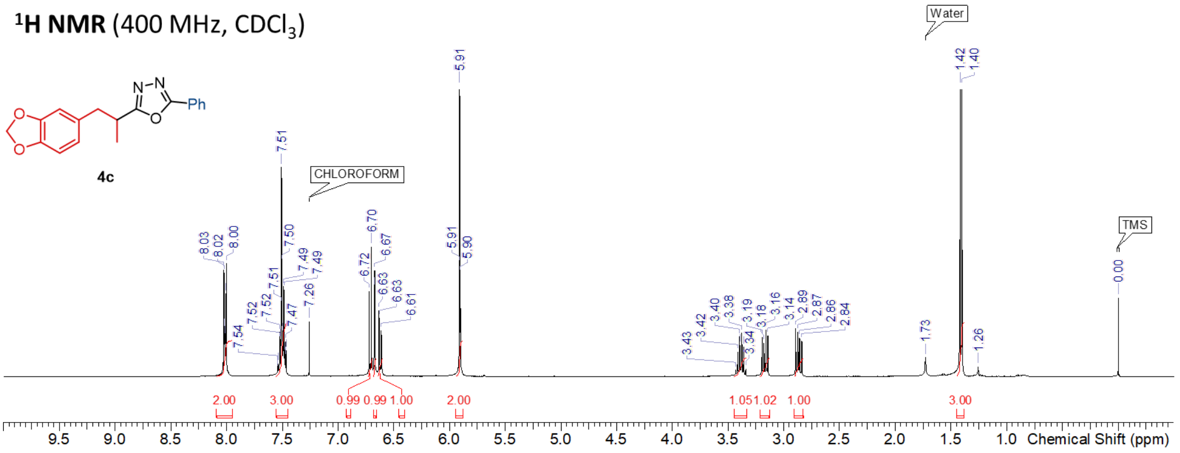

Figure S84. ^{1}H NMR spectrum (400 MHz, $CDCl_3$, 298 K) of **4c**.

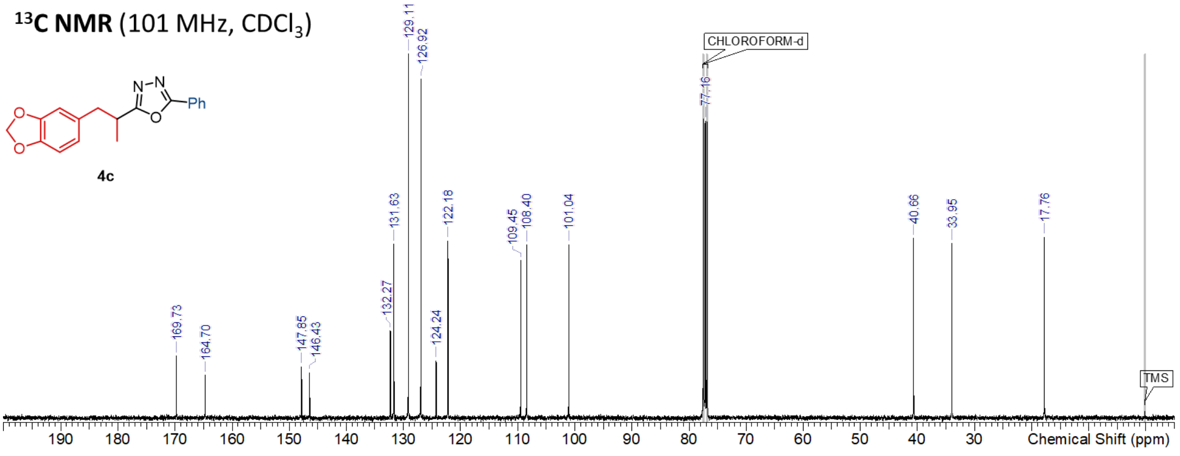

Figure S85. ^{13}C NMR spectrum (101 MHz, $CDCl_3$, 298 K) of **4c**.

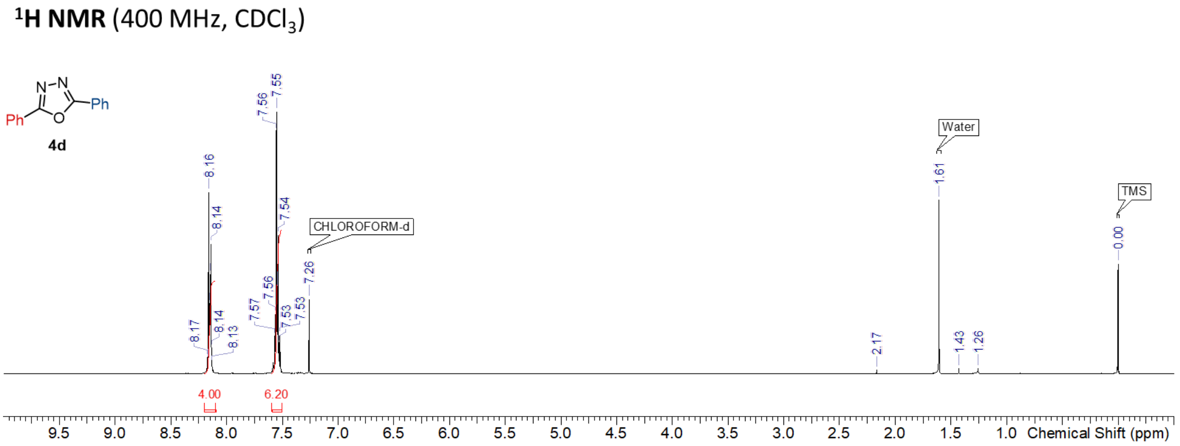

Figure S86. ^{1}H NMR spectrum (400 MHz, $CDCl_3$, 298 K) of **4d**.

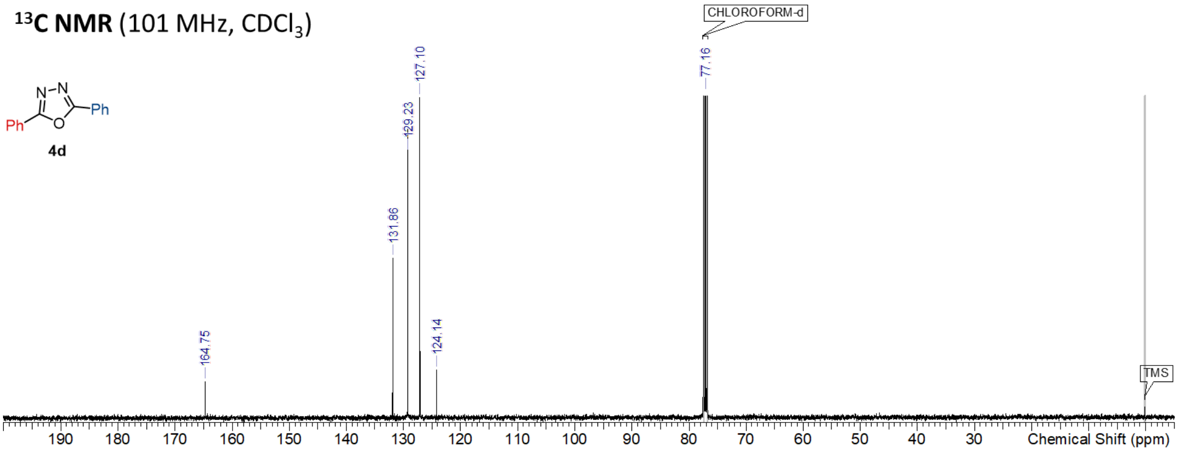

Figure S87. ^{13}C NMR spectrum (101 MHz, $CDCl_3$, 298 K) of **4d**.

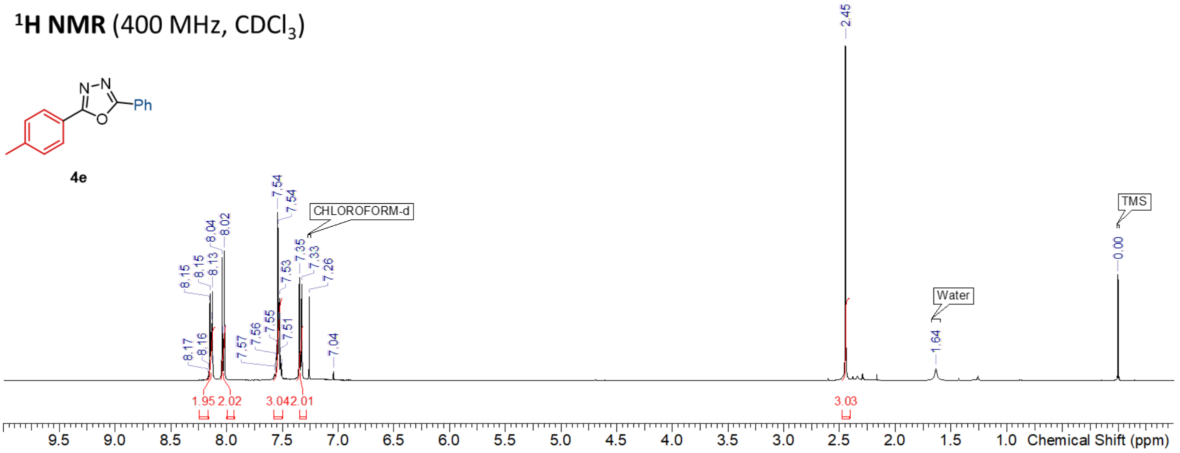

Figure S88. ^{1}H NMR spectrum (400 MHz, $CDCl_3$, 298 K) of **4e**.

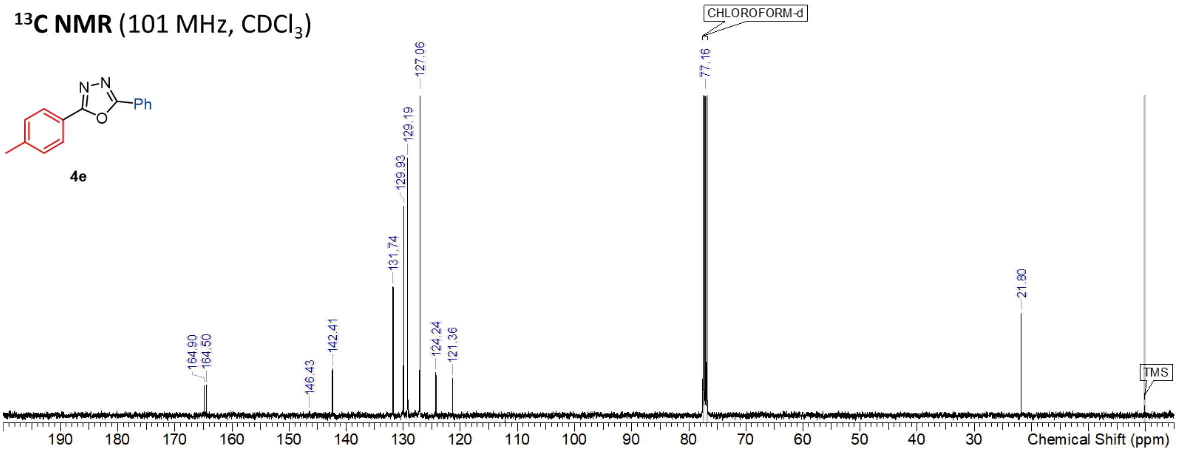

Figure S89. ^{13}C NMR spectrum (101 MHz, $CDCl_3$, 298 K) of **4e**.

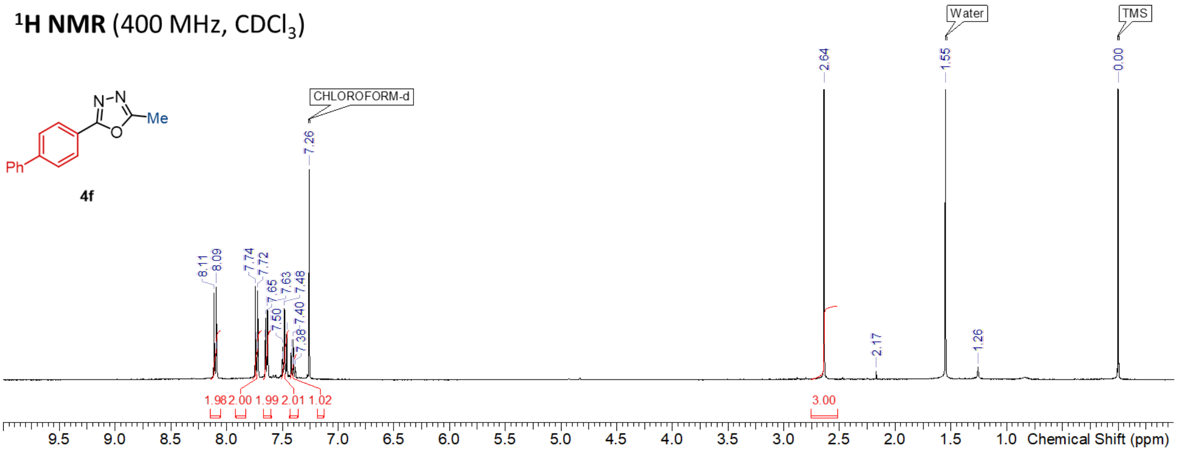

Figure S90. ^{1}H NMR spectrum (400 MHz, $CDCl_3$, 298 K) of **4f**.

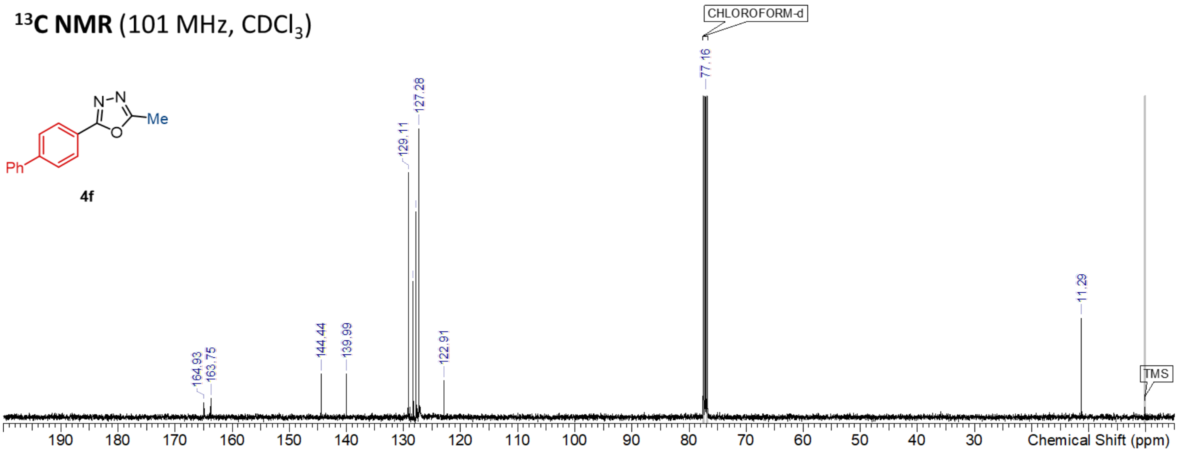

Figure S91. ^{13}C NMR spectrum (101 MHz, $CDCl_3$, 298 K) of **4f**.

^{1}H NMR (400 MHz, $CDCl_3$)

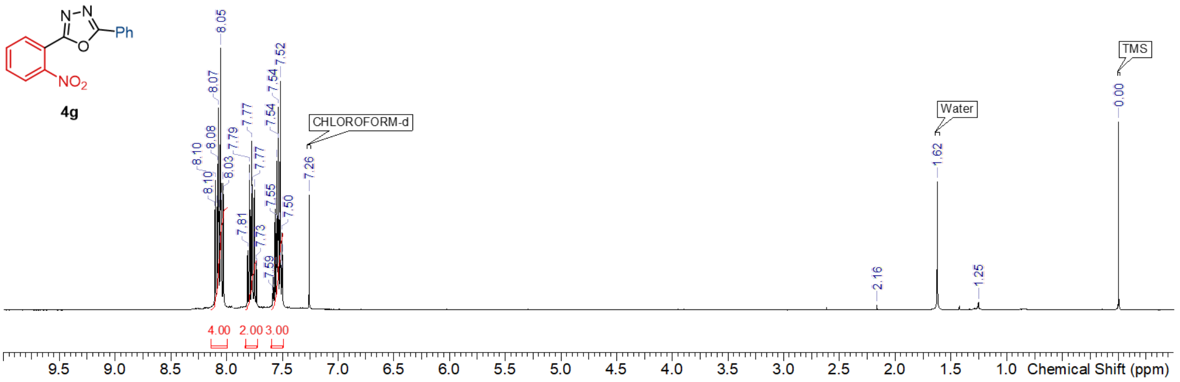

Figure S92. ^{1}H NMR spectrum (400 MHz, $CDCl_3$, 298 K) of **4g**.

^{13}C NMR (101 MHz, $CDCl_3$)

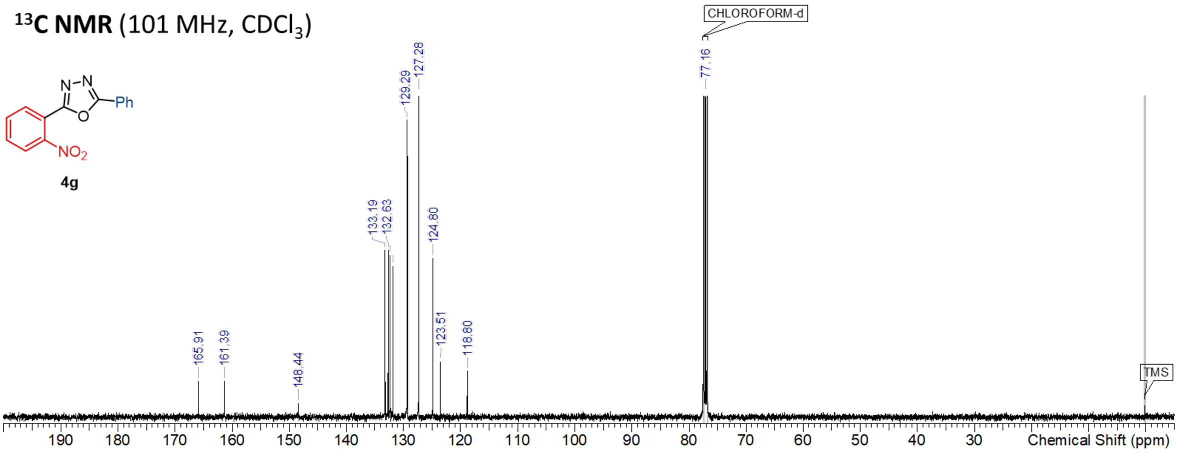

Figure S93. ^{13}C NMR spectrum (101 MHz, $CDCl_3$, 298 K) of **4g**.

^{1}H NMR (400 MHz, $CDCl_3$)

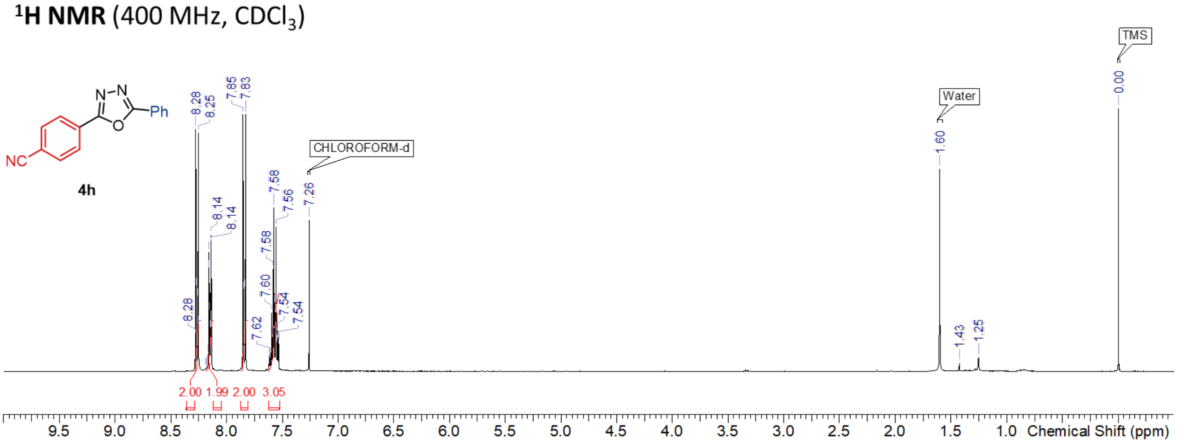

Figure S94. ^{1}H NMR spectrum (400 MHz, $CDCl_3$, 298 K) of **4h**.

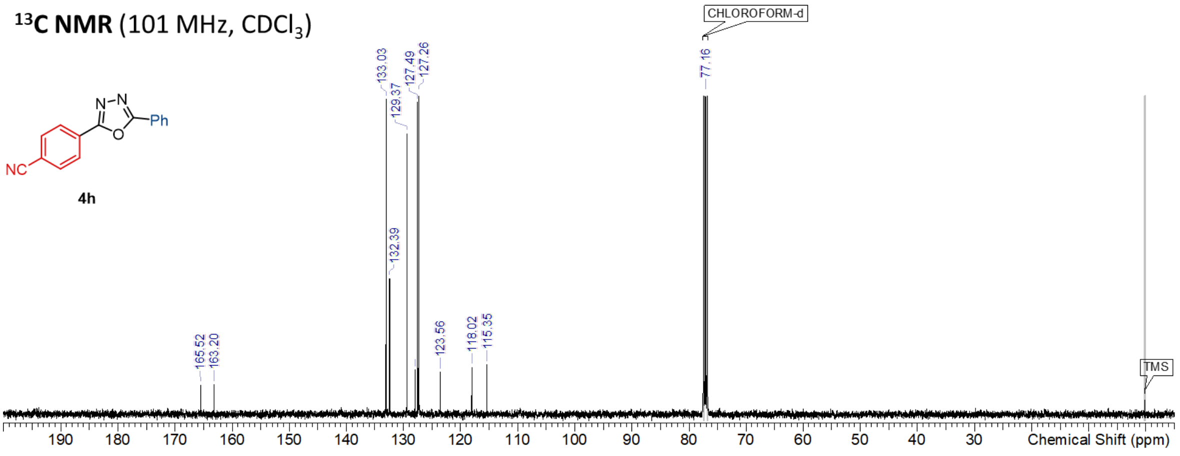

Figure S95. ^{13}C NMR spectrum (101 MHz, $CDCl_3$, 298 K) of **4h**.

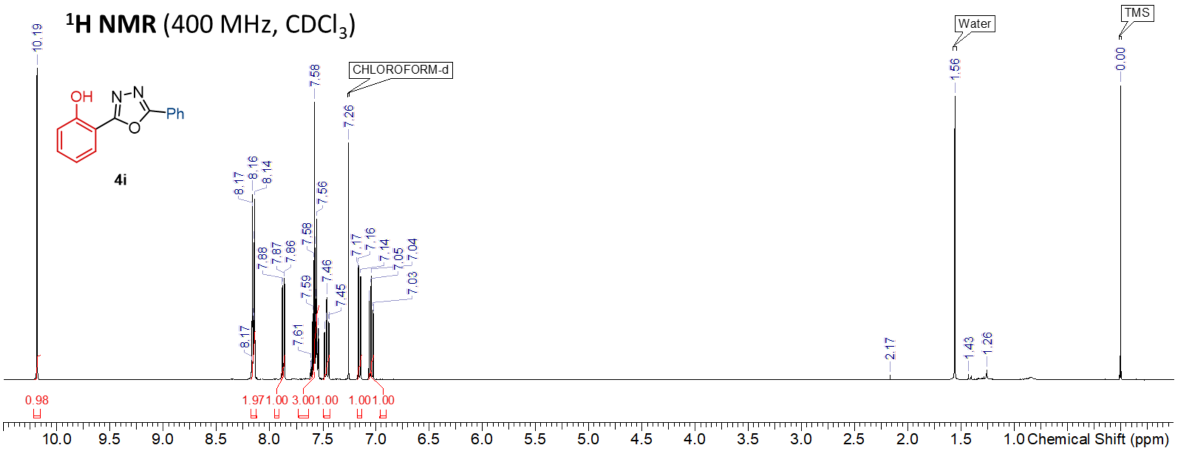

Figure S96. ^{1}H NMR spectrum (400 MHz, $CDCl_3$, 298 K) of **4i**.

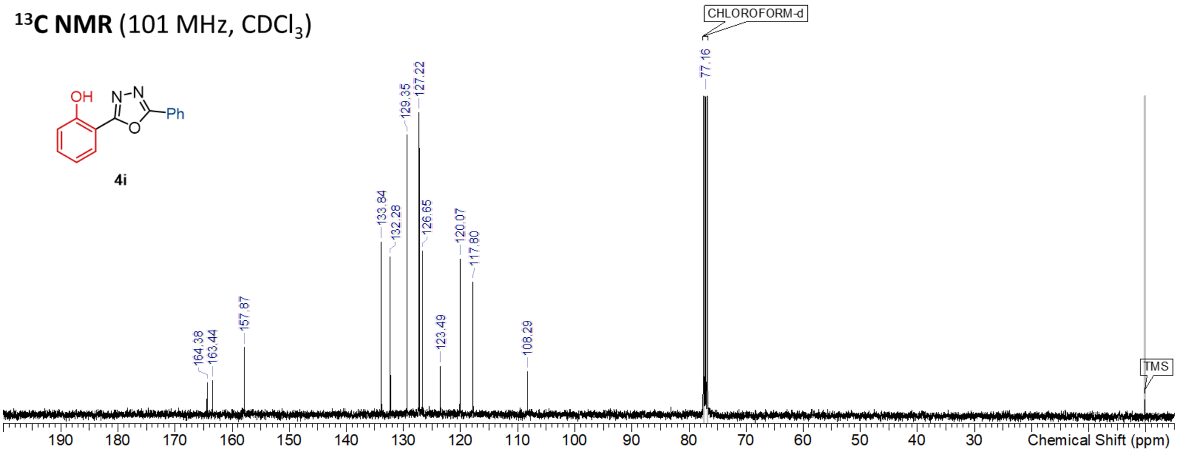

Figure S97. ^{13}C NMR spectrum (101 MHz, $CDCl_3$, 298 K) of **4i**.

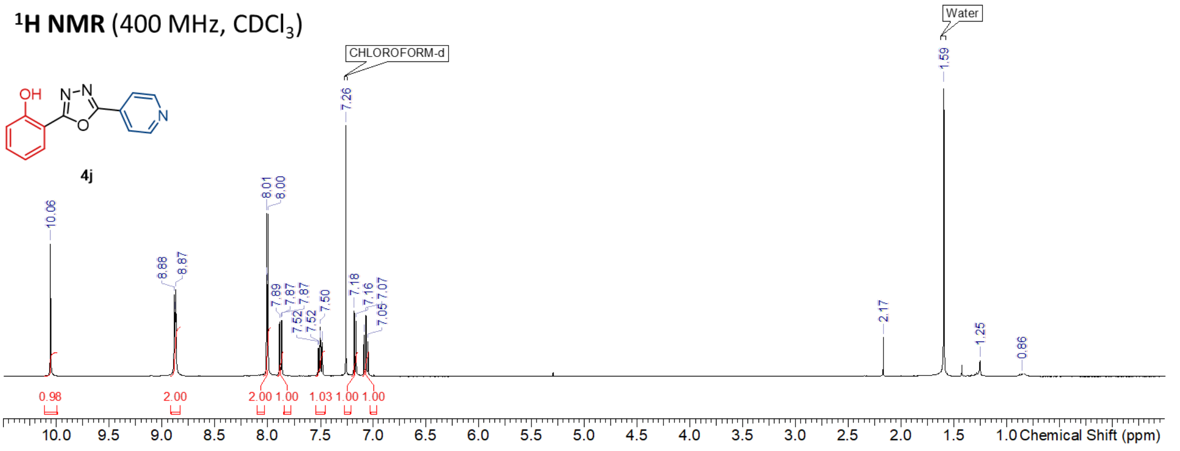

Figure S98. ^{1}H NMR spectrum (400 MHz, $CDCl_3$, 298 K) of **4j**.

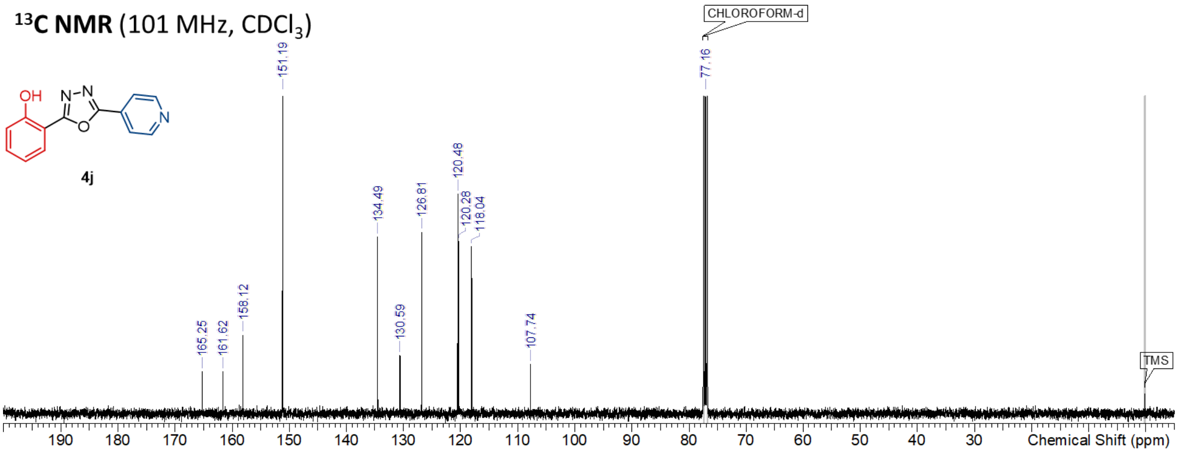

Figure S99. ^{13}C NMR spectrum (101 MHz, $CDCl_3$, 298 K) of **4j**.

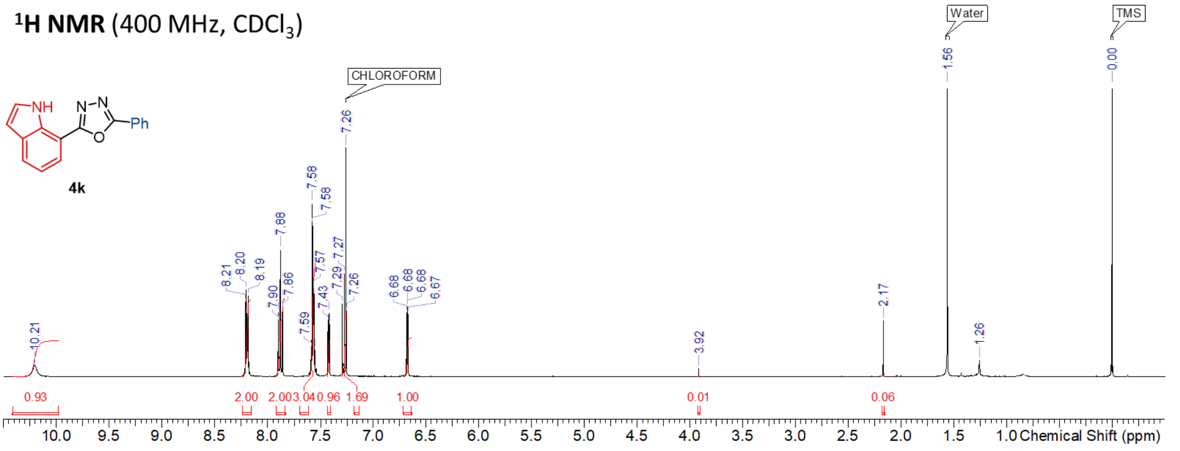

Figure S100. ^{1}H NMR spectrum (400 MHz, $CDCl_3$, 298 K) of **4k**.

^{13}**C NMR** (101 MHz, $CDCl_3$)

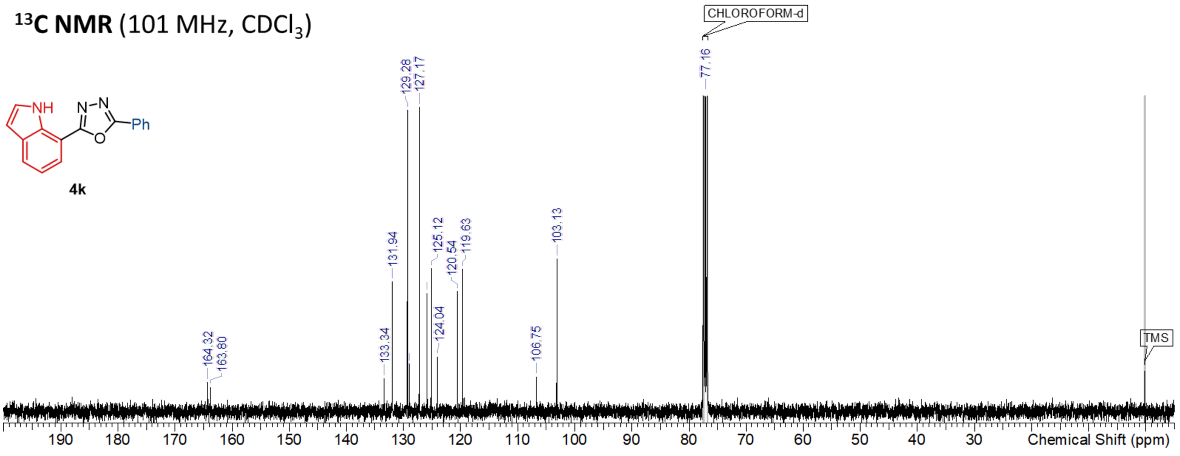

Figure S101. ^{13}C NMR spectrum (101 MHz, $CDCl_3$, 298 K) of **4k**.

^{1}**H NMR** (400 MHz, $CDCl_3$)

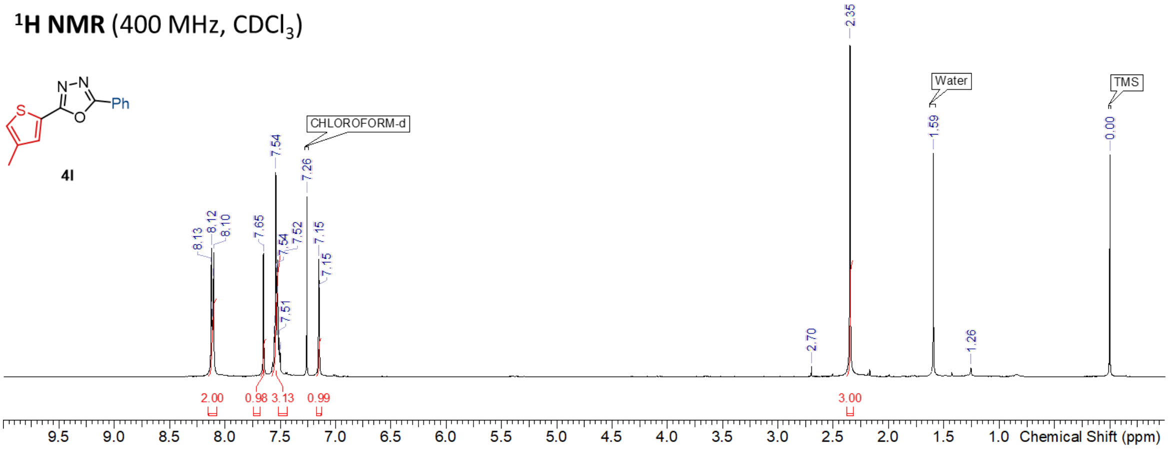

Figure S102. ^{1}H NMR spectrum (400 MHz, $CDCl_3$, 298 K) of **4l**.

^{13}**C NMR** (101 MHz, $CDCl_3$)

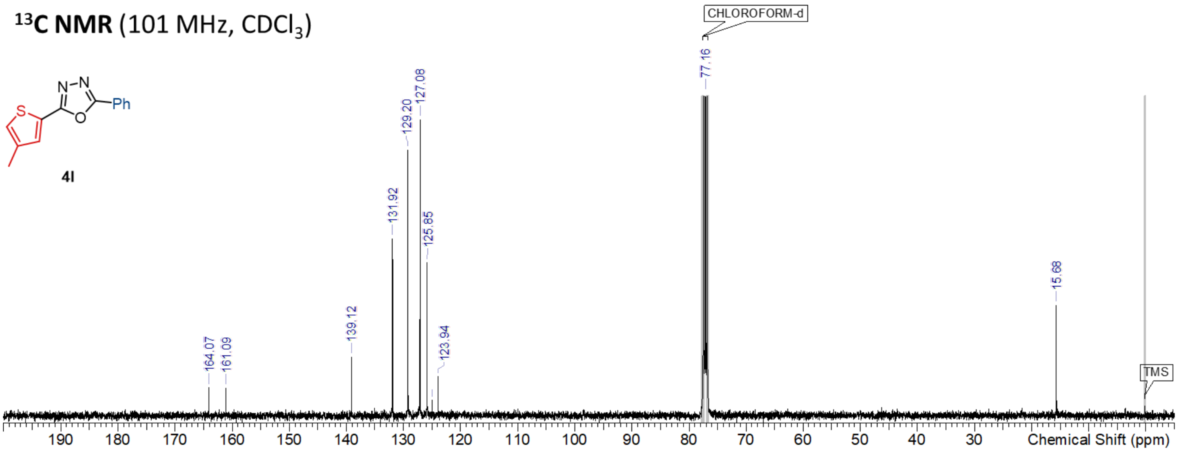

Figure S103. ^{13}C NMR spectrum (101 MHz, $CDCl_3$, 298 K) of **4l**.

^{1}**H NMR** (400 MHz, $CDCl_3$)

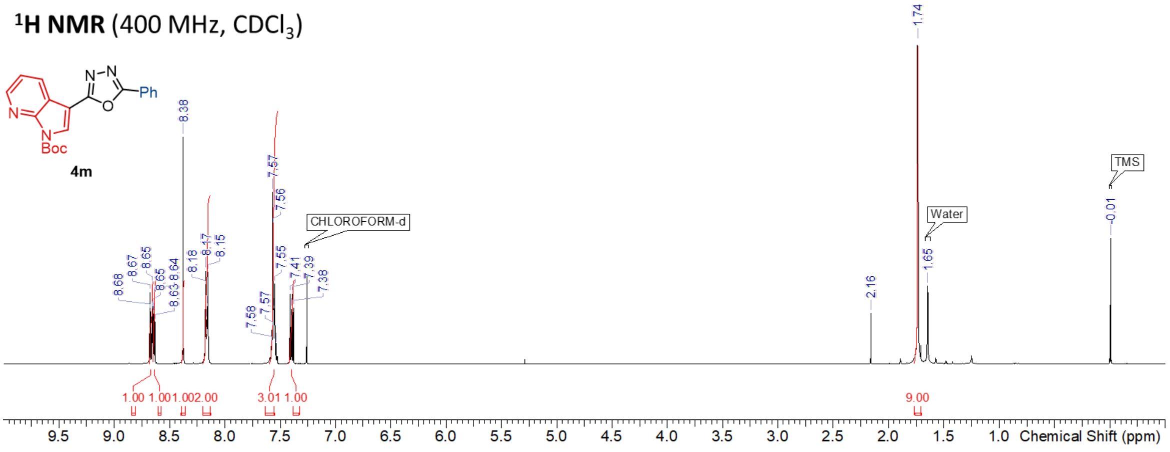

Figure S104. ^{1}H NMR spectrum (400 MHz, $CDCl_3$, 298 K) of **4m**.

^{13}**C NMR** (101 MHz, $CDCl_3$)

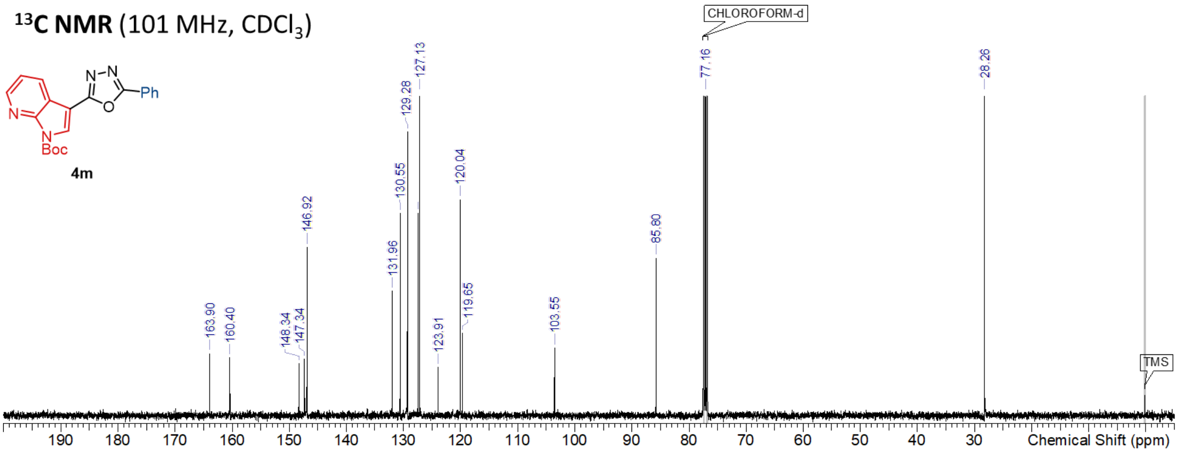

Figure S105. ^{13}C NMR spectrum (101 MHz, $CDCl_3$, 298 K) of **4m**.

^{1}**H NMR** (600 MHz, MeOD-d_4)

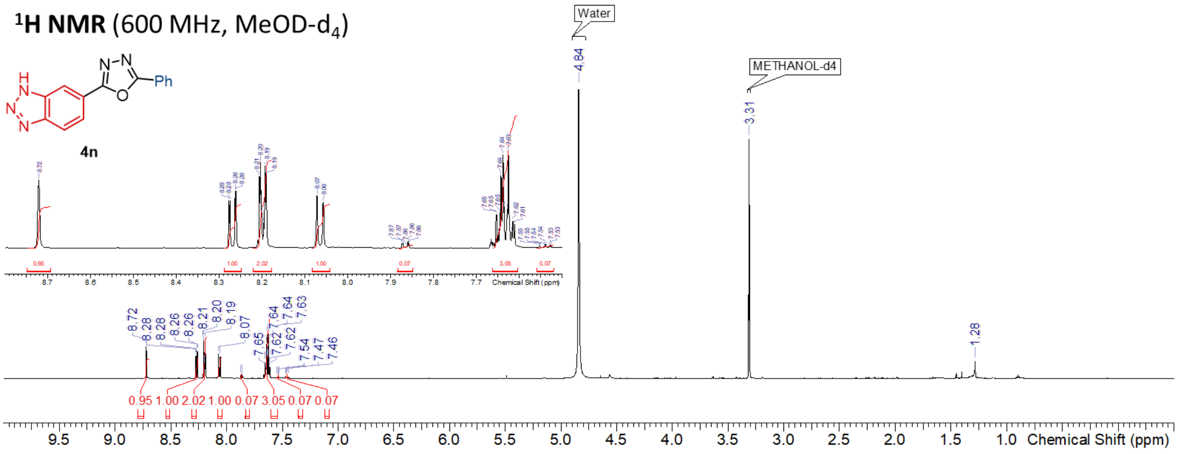

Figure S106. ^{1}H NMR spectrum (600 MHz, MeOD-d_4, 298 K) of **4n**.

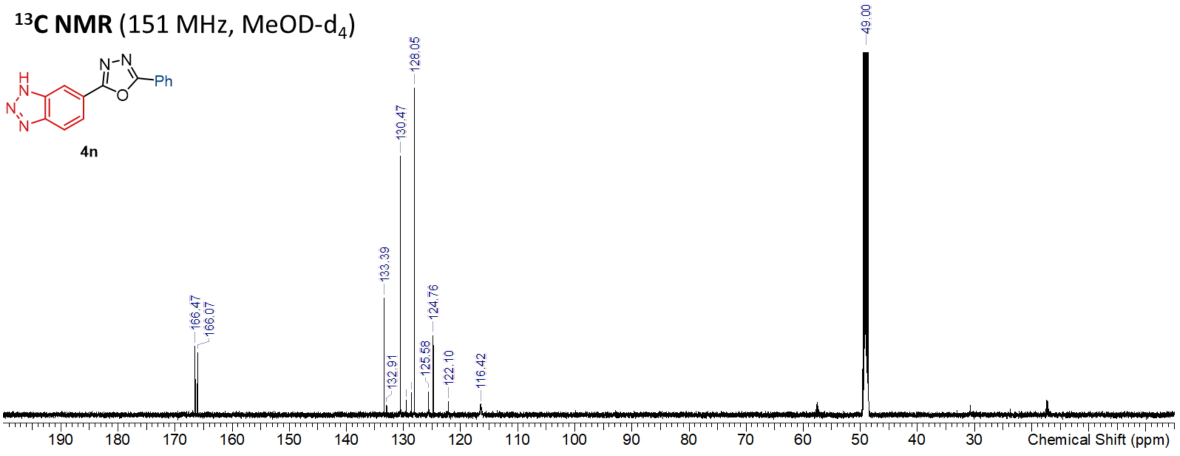

Figure S107. ^{13}C NMR spectrum (151 MHz, MeOD-d$_4$, 298 K) of **4n**.

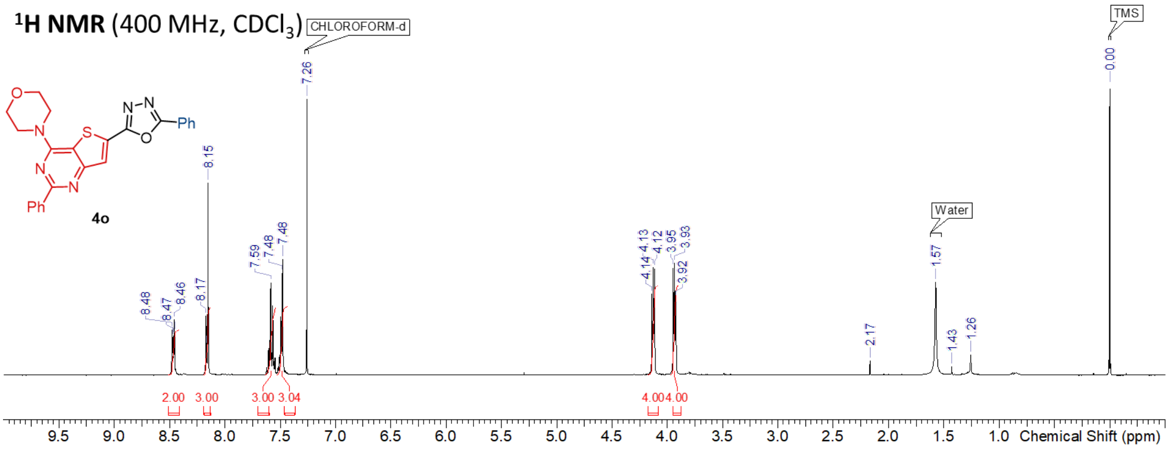

Figure S108. ^{1}H NMR spectrum (400 MHz, $CDCl_3$, 298 K) of **4o**.

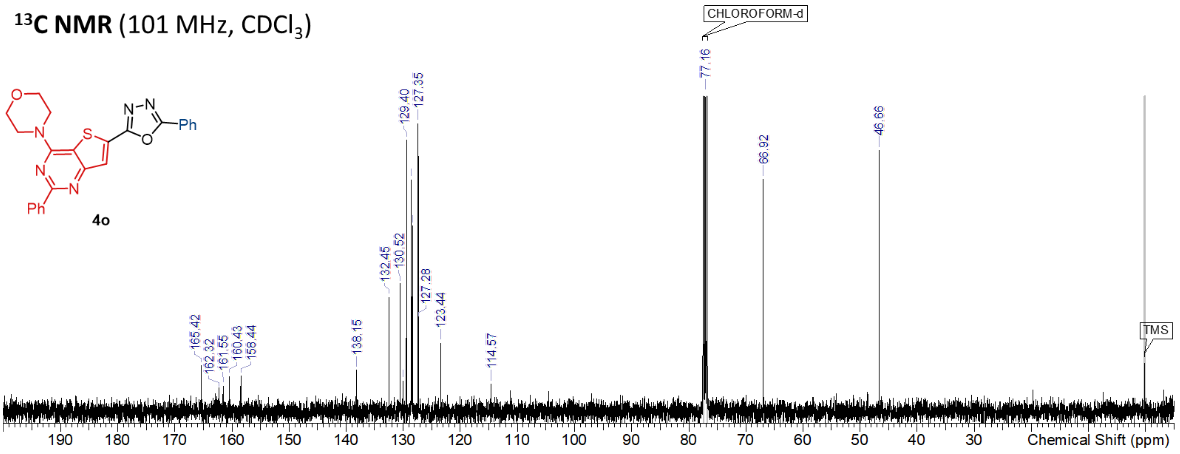

Figure S109. ^{13}C NMR spectrum (101 MHz, $CDCl_3$, 298 K) of **4o**.

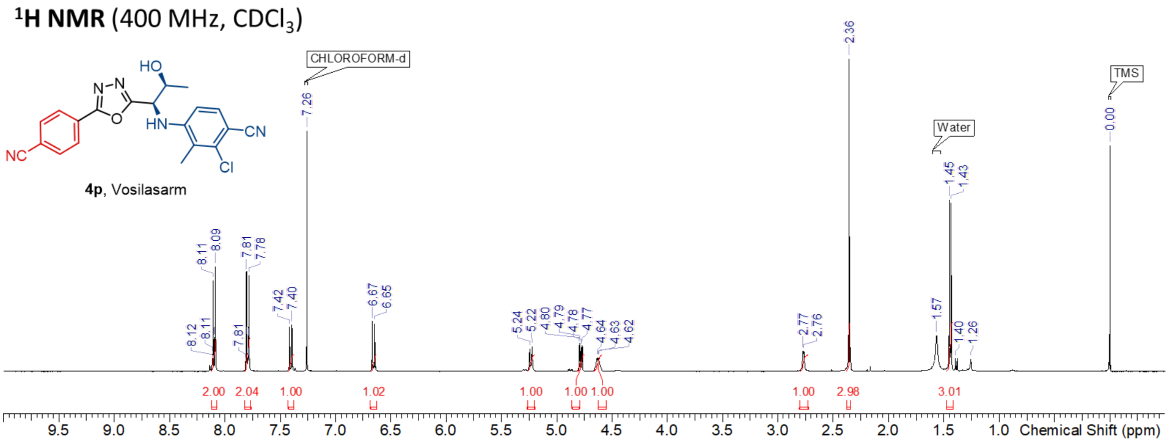

Figure S110. ^{1}H NMR spectrum (400 MHz, $CDCl_3$, 298 K) of **4p**.

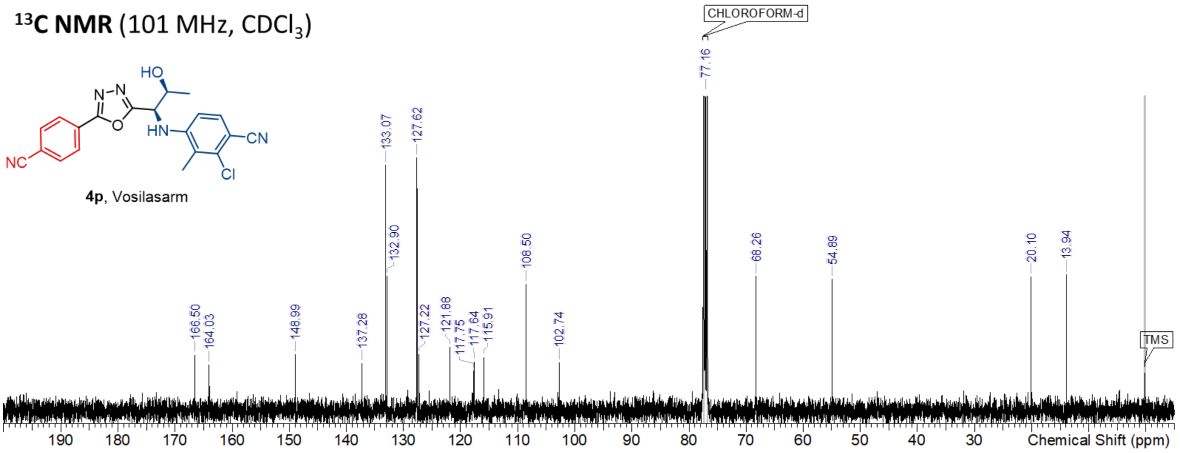

Figure S111. ^{13}C NMR spectrum (101 MHz, $CDCl_3$, 298 K) of **4p**.

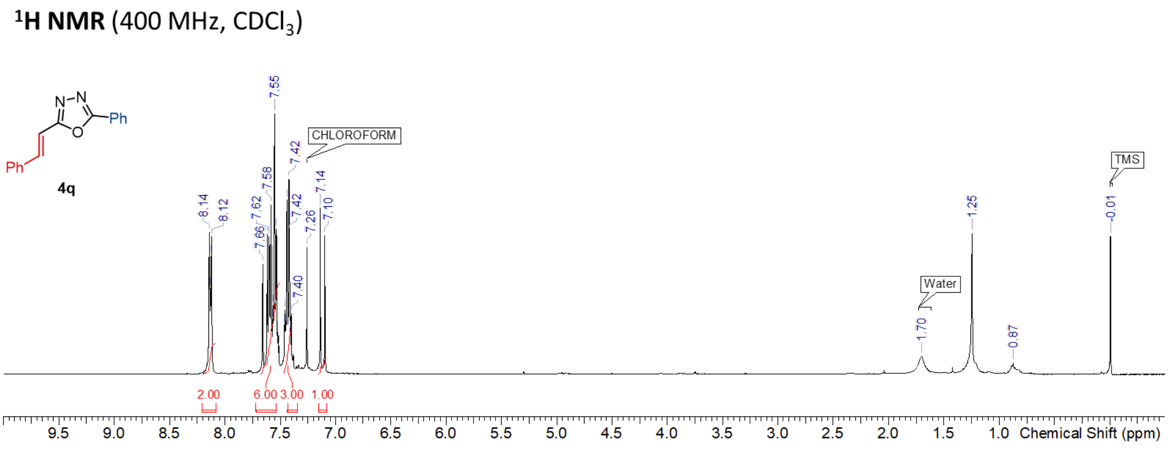

Figure S112. ^{1}H NMR spectrum (400 MHz, $CDCl_3$, 298 K) of **4q**.

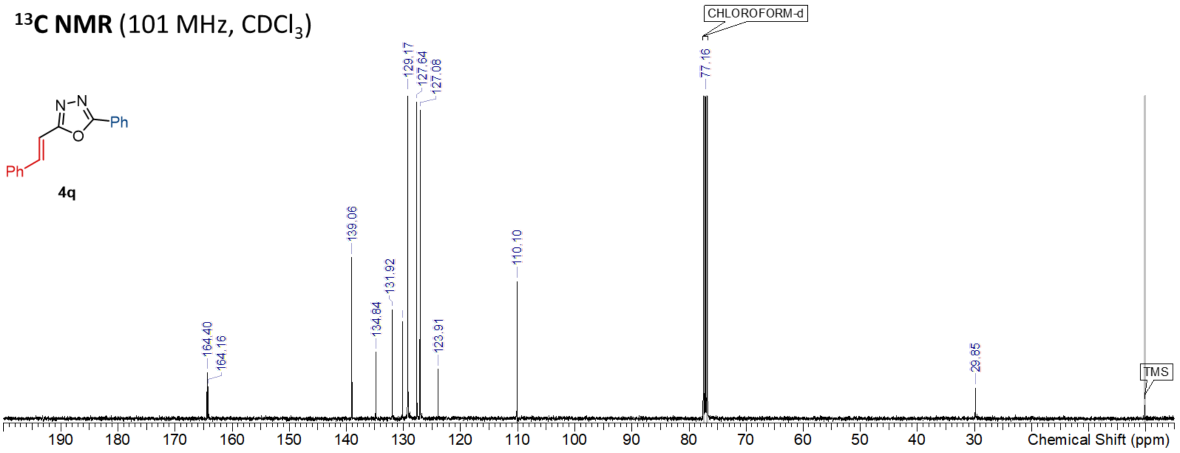

Figure S113. ^{13}C NMR spectrum (101 MHz, $CDCl_3$, 298 K) of **4q**.

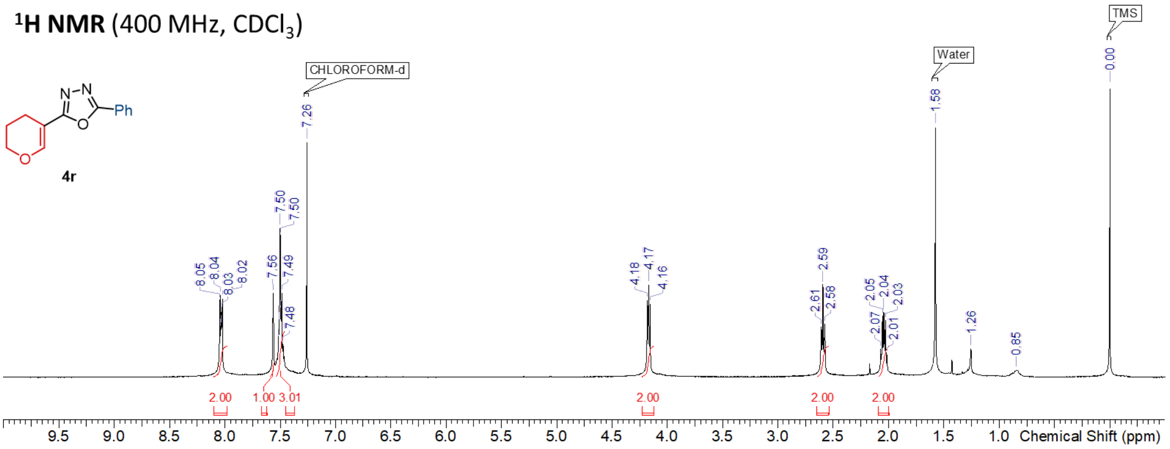

Figure S114. ^{1}H NMR spectrum (400 MHz, $CDCl_3$, 298 K) of **4r**.

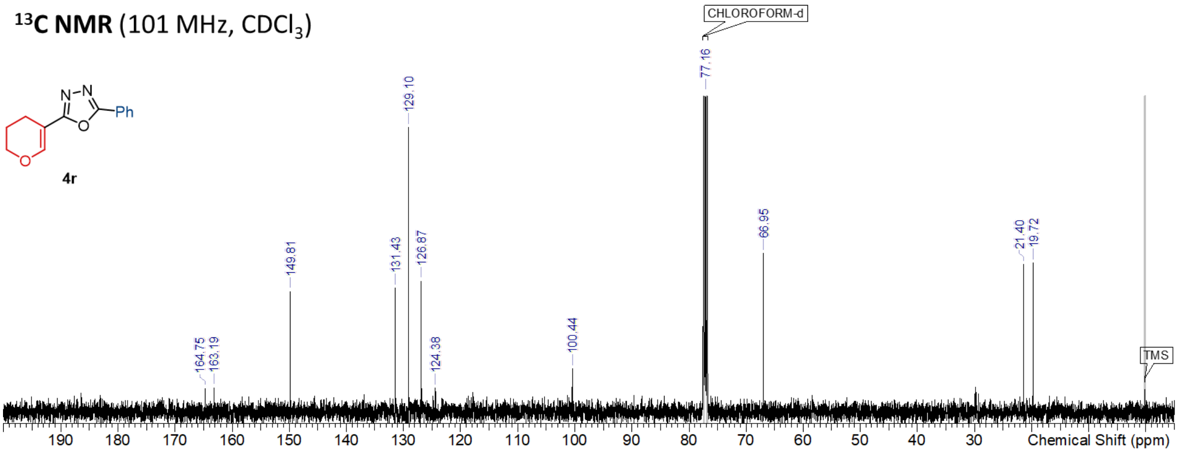

Figure S115. ^{13}C NMR spectrum (101 MHz, $CDCl_3$, 298 K) of **4r**.

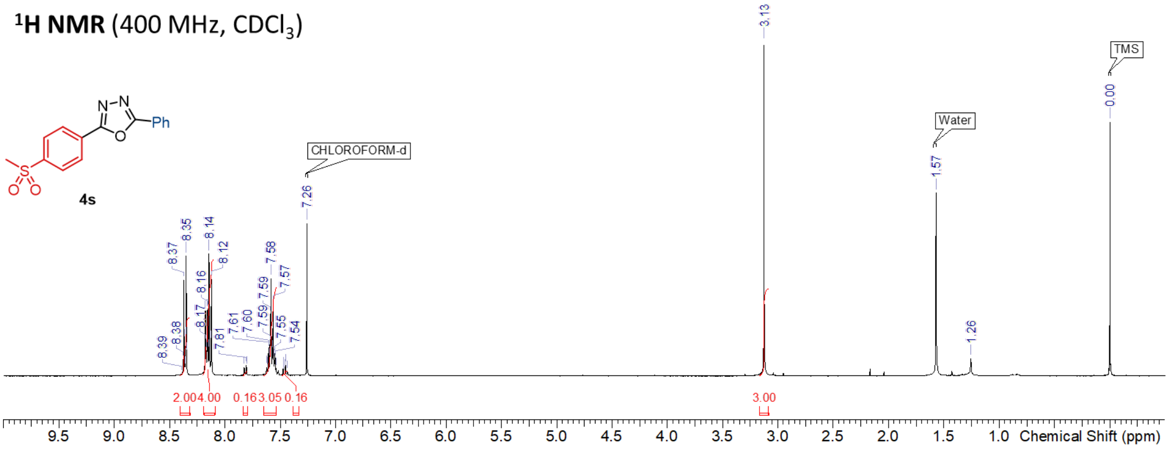

Figure S116. ¹H NMR spectrum (400 MHz, $CDCl_3$, 298 K) of **4s**.

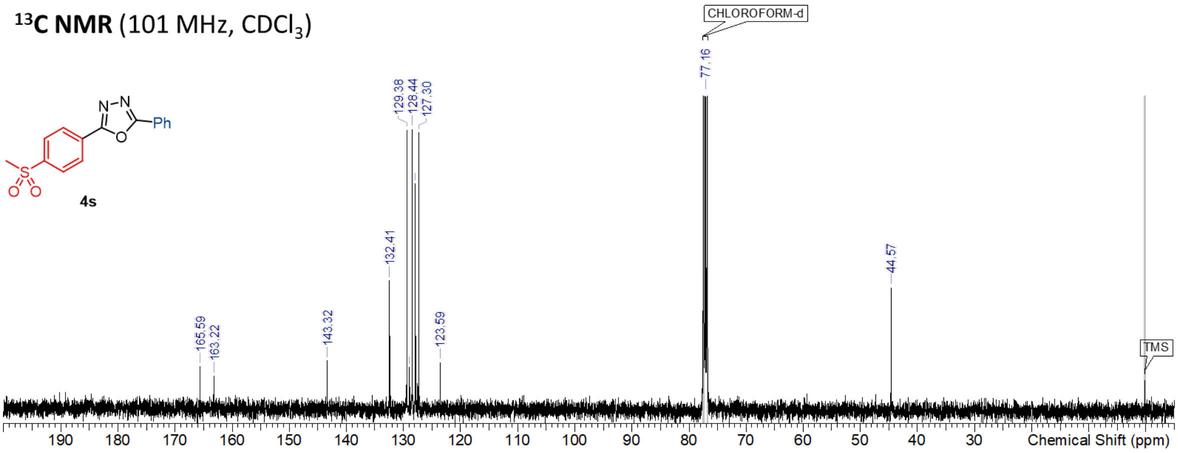

Figure S117. ¹³C NMR spectrum (101 MHz, $CDCl_3$, 298 K) of **4s**.

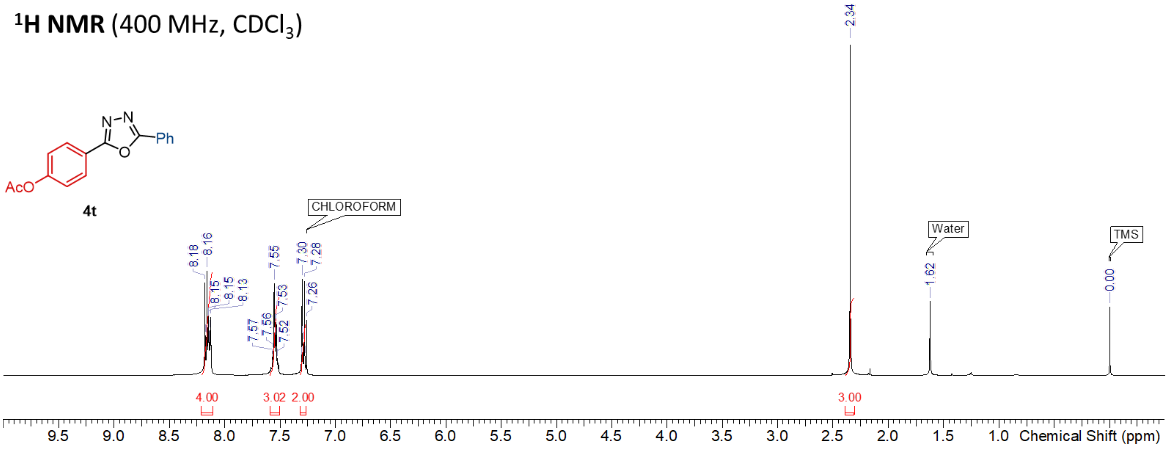

Figure S118. ¹H NMR spectrum (400 MHz, $CDCl_3$, 298 K) of **4t**.

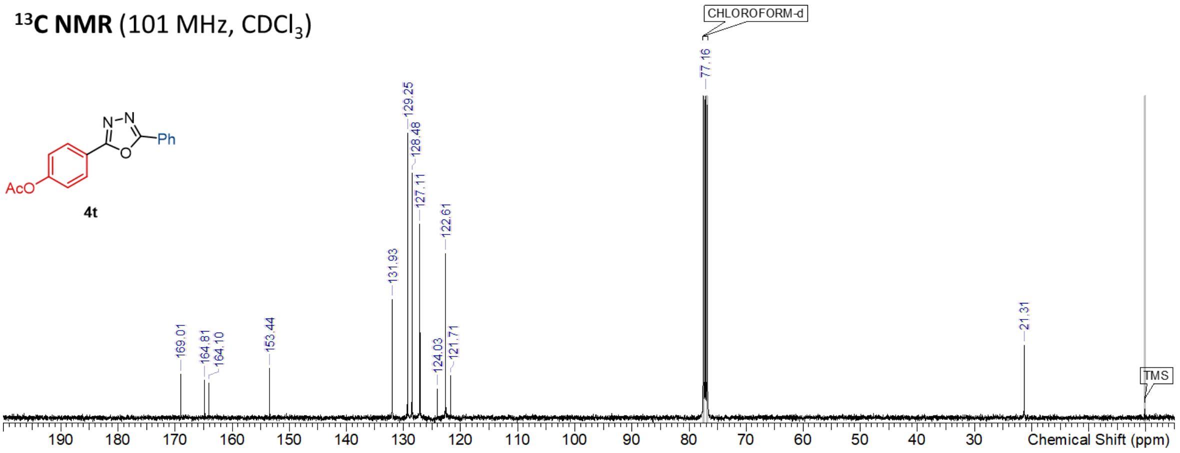

Figure S119. ^{13}C NMR spectrum (101 MHz, $CDCl_3$, 298 K) of **4t**.

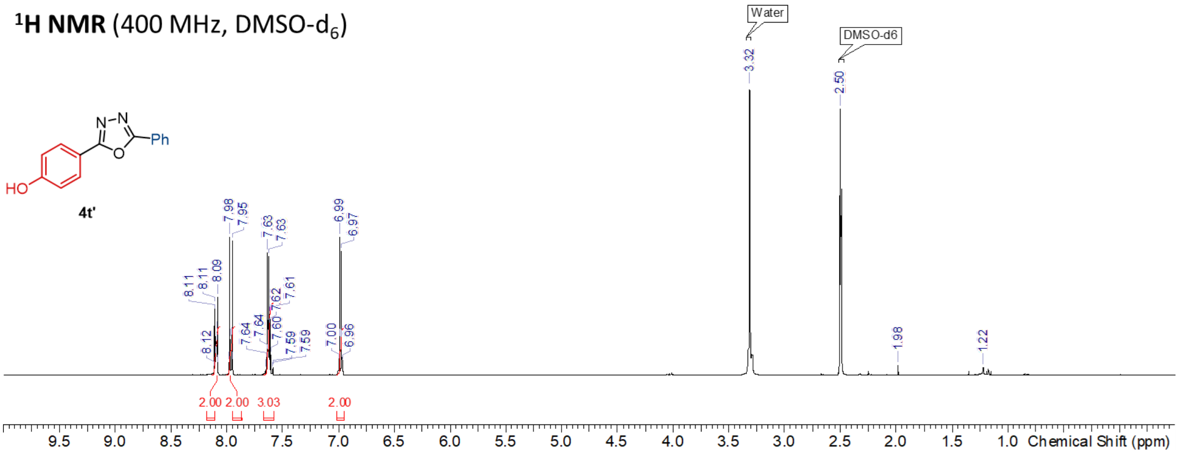

Figure S120. ^{1}H NMR spectrum (400 MHz, DMSO-d_6, 298 K) of **4t'**.

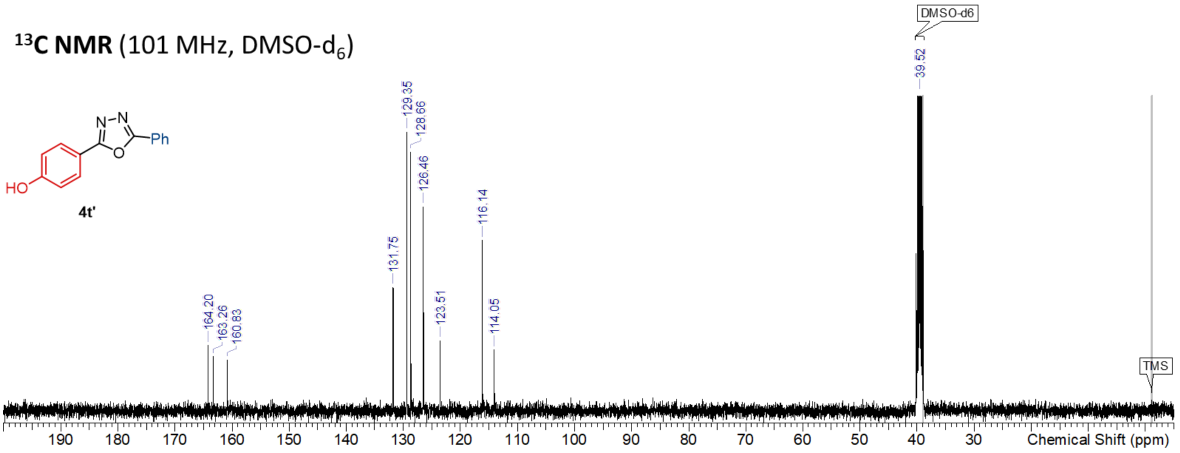

Figure S121. ^{13}C NMR spectrum (101 MHz, DMSO-d_6, 298 K) of **4t'**.

^{1}H NMR (400 MHz, $CDCl_3$)

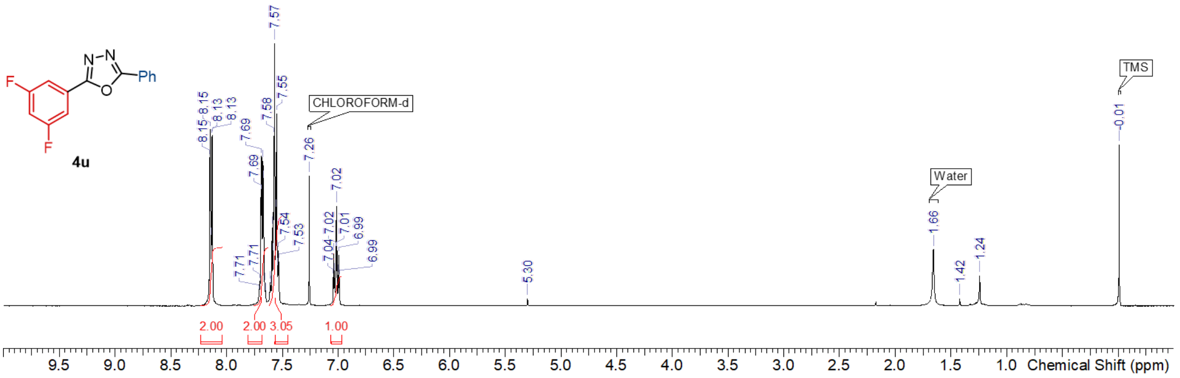

Figure S122. ^{1}H NMR spectrum (400 MHz, $CDCl_3$, 298 K) of **4u**.

^{13}C NMR (101 MHz, $CDCl_3$)

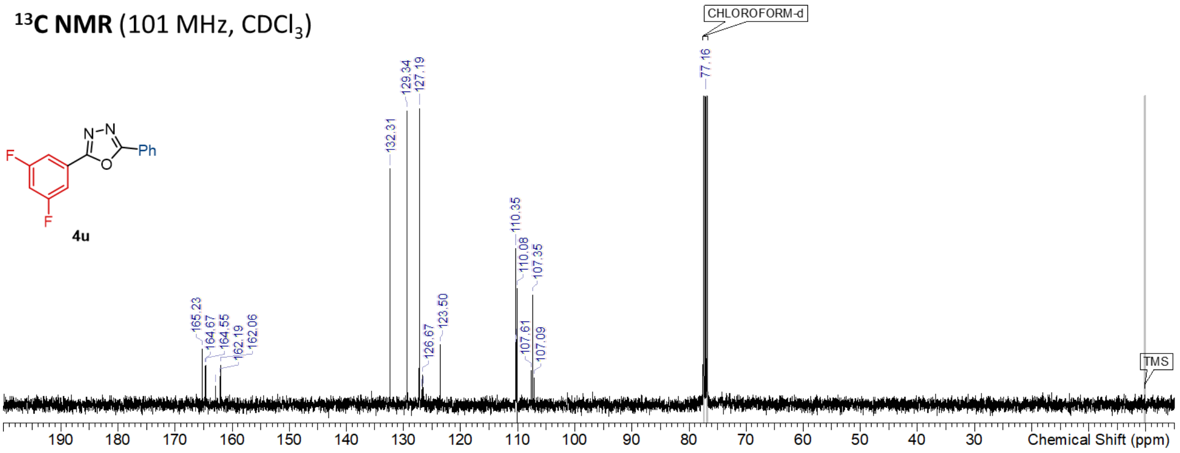

Figure S123. ^{13}C NMR spectrum (101 MHz, $CDCl_3$, 298 K) of **4u**.

^{19}F NMR (376 MHz, $CDCl_3$)

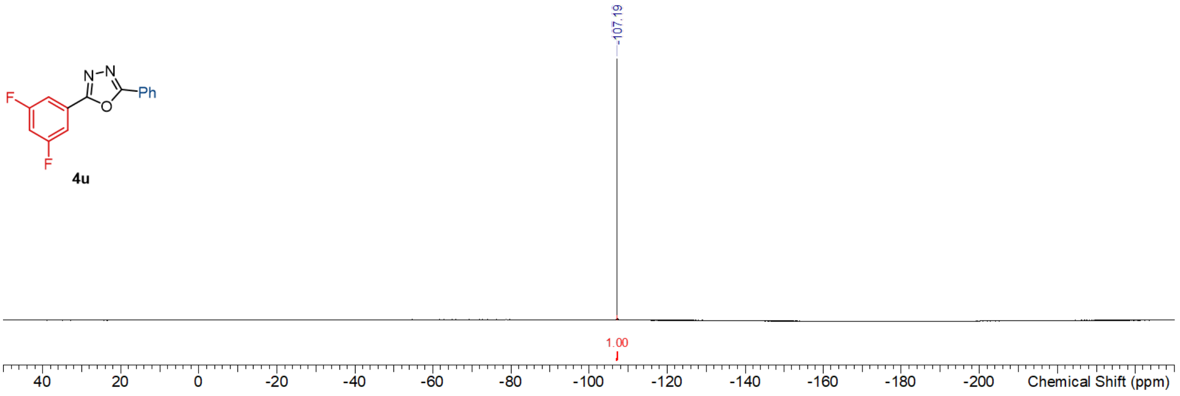

Figure S124. ^{19}F NMR spectrum (376 MHz, $CDCl_3$, 298 K) of **4u**.

^{1}H NMR (400 MHz, $CDCl_3$)

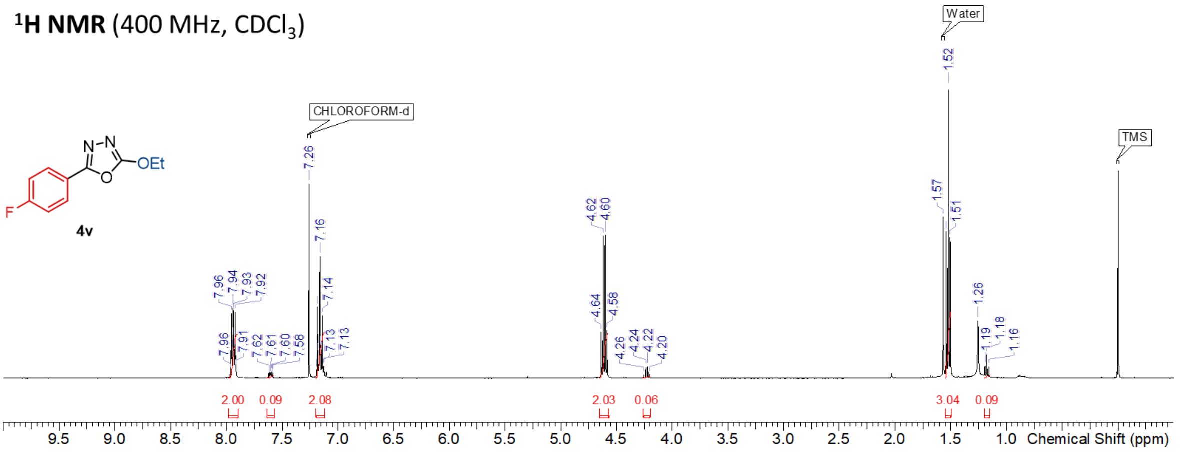

Figure S125. ^{1}H NMR spectrum (400 MHz, $CDCl_3$, 298 K) of **4v**.

^{13}C NMR (101 MHz, $CDCl_3$)

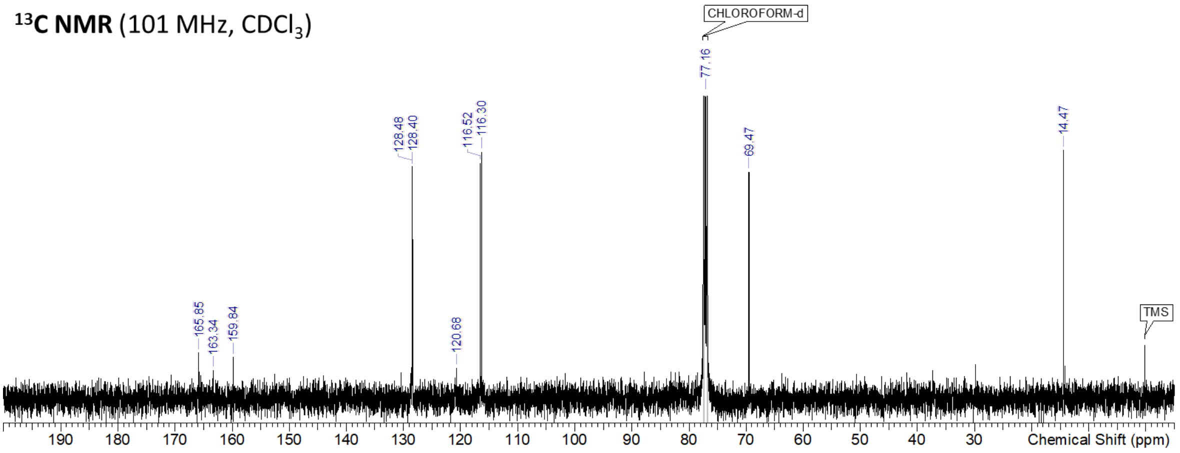

Figure S126. ^{13}C NMR spectrum (101 MHz, $CDCl_3$, 298 K) of **4v**.

^{19}F NMR (376 MHz, $CDCl_3$)

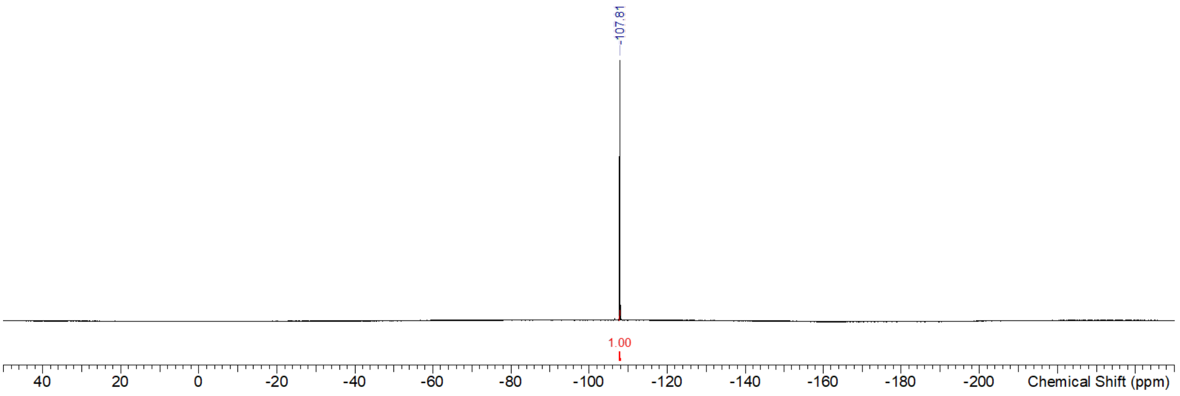

Figure S127. ^{19}F NMR spectrum (376 MHz, $CDCl_3$, 298 K) of **4v**.

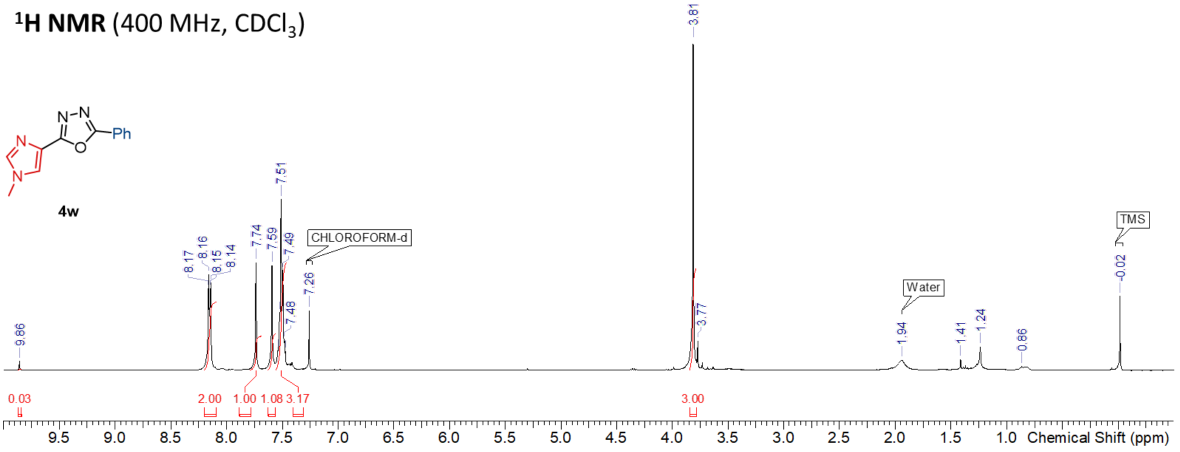

Figure S128. ^{1}H NMR spectrum (400 MHz, $CDCl_3$, 298 K) of **4w**.

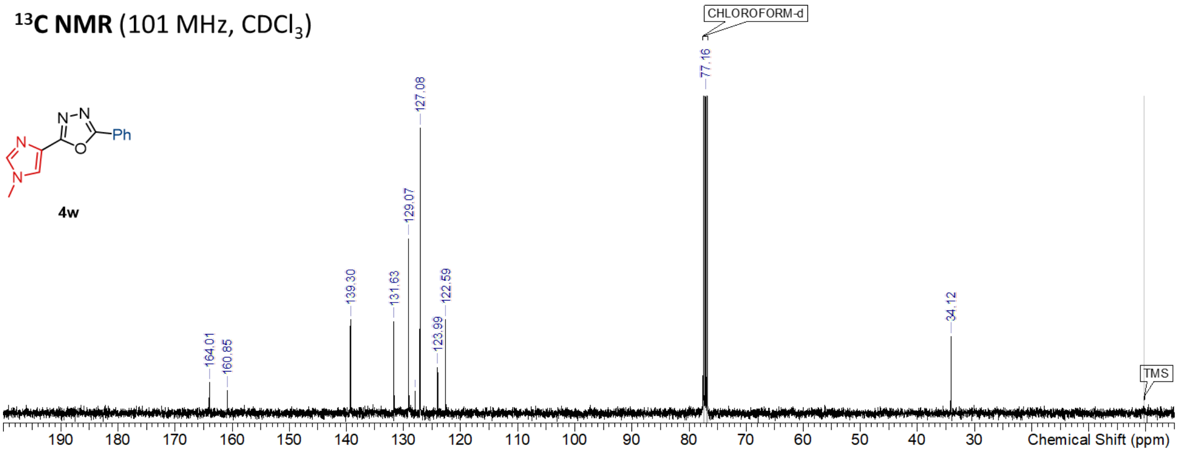

Figure S129. ^{13}C NMR spectrum (101 MHz, $CDCl_3$, 298 K) of **4w**.

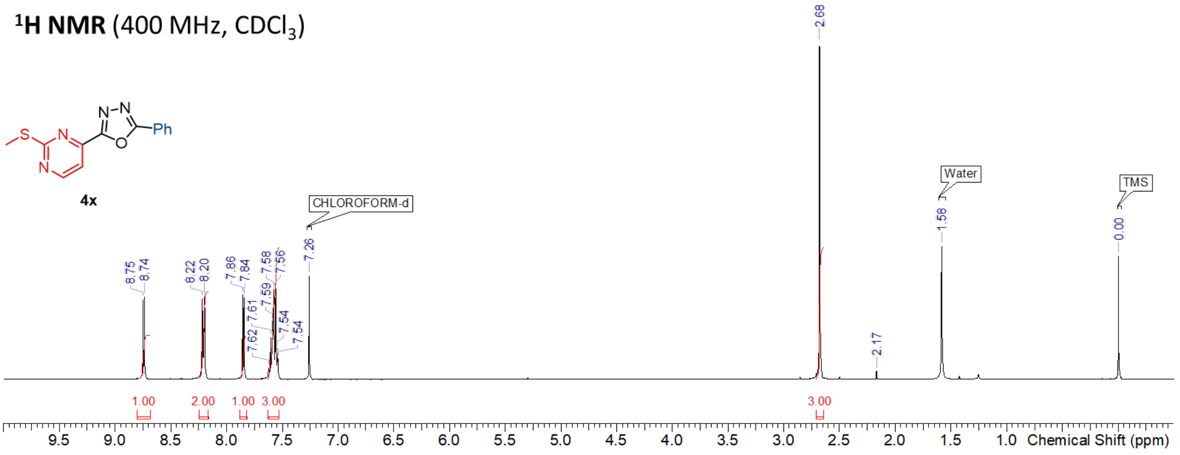

Figure S130. ^{1}H NMR spectrum (400 MHz, $CDCl_3$, 298 K) of **4x**.

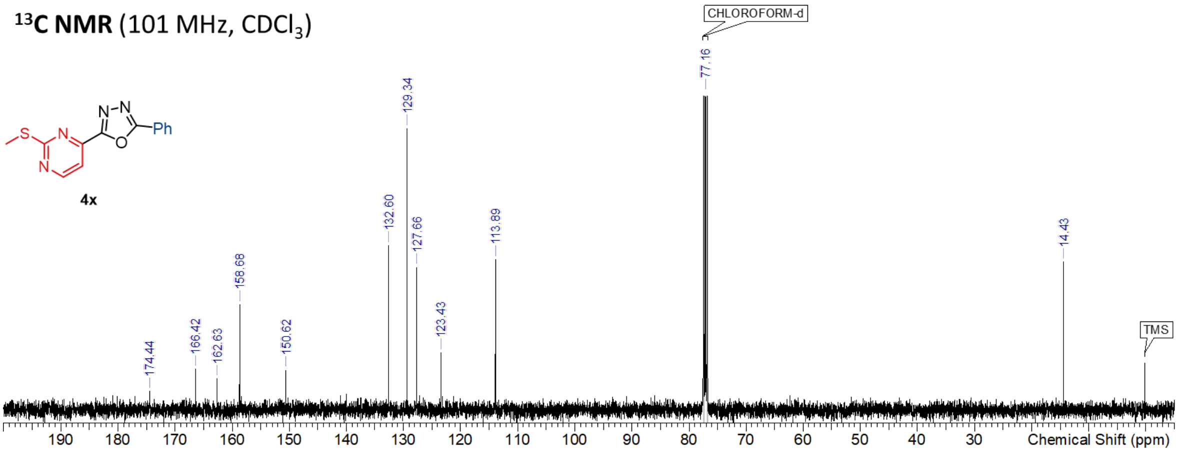

Figure S131. ^{13}C NMR spectrum (101 MHz, $CDCl_3$, 298 K) of **4x**.

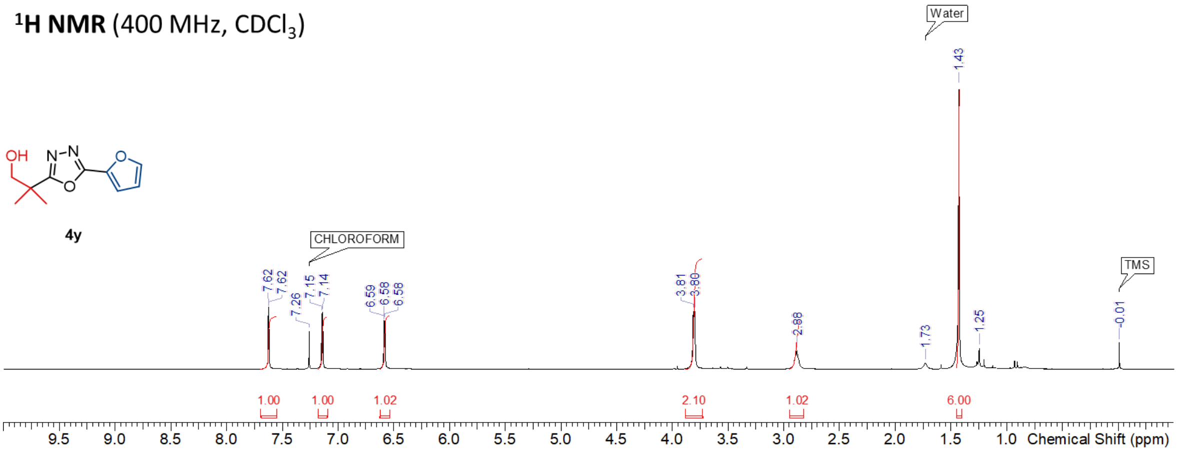

Figure S132. ^{1}H NMR spectrum (400 MHz, $CDCl_3$, 298 K) of **4y**.

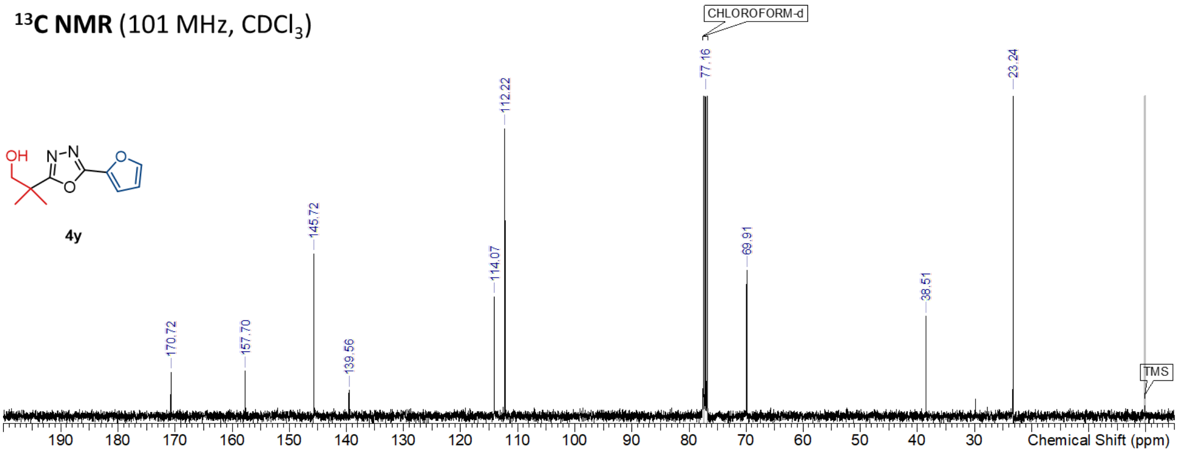

Figure S133. ^{13}C NMR spectrum (101 MHz, $CDCl_3$, 298 K) of **4y**.

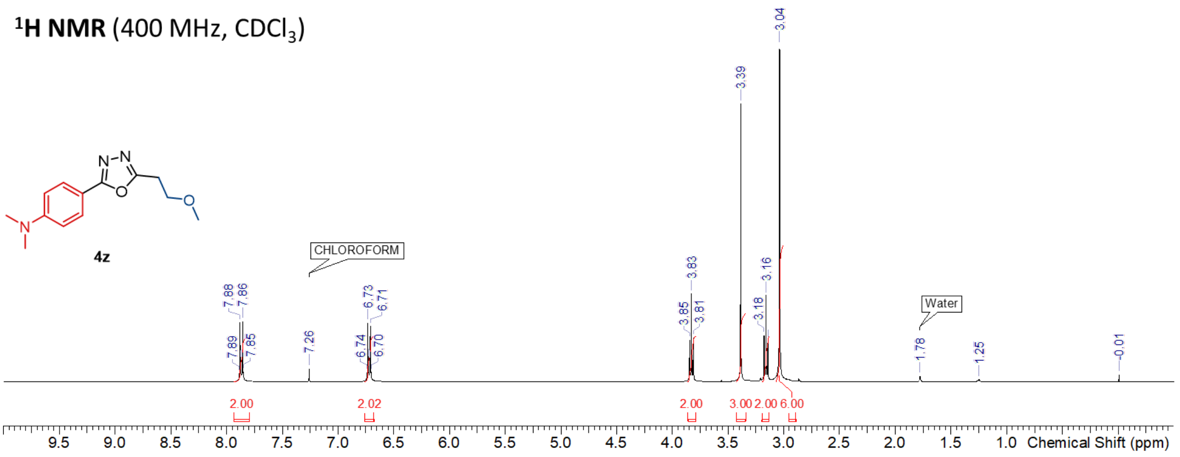

Figure S134. ^{1}H NMR spectrum (400 MHz, $CDCl_3$, 298 K) of **4z**.

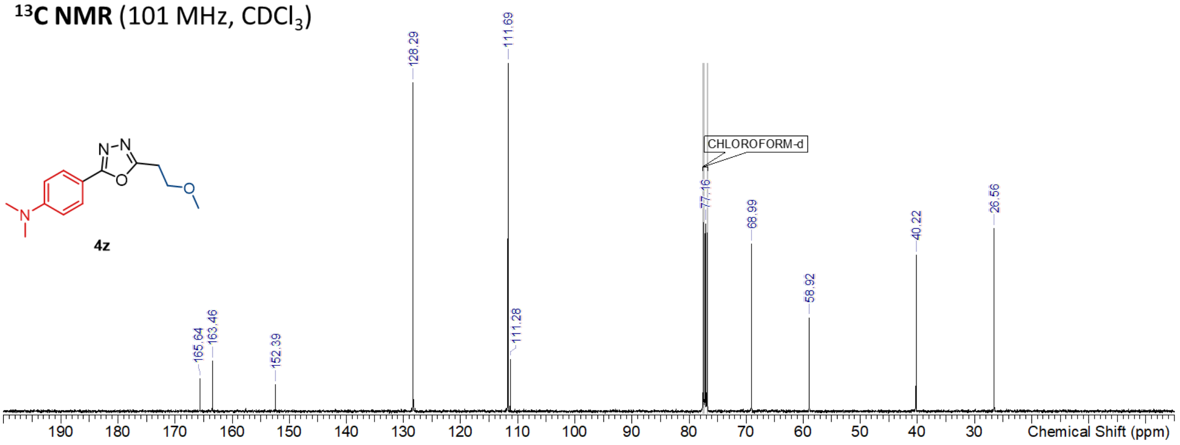

Figure S135. ^{13}C NMR spectrum (101 MHz, $CDCl_3$, 298 K) of **4z**.

9. References

1 J. T. Gerig and S. J. Hammond, *J. Am. Chem. Soc.*, 1984, **106**, 8244–8251.

2 T. Delgado-Maldonado, B. Nogueda-Torres, J. C. Espinoza-Hicks, L. K. Vázquez-Jiménez, A. D. Paz-González, A. Juárez-Saldívar and G. Rivera, *Mol. Divers.*, 2022, **26**, 39–50.

3 A. Palakkathondi, J. M. Oh, S. Dev, T. M. Rangarajan, S. Kaipakasseri, F. S. Kavully, N. Gambacorta, O. Nicolotti, H. Kim and B. Mathew, *ACS Comb. Sci.*, 2020, **22**, 592–599.

4 B. Liu, R. Li, Y. Li, S. Li, J. Yu, B. Zhao, A. Liao, Y. Wang, Z. Wang, A. Lu, Y. Liu and Q. Wang, *J. Agric. Food Chem.*, 2019, **67**, 1795–1806.

5 J. B. P. da Silva, D. M. do A. F. Navarro, A. G. da Silva, G. K. N. Santos, K. A. Dutra, D. R. Moreira, M. N. Ramos, J. W. P. Espíndola, A. D. T. de Oliveira, D. J. Brondani, A. C. L. Leite, M. Z. Hernandes, V. R. A. Pereira, L. F. da Rocha, M. C. A. B. de Castro, B. C. de Oliveira, Q. Lan and K. M. Merz, *Eur. J. Med. Chem.*, 2015, **100**, 162–175.

6 M. Kim and S. Lee, *Synthesis*, 2024, **56**, 2263–2269.

7 S. Luo, X. Xu, P. Zhang, Q. Shi, X. Yang and F. Li, *Org. Lett.*, 2022, **24**, 9081–9085.

8 L. Martínez-Crespo, L. Halgreen, M. Soares, I. Marques, V. Félix and H. Valkenier, *Org. Biomol.*

Chem., 2021, **19**, 8324–8337.

9 C. P. Miller, M. Shomali, C. R. Lyttle, L. S. L. O'Dea, H. Herendeen, K. Gallacher, D. Paquin, D. R. Compton, B. Sahoo, S. A. Kerrigan, M. S. Burge, M. Nickels, J. L. Green, J. A. Katzenellenbogen, A. Tchesnokov and G. Hattersley, *ACS Med. Chem. Lett.*, 2011, **2**, 124–129.

10 K. Donnelly and M. Baumann, *Beilstein J. Org. Chem.*, 2022, **18**, 232–239.

11 B.-K. Kim, H. Ko, E.-S. Jeon, E.-S. Ju, L. S. Jeong and Y.-C. Kim, *Eur. J. Med. Chem.*, 2016, **120**, 202–216.

12 Q. Li, Y. Tao, D. Xu, H. Zhang and L. Duan, *J. Chinese Chem. Soc.*, 2014, **61**, 665–670.

13 D. Matheau-Raven and D. J. Dixon, *J. Org. Chem.*, 2022, **87**, 12498–12505.

14 Y. Fan, Y. He, X. Liu, T. Hu, H. Ma, X. Yang, X. Luo and G. Huang, *J. Org. Chem.*, 2016, **81**, 6820–6825.

15 V. Karabelyov, V. T. Angelova, M. Sharkov, R. Mihaylova, G. Popov, T. Pencheva, V. Manov, M. Dangalov, N. Todorova and M. Kondeva-Burdina, *J. Mol. Struct.*, 2023, **1288**, 135755.

16 J. Ramprasad, N. Nayak, U. Dalimba, P. Yogeeswari, D. Sriram, S. K. Peethambar, R. Achur and H. S. S. Kumar, *Eur. J. Med. Chem.*, 2015, **95**, 49–63.

17 W. Mahy, N. J. Willis, Y. Zhao, H. L. Woodward, F. Svensson, J. Sipthorp, L. Vecchia, R. R. Ruza, J. Hillier, S. Kjær, S. Frew, A. Monaghan, M. Bictash, P. C. Salinas, P. Whiting, J.-P. Vincent, E. Y. Jones and P. V. Fish, *J. Med. Chem.*, 2020, **63**, 12942–12956.

18 Y.-H. Yang and M. Shi, *Tetrahedron Lett.*, 2005, **46**, 6285–6288.

19 L. Wang, J. Cao, Q. Chen and M. He, *J. Org. Chem.*, 2015, **80**, 4743–4748.

20 S. Singh, L. K. Sharma, A. Saraswat, I. R. Siddiqui, H. K. Kehri and R. K. P. Singh, *RSC Adv.*, 2013, **3**, 4237–4245.

21 L. Green, K. Livingstone, S. Bertrand, S. Peace and C. Jamieson, *Chem. Eur. J.*, 2020, **26**, 14866–14870.

22 G.-Z. Lu, Y.-M. Jing, H.-B. Han, Y.-L. Fang and Y.-X. Zheng, *Organometallics*, 2017, **36**, 448–454.

23 J. Grover, N. Bhatt, V. Kumar, N. K. Patel, B. J. Gondaliya, M. Elizabeth Sobhia, K. K. Bhutani and S. M. Jachak, *RSC Adv.*, 2015, **5**, 45535–45544.
